# Triterpenes in breast cancer: a systematic review of preclinical evidence in rodents

**DOI:** 10.3389/fphar.2025.1623772

**Published:** 2025-11-14

**Authors:** Alexandra Prodea, Andreea Munteanu, Mihaela Jorgovan, Oana Batrina, Marius Mioc, Irina Soica, Cristina Trandafirescu, Codruta Soica

**Affiliations:** 1 Faculty of Pharmacy, Victor Babes University of Medicine and Pharmacy, Timisoara, Romania; 2 Research Center for Experimental Pharmacology and Drug Design (X-Pharm Design), Victor Babes University of Medicine and Pharmacy, Timisoara, Romania; 3 Medical School, University College London, London, United Kingdom

**Keywords:** triterpene, triterpenoid, breast cancer, rodents, mice, rat, *in vivo*, anticancer

## Abstract

**Introduction:**

Breast cancer poses a significant health problem for women worldwide due to late-stage diagnosis, toxicity of standard therapy and drug resistance. Several therapeutic alternatives, including triterpenes, show promising therapeutic potential and reduced toxicity *in vitro* and *in vivo* models.

**Methodology:**

We aimed to systematically review the data provided by rodent models of breast cancer regarding the anticancer effect, mechanisms of action and safety of triterpenes to assess if clinical translation to human studies is supported by current evidence. After a two-phase screening process, our search of PubMed/Medline, Web of Science (WOS) and Scopus databases yielded 163 articles that were included in the analysis.

**Results and discussions:**

Triterpenes were used in free form, semisynthetic derivatives (triterpenoids), cotreatment with other drugs or formulated as liposomes, micelles and nanoparticles (NPs). The vote-counting analysis showed a superior effect of triterpenes compared to controls in terms of tumor volume and weight reduction, findings also confirmed by a sensitivity analysis. We also searched for possible sources of heterogeneity in the studies assessed by analyzing several subgroups, which provided valuable information. They exerted their effect through various mechanisms such as apoptosis induction, metastasis and angiogenesis inhibition and decreased several cancer biomarkers such as ki-67, proliferating cell nuclear antigen (PCNA) and matrix metalloproteinases (MMP). The toxicity assessment revealed that triterpenes have in general, insignificant or absent toxicity, with only a small number of studies reporting serious side effects such as leukopenia, hepatotoxicity and mortality at specific doses that were reversed in some cases by the use of carriers, which hold the potential to enhance the therapeutic effect of triterpenes while reducing their systemic toxicity.

**Conclusion:**

We concluded that the current *in vivo* evidence does not support the clinical translation of triterpenes for the treatment of breast cancer due to methodological and clinical heterogeneity as well as the lack of toxicity data in a significant number of studies. Nonetheless, this field holds great potential for clinical translation, which could be attained through more rigorous methodologies and the collection of comprehensive experimental data.

## Introduction

Triterpenes are a diverse group of natural products with a complex structure and significant therapeutic potential. Although they can be found in marine animals or fungi, triterpenes are notably abundant in plants, with more than 20,000 unique structures identified so far (Goddard et al., 2024). Numerous plants play a role in traditional medicine for treating various health conditions, with their uses and preparations supported by scientific research (Joshi, 2023). Due to their diverse medicinal properties, triterpenes can exhibit anticancer, anti-inflammatory, antiviral, antioxidant, antibacterial, and antifungal activities (Gill and Kumar, 2016).

Triterpenes are widely investigated for their anticancer activity, as well as their potential use in cancer treatment and prevention (Nistor et al., 2023). In contrast to traditional chemotherapeutic drugs with high toxicity, relatively non-toxic phytochemicals like triterpenes are more suitable for long-term disease management (Li et al., 2020). Mechanisms that might be responsible for their anticancer activity are cytotoxicity, apoptosis regulation, interference with angiogenesis and dedifferentiation, metastasis inhibition and DNA polymerase blocking (Ghante and Jamkhande, 2019). Moreover, combining bioactive phytochemicals with chemotherapy has demonstrated a synergistic effect (Li et al., 2020).

Breast cancer is a major health threat to women worldwide. Its severity lies in the fact that it is frequently diagnosed in advanced stages, long after it has first developed. As a result, the disease spreads extensively, making treatment more challenging (Hussein et al., 2024). Breast cancer treatments, such as surgery, radiation, hormonal therapy, and chemotherapy, often come with significant side effects. Due to their limited effectiveness, relapse rates are rising, with varying morphological and molecular characteristics. Despite advances in research, challenges remain, especially with treatment side effects and drug resistance (El-Nashar et al., 2022). Moreover, there is an urgent need for natural treatments that can help limit tumor progression, enhance quality of life, and prolong patient survival (Aly et al., 2024).

According to scientific literature, the effectiveness of triterpenoids isolated from different plant species, such as masticadienolic acid, astragaloside IV (AS-IV), oleanolic acid (OA), ursolic acid (UA), ginsenoside Rh2 (Rh2), cucurbitacin B (CuB), saikosaponin D (SsD), asiatic acid (AA), and others (Table 1), has been extensively evaluated using various techniques. These have shown cytotoxicity against breast cancer, both *in vitro* and *in vivo*, through different mechanisms, including inhibiting cell proliferation, reducing mitochondrial membrane potential, suppressing glycolysis (downregulating LDH-A, c-Myc, and PDK1), inducing apoptosis (caspase activation, Bcl-2 modulation, ROS production, DNA damage), causing cell cycle arrest at the G0/G1 phase, modulating PI3K/AKT/mTOR and Beclin-1 pathways, reducing nitric oxide (NO) and TNF-α production, inhibiting STAT3, FAK, and MMPs, and enhancing Cav-1/NF-κB/c-Myc activity, supporting their potential as therapeutic agents (Aly et al., 2024).

**TABLE 1:** Triterpenes administered in free form in rodent models of breast cancer.

Pentacyclic triterpenes			
Triterpene	Structure	Triterpene	Structure
18β-glycyrrhetinic acid (GA)	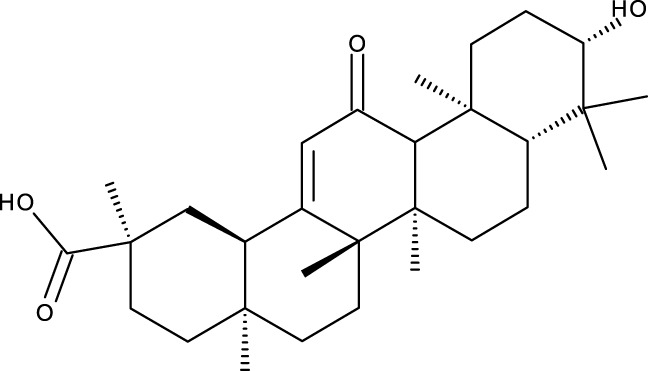	AG36	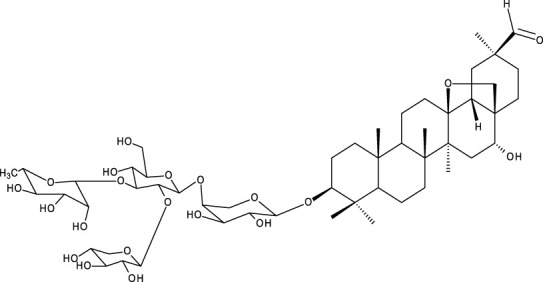
Anemoside A3	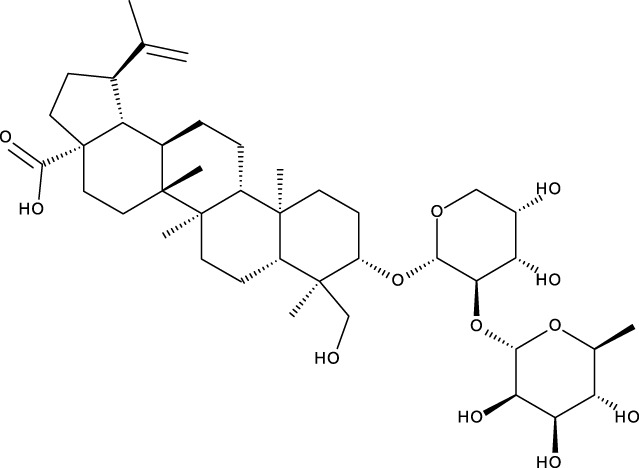	Arnidiol	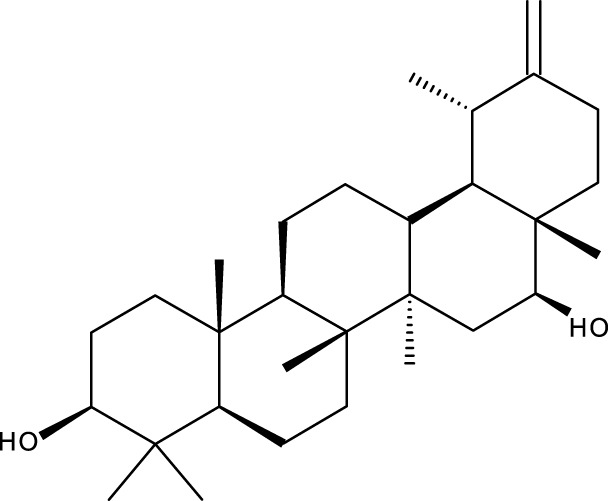
AA	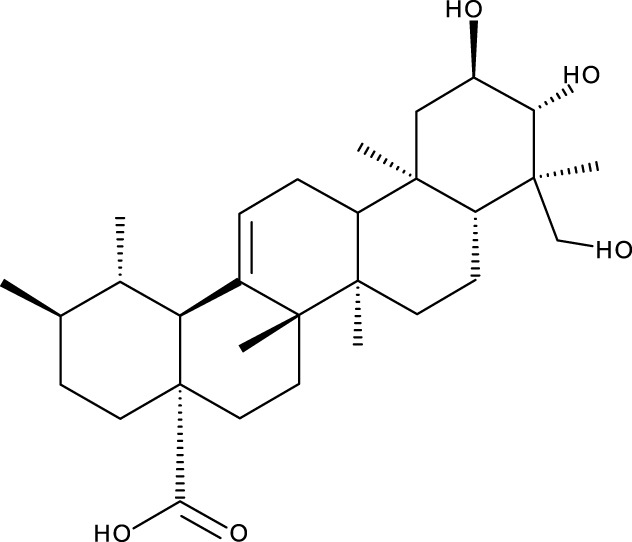	Betulin (Bet)	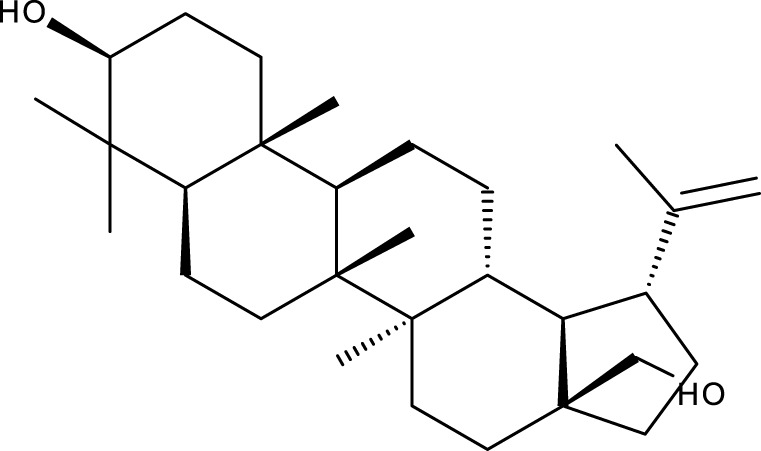
Betulinic acid (BA)	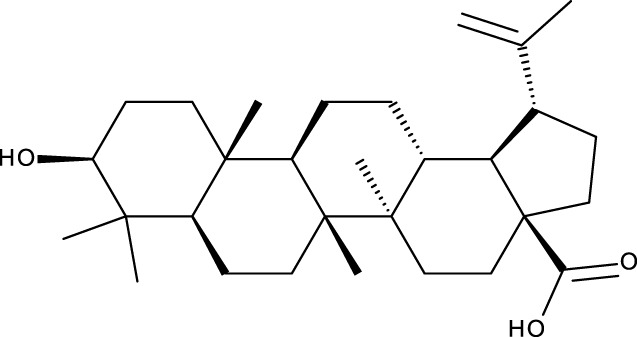	Celastrol (Cel)	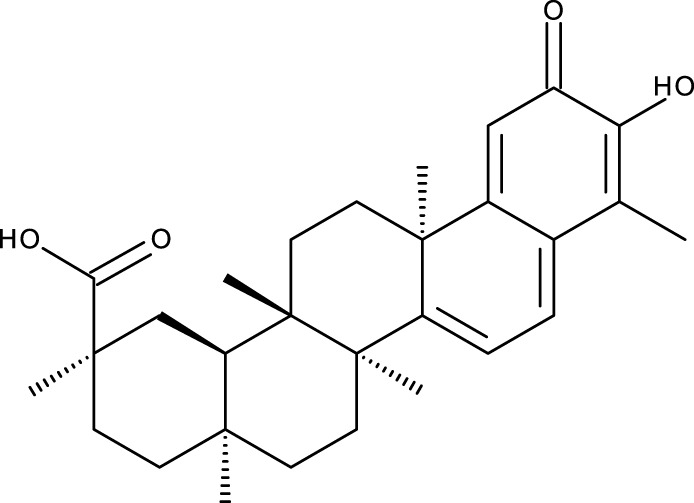
Epifriedelinol	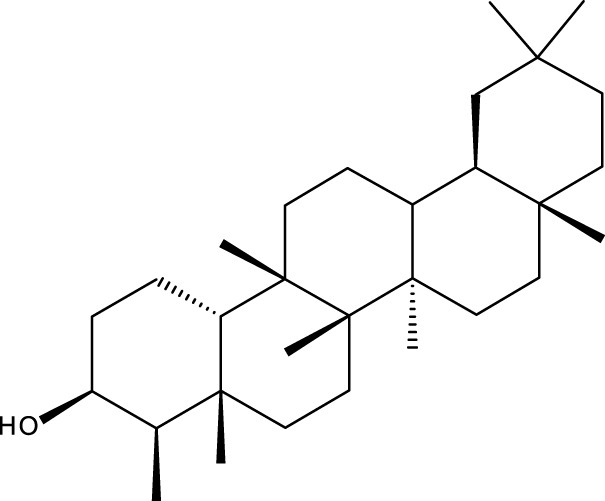	Esculentoside A	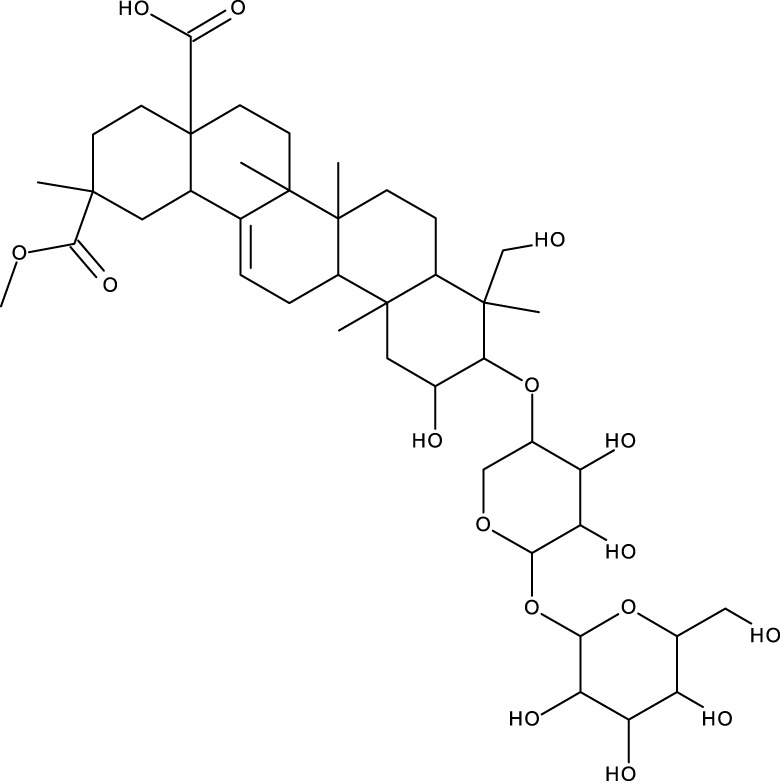
OA	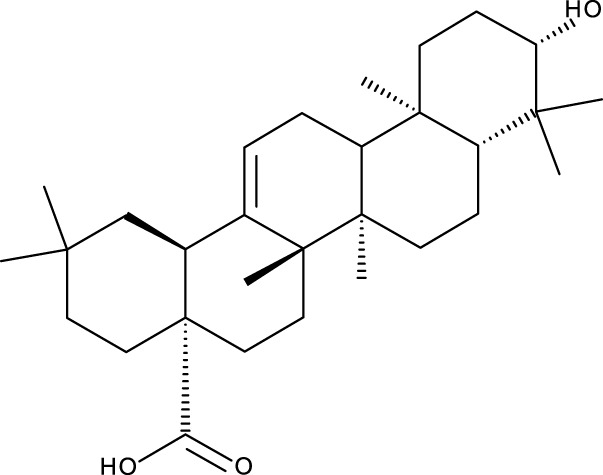	Platycodin D	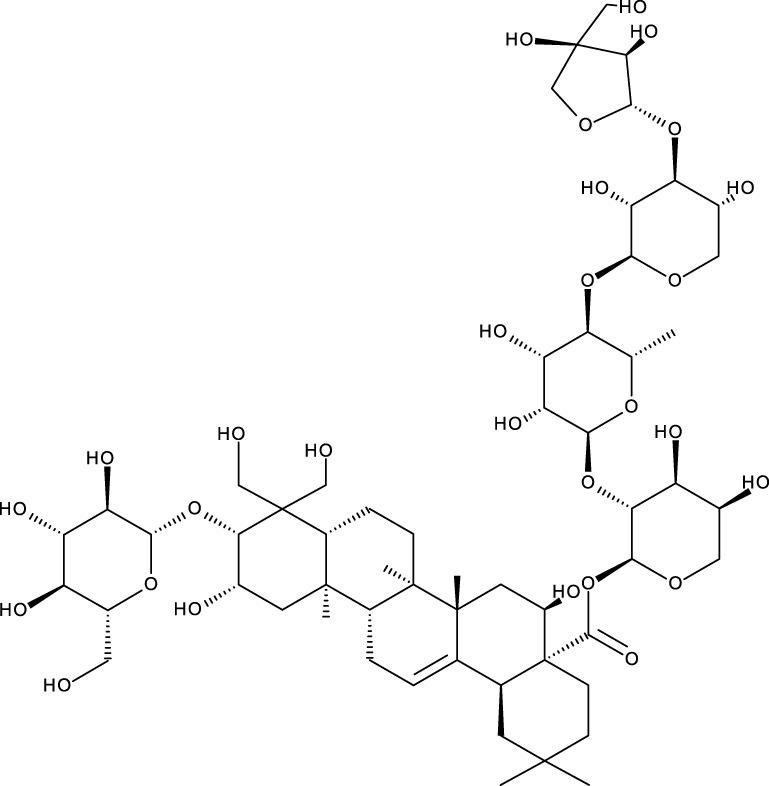
Pristimerin	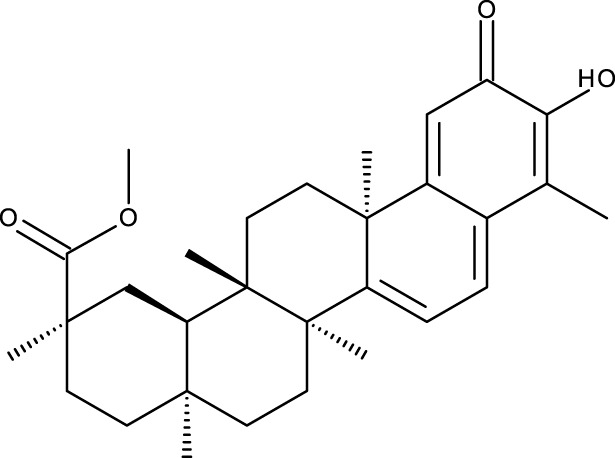	Pygenic acid A	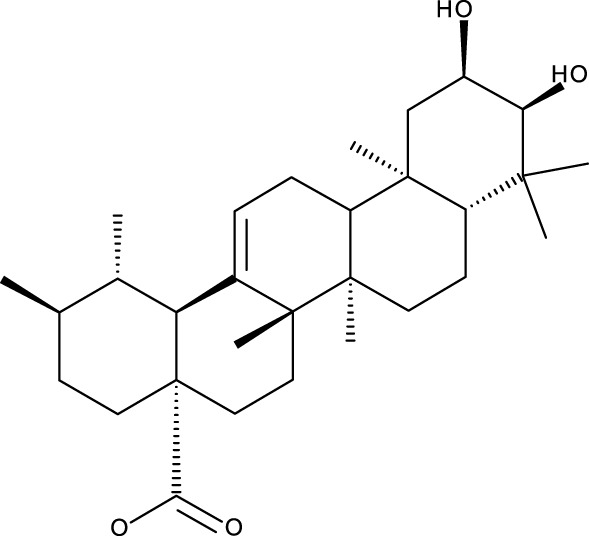
Saikosaponin A (SsA)	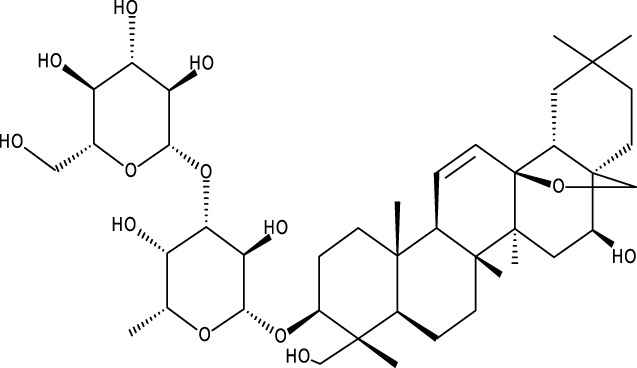	SsD	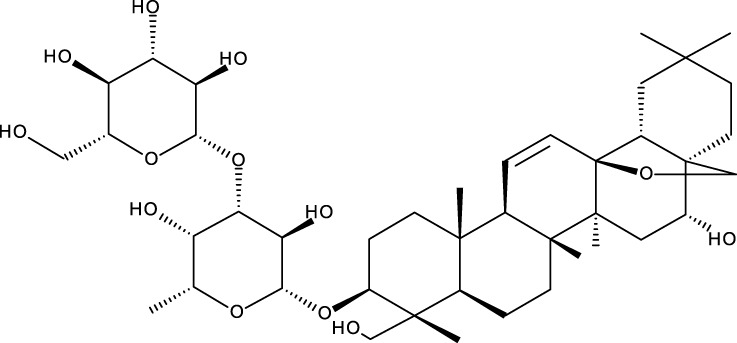
Tubeimoside-1	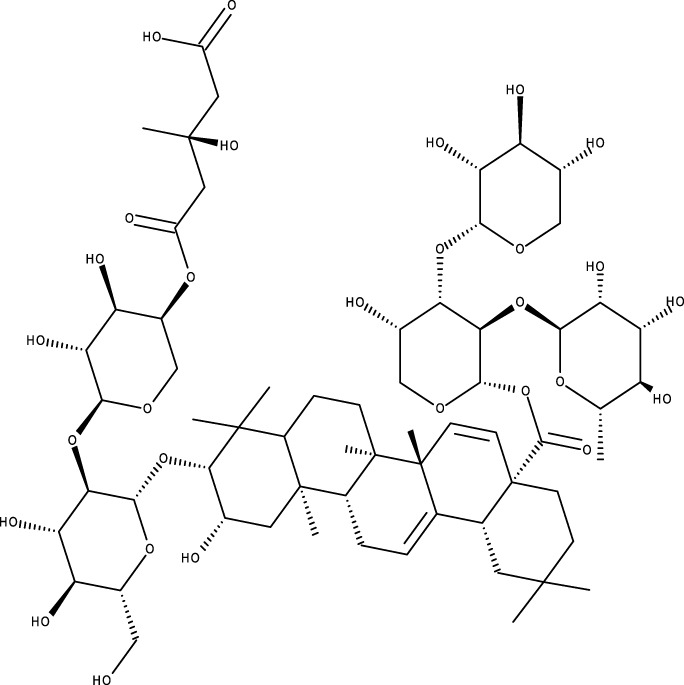	Soyasaponin Ag	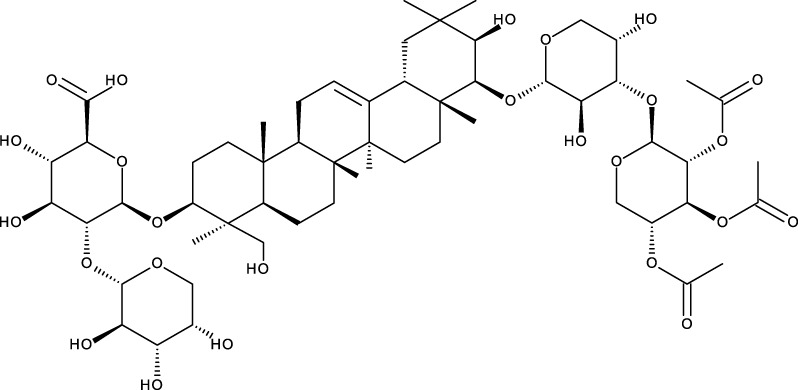
UA	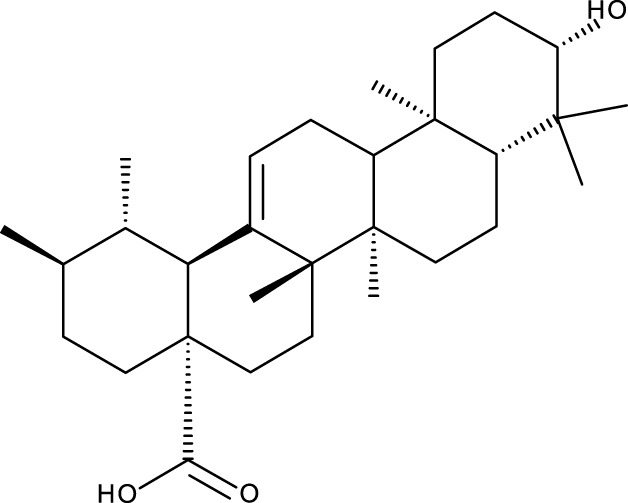		

Tetracyclic Triterpenes			
Triterpene	Structure	Triterpene	Structure
20(S)-protopanaxadiol (20(S)-PPD)	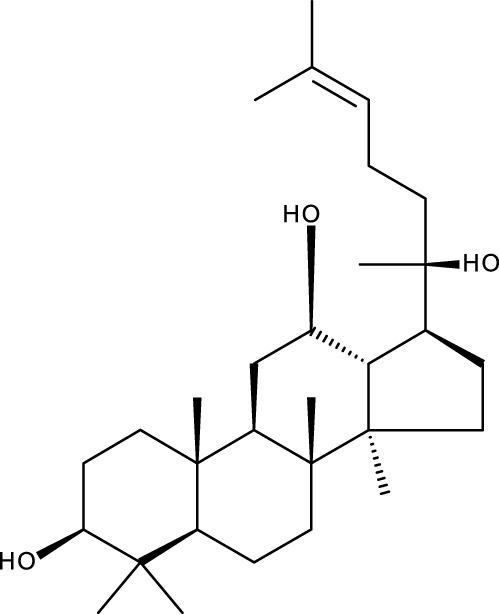	20(S)-Protopanaxatriol	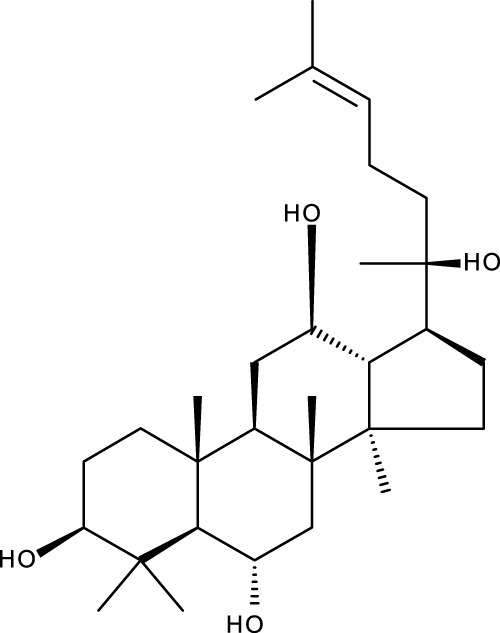
AECHL-1	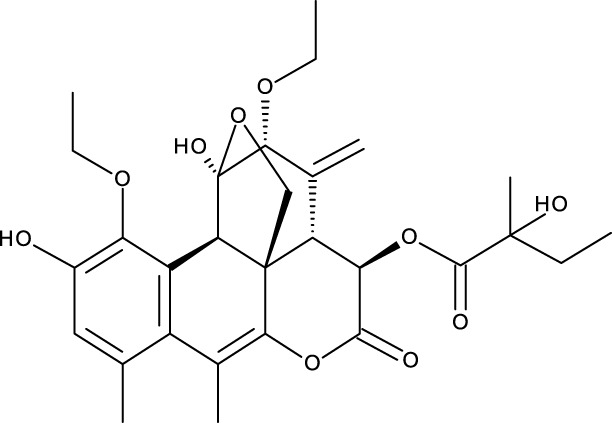	Actein	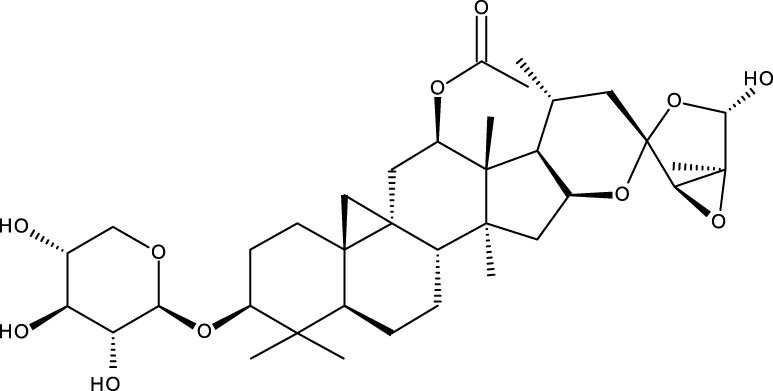
AS-IV	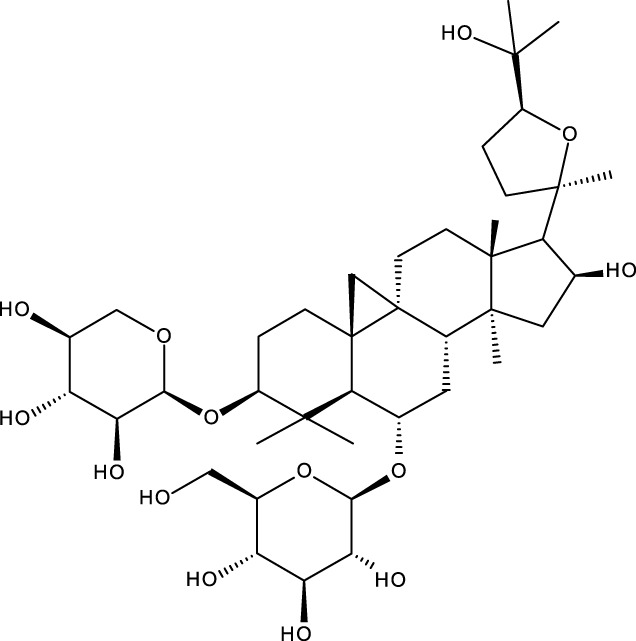	CuB	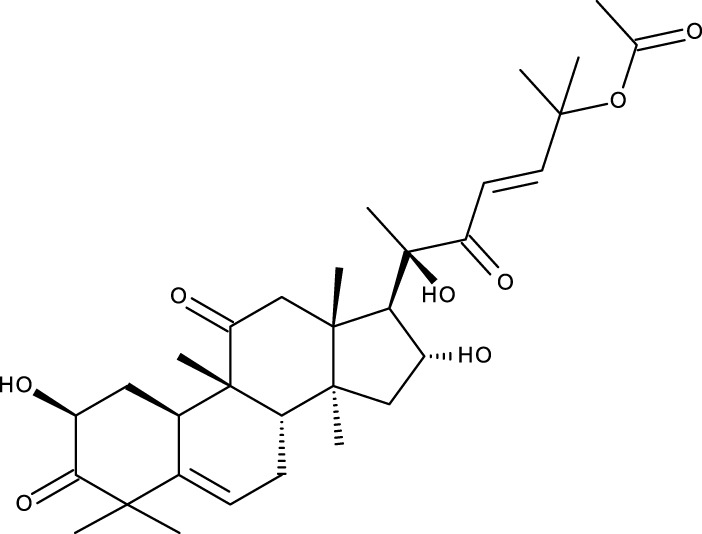
Frondoside A	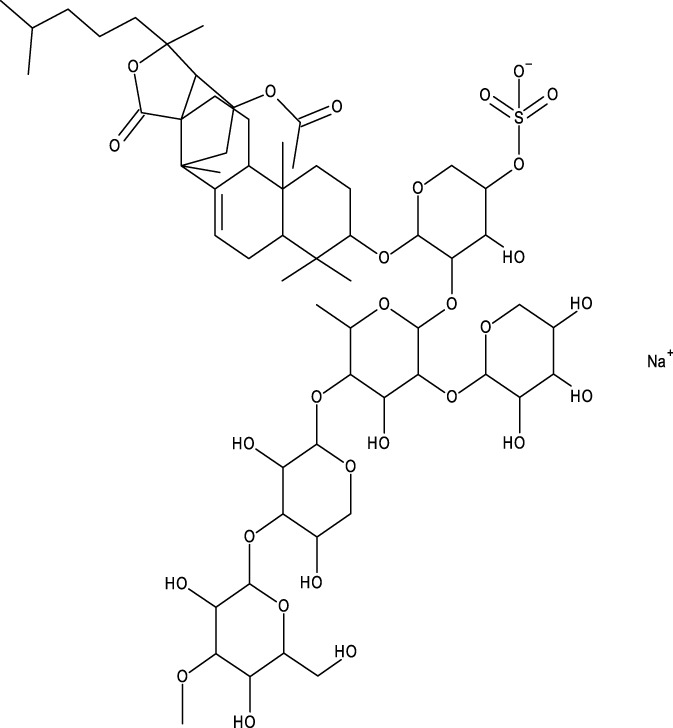	Ginsenoside Rg5 (Rg 5)	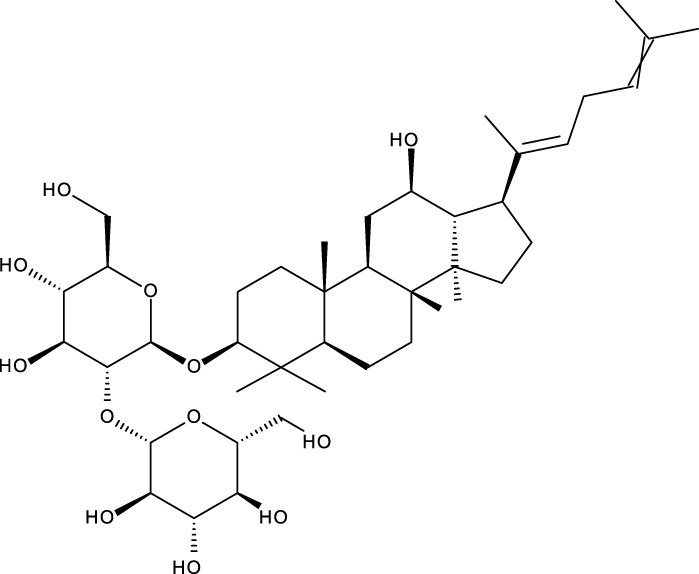
Rh2	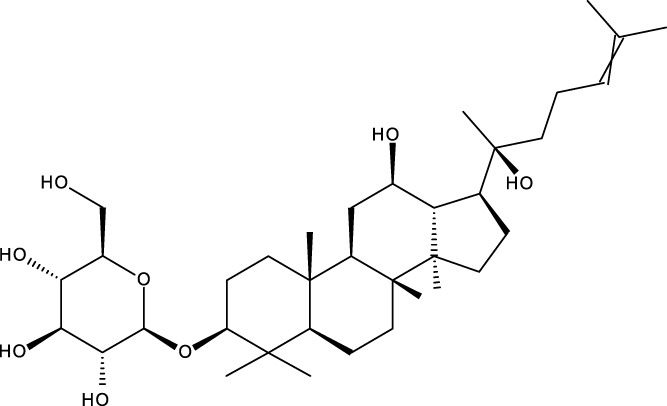	ginsenoside 20(S)-25-methoxyl-dammarane-3b, 12b, 20-triol (25-OCH3-PPD)	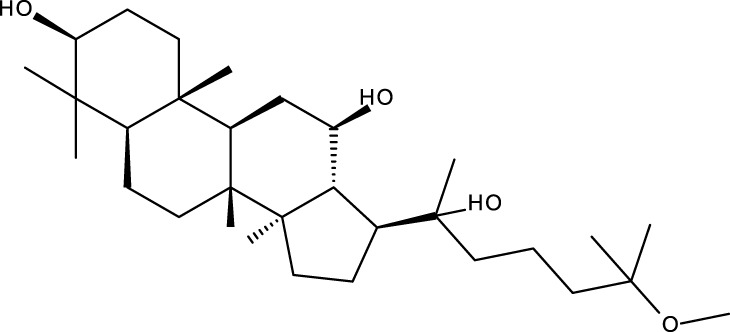
Ginsenoside CK (CK)	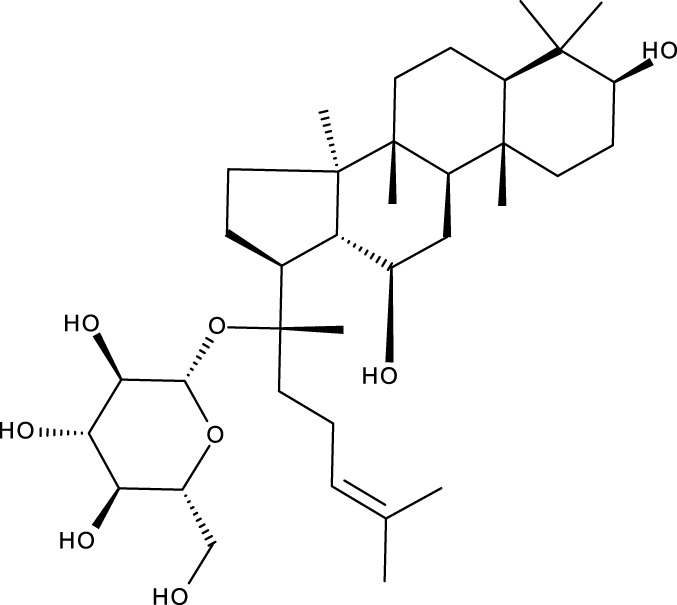	Ginsenoside Rd (Rd)	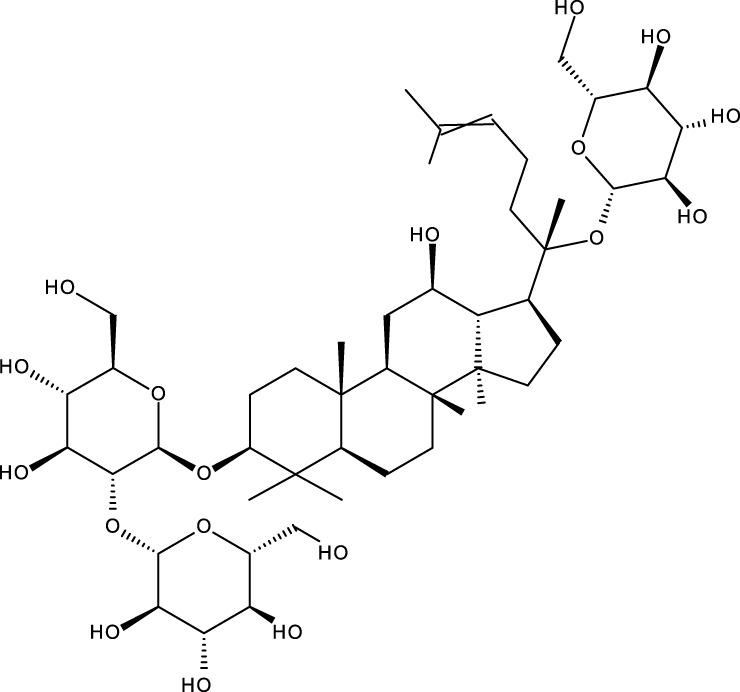
Ginsenoside Rg1 (Rg1)	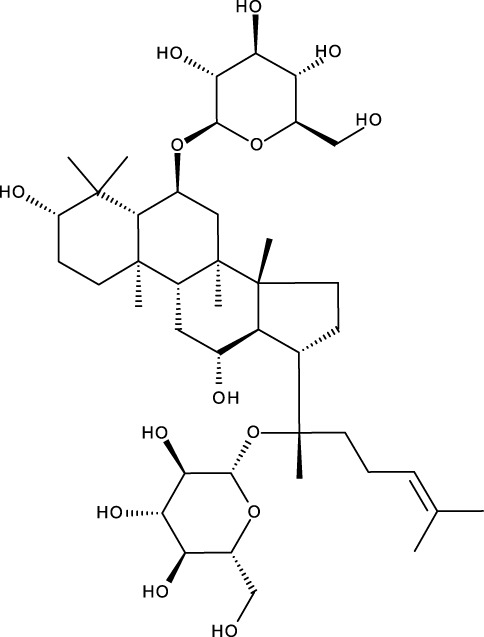	Ginsenoside Rg2 (Rg2)	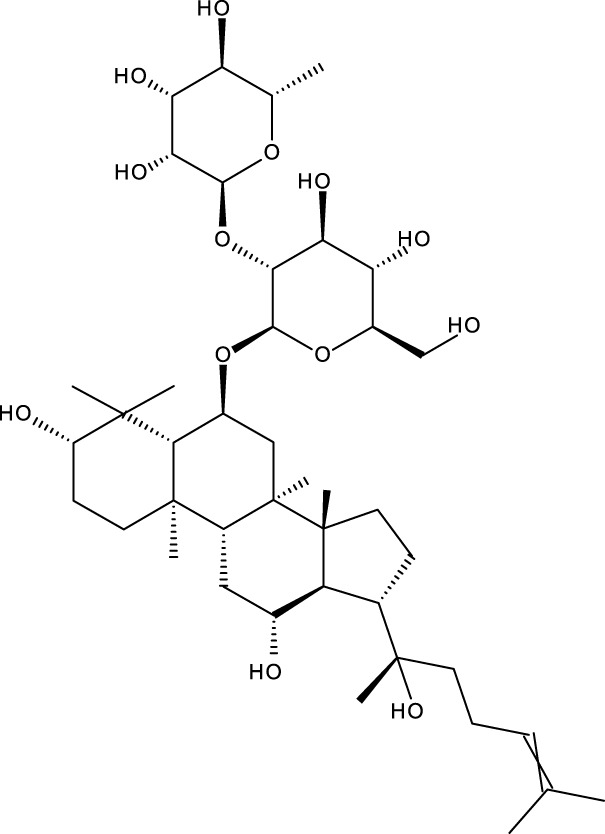
Ginsenoside Rg3 (Rg3)	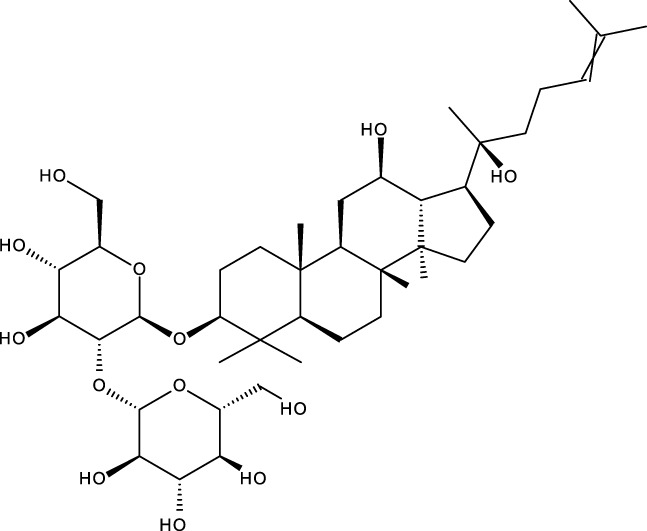	Ginsenoside Rh1 (Rh1)	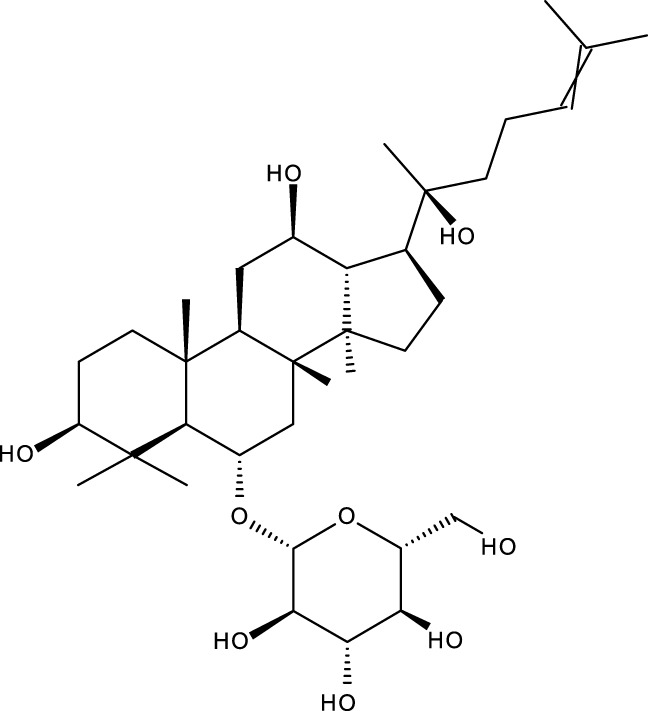
Ginsenoside Rh4 (Rh4)	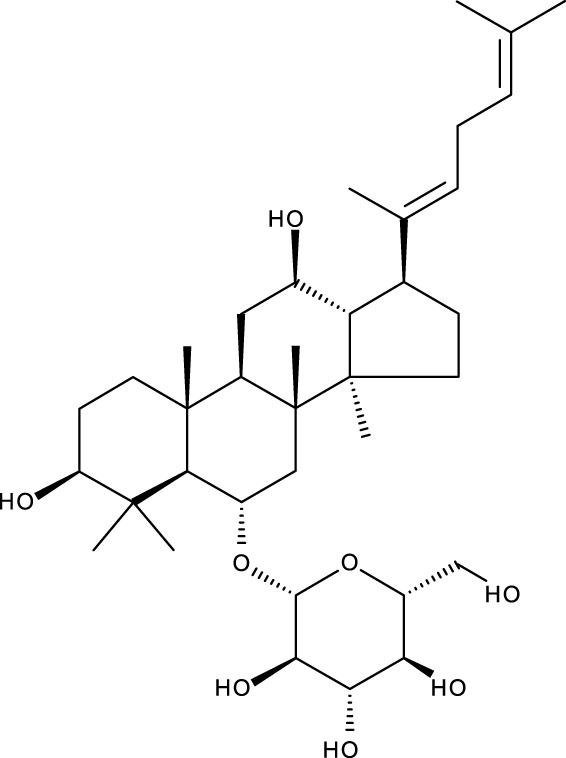	Ginsenoside Rk1 (Rk1)	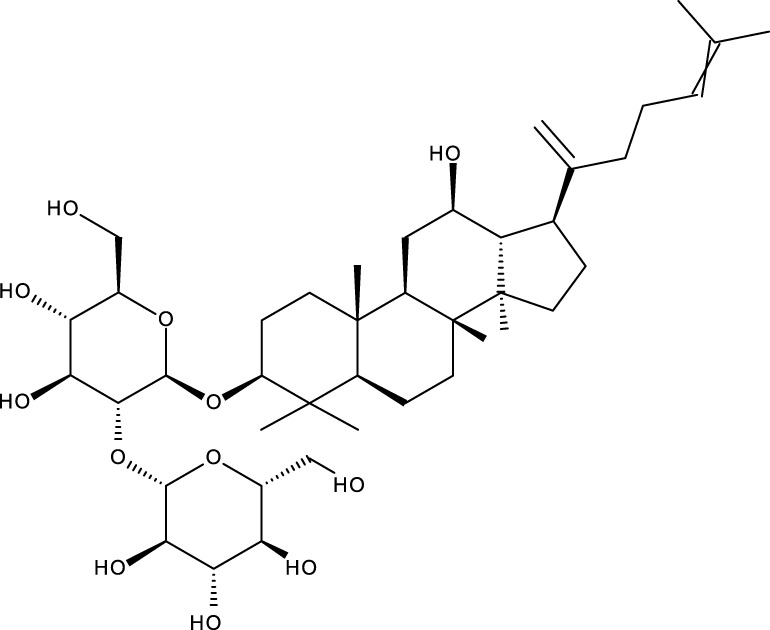
Gypensapogenin H	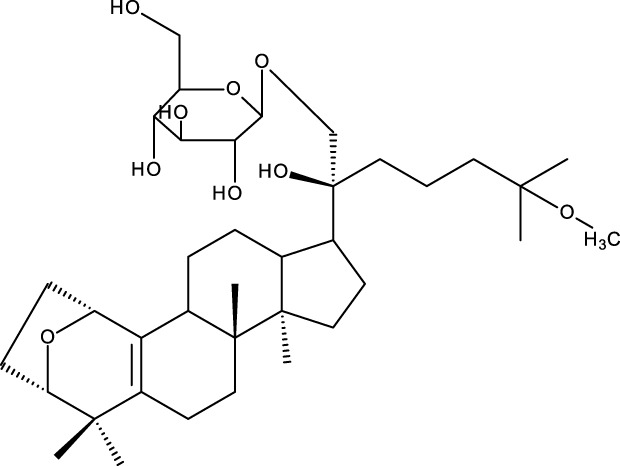	JSI-124 (cucurbitacin I)	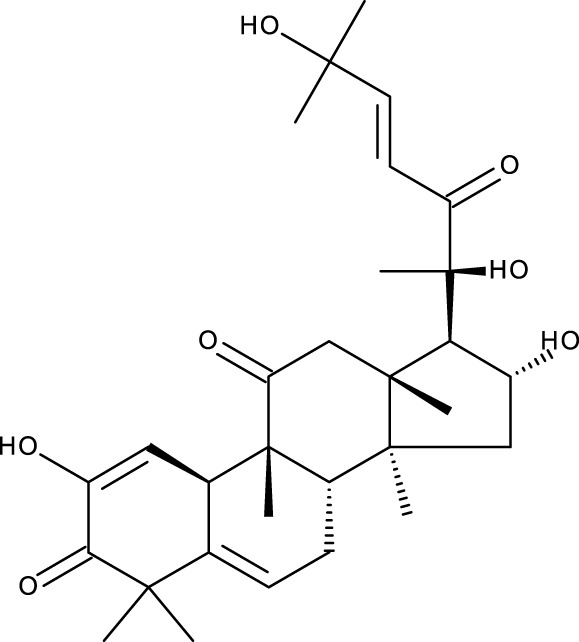
Panaxadiol	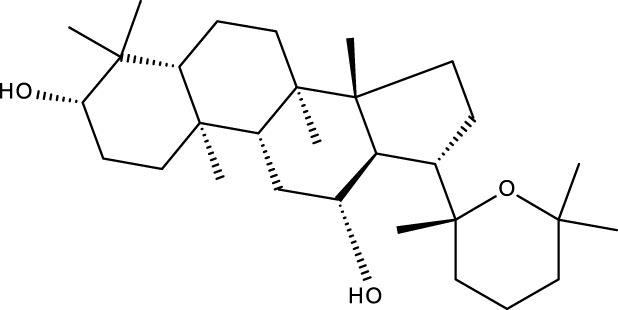	Sipholenol A	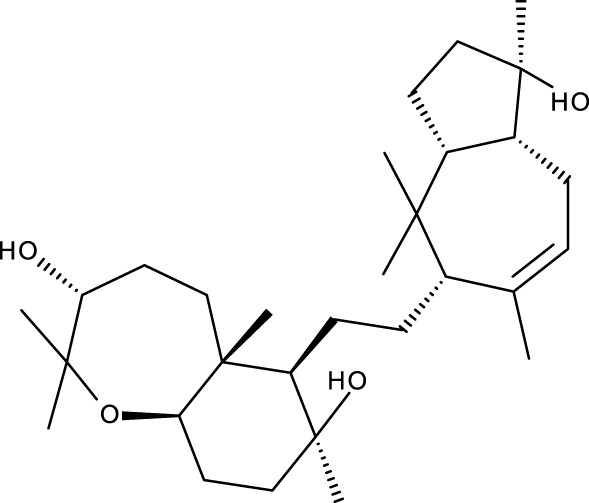
TSN (Toosendanin)	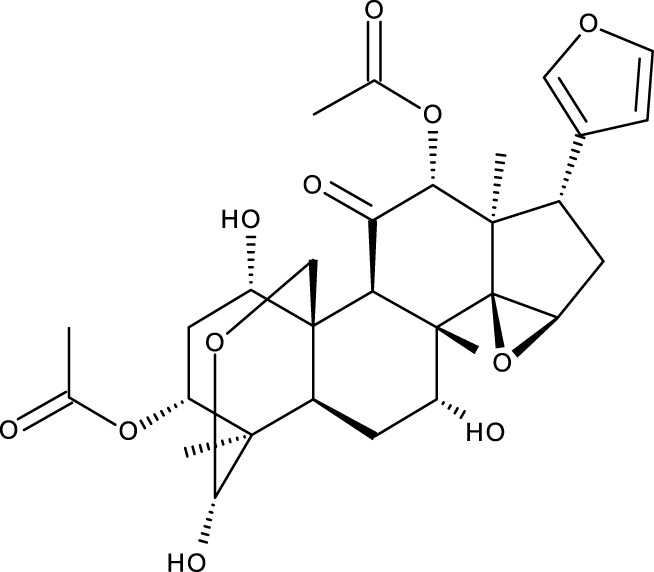	ITSN (Isotoosendanin)	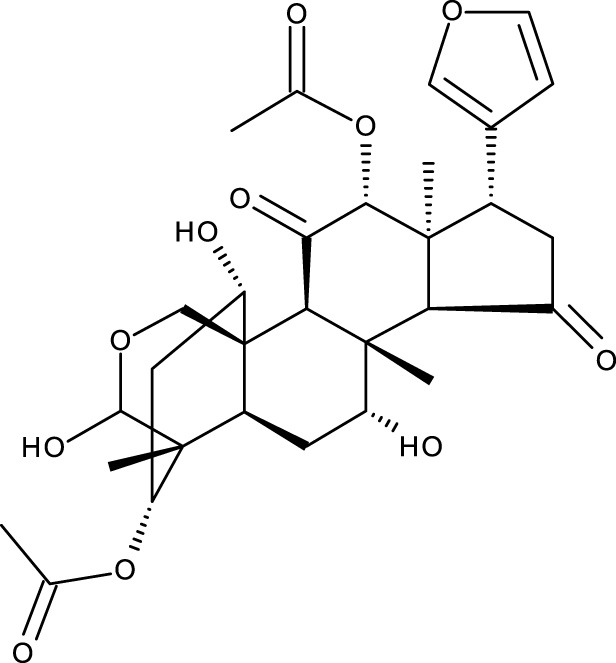

According to the last ALLURE report issued by the European Union in 2022, approximately 250,000 rodents (74% of the total animals) were used for scientific purposes (European Commission, 2025). Despite the ethical dilemmas surrounding animal use in experiments (Kiani et al., 2022) and the available alternatives, such as computer models, *in vitro* cell and tissue cultures, alternative organisms like prokaryotes, protists, fungi, lower vertebrates (e.g., *Danio rerio*/zebrafish), invertebrates (e.g., *Drosophila melanogaster*) or microorganisms (*e.g., Saccharomyces cerevisiae*) (Doke and Dhawale, 2015), *in chemico* and *ex vivo* approaches (van der Zalm et al., 2022), animal experimentation remains invaluable to medical research, enabling the study of biological interactions within a living organism. Mice have revolutionized research by serving as a powerful model for evaluating gene function and understanding the molecular mechanisms underlying cancer pathogenesis *in vivo.* They offer several key advantages over other model systems: their small size, low maintenance costs, rapid reproduction, and the ability to undergo genetic manipulation, an essential feature for replicating the pathophysiology of human cancers (Cheon and Orsulic, 2011).

The current systematic review aims to investigate if the current evidence obtained from studies on breast cancer in rodents supports the further investigation of triterpenes as drug candidates in humans, or if new studies are needed to improve certain areas of knowledge. To the best of our knowledge, this is the first systematic review to analyze the *in vivo* evidence of triterpenes in breast cancer. This subject was only partially addressed by previous narrative reviews that focused on the *in vitro* and *in vivo* effects of only oleanane derivatives (Parikh et al., 2014) or included all triterpenes but for limited time frames: 2017–2023 (Aly et al., 2024) and until 2010 (Bishayee et al., 2011).

## Methodology

### Generating the main research question

The main question of this systematic review was: In rodent models of breast cancer, do triterpenes demonstrate superior therapeutic outcomes compared to a control group, suggesting potential clinical efficacy in human breast cancer patients?

The research question was formulated using the Population-Intervention-Comparator-Outcomes (PICO) framework. The elements of the framework were defined as follows: Population (rodents used to model breast cancer, regardless of the induction method used), Intervention (triterpenes or triterpenoids used alone or in combination solubility enhancers), Comparator (rodents treated with other active controls, inactive vehicles or rodents undergoing no treatment) and Outcomes (primary outcome: tumor volume, tumor weight, data regarding the mechanism of action; secondary outcome: side effects observed, modifications of laboratory results, histopathological toxicity).

### PRISMA statement and international prospective register of systematic review (PROSPERO) protocol registration

This systematic review was conducted according to the Preferred Reporting Items for Systematic reviews and Meta-Analyses (PRISMA) statement (Preferred Reporting, 2024). The protocol was registered in the PROSPERO database with the registration number CRD42024591602 (Prodea et al., 2025). A *post hoc* modification to the PROSPERO protocol was made to include studies investigating the synergistic effects of triterpenes and approved or studied chemotherapeutics to provide a more comprehensive overview of their therapeutic potential in breast cancer.

### Search strategy

We searched three databases (WOS, PubMed/Medline and Scopus) using the following terms: “triterpene”, “triterpenoid”, “betulinic acid”, “ursolic acid”, “lupeol”, “ginsenoside”, “glycyrrhetinic acid”, “celastrol”, “cucurbitacin”, “oleanolic acid”, “saikosaponin”, “lanosterol”, “maslinic acid”, “breast cancer”, “mammary carcinoma”, “breast tumor”, “mammary gland cancer”, “breast adenocarcinoma”, “mammary neoplasm”, “rodent”, “mice”, “mouse”, “rat”, “hamster”, “guinea pig”, “*in vivo*”, “preclinical research”, “xenograft”, “orthotopic model”, “murine model”, “metastasis model”, “animal model”. Finally, we searched the reference lists of three relevant reviews (Aly et al., 2024; Bishayee et al., 2011; Parikh et al., 2014). The search was restricted to articles written in English, and no limits were imposed for the publication date. The complete search strategy, including the search sequences used, is available in the Supplementary Material.

### Selection of studies

Two independent reviewers searched the databases on 14 November 2024 using the predetermined search sequences, comparing the total number of articles to be retrieved. After the literature search, the reference lists from the three databases were imported into the Mendeley software, and duplicates were removed using the appropriate option in Mendeley. Afterwards, the article list was exported to Zotero software, and then into an Excel spreadsheet for further analysis. Articles were included in the analysis if the following criteria were met: 1) comparative studies (studies with at least one separate control group); 2) rodents (mice, rats, guinea pigs, hamsters) used to model breast cancer, regardless of the induction method used; 3) triterpenes or triterpenoids (semisynthetic derivatives of triterpenes) used as pure substances (purified from extracts or obtained through chemical synthesis), alone or in combination with other chemotherapeutics or solubility enhancers (cyclodextrins, liposomes, …). The articles were excluded if the following criteria were met: 1) other language than English; 2) secondary studies; 3) humans, other species of animals (including *in vitro* studies); 4) plant extracts (alone or in combination with solubility enhancers); 5) non-comparative studies (studies without a control group). For the initial screening, two reviewers (A.M. and A.P.) analyzed the articles’ title and abstract. Any disagreements were resolved through discussion with a third reviewer (M.M.). Then the full-text articles were assessed by two reviewers (A.M. and A.P.). Similarly, any disagreements were resolved through discussion with a third reviewer (M.M.).

### Data extraction

Two independent researchers (M.J. and O.B.) performed the data extraction in Google Spreadsheets. Any discrepancies observed were resolved through discussions after double-checking the source. The following information was extracted from the articles: 1) study characteristics (name of the first author and year of publication; title of the article; study type and country); 2) experimental model (animal model characteristics -sex, type and age; type of breast cancer inoculated and tumor inoculation method; triterpene/triterpenoid tested; administration route, dosage, frequency and duration of treatment; 3) primary outcome (tumor weight for triterpene and control; tumor weight trend; tumor volume for triterpene and control; tumor volume trend; information regarding the mechanism of action); 4) secondary outcome (side effects observed; modifications of laboratory results; histopathological toxicity). In studies where multiple time-points were reported for an experiment, the final time-point of the experiment was selected for data extraction, so that the data reflect the end stage of the treatment.

### Bias analysis

The risk of bias was assessed by two independent researchers (A.M. and A.P.) using the Risk of Bias Tool for Animal Studies elaborated by the Systematic Review Center for Laboratory Animals (SYRCLE RoB Tool) (Hooijmans et al., 2014). Using this method, we evaluated six types of bias: selection bias, performance bias, detection bias, attrition bias, reporting bias, and other biases (the report of ethical approval for the animal study). For each category of bias assessed, we used three options: high (showing a high risk of bias), low (representing a low risk of bias), and unclear (where inadequate data was provided). The risk of bias analysis was made using Google Spreadsheet and the risk of bias figures were made using ROBVIS (McGuinness and Higgins, 2021).

### Data synthesis

A meta-analysis was not considered appropriate for this review due to the high diversity of included studies. In particular, endpoint heterogeneity limited statistical pooling as different studies used incompatible measurements of tumor growth, such as tumor weight and tumor volume. Moreover, in many studies, these metrics were only presented in graph form, limiting the extraction of raw data necessary for the meta-analysis. While variance data were available in the majority of the studies, the difference in endpoint measurement times of short-term *versus* long-term studies also made it impossible to statistically analyze the results. Instead, a semi-quantitative synthesis of tumor weight and volume using vote-counting for direction of effect was performed to summarize the results. For studies with multiple treatment groups, we used the any positive approach, where if any of the treatment arms showed a statistically positive effect, the study was categorized as having a positive effect. This approach was used to prevent double-counting the studies reporting a positive effect, offering a concise summary of the evidence.

To explore heterogeneity sources in the study results we performed several subgroup analyses on: i) free triterpenes *versus* terpenoids; ii) the use of delivery systems; iii) monotherapy *versus* cotreatment; iv) tumor models and v) route of administration. For each subgroup, the same vote-counting analysis for tumor weight and volume used for the primary analysis was applied.

### Sensitivity analysis

We conducted a sensitivity analysis to assess the robustness of our findings by analyzing the studies with a low risk of bias in key domains (sequence generation, allocation concealment, blinding of participants and personnel and blinding of outcome assessment). However, due to significant methodological shortcomings identified in our risk of bias assessment, none of the articles were considered to have a low risk of bias in all key domains. Hence, for this sensitivity analysis, we considered a study to have a low risk of bias if it reported a random sequence generation, a crucial step in reducing selection and overall bias. We performed a vote-counting analysis for tumor weight and volume of the 2 studies that fulfilled our criteria and compared the results to our primary analysis of all the included studies.

## Results

### Studies included in the qualitative synthesis and analysis

**FIGURE 1:**
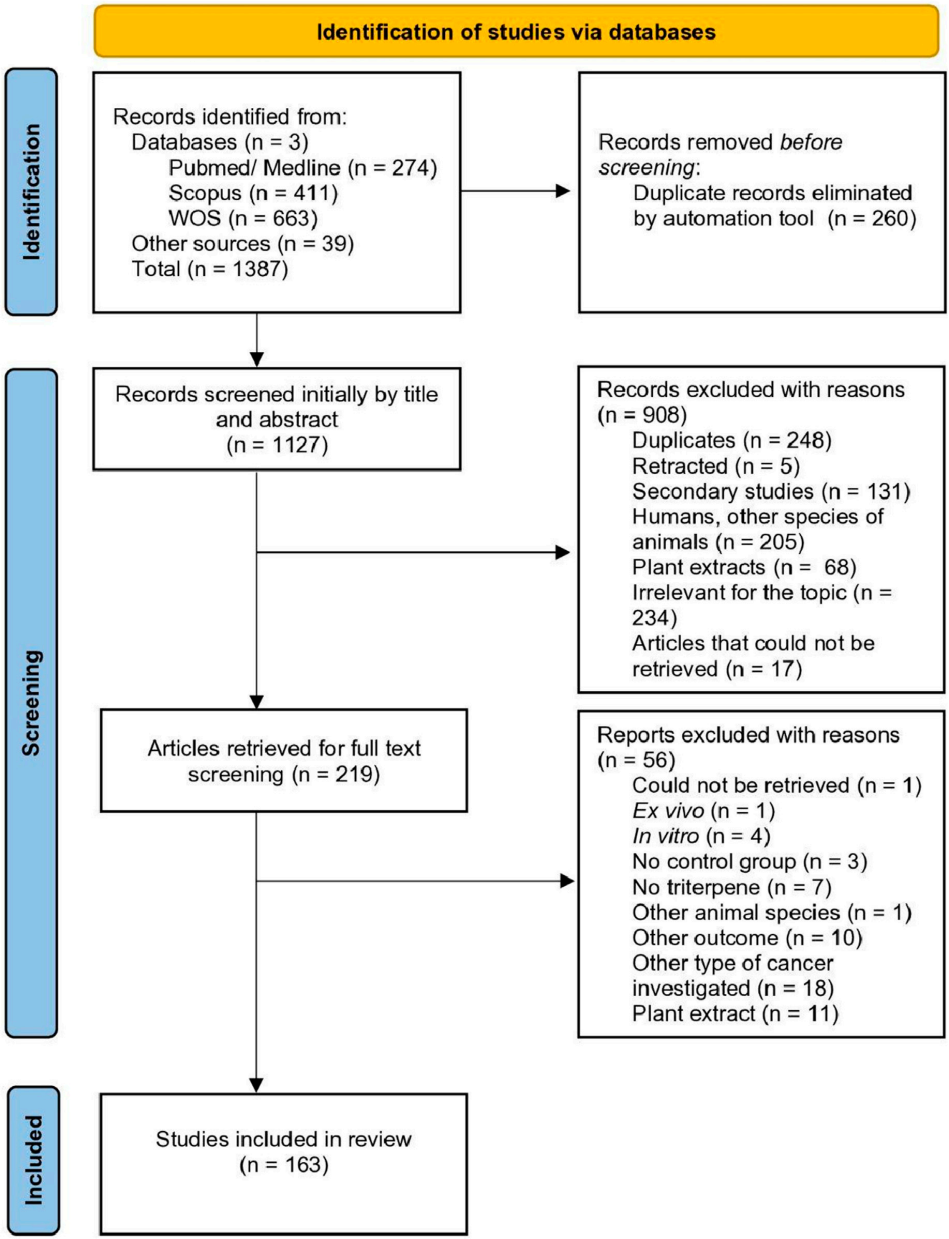
PRISMA diagram depicting the study selection process.

The diagram representing the search process for the review is depicted in Figure 1. Our search resulted in 1348 articles obtained from three databases (WOS, PubMed/Medline and Scopus) and 39 articles obtained from the manual search of relevant reviews on the topic. After duplicate removal and the two-phase screening, 163 articles were included in the systematic review.

### Studies characteristics

**FIGURE 2:**
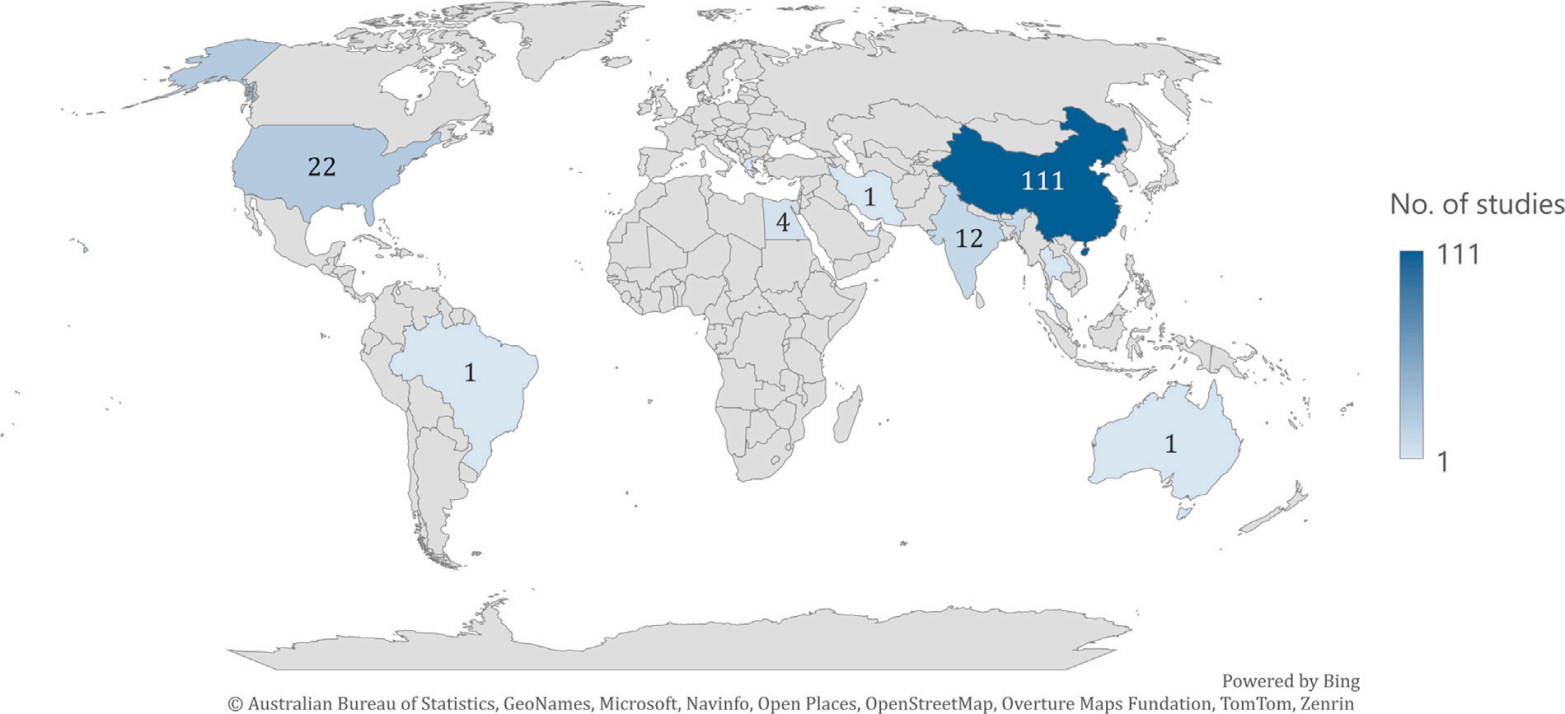
Distribution of articles across countries.

The eligible articles were published between 2003–2024 as follows: 2003 (n = 1); 2005 (n = 1); 2006 (n = 1); 2007 (n = 1); 2008 (n = 3); 2010 (n = 1); 2011 (n = 4); 2012 (n = 4); 2013 (n = 6); 2014 (n = 2); 2015 (n = 1); 2016 (n = 12); 2017 (n = 9); 2018 (n = 15); 2019 (n = 12); 2020 (n = 25); 2021 (n = 18); 2022 (n = 14); 2023 (n = 12) and 2024 (n = 22). The studies originated from different continents: Asia (n = 134; China n = 111; India n = 12; Korea n = 8; Iran n = 1; Thailand n = 1; United Arab Emirates n = 1); North America (n = 22; United States of America n = 22); Africa (n = 4; Egypt n = 4); Europe (n = 1; Greece n = 1); South America (n = 1; Brazil n = 1); and Australia (n = 1) (Figure 2). The study design was cross-sectional for all the articles included (Supplementary Material).

### Animal model

The animals used in the majority of the studies were in-bread species of mice (Balb/c mice, n = 75; C57BL/6 mice, n = 2; C3H/He mice, n = 1; albino mice, n = 1; Swiss Albino mice, n = 1) followed by immunodeficient mice species (Balb/c nu/nu (or nude) mice, n = 32; nude mice, n = 22; SCID mice, n = 4; NCr-nu/nu mice, n = 1; NMRI nude mice, n = 1; NSG mice, n = 1; NOD-SCID mice, n = 1; NMRI-Foxn1nu/Foxn1nu mice, n = 1); genetically engineered mouse models (PyMT mice, n = 2; MMTV-ErbB2/neu mice, n = 1; MMTV-neu mice,n = 1; MMTV-PyVT ± mice, n = 1; MMTV-Cre; p53 (+/−) mice, n = 1) and rats (rats, n = 1; Sprague Dawley rats, n = 6; albino rats, n = 1; Wistar rats, n = 1). Six studies used 2 mouse species for the experiments, Balb/c mice and nude mice (n = 2); Balb/c mice and C57BL/6 mice (n = 1); Balb/c nu/nu mice and Balb/c mice (n = 1); Brca1(Co/Co)) and MMTV-PyMT mice and Balb/c nu/nu mice (n = 1) (Supplementary Material). Regarding the sex of the animals, 136 used female, 3 used male, 1 used both female and male (study with two types of mice), 2 used female but did not specify the sex for the other mice species (study with two types of mice) and 22 did not specify the sex of the animals used. The age of the animals was between 3 and 10 weeks, reported by 112 studies, 2 mentioned the unclear term “adult”, while 49 studies did not report the age of the animals used (Supplementary Material).

The breast cancer animal model was obtained by several methods: using genetically modified mice (n = 6), by 7,12-dimethylbenz(a)anthracene (DMBA) induction (n = 9) and by the inoculation of murine (allografts) and human (xenografts) cancerous cells, such as 4T1 (n = 45); MDA-MB-231 (n = 32); MCF-7 (n = 22); 3T3/4T1 (n = 1); 4T1-fluc-red (n = 1); 4T1-Luc (n = 4); 4T1-Luc-GFP (n = 1); 4T1/rLu/GFP (n = 2); ALDH (high) BCSCs (n = 1); BT-474 (n = 1); BT20 (n = 1); BT474 (n = 1); EAT (n = 3); EMT6M (n = 1); FM3A (n = 1); MCF-7/ADR (n = 2); MCF-7/DOX (n = 1); MCF-7/T (n = 1); MCF10DCIS (n = 1); MDA-MB-231-Luc (n = 3); MDA-MB-231/GFP (n = 1); MDA-MB-361 (n = 1); MDA-MB-468 (n = 2); MMTV-Wnt-1 (from genetically modified mice; n = 2); SKBR-3 (n = 2) and SUM159 (n = 1). Fifteen studies used two or more different methods of cancer inoculation (Supplementary Material).

### Treatment

The compounds were tested in various forms, including triterpenes (n = 66) (Supplementary Material); triterpenoids (n = 18) (Table 2); cotreatment (n = 25) (Supplementary Material); liposomes (n = 12); micelles (n = 7) and NPs (n = 35) (Supplementary Material). When more than one form was tested in a study, we considered the most active form for the classification.

**TABLE 2:** Triterpenoids tested in rodent models of breast cancer.

Parent triterpene	Triterpenoid	Administration route, dosage, frequency, duration of treatment	Controls	Tumor weight trend compared with controls	Tumor volume trend compared with controls	Mechanism of action (*in vivo*)	Safety profile	References
BA	ester derivative of BA and dichloroacetate (Bet-CA)	Intratumorally; 1 mg/kg	Vehicle (not specified)	-	Decreases	Reduces lung metastasis, reduces PCNA expression and low BrdU uptake, increases ROS, angiogenesis inhibition	-	Saha et al. (2016)
Bet	lup-20 (29)-en-3β,28-di-yl-nitrooxy acetate (NBT)	i.p.; 100 mg/kg daily for 11 days	Saline, 5-fluorouracil, Bet	-	Decreases (vs. saline and betulin), similar (vs. 5-fluorouracil)	-	No significant toxicity	Yan et al. (2018)
Cel	Cel-based Proteolysis Targeting Chimeras: compound 6a	3 and 6 mg/kg (compound 6a), daily for 6 days	Vehicle (5% DMSO, 5% PEG300, 5% Tween 80, and 85% saline), Cel	Decreases	Decreases	-	No organ toxicity, reduced toxicity compared to Cel	Gan et al. (2024)
Cel	Lactoferin-Cel-DOX nanoconjugates	i.v.; 2.3 mg/kg DOX +4 mg/kg Cel, 3 times per week for 3 weeks	No treatment, Cel, DOX, Cel + DOX	-	Decreases	DecreasesNF-κB, TNF-α, COX-2 and ki-67 tumor biomarkers	-	Abdelmoneem et al. (2021)
CuB	Prodrug 1 of CuB	i.p.; 3, 5 and 10 mg/kg (prodrug), 3 mg/kg CuB daily for 14 days	Saline, tamoxifen	Decreases (vs. saline), similar (3 and 5 mg/kg vs. tamoxifen), decreases (10 mg/kg vs. tamoxifen)	Decreases (vs. saline), similar (3 and 5 mg/kg vs. tamoxifen), decreases (10 mg/kg vs. tamoxifen)	-	Reduced toxicity compared to CuB (which provoked the death of animals after the first day of treatment)	Suebsakwong et al. (2019)
GA	GA derivates	i.p.; 20 mg/kg and 40 mg/kg GA derivatives (9 days, 12 m) every other day for 15 days	DMSO, GA	Decreases	Decreases	Reduces lung, liver, brain and bone metastasis	-	Kallepu et al. (2020)
OA	2-cyano-3,12-dioxooleana-1,9 (11)-dien-28-oic acid (CDDO-Me)	p.o.; 27 and 81 ppm, daily for 14 days for the DMBA model, 7 days for MNU model	No treatment (used in both models), 5,6-benzoflavone (500ppm) (used in the DMBA model)	Decreases (vs. no treatment), increases (vs. 5,6-benzoflavone)	-	-	No significant toxicity (lower dose), increased toxicity: weight loss and increased mortality (high dose)	Lubet et al. (2016)
OA	CDDO-Me	p.o.; 50 mg/kg daily for 4 weeks	No treatment	-	-	Decreases CXCL12, CCL2 and MMP-9	-	Tran et al. (2012)
OA	CDDO-Me	i.p.; 5 mg/kg twice a week for 3 weeks	Saline	Decreases	Decreases	Inhibition of Wnt/β-catenin signaling pathway	No significant toxicity	Zhou et al. (2020)
OA	CDDO-Me	p.o.; 50 mg/kg daily for 6 weeks	No treatment	-	-	Reduces levels of ErbB2, p-ErbB2, cyclin D1, and gH2AX	No toxicity observed	Kim et al. (2012)
OA	CDDO-Me	p.o.; 50 mg/kg, daily for 8 weeks	No treatment	-	-	Decreases CD3^+^ T cells, inhibition of VEGF and IL-10, increases TNF-α	Weight loss	Ball et al. (2020)
OA	CDDO-Me	i.p.; 5 mg/kg every 3 days, 5 doses (spontaneous model); 2 mg/kg every 3 days, 10 doses (xenograft)	DMSO	-	Decreases	Decreases Rab 13 expression, reduced ALDH activity	-	Wang et al. (2022)
OA	methyl 3β-O-[4-(2-aminoethylamino)-4-oxo-butyryl]olean-12-ene-28-oate (DABO-Me)	Intratumorally; daily for 25 days	PBS	-	Decreases	-	-	Feng et al. (2020)
OA	methyl-25-hydroxy-3-oxoolean-12-en-28-oate (AMR-Me)	p.o.; 0.8; 1.2 mg/kg and 1.6 mg/kg; 3 times a week for 18 weeks (2 prior DMBA and 16 post DMBA)	No treatment	Decreases	-	Upregulation of Bax, BAD, CASP 3, CASP 7 and PARP, downregulation of Bcl-2 expression (in medium and high dose of AMR-Me groups)	No toxicity observed	Bishayee et al. (2013)
OA	AMR-Me	p.o.; 0.8; 1.2 or 1.6 mg/kg, 3 times per week for 18 weeks (2 weeks prior to DMBA and 16 weeks after DMBA)	No treatment	-	-	Suppresion of Wnt/β-catenin signaling pathway, decreases PCNA, angiogenesis inhibition	No significant toxicity	Mandal et al. (2013)
OA	AMR-Me	p.o.; 0.8; 1.2 or 1.6 mg/kg, 3 times per week for 18 weeks (2 weeks prior to DMBA and 16 weeks after DMBA)	No treatment	-	-	Inhibition of COX-2, HSP90, and NF-kB	-	Mandal et al. (2014)
Rb1	CK	i.p; 0.2 or 1 mg/kg every other day for 3 weeks	No treatment	Decreases	Decreases	-	No significant toxicity	Lee et al. (2018)
Rh2	Rh2-containing arginine-reduced graphene	i.v.; 3 mg/kg, every 3 days for 32 days	PBS, Rh2, Graphene-arginine	Decreases	Decreases	Reduces liver metastasis, decreases levels of TGF-β, IL-10, and Foxp3 and increases level of IFNγ	Mild liver toxicity	Farhangfar et al. (2022)

The most studied triterpenes in free form were UA (n = 5); BA (n = 4); CuB (n = 4); 20(S)-PPD (n = 2); actein (n = 2); AECHL-1 (n = 2); AS-IV (n = 2); Cel (n = 2); ginsenoside Rk1 (n = 2); OA (n = 2); pristimerin (n = 2) and SsA (n = 2) (Supplementary Material). Triterpenoids derived from OA (n = 10) and Cel (n = 2) as well as co-treatment with ginsenoside Rg3 (n = 4); cucurbitacin B (n = 2); TSN (n = 3) and SsD (n = 2) with standard anticancer drugs such as DOX, PTX and tamoxifen were also studied in rodent models of breast cancer (Supplementary Material). Additionally, formulations of Cel (n = 13); OA (n = 7); UA: (n = 7); Rg3: (n = 6); BA: (n = 4); Rh2: (n = 4); AA: (n = 2) and GA: (n = 2) were also investigated (Supplementary Material).

They were administered by various routes, including i. p. (n = 58); i. v. (n = 48); p. o. (n = 31); i. g. (n = 9); s. c. (n = 2); intratumorally (n = 3) and peritumorally (n = 1). Six studies used the unclear term injected and five studies did not mention the route of administration used. The tested triterpenes were given in doses ranging from 1.5 μg/kg (AECHL-1) to 250 mg/kg (BA) (Table 2, Supplementary Material) and four studies did not mention the dose used. The dosing regimen varied from a single dose to daily doses over several days, with the duration of treatment lasting from 1 day to 70 days 11 studies did not mention the duration of treatment. Studies included either a negative control group (n = 63), a positive control group (n = 1), or both (n = 99) (Table 2, Supplementary Material).

### Primary outcome

The primary outcomes measured for this review were tumor weight trend, tumor volume trend and information regarding the mechanism of action. Tumor weight trend was reported to be decreased compared to controls (n = 65), decreased compared to negative control and increased compared to positive control (n = 9), decreased compared to negative control and similar to positive control (n = 5), decreased compared to controls only for the higher triterpene dose tested (n = 5) and 77 studies did not report the tumor weight (Table 2, Supplementary Material). Tumor volume was reported to be decreased compared to controls (n = 107), decreased compared to negative control and increased compared to positive control (n = 8), decreased compared to negative control and similar to positive control (n = 8), decreased compared to controls only for the higher triterpene dose tested (n = 2) and 38 studies did not report the tumor volume (Table 2, Supplementary Material). Numerical values extracted from the tumor weight and volume are available in the Supplementary Material.

Several mechanisms of action responsible for the anticancer effect have been identified, including the induction of apoptosis, the inhibition of angiogenesis, the inhibition of the Wnt/β-catenin signaling pathway, the activation of the IL-12/STAT4 pathway, and the activation of the JNK1/2 pathway. Modification of cancer biomarkers, such as decreased PCNA and Ki-67, increased Bax/Bcl-2 ratio, increased cleaved caspase-3, decreased VEGF and CD31, and decreased matrix metalloproteinases, especially MMP-2 and MMP-9, were also observed (Table 2, Supplementary Material). In 29 studies, reduced lung metastasis was reported, four studies reported reduced liver metastasis, 2 studies reported reduced brain metastasis and one reported reduced bone metastasis. A number of 33 studies did not provide any information regarding the mechanism of action responsible for the anticancer effect in rodents.

### Secondary outcome

The secondary outcomes of our review were to gather information regarding the safety of triterpenes, including modifications of laboratory results, organ toxicity, and side effects. Thirteen studies reported no observed toxicity, 36 studies reported no significant toxicity, and 13 mentioned no organ toxicity. In 43 studies, animal weight was monitored before and after treatment, which either remained unchanged or the change was insignificant. There were several exceptions to this, including 30 mg/kg OA, 2 mg/kg Cel, 81 ppm and 50 mg/kg CDDO-Me, an association of 250 mg/kg BA+ 10 mg/kg taxol, NPs containing 0.1 mg/kg Squ, 10 mg/kg ginsenoside Rh2, and 2.5 mg/kg IR780 + laser, which caused significant weight loss. In one study, administration of 3 mg/kg CuB led to the death of all animals after the first administration (Suebsakwong et al., 2019) and in association with gemcitabine, 1 mg/kg CuB provoked leukopenia (Aribi et al., 2013). In another study using 4 mg/kg Cel, significant mortality was observed (Raja et al., 2011). A total of 46 studies did not provide any information regarding the safety observed *in vivo* of the tested compounds and formulations (Tables 2, 3, Supplementary Material).

**TABLE 3:** Toxicity data reported in rodent models of breast cancer.

Toxicity reports	Triterpenes	Triterpenoids	Cotreatment	Triterpenes + carriers	Total reports
Mortality	2	1	0	0	3
Weight loss	3	1	3	3	10
Histopathological changes	1	1	0	2	4
Leukopenia	1	0	0	0	1
Other blood tests modifications	2	0	1	1	4
Edema at the site of injection	1	0	0	0	1

### Risk of bias

**FIGURE 3:**
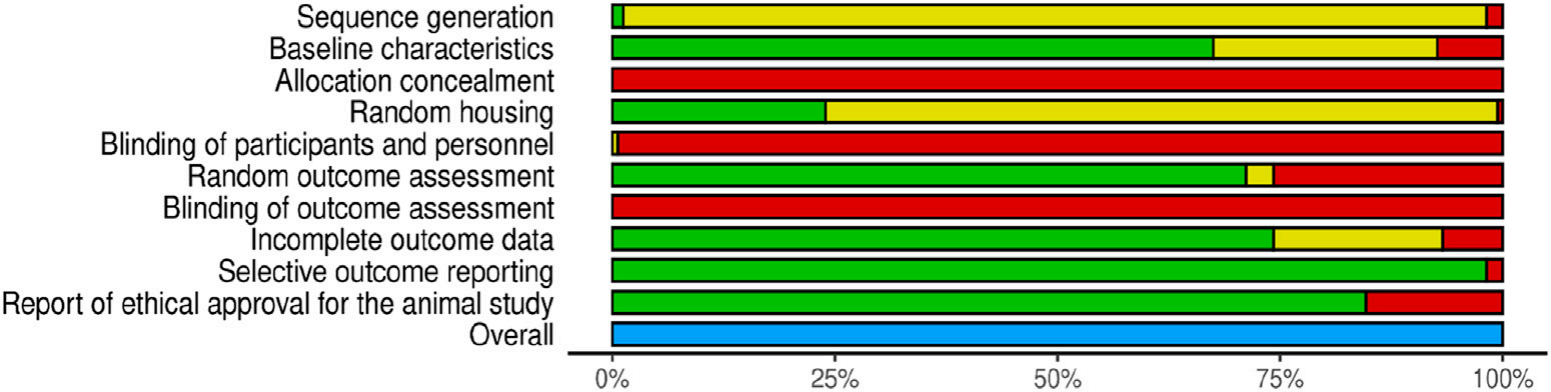
Risk of Bias assessment according to SYRCLE criteria (green-low risk of bias, yellow-unclear risk of bias, red-high risk of bias, blue-not assessed).

The results of the risk of bias assessment are summarized in Table 4 and Figure 3. The detailed analysis of individual studies is presented in the Supplementary Material. Our analysis revealed significant methodological limitations across all included studies; none of the included studies checked all the criteria assessed.

**TABLE 4:** Summary of risk of bias assessment (SYRCLE tool).

Type of bias		Low (%)	Unclear (%)	High (%)
Selection bias	Sequence generation	1.23	96.93	1.84
	Baseline characteristics	67.49	25.15	7.36
	Allocation concealment	0	0	100
Performance bias	Random housing	23.93	75.46	0.61
	Blinding of participants and personnel	0	0.61	99.39
Detection bias	Random outcome assessment	71.17	3.06	25.77
	Blinding of outcome assessment	0	0	100
Attrition bias	Incomplete outcome data	74.23	19.32	6.45
Reporting bias	Selective outcome reporting	98.16	0	1.84
Other	Report of ethical approval for the animal study	84.66	0	15.34

For this review, we considered sequence generation, allocation concealment, blinding of participants and personnel, and blinding of outcome assessment as key domains when assessing the quality of the studies against key ARRIVE guidelines (Percie du Sert et al., 2020). A major limitation was the high proportion of studies with unclear risk of bias for random sequence generation (96.93%), with only a very small proportion providing details on a proper randomization method. Moreover, none of the studies have provided proper allocation concealment and blinding of personnel and outcome assessors, indicating a high detection and performance bias.

We performed a semi-quantitative analysis of the results for tumor weight and volume using vote counting for direction of effect, which are summarized in Table 5.

**TABLE 5:** Vote-counting analysis of the effect of triterpenes on tumor weight and volume.

Direction of observed effect	Triterpenes	Triterpenoids	Cotreatment	Carriers	Total
Tumor weight					
Positive effect	39	8	12	27	86
No/negative effect	0	0	0	0	0
No data available	27	10	13	27	77
Tumor volume					
Positive effect	48	11	19	47	125
No/negative effect	0	0	0	0	0
No data available	18	7	6	7	38

Our sensitivity analysis of the 2 articles that reported proper sequence generation showed that triterpenes had a positive effect on tumor weight in both studies and a positive effect on tumor volume in one of the studies, while the other did not measure this parameter.

To explore potential sources of heterogeneity in our results, we performed a vote-counting analysis on several predefined subgroups. The results of our assessment are presented in Table 6. For the free triterpenes *versus* triterpenoids subgroup administered in monotherapy, triterpenes show a positive effect in a higher percentage of studies compared with triterpenoids, results consistent for both tumor weight (59.09% vs. 44.44%) and volume (72.73% vs. 33.33%). These results are also confirmed in the cotreatment subgroup, where free triterpenes perform better for both outcomes, showing a positive effect on tumor weight (50% vs. 33.33%) and a strong positive effect on tumor volume (81.82% vs. 33.33%).

**TABLE 6:** Vote-counting subgroup analysis of triterpene effects on tumor weight and volume.

Subgroup	Treatment type	Tumor weight trend compared with controls			Tumor volume trend compared with control			Total reports
Free triterpene/derivatives		Positive effects	No/Negative effects	No data availabe	Positive effects	No/Negative effects	No data available	
Free triterpene	Monotherapy	39	0	27	48	0	18	66
	Cotreatment	11	0	11	18	0	4	22
Triterpenoids (derivatives)	Monotherapy	8	0	10	11	0	7	18
	Cotreatment	1	0	2	1	0	2	3
With/without delivery systems								
With delivery systems	Monotherapy	10	0	10	16	0	4	20
	Cotreatment	17	0	17	31	0	3	34
Without delivery systems	Monotherapy	47	0	37	59	0	25	84
	Cotreatment	12	0	13	19	0	6	25
Tumor models/cell lines								
4T1 cells		28	0	17	40	0	5	45
MDA-MB-231 cells		16	0	16	29	0	3	32
MCF-7 cells		11	0	10	19	0	2	22
Multiple tumor types		7	0	8	8	0	7	15
DMBA -induced tumors		3	0	6	3	0	6	9
Transgenic mice		1	0	5	1	0	5	6
4T1-Luc cells		2	0	2	3	0	1	4
MDA-MB-231-Luc cells		0	0	3	1	0	2	3
EAT tumors		1	0	2	2	0	1	3
SKBR-3 cells		1	0	1	1	0	1	2
MMTV-Wnt1 tumors		2	0	0	2	0	0	2
MDA-MB-468 cells		0	0	2	2	0	0	2
MCF-7/adr cells		1	0	1	2	0	0	2
BT-474 cells		0	0	1	0	0	1	2
SUM159 cells		1	0	0	1	0	0	1
MDA-MB-361 cells		1	0	0	1	0	0	1
MDA-MB-231/GFP		1	0	0	1	0	0	1
MCF10DCIS cells		1	0	0	1	0	0	1
MCF-7/T cells		1	0	0	1	0	0	1
MCF-7/DOX cells		1	0	0	1	0	0	1
FM3A cells		0	0	1	0	0	1	1
EMT6M cells		1	0	0	1	0	0	1
BT20 cells		1	0	0	1	0	0	1
ALDH (high) BCSCs cells		1	0	0	1	0	0	1
4T1/rLu/GFP cells		0	0	1	0	0	1	1
4T1-luc-GFP cells		1	0	0	0	0	1	1
4T1-fluc-red cells		1	0	0	1	0	0	1
3T3/4T1 cells		1	0	0	0	0	1	1
Route of administration								
p.o		16	0	15	16	0	15	31
i.v		24	0	24	43	0	5	48
i.p		33	0	25	46	0	12	58
i.g		5	0	4	6	0	3	9
s.c		0	0	2	2	0	0	2
intratumorally		0	0	3	3	0	0	3
peritumorally		1	0	0	1	0	0	1
Injected (route not specified)		3	0	3	5	0	1	6

In monotherapy, the use of delivery systems showed similar positive effects on tumor weight (50.0% and 55.95%) compared to the free compounds. Furthermore, in terms of tumoral volume, it was shown that their inclusion in delivery systems led to better outcomes compared to their non-included counterparts (80.0% and 70.24%). As for the cotreatment group, the difference between the positive effects observed for triterpenes included in delivery systems was more pronounced compared to the free compounds in terms of tumor volume (91.18% and 76.0%), while displaying similar positive effects in tumor weight (50.0% and 48.0%).

The subgroup analysis on tumoral models showed the positive effects were more frequently observed on 4T1 models (62.22% in tumor weight and 88.89% in tumor volume) compared to MBA-MB-231 (50.0% and 90.63%) and MCF-7 (50.0% and 86.36%). However, on DMBA-induced tumor models, transgenic mice and 4T1-Luc cells, the proportion of positive effects was significantly lower on both endpoints (46.67% tumor weight and 53.33% tumor volume; 33.33% and 33.33%; 16.67% and 16.67%).

In terms of route of administration, it was shown that the most positive effects on tumor volume were observed when the compounds were administered i. v. (89.58%), i. p. (79.31%), i. g. (66.67%), while the p. o. administration showed lower results compared to the other routes (51.61%). In regards to the tumor weight, all the routes showed similar results, the highest ones registered for i. p. administration (56.9%) and i. g. administration (55.56%).

## Discussions

Our analysis revealed that triterpenes, triterpenoids, their formulations or cotreatments with other drugs reduce tumor weight, volume and metastasis of breast cancer in rodent models. These effects are achieved through diverse mechanisms, showing reduced toxicity as well.

The eligible articles were published between 2003–2024, with a notable increase observed from 2016 onward, supporting the increased interest in medicinal plants, often used in ethnomedicine, as a rich source of compounds with therapeutic potential in cancer prevention and management (Hashim et al., 2024). The majority of the articles were published in China and India, two Asian countries where traditional medicine is well-preserved (Liu et al., 2023) and remains the main treatment option for the majority of the population (Liu, 2021; Yao et al., 2023), due to tradition and affordability (Oyebode et al., 2016). Unsurprisingly, a 2020 literature survey (Yeung et al., 2020) indicates that for the last 2 decades, these countries have been the leading contributors to ethnopharmacology, the interdisciplinary exploration of traditional remedies, including plants, minerals, fungi, microbes and animals (Pirintsos et al., 2022; Schultz and Garbe, 2023).

The rodent models used in breast cancer studies have specific characteristics that make them suitable for certain research purposes (Welsh, 2013). Inbred mice species, which were used by the majority of studies included in the analysis, are widely accessible, affordable and have a high genetic similarity to humans (Boggess et al., 2004); they also possess traits that make them relevant for breast cancer studies, such as the susceptibility of Balb/c mice to MMTV-induced mammary tumors (Cheon and Orsulic, 2011). The immunodeficient mice were the second most prevalent choice identified and, despite allowing the formation and study of human breast cancer xenografts, the tumor growth and drug response in mice might not entirely replicate the human response (Shultz et al., 2014). Genetically modified mice, which are considered a better option for target validation, tumor response assessment, and identification of pharmacodynamic drug markers (Sharpless and DePinho, 2006), were also used. However, even with specific genetic alterations made to mimic human disease, inherent differences between species are inevitable and have to be considered (Borowsky, 2011). A few studies have also used rats, which show better similarities to human breast cancer compared to mouse models (Miller et al., 2022), in particular for the hormone receptor-positive type, which accounts for 70% of the invasive cancer forms in humans (Nicotra et al., 2024). Two species of mice were used in six studies, enhancing our understanding of the mechanisms and effects of triterpenes in various cancer models. However, in the future, these extensive studies should be made on highly active compounds to minimize unnecessary animal use in experiments, aligning with international regulations such as the European Union’s “Three Rs” principles that promote replacement, reduction, and refinement in animal research (European Commission, 2025).

Female animals were predominantly used in the studies, reflecting the human incidence as women’s breast cancer cases make up 99% of the total (Kreiter et al., 2014), with only 1% affecting men (Gómez-Raposo et al., 2010). However, about 10% of the studies did not report the sex of the animals used. This could be a serious limitation as sex differences are considered an important factor in the pathogenesis of cancer and in the drug response to chemotherapeutics (Klein and Flanagan, 2016). The studies that reported the animals’ age varied between 3 and 10 weeks; however, a significant proportion of the studies failed to report the age of the animals or used unclear terms such as “adult”. The reasons for choosing the age of the rodents in studies seem to be linked more to practicality and cost, rather than rodent particularities in different development stages (Jackson et al., 2017). For instance, liver metabolism differs in young *versus* old rodents (Mori et al., 2007; Pibiri et al., 2015) and can seriously impact xenobiotic metabolism. Future studies need to report more accurately the information regarding the animals used for their experiments and take into consideration age-related particularities when designing an experiment.

In the evaluated articles, breast cancer has been mainly modeled using allografts and xenografts obtained through various methods of administration, such as subcutaneously, intratumorally, and intravenously (Supplementary Material). Allografts are created by inoculating cells from the same species, typically mice, into another immunocompetent host (Yang et al., 2017) - although they are easily obtained their similarity to human breast cancer is limited. Xenografts are formed by injecting human cancerous cells into immunodeficient mice, offering a better but not perfect resemblance to human conditions for drug testing (Liu C et al., 2021; Singhal et al., 2023). Some studies utilized genetically modified mice, better suited for studying targeted therapies, modeling drug resistance and target validation (Holen et al., 2017). DMBA (7,12-dimethylbenzantracene), which induces breast cancer by generating free radicals and DNA damage (Khazaei et al., 2018) was also used scarcely as this model has inherent limitations such as difficulty to predict the location and time necessary for tumor development (Boix-Montesinos et al., 2021).

A significant proportion of the studies have investigated the effects of free triterpenes, naturally occurring compounds with anticancer potential in breast cancer (Şoica et al., 2021). However, due to their reduced bioavailability, they have a limited therapeutic effect (Atriya et al., 2023) Several strategies have been employed to overcome this limitation, as reflected in our search, where 60% of the articles utilized a wide range of approaches, including chemical derivatization, association with other drugs, and formulations for an enhanced anticancer effect.

Pentacyclic triterpenes and dammarane triterpenes (ginsenosides) were well-represented in the analyzed studies either in free form, derivatives, cotreatment or formulations due to their proven *in vitro* potential in breast cancer (Atriya et al., 2023; Deng et al., 2023).

Triterpenes were predominantly administered by i. p., i. v. and p. o. routes that are considered suitable for repeated administrations in rodents despite their disadvantages such as high first-pass metabolism (i.p. and p. o.), technical challenges (i.v.), need for sterility (i.p. and i. v.) and risk of injury if they are not properly used. In particular, i. p. administration is considered appropriate for proof-of-concept studies where the primary objective is to assess a candidate’s direct biological effect over pharmacokinetic investigations (Al Shoyaib et al., 2020), while p. o. and i. v routes are considered more suitable for clinical transition.

The triterpenes and triterpenoids were administered in doses ranging from 1.5 μg/kg (AECHL-1) to 250 mg/kg (BA). This wide range results from the structural diversity of this class, with more than 30,000 compounds isolated so far (Banerjee et al., 2019) and numerous semisynthetic derivatives (Nistor et al., 2022) that lead to different *in vivo* potencies. In preliminary studies, it is common for various compound doses to be tested to identify the maximum tolerated dose, an important parameter for toxicity assessment (Amorim et al., 2024). Two studies testing 2 and 4 mg/kg Cel (Raja et al., 2011) and 27 and 81 ppm CDDO-Me have found increased toxicity at the higher doses, providing valuable information regarding the safety profiles of these compounds. Several studies have incorporated the tested compounds into animal feed, while others have administered the compounds through gavage (forced feeding) (Table 2, Supplementary Material). While incorporating the compounds into the diet better simulates human intake and is more appropriate for chemoprevention studies, it also leads to unpredictable intake (Kapetanovic et al., 2011). On the other hand, gavage offers better drug intake accuracy but is considered more stressful for the animals and can lead to gavage-related reflux (Damsch et al., 2011) and esophageal injuries (Al Shoyaib et al., 2020).

The diversity of treatment duration across studies offers a broad perspective on the effect and toxicity of triterpenes, as single dose studies are important for preliminary evaluation of a new candidate (Organisation for Economic Co-operation and Development, 2002). Longer studies, which are better suited for the potential of clinical translation (Organisation for Economic Co-operation and Development, 2025), offer a more in-depth understanding on the effects of triterpenes. The treatment duration is limited by other factors such as costs involved and the need to reduce the animal pain in the late-phase of tumor development (Knight, 2011).

The studies that provided data on tumor weight and volume indicated that triterpenes, in all tested forms, reduced both weight and volume compared to negative controls. These values were either lowered or comparable to those in positive controls, with only a few studies reporting increased values compared to positive controls. Notably, one study (Song et al., 2020) employed only the positive control (β-actin) necessary to normalize the expression of certain proteins related to Rg3’s mechanism of action in breast cancer. This approach is justified here, as the anticancer effect of Rg3 in breast cancer has already been shown in previous studies (Liu Z et al., 2021), and the study focused on elucidating its mechanisms rather than validating its effectiveness. Although these results might not identically translate in human studies due to species particularities (Zheng et al., 2021), these consistent findings across diverse rodent models reinforce the efficacy of triterpenes in breast cancer. However, many articles reported neither one nor both of these outcomes. This might be due to the use of alternative outcome measurements such as tumor growth inhibition or tumor burden score, that were not collected for this review and are a limitation of our review design. Another limitation to be considered is the subjective interpretation of tumor weight and volume trends from the graphics, as the data was provided graphically rather than numerically. We have employed independent double screening and resolved the disagreements through discussions with a third reviewer to diminish this bias.

**FIGURE 4:**
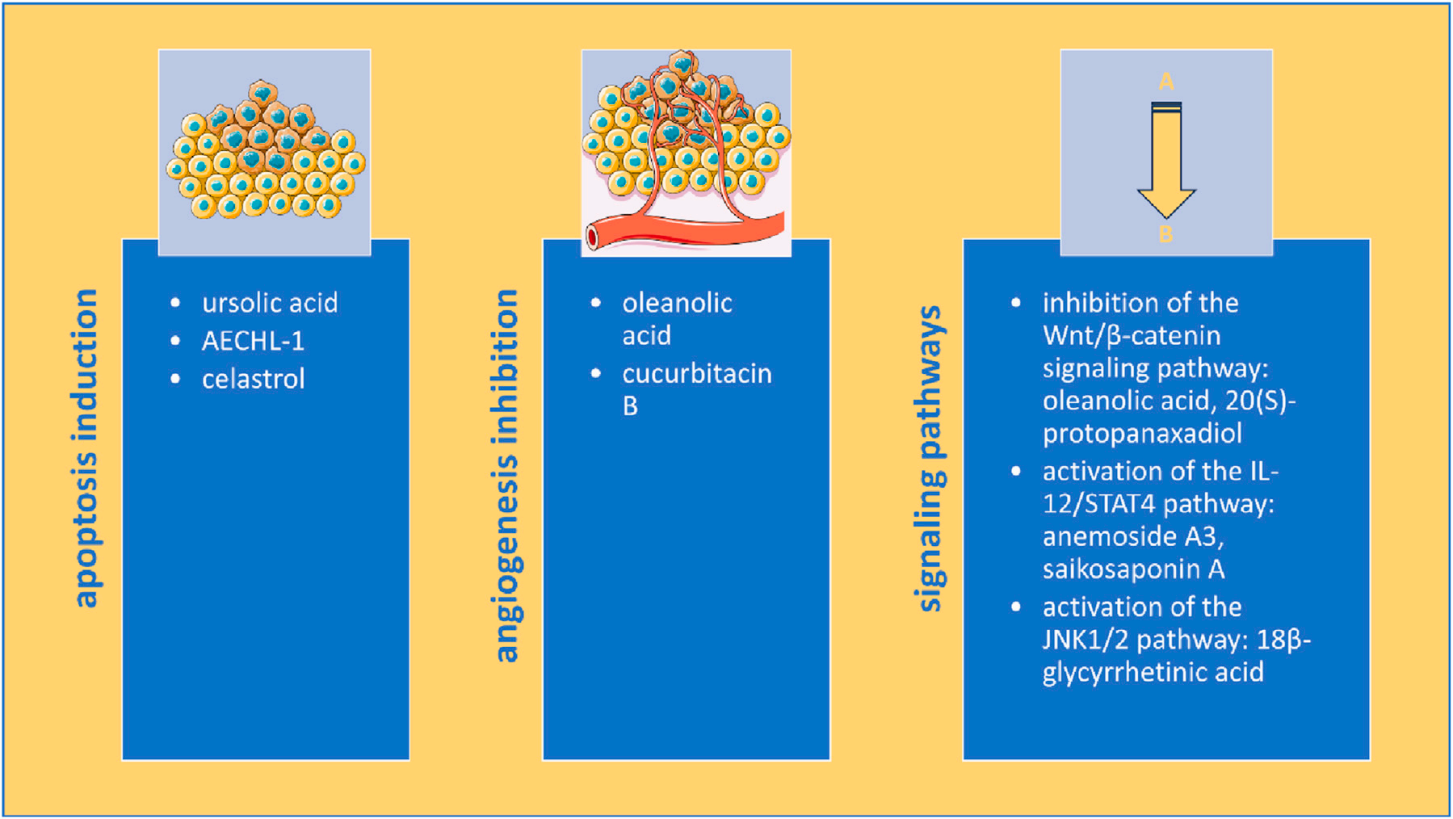
Multimodal anticancer mechanisms of triterpenes in rodent models of breast cancer (image adapted from Servier Medical Art (https://smart.servier.com/), licensed under CC BY 4.0 (https://creativecommons.org/licenses/by/4.0/).

Although elucidating the mechanism of action is not essential for clinical translation or use, after the thalidomide incident, more resources were invested in ensuring drug safety, including mechanism investigation before drugs are approved by regulatory agencies (Greene and Podolsky, 2012). The most common mechanisms identified in our analysis were apoptosis induction and angiogenesis inhibition (Figure 4).

Apoptosis is a programmed cell death that can be induced through intrinsic and extrinsic pathways that lead to caspase activation and characteristic cell alterations such as DNA fragmentation and blebbing of the cell membrane (Singh and Lim, 2022). Inhibition of apoptosis is partly responsible for the anticancer effect of standard drugs used for breast cancer treatment, such as DOX (Nicoletto and Ofner, 2022), PTX (Zhao et al., 2022) and tamoxifen (Ahmed et al., 2021). Several studies in our analysis have also observed that triterpenes increased Bax/Bcl-2 ratio and cleaved caspase-3 levels, two apoptosis markers (Saddam et al., 2024), effects confirmed in human breast cancer cell lines such as MCF-7 and MDA-MB231 as well (Shahrestanaki et al., 2023).

Angiogenesis, the process of new blood vessel formation from pre-existing ones, has an important role in the development and dissemination of solid tumors, including breast cancer (Ayoub et al., 2022). The efficacy of angiogenesis inhibitors in breast cancer is limited due to tumor resistance by activation of pre-angiogenic factors (Ruan et al., 2022) and bevacizumab, a humanized anti-VEGF monoclonal antibody, previously approved through FDA’s Accelerated Approval Program for the treatment of metastatic breast cancer had this indication revoked in 2011 due to safety and efficacy concerns (Sasich and Sukkari, 2012). Our analysis showed that triterpenes decreased VEGF and CD31, two vital angiogenesis markers that have been measured in breast cancer patients where increased VEGF serum and tissue levels was associated with distant metastasis and poor outcome (Ali et al., 2011) and increased CD31 tissue levels were associated with high invasiveness (Sapino et al., 2001).

Angiogenesis is also involved in the metastatic process of breast cancer, promoting tumor dissemination to other organs (Ayoub et al., 2022; Harry and Ormiston, 2021). The collected data indicates that many triterpenes can reduce lung metastasis in rodents, with a limited number of studies assessing the other organs commonly affected by breast cancer metastasis, such as the bones, liver, and brain (Ibragimova et al., 2023). Wnt/β-catenin signaling plays an important role in cell proliferation, metastasis, immune microenvironment regulation, and therapeutic resistance in breast cancer (Xu et al., 2020). Currently, none of the Wnt/β-catenin signaling inhibitors studied have passed phase II trials, despite this being the most altered pathway in breast cancer and an important chemotherapeutic target (Mukherjee and Panda, 2020). Cell proliferation can also be assessed through specific markers such as PCNA, Ki-67, and minichromosome maintenance proteins (MCM), which can have prognostic value for breast cancer (Juríková et al., 2016). Triterpenes have inhibited the Wnt/β-catenin signaling pathway and decreased PCNA and Ki-67 levels, showing antiproliferative potential in rodents. Triterpenes have also decreased matrix metalloproteinases (MMP) in rodents, especially MMP-2 and -9, which seem to play a crucial role in breast cancer tumorigenesis, though their role is not completely understood. While both have been established in breast cancer patients, increased MMP-9 levels are associated with a poor prognosis, while increased MMP-2 levels are linked to prolonged survival time (Cheng et al., 2022). Currently, no MMP inhibitors have received FDA approval for breast cancer treatment, and many broad-spectrum MMP inhibitors have been halted in early-stage clinical trials due to a lack of specificity and toxicity (Kwon, 2023).

Other signaling pathways emerging as potential chemotherapeutic targets for breast cancer (Núñez-Marrero, 2019; Wood et al., 2018), including the IL-12/STAT4 pathway and the JNK1/2 pathway, were also altered by triterpenes, showcasing their versatile abilities to suppress breast cancer.

As some of these mechanisms have been identified in both *in vitro* studies of human breast cancer cells and in rodents, which are more complex models, this might support the clinical relevance of triterpenes for human studies. A comparison of standard therapeutic classes used in breast cancer and triterpenes’ mechanisms is presented in Table 7. Although triterpenes do not exert their effect in breast cancer exactly as standard drugs, their multi-target approach could lower cancer resistance and toxicity. Nonetheless, precautions must be taken concerning emerging mechanisms involved in breast cancer that, despite having tremendous potential as drug targets, also pose significant risks until more information is collected.

**TABLE 7:** Mechanistic comparison: triterpenes vs. standard therapeutic classes.

Therapeutic approach	Drugs	Primary mechanism of action	References	Triterpenes mechanism (comparison)
Chemoterapy	Alkylating agents (cisplatin)	Inducing cell death by apoptosis	Oliveira et al. (2024)	Activation of apoptosis through several mechanisms, increased cleaved caspase-3 Bax/Bcl-2 ratio
	Antimitotic agents (PTX, docetaxel, ixabepilone, eribulin)	Stabilizing microtubules and preventing cell division		Inhibit cell division through cell cycle arrest G0/G1
	Antimetabolites (5-fluorouracil, capecitabine)	Inhibit thymidylate synthase		Do not target thymidylate synthase, but induce cell death through multiple mechanisms
	Anthracyclines (DOX, epirubicin)	Inhibit topoisomerase II and increase the production of ROS		Increase ROS production through other pathways
Hormone therapy	Selective estrogen receptor modulators (tamoxifen)	Acts as an antagonist of estrogen receptors in breast tissue and an agonist in bone tissue	Marina et al. (2023)	Do not directly influence the estrogen pathway
	Aromatase inhibitors (letrozole)	Inhibit the aromatase and block the synthesis of estrogen in peripheral tissues	Bhatnagar (2007)	
HER-2 targeted therapy	Monoclonal antibodies (trastuzumab)	Binds to the HER-2 receptor and inhibits downstream signaling, inducing cytotoxicity and phagocytosis	Mercogliano et al. (2023)	Can decrease the expression of HER-2 receptors or inhibit HER-2 phosphorylation
CDK4/6 inhibitors	Palbociclib, ribociclib, abemaciclib	Inhibit CDK4/6-RB pathway in breast cancer cells and induce cell cycle arrest in G1	Pernas et al. (2018)	Induce cell cycle arrest in G0/G1
Angiogenesis inhibitors	Bevacizumab (FDA authorization revoked for breast cancer)	Inhibit VEGF-A and tumor development	Li et al. (2022)	Inhibition of angiogenesis markers: VEGF and CD31 expression

The data presented in the majority of the studies suggest that triterpenes have a promising safety profile with no observed or significant toxicity in rodents. However, several studies have observed significant weight loss and in rare cases, leukopenia, hepatotoxicity and mortality at certain doses. Specifically, CuB in association with gemcitabine led to leukopenia at doses of 1 mg/kg (Aribi et al., 2013) and mortality at 3 mg/kg dose (Suebsakwong et al., 2019), effects that are absent when a smaller dose or a CuB prodrug is used. Triterpenes also reduced the toxicity of standard drugs such as DOX when used in combination, suggesting the benefit of their use in associations. However, a significant gap exists due to lack of toxicity assessment in several studies analyzed, impediments an objective toxicity assessment of these compounds, which further studies must address.

The development of nanotechnology has had a major impact on the creation of pharmaceutical formulations with improved bioavailability, safety, and patient compliance, allowing for the delivery of highly lipophilic or chemically unstable drugs (Milan et al., 2022). The results discussed in one review support the strong antitumor potential of delivery systems incorporating pentacyclic triterpenoids. Among these, self-assembling nanocarriers with inherent anticancer activity have emerged as a particularly promising direction for future research. Such systems not only reduce the risk of adverse drug events but also enhance drug accumulation at the tumor site (Kaps et al., 2021). For instance, nanoformulations containing BA, such as NPs, liposomes, polymeric micelles, nanotubes, nanogels, nanofibers, and nanosuspensions have demonstrated excellent therapeutic effects across a wide range of cancers, including breast cancer. In particular, they have substantially improved the solubility and bioavailability of BA, while also enhancing its anticancer efficacy through strategies such as controlled release and targeted delivery (Wang et al., 2024). Furthermore, the data analyzed in our review supports that the inclusion of free triterpenic derivatives in different types of nanocarriers might reduce toxic effects, leading to reduced mortality rates, no significant weight loss, no modifications in the rodents’ blood tests and no observed histopathological changes.

In the risk of bias analysis, we did not identify any study that checked all the criteria assessed, showing that the methodological rigor in animal studies can be improved in the future. As no study reported an allocation concealment or blinding of the research personnel regarding the treatment and control groups, the results reported and our analysis might be negatively impacted and should be taken into account. Moreover, our analysis revealed that a significant number of studies had unclear risk of bias for both sequence generation (96.93%) and random housing (75.46%), posing significant concerns that in the absence of proper animal randomization, the effects of triterpenes in breast cancer models might be overestimated. Other limitations of the review to be considered are the absence of a quality assessment due to the extensive number of included studies, publication bias (exclusion of abstracts, conferences, or proceedings), language bias (English-language only), database bias (only searching three databases), positive results bias (positive results are more likely to be published) and articles that could not be retrieved and were excluded from the analysis as they might contain relevant information on the subject.

Our primary vote-counting analysis showed that triterpenes reduce tumor weight (52.76%) and volume (76.68%) in a significant number of studies. However, as a considerable number of studies, (23.32% for tumor volume and 47.24% for tumor weight), failed to measure and report these metrics, these encouraging findings should be interpreted with caution, taking into account the methodological shortcomings identified in the risk of bias assessment. Our primary results are also supported by a sensitivity analysis of articles with more rigorous methodology. The analysis showed that triterpenes have a positive effect on both tumor weight (100%) and volume (50%), indicating that the overall findings are not only a result of studies with methodological weaknesses. Nevertheless, the reduced sample size used for this analysis (n = 2) limits our ability to draw a definitive conclusion. Further research with more meticulously designed protocols, aligned with renowned guidelines such ARRIVE guidelines (Percie du Sert et al., 2020), are needed to confirm these promising results.

We performed a vote-counting analysis on predefined subgroups to explore potential sources of heterogeneity in our assessment. Our subgroup analysis revealed that free triterpenes performed better than triterpenoids on tumor weight and volume, results consistent in both monotherapy and cotreatment settings. These results come in contrast with the results of many *in vitro* studies, which often report superior anticancer effects for triterpenoids (Lombrea et al., 2023; Ye et al., 2017). This suggests that in rodent models, the anticancer effect is highly dependent on the chemical structure of native triterpenes, a factor that may be related to differences in the mechanism of action and bioavailability. Our analysis also showed that in the monotherapy group, the use of carriers has a potent effect, particularly in reducing tumor volume, while the effect on tumor weight was similar to that of free compounds. The same consistent results were observed in the cotreatment group, but in this group, the differences were more pronounced, suggesting the use of carriers in combinational therapy might exacerbate the antitumor effect of triterpenes.

Taking into account the utilized tumor models, the analysis revealed that the positive effects were mostly observed in 4T1, MBA-MB-231 and MCF-7 tumor models. A solid explanation for this predilection might be supported by the large variety of preclinical studies involving these types of cell lines (Atiya et al., 2019; Lee et al., 2015; Sahu et al., 2024; Tao et al., 2008). The highly observed percentages for the positive effects (50%–62% for tumor weight and 85%–90% for tumor volume) might suggest a robust efficacy tendency in these established models. It has been well established that these cell lines represent standard models for triple-negative mammary cancer (4T1 and MBA-MB-231) and luminal (MCF-7) cancer (Garbar et al., 2017), hence offering translational relevance. However, in chemical-induced (DMBA) or transgenic models, the results showed less effective results (33% and 16%). These results could be explained by the fact that these rodent models are limited by their low heterogeneity compared to the highly heterogeneous human tumors (Lampreht Tratar et al., 2018) hence, the anticancer effects of triterpenes and triterpenoids could be stronger in a highly heterogeneous tumor microenvironment (Tonello et al., 2025). These results strongly suggest that more complex models that mimic the human tumoral environment are required in future studies.

Comparing the results in the route of administration subgroup, we found that all routes showed similar results for tumor weight, showing that the long-term effect of triterpenes is not dependent on the route of administration. In contrast, the route of administration had a significant effect on the tumor volume, a marker for early tumor shrinkage in breast cancer (Che et al., 2020). Interestingly, the routes that avoid the first-pass metabolism, i. v. and i. p. had a strong positive effect in reducing tumor volume, while the i. g. administration, which also avoids first-pass metabolism, had shown a lower efficacy, suggesting that the influence of the gastric environment might reduce triterpenes’ bioavailability and efficacy. These findings are also supported by the moderate positive effect on tumor volume of triterpenes administered orally.

## Future directions

Current evidence of triterpenes in rodent models of breast cancer is promising but to achieve clinical translation, future investigations are needed. Several areas of improvement are the following:

- optimization of preclinical models: stringent methodologies (baseline characteristics, allocation concealment, blinding of personnel, ethical approval), methodological transparency, use of patient-xenograft models for better translatability;
- mechanistic understanding: study of molecular pathways involved in the anticancer effect of triterpenes in different subtypes of breast cancer;
- optimization of nanoformulations and its use in combinational therapy: to overcome bioavailability and toxicological challenges;
- complex pharmacokinetic and pharmacodynamic studies: complex studies to help identify the optimal formulations and doses in animal models;
- rigorous toxicological studies: study of the molecular mechanisms involved in the toxicity of triterpenes (e.g., hepatotoxicity, leukopenia) and identification of strategies to reduce these effects.

## Conclusion

Our analysis showed that triterpenes were tested in free form, chemical derivatives, delivery systems (liposomes, micelles, NPs) and association with other drugs in different rodent models of breast cancer, yielding promising results in terms of tumor weight and volume reduction. Our primary vote-counting analysis results have shown that triterpenes have positive effects on key outcomes, such as tumor weight and volume, findings confirmed by a sensitivity analysis. The subgroup analysis highlighted key findings: free triterpenes were more effective than triterpenoids in both monotherapy and combination treatments; delivery systems significantly improved early tumor volume reduction, especially in cotreatment scenarios; greater positive effects were seen in well-established tumor models (4T1, MDA-MB-231, and MCF-7), while chemically-induced or transgenic models were less effective; all administration routes demonstrated similar long-term effects on tumor weight, but methods bypassing first-pass metabolism (i.v. and i. p.) led to greater early tumor volume reduction.

Triterpenes exerted their effects primarily through the induction of apoptosis, along with angiogenesis and metastasis inhibition; however, other mechanisms, including the inhibition of the Wnt/β-catenin signaling pathway, the activation of the IL-12/STAT4 pathway, and the activation of the JNK1/2 pathway, were also observed. Determining the structure-activity relationship could accelerate the understanding of the triterpenes’ mechanism of action.

Reduced toxicity has been observed in the majority of the studies, with only a few reporting significant weight loss that might indicate some degree of toxicity. Notable side effects, such as hepatotoxicity, mortality and leukopenia were also observed in a limited number of studies for certain triterpenes used in higher doses; toxicity that could be reduced by the use of nanocarriers. Major limitations that hamper our assessment are the lack of data on the mechanisms of action or safety in a substantial number of studies and the methodological shortcomings identified in the risk of bias assessment (lack of randomization and blinding), which could have contributed to the observed positive effects of triterpenes.

Future research must employ more rigorously designed studies aligned with international guidelines to ensure transparency and reproducibility that are needed to validate these results. We conclude that while the evidence for the effects of triterpenes in rodent models of breast cancer is positive, it currently does not support clinical translation until further understanding is obtained regarding the mechanisms of action, the structure-activity relationship, and safety.

## Data Availability

The original contributions presented in the study are included in the article/Supplementary Material, further inquiries can be directed to the corresponding author.

## Glossary

20(S)-PPD: protopanaxadiol
25-OCH3-PPD: ginsenoside 20(S)-25-methoxyl-dammarane-3b, 12b, 20-triol
AA: asiatic acid
AMR-Me: methyl-25-hydroxy-3-oxoolean-12-en-28-oate
AS-IV: astragaloside IV
BA: betulinic acid
Bet: betulin
Bet-CA: ester derivative of BA and dichloroacetate
BoA: betulonic acid
BXL0124: 1α,25-dihydroxy vitamin D3
CA: corosolic acid
CDDO: 2-cyano-3,12-dioxooleana-1,9-dien-28-oic acid
CDDO-Im: 1-(2-cyano-3,12-dioxooleana-1,9-dien-28-oyl) imidazole
CDDO-Me: 2-cyano-3,12-dioxooleana-1,9(11)-dien-28-oic acid
Ce6: Clorine 6
Cel: celastrol
CK: ginsenoside CK, 20-O-beta-D-glucopyranosyl-20(S)-proto-panaxa-diol, compound K
CMC-Na: sodium carboxymethyl cellulose
CuB: cucurbitacin B
DABO-Me: methyl 3β-O-[4-(2-aminoethylamino)-4-oxo-butyryl]olean-12-ene-28-oate
DMBA: 7,12-dimethylbenz(a)anthracene
DMSO: dimethyl sulfoxide
DOX: doxorubicin, adryamicin
DPBS: Dulbecco’s Phosphate-Buffered Saline
GA: 18β-glycyrrhetinic acid
HBSS: Hanks’ Balanced Salt Solution
HCPT: hydroxycamptothecin
Ir: irinotecan
JSI-124: cucurbitacin I
ITSN: isotoosendanin
LMWH: Low molecular weight heparin
MMP: matrix metalloproteinases
NBT: lup-20(29)-en-3β,28-di-yl-nitrooxy acetate
NIR: near-infrared radiation
NPs: nanoparticles
OA: oleanolic acid
PBS: phosphate-buffered saline
PCNA: proliferating cell nuclear antigen
PD-L1: programmed cell death 1 ligand 1 antibody
PEG: polyethylene glycol
PICO: Population-Intervention-Comparator-Outcomes
PRISMA: Preferred Reporting Items for Systematic reviews and Meta-Analyses
PROSPERO: International Prospective Register of Systematic Review
PSD: pulsatilla saponin D
PSN: P-selectin targeting peptide
PTX: paclitaxel
QDT: oleanolic quaternary ammonium derivative
Rb1: ginsenoside Rb1
Rb3: ginsenoside Rb3
Rd: ginsenoside Rd
Rg1: ginsenoside Rg1
Rg2: ginsenoside Rg2
Rg3: ginsenoside Rg3
Rg5: ginsenoside Rg5
Rh1: ginsenoside Rh1
Rh2: ginsenoside Rh2
Rh4: ginsenoside Rh4
Rk1: ginsenoside Rk1
SsA: saikosaponin A
SsD: saikosaponin D
Squ: squamocin
TRAIL: tumor necrosis factor-related apoptosis-inducing ligand
TSN: toosendanin
UA: ursolic acid
WOS: Web of science
