# Supplementary material for: Triterpenes in breast cancer: a systematic review of preclinical evidence in rodents
Source: Front Pharmacol. 2025 Nov 14;16:1623772. doi: 10.3389/fphar.2025.1623772 (PMC12660234; doi:10.3389/fphar.2025.1623772)
Supplement: Supplementary file 1 [file DataSheet1.pdf]

Supplementary material

Table S1. Search strategy

**Search terms**	triterpene, triterpenoid, betulinic acid, ursolic acid, lupeol, ginsenoside, glycyrrhetinic acid, celastrol, cucurbitacin, oleanolic acid, saikosaponin, lanosterol, maslinic acid, breast cancer, mammary carcinoma, breast tumor, mammary gland cancer, breast adenocarcinoma, mammary neoplasm, rodent, mice, mouse, rat, hamster, guinea pig, in vivo, preclinical research, xenograft, orthotopic model, murine model, metastasis model, animal model
**Search sequences**	**PubMed/Medline and WOS**:<br>(triterpene OR triterpenoid OR betulinic acid OR ursolic acid OR lupeol OR ginsenoside OR glycyrrhetinic acid OR celastrol OR cucurbitacin OR oleanolic acid OR saikosaponin OR lanosterol OR maslinic acid) AND (breast cancer OR mammary carcinoma OR breast tumor OR mammary gland cancer OR breast adenocarcinoma OR mammary neoplasm) AND (rodent OR mice OR mouse OR guinea pig OR in vivo OR preclinical research OR rat OR hamster OR xenograft OR orthotopic model OR murine model OR metastasis model OR animal model)<br>Filters: Search field (title/abstract); Article language (English)

	**WOS**:<br>(triterpene OR triterpenoid OR betulinic acid OR ursolic acid OR lupeol OR ginsenoside OR glycyrrhetinic acid OR celastrol OR cucurbitacin OR oleanolic acid OR saikosaponin OR lanosterol OR maslinic acid) AND (breast cancer OR mammary carcinoma OR breast tumor OR mammary gland cancer OR breast adenocarcinoma OR mammary neoplasm) AND (rodent OR mice OR mouse OR guinea pig OR in vivo OR preclinical research OR rat OR hamster OR xenograft OR orthotopic model OR murine model OR metastasis model OR animal model)<br>Filters: Search field (topic); Language (English)<br><br>**Scopus:**<br>(triterpene OR triterpenoid OR "betulinic acid" OR "ursolic acid" OR lupeol OR ginsenoside OR "glycyrrhetinic acid" OR celastrol OR cucurbitacin OR "oleanolic acid" OR saikosaponin OR lanosterol OR "maslinic acid") AND ("breast cancer" OR "mammary carcinoma" OR "breast tumor" OR "mammary gland cancer" OR "breast adenocarcinoma" OR "mammary neoplasm") AND (rodent OR mice OR mouse OR "guinea pig" OR "in vivo" OR "preclinical research" OR rat OR hamster OR xenograft OR "orthotopic model" OR murine model OR "metastasis model" OR "animal model")<br>Filters: Search within (article title, abstract and key words); Language (English)
**Databases searched**	PubMed/Medline<br>WOS

	Scopus
**Date of the search**	14 November 2024
**Other sources**	Reference lists of relevant reviews on the subject
**Part of journals searched**	title, abstract and keywords
**Years of search**	no restrictions set
**Language**	English language only
**Types of studies to be included**	peer-reviewed articles

Table S2. Triterpenes (D/A*- data available within the source article in graphical form, N/A- data not available)

Name of the first author and year of publication	Title of the article	Country	Study type	Animal model (sex, type, age)	Type of breast cancer inoculated and tumor inoculation method	Triterpene	Tumor weight (tested compound)	Tumor weight (control)	Tumor volume (tested compounds)	Tumor volume (control)	Reference
Yang 2019	16-Tigloyl linked barrigenol-like triterpenoid from Semen Aesculi and its anti-tumor activity <i>in vivo</i> and <i>in vitro</i>	China	Cross sectional	female Balb/c mice, 7-8 weeks	4T1 cells; s.c. injection into the right armpit	16a-Tigloyl-O-protoaescigenin	D/A*	D/A*	1 mg/kg: 380 mm^3	660 mm^3	(Yang et al., 2019)

Cheng 2023	Glycyrrhetinic acid suppresses breast cancer metastasis by inhibiting M2-like macrophage polarization via activating JNK1/2 signaling	China	Cross sectional	female Balb/c mice, 6–8 weeks	4T1 cells; s.c.; right axillary of each mouse	GA	D/A*	D/A*	D/A*	D/A*	(Cheng et al., 2023)
Peng 2019	Ginsenoside 20(S)-protopanaxadiol inhibits triple-negative breast cancer metastasis in vivo by targeting EGFR-mediated MAPK pathway.	China	Cross sectional	female Balb/c nu/nu mice, 5 weeks and Balb/c mice, 5–6 weeks	MDA-MB-231,MDA-MB-231-GFP cells or 4T1-luc cells; implanted; 4th mammary fat pad	20(S)-protopanaxadiol	D/A*	D/A*	D/A*	D/A*	(Peng et al., 2019)
Zhang 2018	20(S)-protopanaxadiol-induced apoptosis in MCF-7 breast cancer cell line through the inhibition of PI3K/AKT/mTOR signaling pathway	China	Cross sectional	female Balb/c mice, 6 weeks	MCF-7 cells; s.c.; right flanks	20(S)-protopanaxadiol	N/A	N/A	low dose group: 2065.1 ± 105.2 mm^3; high dose group: 1609.1 ± 96.2 mm^3	2514.9 ± 221.7 mm^3	(H. Zhang et al., 2018)

Li 2021	Ginsenoside (20S)-protopanaxatriol induces non-protective autophagy and apoptosis by inhibiting Akt/mTOR signaling pathway in triple-negative breast cancer cells	China	Cross sectional	female nude mice, 6-8 weeks	MDA-MB-231 cells; injected; right axilla	20(S)-protopanaxatriol	D/A*	D/A*	D/A*	D/A*	(Li et al., 2021)
Wu 2020	Actein Inhibits Tumor Growth and Metastasis in HER2-Positive Breast Tumor Bearing Mice via Suppressing AKT/mTOR and Ras/Raf/MAPK Signaling Pathways	China	Cross sectional	female SCID mice, 6–8 weeks old	MDA-MB-361 cells; s.c.; mammary fat pad	actein	D/A*	D/A*	D/A*	D/A*	(Wu et al., 2020)
Yue 2016	New potential beneficial effects of actein, a triterpene glycoside isolated from Cimicifuga species, in breast cancer treatment.	China	Cross sectional	female Balb/c mice	4T1 cells	actein	D/A*	D/A*	D/A*	D/A*	(Yue et al., 2016)
Dasgupta 2016	AECHL-1 targets breast cancer progression via inhibition of metastasis, prevention of EMT and suppression of Cancer Stem Cell characteristics.	India	Cross sectional	female SCID mice, 6 weeks	MCF-7 and MDA-MB-231 cells; s.c.; dorsolateral flank	AECHL-1	N/A	N/A	N/A	N/A	(Dasgupta et al., 2016)

Sawant 2016	Novel triterpenoid AECHL-1 induces apoptosis in breast cancer cells by perturbing the mitochondria-endoplasmic reticulum interactions and targeting diverse apoptotic pathways.	India	Cross sectional	female SCID mice, 6-8 weeks	MCF-7 cells; s.c.; right flank	AECHL-1	D/A*	D/A*	D/A*	D/A*	(Sawant et al., 2016)
Mu 2017	AG36 Inhibits Human Breast Cancer Cells Proliferation by Promotion of Apoptosis In vitro and In vivo.	China	Cross sectional	female Balb/c mice	MCF-7, s.c. into the right anterior armpit	AG36	D/A*	D/A*	D/A*	D/A*	(Mu et al., 2017)
Yin 2021	Anemoside A3 activates TLR4-dependent M1-phenotype macrophage polarization to represses breast tumor growth and angiogenesis	China	Cross sectional	female Balb/c mice, 6 weeks	4T1-Luc cells; orthotopically injected; right fourth mammary gland	anemoside A3	D/A*	D/A*	D/A*	D/A*	(Yin et al., 2021)
Hu 2020 (1)	ROCK1 activation-mediated mitochondrial translocation of Drp1 and cofilin are required for arnidiol-induced mitochondrial fission and apoptosis	China	Cross sectional	female nude mice, 5–6 weeks	MDA-MB-231 cells; s.c.; flanks mice	arnidiol	N/A	N/A	D/A*	D/A*	(Hu et al., 2020)

Gou 2020	Asiatic Acid Interferes with Invasion and Proliferation of Breast Cancer Cells by Inhibiting WAVE3 Activation through PI3K/AKT Signaling Pathway	China	Cross sectional	nude mice	MDA-MB-231 cells; s.c.; right armpit	AA	D/A*	D/A*	D/A*	D/A*	(Gou et al., 2020)
Hu 2020	Astragaloside IV inhibits cell proliferation and metastasis of breast cancer via promoting the long noncoding RNA TRHDE-AS1	China	Cross sectional	male Balb/c nu/nu mice, 5 weeks	MDA-MB-231- and TRHDE-AS1 silcenced MDA-MB-231 cells; s.c. implanted; flanks of the mice	AS-IV	N/A	N/A	D/A*	D/A*	(Hu et al., 2021)
Jiang 2017 (1)	Astragaloside IV inhibits breast cancer cell invasion by suppressing Vav3 mediated Rac1/MAPK signaling.	China	Cross sectional	female Balb/c nu/nu mice, 6 weeks	MDA-MB-231 cells; implanted; mammary fat pads	AS-IV	N/A	N/A	D/A*	D/A*	(Ke Jiang et al., 2017)
Zhang 2021 (1)	Betulin ameliorates 7,12-dimethylbenz(a)anthracene-induced rat mammary cancer by modulating MAPK and	China	Cross sectional	female rats, adult	DMBA; p.o.	Bet	N/A	N/A	N/A	N/A	(J. Zhang et al., 2021)

	AhR/Nrf-2 signaling pathway										
Damle 2013	Anticancer activity of betulinic acid on MCF-7 tumors in nude mice.	India	Cross sectional	female Balb/c mice	MCF-7 cells; s.c.; right flanks	BA	N/A	N/A	50 mg/kg: 0.64 ± 0.06; 100 mg/kg: 0.31 ± 0.07	no treatment: 1.33 ± 0.06; DMSO: 1.27 ± 0.09	(Damle et al., 2013)
Jiao 2019	Betulinic acid suppresses breast cancer aerobic glycolysis via caveolin-1/NF-κB/c-Myc pathway.	China	Cross sectional	female MMTV-PyVT+/− mice, 6 weeks	transgenic mice	BA	N/A	N/A	D/A*	D/A*	(Jiao et al., 2019)
Mertens-Talcott 2012	Betulinic acid decreases ER-negative breast cancer cell growth in vitro and in vivo: role of Sp transcription factors and microRNA-27a:ZBTB10.	USA	Cross sectional	female Balb/c nu/nu mice, 3–4 weeks	MDA-MB-231 cells; s.c.; flank mouse	BA	D/A*	D/A*	D/A*	D/A*	(Mertens-Talcott et al., 2013)

Arif 2024	Network Pharmacology and Experimental Validation to Explore the Potential Mechanism of Nigella sativa for the Treatment of Breast Cancer	USA	Cross section al	female Albino rats	DMBA ; p.o.	BA	N/A	N/A	N/A	N/A	(Arif et al., 2024)
Raja 2011	Anticancer activity of Celastrol in combination with ErbB2-targeted therapeutics for treatment of ErbB2-overexpressing breast cancers.	USA	Cross section al	female NOD-SCID mice, 4-6 weeks	BT-474 cells; injected	Cel	N/A	N/A	N/A	N/A	(Raja et al., 2011)
Li 2018	Celastrol induces ubiquitin-dependent degradation of mTOR in breast cancer cells.	China	Cross section al	female nude mice, 5 weeks	MDA-MB-231 cells; injected; into the 4th breast	Cel	N/A	N/A	D/A*	D/A*	(Li et al., 2018)
Wakimoto 2008	CuB has a potent antiproliferative effect on breast cancer cells in vitro and in vivo	USA	Cross section al	female nude mice	MDA-MB-231 cells orthotopical ly; breasts of female nude mice	CuB	0.87 g	1.29 g	N/A	N/A	(Wakimot o et al., 2008)

Sinha 2016	CuB inhibits breast cancer metastasis and angiogenesis through VEGF-mediated suppression of FAK/MMP-9 signaling axis.	India	Cross section al	female Balb/c mice	4T1 cells, s.c. into the mammary fat pad	CuB	D/A*	D/A*	D/A*	D/A*	(Sinha et al., 2016)
Gupta 2014	Inhibition of Integrin-HER2 signaling by CuB leads to in vitro and in vivo breast tumor growth suppression.	USA	Cross section al	female nude mice, 4-6 weeks and female Balb/c, 4-6 weeks	MDA-MB231 cells; injected orthotopical ly; mammary fat pads and 4T1 cells; injected; mammary fat pads	CuB	D/A*	D/A*	xenograft: 111,3 ± 18,4 mm³; allograft: 1446,7 ± 142,6 mm³	xenograft: 249,5 ± 30,3 mm³; allograft: 2355,3 ± 236,4 mm³	(Gupta and Srivastava , 2014)
Liang 2018	CuB inhibits the migration and invasion of breast cancer cells by altering the biomechanical properties of cells	China	Cross section al	female Balb/c mice, 6 weeks	SKBR-3 cells; injected; mammary fat pad	CuB	N/A	N/A	N/A	N/A	(Liang et al., 2019)

Zhang 2022 (3)	Epifriedelinol Ameliorates DMBA-Induced Breast Cancer in Albino Rats by Regulating the PI3K/AKT Pathway	China	Cross sectional	female Wistar rats (adult)	DMBA; p.o.	epifriedelinol	D/A*	D/A*	D/A*	D/A*	(Jing Zhang et al., 2022)
Liu 2018 (1)	Esculentoside A suppresses breast cancer stem cell growth through stemness attenuation and apoptosis induction by blocking IL-6/STAT3 signaling pathway.	China	Cross sectional	female Balb/c mice, 6 weeks	EMT6M cells; s.c.; inoculated	esculentoside A	10 mg/kg: 1.56 ± 046 g; 20 mg/kg: 1.36 ± 0.37 g; 30 mg/kg: 1.26 ± 0.45 g	2.18 ± 0.31 g	D/A*	D/A*	(Liu et al., 2018)
Al Marzouqi 2011	Frondoside A inhibits human breast cancer cell survival, migration, invasion and the growth of breast tumor xenografts.	United Arab Emirates	Cross sectional	female NMRI nude mice, 6-10 weeks	MDA-MB-231 cells; s.c.; lateral flank of the mice	frondoside A	D/A*	D/A*	D/A*	D/A*	(Al Marzouqi et al., 2011)
Liu 2018 (2)	Ginsenoside Rg5 induces apoptosis and autophagy via the inhibition of the PI3K/Akt pathway against breast cancer in a mouse model	China	Cross sectional	female Balb/c nu/nu mice, 4 weeks	MCF-7 cells; s.c.; left flank	ginsenoside Rg5	D/A*	D/A*	D/A*	D/A*	(Liu and Fan, 2018)

Yang 2024 (1)	Ginsenoside Rh2 enhances immune surveillance of natural killer (NK) cells via inhibition of ERp5 in breast cancer	China	Cross section al	female Balb/c nu/nu mice, 6 weeks	MDA-MB-231 cells; injected; mammary fat pads	ginsenoside Rh2	N/A	N/A	D/A*	D/A*	(C. Yang et al., 2024)
Wang 2012	Natural product ginsenoside 25-OCH3-PPD inhibits breast cancer growth and metastasis through down-regulating MDM2.	China	Cross section al	nude mice, 4–6 weeks	MCF-7 and MDA-MB-468 cells; i.v.; tail vein	ginsenoside 20(S)-25-methoxyl-dammarane-3b, 12b, 20-triol (25-OCH3-PPD)	N/A	N/A	N/A	N/A	(Wang et al., 2012)
Zhang 2022 (4)	Ginsenoside CK induces apoptosis in triple-negative breast cancer cells by targeting glutamine metabolism	China	Cross section al	female Balb/c nu/nu mice, 4–6 weeks	SUM159 cells; injected; left axilla of mice.	ginsenoside CK	D/A*	D/A*	D/A*	D/A*	(B. Zhang et al., 2022)
Zhang 2017	Ginsenoside Rd regulates the Akt/mTOR/p70S6K signaling cascade and suppresses angiogenesis and breast tumor growth.	China	Cross section al	female Balb/c mice, 5 weeks	MDA-MB-231 cells; s.c.; right flank of each mouse.	ginsenoside Rd	D/A*	D/A*	D/A*	D/A*	(Zhang et al., 2017)

Chu 2020	Ginsenoside Rg1 Induces Apoptotic Cell Death in Triple-Negative Breast Cancer Cell Lines and Prevents Carcinogen-Induced Breast Tumorigenesis in Sprague Dawley Rats	China	Cross sectional	female Sprague-Dawley rats, 6-7 weeks	DMBA; s.c.; near the mammary gland	ginsenoside Rg1	N/A	N/A	10.31 ± 1.98 mm³	20.85 ± 2.94 mm³	(Chu et al., 2020)
Jeon 2021	Ginsenoside-Rg2 affects cell growth via regulating ROS-mediated AMPK activation and cell cycle in MCF-7 cells.	Korea	Cross sectional	male Balb/c nu/nu mice, 6 weeks	MCF-7 cells; s.c.; right limb of mice	ginsenoside Rg2	D/A*	D/A*	67 ± 24 mm³	PBS: 117 ± 12 mm³<br>4-hydroxytamoxifen: 22 ± 6 mm³	(Jeon et al., 2021)
Ning 2024	Ginsenoside Rg3 decreases breast cancer stem-like phenotypes through impairing MYC mRNA stability	China	Cross sectional	female Balb/c nu/nu mice, 4-6 weeks	MDA-MB-231 cells and seccondary tumor xenografts; s.c. into the right flanks (primary tumor); s.c. into four different flanks of each mouse (xenograft)	ginsenoside Rg3	N/A	N/A	N/A	N/A	(Ning et al., 2024)

Song 2020	Inhibitory effect of ginsenoside Rg3 on cancer stemness and mesenchymal transition in breast cancer via regulation of myeloid-derived suppressor cells	Korea	Cross sectional	female C3H/He mice, 6 weeks	FM3A cells; s.c.; right flanks	ginsenoside Rg3	N/A	N/A	N/A	N/A	(Song et al., 2020)
Huynh 2021	Ginsenoside rh1 induces mcf-7 cell apoptosis and autophagic cell death through ros-mediated akt signaling	Korea	Cross sectional	female Balb/c nu/nu mice	MCF-7 cells; s.c.; right flanks	ginsenoside Rh1	D/A*	D/A*	D/A*	D/A*	(Huynh et al., 2021)
Choi 2011	Ginsenoside Rh2 induces Bcl-2 family proteins-mediated apoptosis in vitro and in xenografts in vivo models.	Korea	Cross sectional	female nude mice , 6–8 weeks	MDA-MB-231 cells; s.c. right back	ginsenoside Rh2	N/A	N/A	N/A	N/A	(Choi et al., 2011)
Duan 2018	The anti-tumor effect of ginsenoside Rh4 in MCF-7 breast cancer cells in vitro and in vivo.	China	Cross sectional	female Balb/c nu/nu mice, 4 weeks	MCF-7; s.c.; left forelimb pit	ginsenoside Rh4	N/A	N/A	D/A*	D/A*	(Duan et al., 2018)
Hong 2019 (1)	Ginsenoside Rk1 induces cell cycle arrest and apoptosis in MDA-MB-231 triple negative breast cancer cells.	China	Cross sectional	female Balb/c mice, 4 weeks	MDA-MB-231 cells	ginsenoside Rk1	D/A*	D/A*	N/A	N/A	(Hong and Fan, 2019a)

Hong 2019 (2)	Ginsenoside Rk1 induces cell death through ROS-mediated PTEN/PI3K/Akt/mTOR signaling pathway in MCF-7 cells	China	Cross section al	female Balb/c nu/nu mice	MCF-7 cells; injected;	ginsenoside Rk1	D/A*	D/A*	D/A*	D/A*	(Hong and Fan, 2019b)
Tan 2022	Gypensapogenin H suppresses tumor growth and cell migration in triple-negative breast cancer by regulating PI3K/AKT/NF-κB/MMP-9 signaling pathway	China	Cross section al	female Balb/c mice, 6 weeks	MDA-MB-231 cells; injected; right armpit	gypensapoge nin H	D/A*	D/A*	D/A*	D/A*	(Tan et al., 2022)
Blaskovic h 2003	Discovery of JSI-124 (Cucurbitacin I), a selective janus kinase/signal transducer and activator of transcription 3 signaling pathway inhibitor with potent antitumor activity against human and murine cancer cells in mice	USA	Cross section al	female nude mice, 8 weeks	MDA-MB-468 cells; s.c.; right and left flank	JSI-124	N/A	N/A	D/A*	D/A*	(Blaskovi ch et al., 2003)

He 2024	Oleanolic acid inhibits the tumor progression by regulating Lactobacillus through the cytokine-cytokine receptor interaction pathway in 4T1-induced mice breast cancer model	China	Cross sectional	female Balb/c mice, 4–6 weeks	4T1 cells; s.c.; under the flank wall	OA	D/A*	D/A*	D/A*	D/A*	(He et al., 2024)
Liang 2021	Transcriptome study of oleanolic acid in the inhibition of breast tumor growth based on high-throughput sequencing	China	Cross sectional	female Balb/c mice, 4-5 weeks	4T1 cells; s.c.; into 5~6 intercostals and the lateral abdominal wall of the chest wall	OA	D/A*	D/A*	N/A	N/A	(Liang et al., 2021)
Xu 2021	Panaxadiol as a major metabolite of AD-1 can significantly inhibit the proliferation and migration of breast cancer cells: In vitro and in vivo study	China	Cross sectional	female Balb/c mice, 6 weeks	MDA-MB-231 cells; s.c.; left armpit	panaxadiol	D/A*	D/A*	D/A*	D/A*	(Xu et al., 2021)

Kong 2016	Platycodin D, a metabolite of Platycodin grandiflorum, inhibits highly metastatic MDA-MB-231 breast cancer growth in vitro and in vivo by targeting the MDM2 oncogene	China	Cross sectional	female Balb/c nu/nu mice, 4-6 weeks	MDA-MB-231 cells; s.c.; right subaxillary area	platycodin D	D/A*	D/A*	D/A*	D/A*	(Kong et al., 2016)
Chun 2013	Platycodin D inhibits migration, invasion, and growth of MDA-MB-231 human breast cancer cells via suppression of EGFR-mediated Akt and MAPK pathways.	Korea	Cross sectional	female Balb/c nu/nu mice, 6 weeks	MDA-MB-231 cells; s.c.; right flank	platycodin D	N/A	N/A	D/A*	D/A*	(Chun and Kim, 2013)
Ye 2023	Platycodin D induces neutrophil apoptosis by downregulating PD-L1 expression to inhibit breast cancer pulmonary metastasis	China	Cross sectional	female Balb/c mice	4T1 cells inoculated into the fourth mammary fat pads	platycodin D	D/A*	D/A*	D/A*	D/A*	(Ye et al., 2023)

Cevatemre 2017	A promising natural product, pristimerin, results in cytotoxicity against breast cancer stem cells in vitro and xenografts in vivo through apoptosis and an incomplete autopaghy in breast cancer	Greece	Cross sectional	female NOD.CB17-Prkdc(scid)/J, 6-8 weeks	MCF-7 and MDA-MB-231 cells; s.c.	pristimerin	D/A*	D/A*	N/A	N/A	(Cevatemre et al., 2018)
Zhao 2019 (1)	Pristimerin induces apoptosis and autophagy via activation of ROS/ASK1/JNK pathway in human breast cancer in vitro and in vivo	China	Cross sectional	female Balb/c nu/nu mice, 4 weeks	MDA-MB-231 cells; s.c.; right flank	pristimerin	D/A*	D/A*	D/A*	D/A*	(Q. Zhao et al., 2019)
Lim 2020	Pygenic acid A (PA) sensitizes metastatic breast cancer cells to anoikis and inhibits metastasis in vivo	Korea	Cross sectional	Balb/c mice	4T1-luc cells; i.v.; tail vein	pygenic acid A	N/A	N/A	N/A	N/A	(Lim et al., 2020)
Zhao 2019 (2)	Saikosaponin A inhibits breast cancer by regulating Th1/Th2 balance	China	Cross sectional	female Sprague-Dawley rats	DMBA; intragastrical	saikosaponin A	SSa=2,80 ± 1,82 g	CMC-Na: 6,39 ± 3,6 g; tamoxifen: 2.11 ± 0.87 g	SSa=2,31 ± 0,89 mm³	CMC-Na: 6,21 ± 1,12 mm³; tamoxifen: 1.97 ± 1.26 mm³	(X. Zhao et al., 2019)

Wang 2020 (3)	Saikosaponin A Inhibits Triple-Negative Breast Cancer Growth and Metastasis Through Downregulation of CXCR4	China	Cross section al	Balb/c mice	MDA-MB-231-Luc cells; injected into the upper left mammary fat pads	saikosaponin A	N/A	N/A	N/A	N/A	(Y. Wang et al., 2020)
Yang 2024 (3)	Combination of histological and metabolomic assessments to evaluate the potential pharmacological efficacy of saikosaponin D	China	Cross section al	female Balb/c mice, 4–6 weeks	4T1 cells; s.c.; axilla of the right forelimb	SsD	N/A	N/A	N/A	N/A	(T. Yang et al., 2024)
Akl 2014	The marine-derived sipholenol A-4-O-3',4'-dichlorobenzoate inhibits breast cancer growth and motility in vitro and in vivo through the suppression of Brk and FAK signaling.	USA	Cross section al	female nude mice, 4–5 weeks	MDA-MB-231/GFP cells; injected; second mammary fat pad just beneath the nipple	sipholenol A	D/A*	D/A*	D/A*	D/A*	(Akl et al., 2014)
Huang 2021	Soyasaponin Ag inhibits triple-negative breast cancer progression via targeting the DUSP6/MAPK signaling	China	Cross section al	female Balb/c nu/nu mice, 4 weeks	MDA-MB-231 cells; s.c.; left and right thigh flanks	soyasaponin Ag	D/A*	D/A*	D/A*	D/A*	(Huang et al., 2021)

Zhang 2022 (1)	Toosendanin and isotoosendanin suppress triple-negative breast cancer growth via inducing necrosis, apoptosis and autophagy	China	Cross section al	female Balb/c mice, 4 weeks	4T1-luc-GFP cells; s.c. into the fourth right mammary fat pad at the base of the nipple	toosendanin (TSN); isotoosendani n (ITSN)	D/A*	D/A*	N/A	N/A	(Jingnan Zhang et al., 2022)
Peng 2016	Tubeimoside-1 suppresses breast cancer metastasis through downregulation of CXCR4 chemokine receptor expression	China	Cross section al	female nude mice, 6 weeks	MDA-MB-231-Luc cells; i.v.	tubeismoside-1	N/A	N/A	N/A	N/A	(Peng et al., 2016)
Zhang 2021 (2)	Identifying the Effect of Ursolic Acid Against Triple-Negative Breast Cancer: Coupling Network Pharmacology With Experiments Verification	China	Cross section al	female Balb/c nu/nu mice, 6 weeks	MDA-MB-231 cells; injected; right armpit	UA	0.5720 ± 0.11 (20 mg/kg), 0.4320 ± 0.09 (50 mg/kg), and 0.3120 ± 0.09 g (100 mg/kg)	0.776 ± 0.1647 g	460.7 ± 103.4 mm³ (20 mg/kg), 335.4 ± 123.0 mm³ (50 mg/kg), and 209.5 ± 97.90 mm³ (100 mg/kg).	686.6 ± 211.9 mm³	(Y. Zhang et al., 2021)
De Angel 2010	Antitumor effects of ursolic acid in a mouse model of postmenopausal breast cancer.	USA	Cross section al	female C57BL/6 mice, 6 weeks	MMTV-Wnt-1 cells; in the 9th mammary fat pad	UA	D/A*	D/A*	D/A*	D/A*	(De Angel et al., 2010)

Yang 2024 (2)	Ursolic acid inhibits the proliferation of triple-negative breast cancer stem-like cells through NRF2-mediated ferroptosis	China	Cross sectional	female Balb/c mice, 5 weeks	MDA-MB-231 cells; s.c.; right flank of mice.	UA	N/A	N/A	D/A*	D/A*	(X. Yang et al., 2024)
Wang 2021 (2)	Ursolic Acid Inhibits Breast Cancer Metastasis by Suppressing Glycolytic Metabolism <i>via</i> Activating SP1/Caveolin-1 Signaling	China	Cross sectional	female Balb/c mice	4T1-Luc cells; s.c. into the mammary fat pads of mice	UA	N/A	N/A	D/A*	D/A*	(S. Wang et al., 2021)
Gao 2016	Hypoxia pathway and hypoxia-mediated extensive extramedullary hematopoiesis are involved in ursolic acid's anti-metastatic effect in 4T1 tumor bearing mice.	China	Cross sectional	female Balb/c mice, 4 weeks	4T1-Luc cells; injected; mammary fat pad	UA	0.70 ± 0.11 g	1.01 ± 0.14 g	D/A*	D/A*	(Gao et al., 2016)

References Table S2

Akl, M., Foudah, A., Ebrahim, H., Meyer, S., & Sayed, K. (2014). The Marine-Derived Sipholenol A-4-O-3′,4′-Dichlorobenzoate Inhibits Breast Cancer Growth and Motility in Vitro and in Vivo through the Suppression of Brk and FAK Signaling. *Marine Drugs*, *12*(4), 2282–2304. https://doi.org/10.3390/md12042282

Al Marzouqi, N., Iratni, R., Nemmar, A., Arafat, K., Ahmed Al Sultan, M., Yasin, J., Collin, P., Mester, J., Adrian, T. E., & Attoub, S. (2011). Frondoside A inhibits human breast cancer cell survival, migration, invasion and the growth of breast tumor xenografts. *European Journal of Pharmacology*, *668*(1–2), 25–34. https://doi.org/10.1016/j.ejphar.2011.06.023

Arif, R., Bukhari, S. A., Mustafa, G., Ahmed, S., & Albeshr, M. F. (2024). Network Pharmacology and Experimental Validation to Explore the Potential Mechanism of Nigella sativa for the Treatment of Breast Cancer. *Pharmaceuticals*, *17*(5), 617. https://doi.org/10.3390/ph17050617

Blaskovich, M. A., Sun, J., Cantor, A., Turkson, J., Jove, R., & Sebti, S. M. (2003). Discovery of JSI-124 (cucurbitacin I), a selective Janus kinase/signal transducer and activator of transcription 3 signaling pathway inhibitor with potent antitumor activity against human and murine cancer cells in mice. *Cancer Research*, *63*(6), 1270–1279.

Cevatemre, B., Erkısa, M., Aztopal, N., Karakas, D., Alper, P., Tsimplouli, C., Sereti, E., Dimas, K., Armutak, E. I. I., Gurevin, E. G., Uvez, A., Mori, M., Berardozzi, S., Ingallina, C., D'Acquarica, I., Botta, B., Ozpolat, B., & Ulukaya, E. (2018). A promising natural product, pristimerin, results in cytotoxicity against breast cancer stem cells in vitro and xenografts in vivo through apoptosis and an incomplete autopaghy in breast cancer. *Pharmacological Research*, *129*, 500–514. https://doi.org/10.1016/j.phrs.2017.11.027

Cheng, Y., Zhong, X., Nie, X., Gu, H., Wu, X., Li, R., Wu, Y., Lv, K., Leung, G. P.-H., Fu, C., Lee, S. M.-Y., Zhang, J., & Li, J. (2023). Glycyrrhetinic acid suppresses breast cancer metastasis by inhibiting M2-like macrophage polarization via activating JNK1/2 signaling. *Phytomedicine*, *114*, 154757. https://doi.org/10.1016/j.phymed.2023.154757

Choi, S., Oh, J., & Kim, S. (2011). Ginsenoside Rh2 induces Bcl-2 family proteins-mediated apoptosis *in vitro* and in xenografts *in vivo* models. *Journal of Cellular Biochemistry*, *112*(1), 330–340. https://doi.org/10.1002/jcb.22932

Chu, Y., Zhang, W., Kanimozhi, G., Brindha, G. R., & Tian, D. (2020). Ginsenoside Rg1 Induces Apoptotic Cell Death in Triple-Negative Breast Cancer Cell Lines and Prevents Carcinogen-Induced Breast Tumorigenesis in Sprague Dawley Rats. *Evidence-Based Complementary and Alternative Medicine*, *2020*(1). https://doi.org/10.1155/2020/8886955

Chun, J., & Kim, Y. S. (2013). Platycodin D inhibits migration, invasion, and growth of MDA-MB-231 human breast cancer cells via suppression of EGFR-mediated Akt and MAPK pathways. *Chemico-Biological Interactions*, *205*(3), 212–221. https://doi.org/10.1016/j.cbi.2013.07.002

Damle, A. A., Pawar, Y. P., & Narkar, A. A. (2013). Anticancer activity of betulinic acid on MCF-7 tumors in nude mice. *Indian Journal of Experimental Biology*, *51*(7), 485–491.

Dasgupta, A., Sawant, M. A., Kavishwar, G., Lavhale, M., & Sitasawad, S. (2016). AECHL-1 targets breast cancer progression via inhibition of metastasis, prevention of EMT and suppression of Cancer Stem Cell characteristics. *Scientific Reports*, *6*(1), 38045. https://doi.org/10.1038/srep38045

De Angel, R. E., Smith, S. M., Glickman, R. D., Perkins, S. N., & Hursting, S. D. (2010). Antitumor Effects of Ursolic Acid in a Mouse Model of Postmenopausal Breast Cancer. *Nutrition and Cancer*, *62*(8), 1074–1086. https://doi.org/10.1080/01635581.2010.492092

Duan, Z., Wei, B., Deng, J., Mi, Y., Dong, Y., Zhu, C., Fu, R., Qu, L., & Fan, D. (2018). The anti-tumor effect of ginsenoside Rh4 in MCF-7 breast cancer cells in vitro and in vivo. *Biochemical and Biophysical Research Communications*, *499*(3), 482–487. https://doi.org/10.1016/j.bbrc.2018.03.174

Gao, J.-L., Shui, Y.-M., Jiang, W., Huang, E.-Y., Shou, Q.-Y., Ji, X., He, B.-C., Lv, G.-Y., & He, T.-C. (2016). Hypoxia pathway and hypoxia-mediated extensive extramedullary hematopoiesis are involved in ursolic acid's anti-metastatic effect in 4T1 tumor bearing mice. *Oncotarget*, *7*(44), 71802–71816. https://doi.org/10.18632/oncotarget.12375

Gou, X., Bai, H., Liu, L., Chen, H., Shi, Q., Chang, L., Ding, M., Shi, Q., Zhou, M., Chen, W., & Zhang, L. (2020). Asiatic Acid Interferes with Invasion and Proliferation of Breast Cancer Cells by Inhibiting WAVE3 Activation through PI3K/AKT Signaling Pathway. *BioMed Research International*, *2020*(1). https://doi.org/10.1155/2020/1874387

Gupta, P., & Srivastava, S. K. (2014). Inhibition of HER2-integrin signaling by Cucurbitacin B leads to *in vitro* and *in vivo* breast tumor growth suppression. *Oncotarget*, *5*(7), 1812–1828. https://doi.org/10.18632/oncotarget.1743

He, K., Meng, X., Su, J., Jiang, S., Chu, M., & Huang, B. (2024). Oleanolic acid inhibits the tumor progression by regulating Lactobacillus through the cytokine-cytokine receptor interaction pathway in 4T1-induced mice breast cancer model. *Heliyon*, *10*(5), e27028. https://doi.org/10.1016/j.heliyon.2024.e27028

Hong, Y., & Fan, D. (2019a). Ginsenoside Rk1 induces cell cycle arrest and apoptosis in MDA-MB-231 triple negative breast cancer cells. *Toxicology*, *418*, 22–31. https://doi.org/10.1016/j.tox.2019.02.010

Hong, Y., & Fan, D. (2019b). Ginsenoside Rk1 induces cell death through ROS-mediated PTEN/PI3K/Akt/mTOR signaling pathway in MCF-7 cells. *Journal of Functional Foods*, *57*, 255–265. https://doi.org/10.1016/j.jff.2019.04.019

Hu, J., Zhang, H., Li, J., Jiang, X., Zhang, Y., Wu, Q., Shen, liwen, Shi, J., & Gao, N. (2020). ROCK1 activation-mediated mitochondrial translocation of Drp1 and cofilin are required for arnidiol-induced mitochondrial fission and apoptosis. *Journal of Experimental & Clinical Cancer Research*, *39*(1), 37. https://doi.org/10.1186/s13046-020-01545-7

Hu, S., Zheng, W., & Jin, L. (2021). Astragaloside IV inhibits cell proliferation and metastasis of breast cancer via promoting the long noncoding RNA TRHDE-AS1. *Journal of Natural Medicines*, *75*(1), 156–166. https://doi.org/10.1007/s11418-020-01469-8

Huang, S., Huang, P., Wu, H., Wang, S., & Liu, G. (2021). Soyasaponin Ag inhibits triple-negative breast cancer progression via targeting the DUSP6/MAPK signaling. *Folia Histochemica et Cytobiologica*, *59*(4), 291–301. https://doi.org/10.5603/FHC.a2021.0029

Huynh, D. T. N., Jin, Y., Myung, C.-S., & Heo, K.-S. (2021). Ginsenoside Rh1 Induces MCF-7 Cell Apoptosis and Autophagic Cell Death through ROS-Mediated Akt Signaling. *Cancers*, *13*(8), 1892. https://doi.org/10.3390/cancers13081892

Jeon, H., Huynh, D. T. N., Baek, N., Nguyen, T. L. L., & Heo, K.-S. (2021). Ginsenoside-Rg2 affects cell growth via regulating ROS-mediated AMPK activation and cell cycle in MCF-7 cells. *Phytomedicine*, *85*, 153549. https://doi.org/10.1016/j.phymed.2021.153549

Jiang, K., Lu, Q., Li, Q., Ji, Y., Chen, W., & Xue, X. (2017). Astragaloside IV inhibits breast cancer cell invasion by suppressing Vav3 mediated Rac1/MAPK signaling. *International Immunopharmacology*, *42*, 195–202. https://doi.org/10.1016/j.intimp.2016.10.001

Jiao, L., Wang, S., Zheng, Y., Wang, N., Yang, B., Wang, D., Yang, D., Mei, W., Zhao, Z., & Wang, Z. (2019). Betulinic acid suppresses breast cancer aerobic glycolysis via caveolin-1/NF-κB/c-Myc pathway. *Biochemical Pharmacology*, *161*, 149–162. https://doi.org/10.1016/j.bcp.2019.01.016

Kong, Y., Lu, Z.-L., Wang, J.-J., Zhou, R., Guo, J., Liu, J., Sun, H.-L., Wang, H., Song, W., Yang, J., & Xu, H.-X. (2016). Platycodin D, a metabolite of Platycodin grandiflorum, inhibits highly metastatic MDA-MB-231 breast cancer growth in vitro and in vivo by targeting the MDM2 oncogene. *Oncology Reports*, *36*(3), 1447–1456. https://doi.org/10.3892/or.2016.4935

Li, X., Zhu, G., Yao, X., Wang, N., Hu, R., Kong, Q., Zhou, D., Long, L., Cai, J., & Zhou, W. (2018). Celastrol induces ubiquitin-dependent degradation of mTOR in breast cancer cells. *OncoTargets and Therapy*, *Volume 11*, 8977–8985. https://doi.org/10.2147/OTT.S187315

Li, Y., Wang, P., Zou, Z., Pan, Q., Li, X., Liang, Z., Li, L., Lin, Y., Peng, X., Zhang, R., Tian, H., & Han, L. (2021). Ginsenoside (20S)-protopanaxatriol induces non-protective autophagy and apoptosis by inhibiting Akt/mTOR signaling pathway in triple-negative breast cancer cells. *Biochemical and Biophysical Research Communications*, *583*, 184–191. https://doi.org/10.1016/j.bbrc.2021.10.067

Liang, J., Zhang, X., Yuan, J., Zhang, H., Liu, D., Hao, J., Ji, W., Wu, X., & Chen, D. (2019). Cucurbitacin B inhibits the migration and invasion of breast cancer cells by altering the biomechanical properties of cells. *Phytotherapy Research*, *33*(3), 618–630. https://doi.org/10.1002/ptr.6250

Liang, Z., Pan, R., Meng, X., Su, J., Guo, Y., Wei, G., Zhang, Z., & He, K. (2021). Transcriptome study of oleanolic acid in the inhibition of breast tumor growth based on high-throughput sequencing. *Aging*, *13*(19), 22883–22897. https://doi.org/10.18632/aging.203582

Lim, G.-E., Sung, J. Y., Yu, S., Kim, Y., Shim, J., Kim, H. J., Cho, M. L., Lee, J.-S., & Kim, Y.-N. (2020). Pygenic Acid A (PA) Sensitizes Metastatic Breast Cancer Cells to Anoikis and Inhibits Metastasis In Vivo. *International Journal of Molecular Sciences*, *21*(22), 8444. https://doi.org/10.3390/ijms21228444

Liu, C., Dong, L., Sun, Z., Wang, L., Wang, Q., Li, H., Zhang, J., & Wang, X. (2018). Esculentoside A suppresses breast cancer stem cell growth through stemness attenuation and apoptosis induction by blocking IL-6/STAT3 signaling pathway. *Phytotherapy Research*, *32*(11), 2299–2311. https://doi.org/10.1002/ptr.6172

Liu, Y., & Fan, D. (2018). Ginsenoside Rg5 induces apoptosis and autophagy *via* the inhibition of the PI3K/Akt pathway against breast cancer in a mouse model. *Food & Function*, *9*(11), 5513–5527. https://doi.org/10.1039/C8FO01122B

Mertens-Talcott, S. U., Noratto, G. D., Li, X., Angel-Morales, G., Bertoldi, M. C., & Safe, S. (2013). Betulinic acid decreases ER-negative breast cancer cell growth in vitro and in vivo: Role of Sp transcription factors and microRNA-27a:ZBTB10. *Molecular Carcinogenesis*, *52*(8), 591–602. https://doi.org/10.1002/mc.21893

Mu, L.-H., Wang, Y.-N., Wang, D.-X., Zhang, J., Liu, L., Dong, X.-Z., Hu, Y., & Liu, P. (2017). AG36 Inhibits Human Breast Cancer Cells Proliferation by Promotion of Apoptosis In vitro and In vivo. *Frontiers in Pharmacology*, *8*. https://doi.org/10.3389/fphar.2017.00015

Ning, J.-Y., Zhang, Z.-H., Zhang, J., Liu, Y.-M., Li, G.-C., Wang, A.-M., Li, Y., Shan, X., Wang, J.-H., Zhang, X., & Zhao, Y. (2024). Ginsenoside Rg3 decreases breast cancer stem-like phenotypes through impairing MYC mRNA stability. *American Journal of Cancer Research*, *14*(2), 601–615. https://doi.org/10.62347/GYXE7741

Peng, B., He, R., Xu, Q., Yang, Y., Hu, Q., Hou, H., Liu, X., & Li, J. (2019). Ginsenoside 20(S)-protopanaxadiol inhibits triple-negative breast cancer metastasis in vivo by targeting EGFR-mediated MAPK pathway. *Pharmacological Research*, *142*, 1–13. https://doi.org/10.1016/j.phrs.2019.02.003

Peng, Y., Zhong, Y., & Li, G. (2016). Tubeimoside-1 suppresses breast cancer metastasis through downregulation of CXCR4 chemokine receptor expression. *BMB Reports*, *49*(9), 502–507. https://doi.org/10.5483/BMBRep.2016.49.9.030

Raja, S. M., Clubb, R. J., Ortega-Cava, C., Williams, S. H., Bailey, T. A., Duan, L., Zhao, X., Reddi, A. L., Nyong, A. M., Natarajan, A., Band, V., & Band, H. (2011). Anticancer activity of Celastrol in combination with ErbB2-targeted therapeutics for treatment of ErbB2-overexpressing breast cancers. *Cancer Biology & Therapy*, *11*(2), 263–276. https://doi.org/10.4161/cbt.11.2.13959

Sawant, M. A., Dasgupta, A., Lavhale, M. S., & Sitasawad, S. L. (2016). Novel triterpenoid AECHL-1 induces apoptosis in breast cancer cells by perturbing the mitochondria–endoplasmic reticulum interactions and targeting diverse apoptotic pathways. *Biochimica et Biophysica Acta (BBA) - General Subjects*, *1860*(6), 1056–1070. https://doi.org/10.1016/j.bbagen.2016.02.002

Sinha, S., Khan, S., Shukla, S., Lakra, A. D., Kumar, S., Das, G., Maurya, R., & Meeran, S. M. (2016). Cucurbitacin B inhibits breast cancer metastasis and angiogenesis through VEGF-mediated suppression of FAK/MMP-9 signaling axis. *The International Journal of Biochemistry & Cell Biology*, *77*, 41–56. https://doi.org/10.1016/j.biocel.2016.05.014

Song, J.-H., Eum, D.-Y., Park, S.-Y., Jin, Y.-H., Shim, J.-W., Park, S.-J., Kim, M.-Y., Park, S.-J., Heo, K., & Choi, Y.-J. (2020). Inhibitory effect of ginsenoside Rg3 on cancer stemness and mesenchymal transition in breast cancer via regulation of myeloid-derived suppressor cells. *PLOS ONE*, *15*(10), e0240533. https://doi.org/10.1371/journal.pone.0240533

Tan, H., Zhang, M., Xu, L., Zhang, X., & Zhao, Y. (2022). Gypensapogenin H suppresses tumor growth and cell migration in triple-negative breast cancer by regulating PI3K/AKT/NF-κB/MMP-9 signaling pathway. *Bioorganic Chemistry*, *126*, 105913. https://doi.org/10.1016/j.bioorg.2022.105913

Wakimoto, N., Yin, D., O'Kelly, J., Haritunians, T., Karlan, B., Said, J., Xing, H., & Koeffler, H. P. (2008). Cucurbitacin B has a potent antiproliferative effect on breast cancer cells *in vitro* and *in vivo*. *Cancer Science*, *99*(9), 1793–1797. https://doi.org/10.1111/j.1349-7006.2008.00899.x

Wang, S., Chang, X., Zhang, J., Li, J., Wang, N., Yang, B., Pan, B., Zheng, Y., Wang, X., Ou, H., & Wang, Z. (2021). Ursolic Acid Inhibits Breast Cancer Metastasis by Suppressing Glycolytic Metabolism via Activating SP1/Caveolin-1 Signaling. *Frontiers in Oncology*, *11*. https://doi.org/10.3389/fonc.2021.745584

Wang, W., Zhang, X., Qin, J.-J., Voruganti, S., Nag, S. A., Wang, M.-H., Wang, H., & Zhang, R. (2012). Natural Product Ginsenoside 25-OCH3-PPD Inhibits Breast Cancer Growth and Metastasis through Down-Regulating MDM2. *PLoS ONE*, *7*(7), e41586. https://doi.org/10.1371/journal.pone.0041586

Wang, Y., Zhao, L., Han, X., Wang, Y., Mi, J., Wang, C., Sun, D., Fu, Y., Zhao, X., Guo, H., & Wang, Q. (2020). Saikosaponin A Inhibits Triple-Negative Breast Cancer Growth and Metastasis Through Downregulation of CXCR4. *Frontiers in Oncology*, *9*. https://doi.org/10.3389/fonc.2019.01487

Wu, X.-X., Yue, G. G.-L., Dong, J.-R., Lam, C. W.-K., Wong, C.-K., Qiu, M.-H., & Lau, C. B.-S. (2020). Actein Inhibits Tumor Growth and Metastasis in HER2-Positive Breast Tumor Bearing Mice via Suppressing AKT/mTOR and Ras/Raf/MAPK Signaling Pathways. *Frontiers in Oncology*, *10*. https://doi.org/10.3389/fonc.2020.00854

Xu, L., Zhang, X., Xiao, S., Li, X., Jiang, H., Wang, Z., Sun, B., & Zhao, Y. (2021). Panaxadiol as a major metabolite of AD-1 can significantly inhibit the proliferation and migration of breast cancer cells: In vitro and in vivo study. *Bioorganic Chemistry*, *116*, 105392. https://doi.org/10.1016/j.bioorg.2021.105392

Yang, C., Qian, C., Zheng, W., Dong, G., Zhang, S., Wang, F., Wei, Z., Xu, Y., Wang, A., Zhao, Y., & Lu, Y. (2024). Ginsenoside Rh2 enhances immune surveillance of natural killer (NK) cells via inhibition of ERp5 in breast cancer. *Phytomedicine*, *123*, 155180. https://doi.org/10.1016/j.phymed.2023.155180

Yang, T., Li, X., Wang, X., Meng, X., Zhang, Z., Zhao, M., & Su, R. (2024). Combination of histological and metabolomic assessments to evaluate the potential pharmacological efficacy of saikosaponin D. *Journal of Pharmaceutical and Biomedical Analysis*, *242*, 116001. https://doi.org/10.1016/j.jpba.2024.116001

Yang, X., Liang, B., Zhang, L., Zhang, M., Ma, M., Qing, L., Yang, H., Huang, G., & Zhao, J. (2024). Ursolic acid inhibits the proliferation of triple-negative breast cancer stem-like cells through NRF2-mediated ferroptosis. *Oncology Reports*, *52*(1), 94. https://doi.org/10.3892/or.2024.8753

Yang, Y., Long, L., Zhang, X., Song, K., Wang, D., Xiong, X., Gao, H., & Sha, L. (2019). 16-Tigloyl linked barrigenol-like triterpenoid from Semen Aesculi and its anti-tumor activity *in vivo* and *in vitro*. *RSC Advances*, *9*(54), 31758–31772. https://doi.org/10.1039/C9RA06015D

Ye, Y., Xie, Y., Pei, L., Jiang, Z., Wu, C., & Liu, S. (2023). Platycodin D induces neutrophil apoptosis by downregulating PD-L1 expression to inhibit breast cancer pulmonary metastasis. *International Immunopharmacology*, *115*, 109733. https://doi.org/10.1016/j.intimp.2023.109733

Yin, L., Fan, Z., Liu, P., Chen, L., Guan, Z., Liu, Y., & Luo, Y. (2021). Anemoside A3 activates TLR4-dependent M1-phenotype macrophage polarization to represses breast tumor growth and angiogenesis. *Toxicology and Applied Pharmacology*, *432*, 115755. https://doi.org/10.1016/j.taap.2021.115755

Yue, G. G.-L., Xie, S., Lee, J. K.-M., Kwok, H.-F., Gao, S., Nian, Y., Wu, X.-X., Wong, C.-K., Qiu, M.-H., & Lau, C. B.-S. (2016). New potential beneficial effects of actein, a triterpene glycoside isolated from Cimicifuga species, in breast cancer treatment. *Scientific Reports*, *6*(1), 35263. https://doi.org/10.1038/srep35263

Zhang, B., Fu, R., Duan, Z., Shen, S., Zhu, C., & Fan, D. (2022). Ginsenoside CK induces apoptosis in triple-negative breast cancer cells by targeting glutamine metabolism. *Biochemical Pharmacology*, *202*, 115101. https://doi.org/10.1016/j.bcp.2022.115101

Zhang, E., Shi, H., Yang, L., Wu, X., & Wang, Z. (2017). Ginsenoside Rd regulates the Akt/mTOR/p70S6K signaling cascade and suppresses angiogenesis and breast tumor growth. *Oncology Reports*, *38*(1), 359–367. https://doi.org/10.3892/or.2017.5652

Zhang, H., Xu, H.-L., Wang, Y.-C., Lu, Z.-Y., Yu, X.-F., & Sui, D.-Y. (2018). 20(S)-Protopanaxadiol-Induced Apoptosis in MCF-7 Breast Cancer Cell Line through the Inhibition of PI3K/AKT/mTOR Signaling Pathway. *International Journal of Molecular Sciences*, *19*(4), 1053. https://doi.org/10.3390/ijms19041053

Zhang, J., He, Y., Zhou, Y., Hong, L., Jiang, Z., Zhao, Y., & Pan, Z. (2022). Epifriedelinol Ameliorates DMBA-Induced Breast Cancer in Albino Rats by Regulating the PI3K/AKT Pathway. *The Tohoku Journal of Experimental Medicine*, *257*(4), 2022.J030. https://doi.org/10.1620/tjem.2022.J030

Zhang, J., Yang, F., Mei, X., Yang, R., Lu, B., Wang, Z., & Ji, L. (2022). Toosendanin and isotoosendanin suppress triple-negative breast cancer growth via inducing necrosis, apoptosis and autophagy. *Chemico-Biological Interactions*, *351*, 109739. https://doi.org/10.1016/j.cbi.2021.109739

Zhang, J., Zhou, B., Sun, J., Chen, H., & Yang, Z. (2021). Betulin ameliorates 7,12-dimethylbenz(a)anthracene-induced rat mammary cancer by modulating MAPK and AhR/Nrf-2 signaling pathway. *Journal of Biochemical and Molecular Toxicology*, *35*(7). https://doi.org/10.1002/jbt.22779

Zhang, Y., Ma, X., Li, H., Zhuang, J., Feng, F., Liu, L., Liu, C., & Sun, C. (2021). Identifying the Effect of Ursolic Acid Against Triple-Negative Breast Cancer: Coupling Network Pharmacology With Experiments Verification. *Frontiers in Pharmacology*, *12*. https://doi.org/10.3389/fphar.2021.685773

Zhao, Q., Liu, Y., Zhong, J., Bi, Y., Liu, Y., Ren, Z., Li, X., Jia, J., Yu, M., & Yu, X. (2019). Pristimerin induces apoptosis and autophagy via activation of ROS/ASK1/JNK pathway in human breast cancer in vitro and in vivo. *Cell Death Discovery*, *5*(1), 125. https://doi.org/10.1038/s41420-019-0208-0

Zhao, X., Liu, J., Ge, S., Chen, C., Li, S., Wu, X., Feng, X., Wang, Y., & Cai, D. (2019). Saikosaponin A Inhibits Breast Cancer by Regulating Th1/Th2 Balance. *Frontiers in Pharmacology*, *10*. https://doi.org/10.3389/fphar.2019.00624

Table S3. Triterpenoids (D/A*- data available within the source article in graphical form, N/A- data not available)

**Name of the first author and year of publication**	**Title of the article**	**Country**	**Study type**	**Animal model (sex, type, age)**	**Type of breast cancer inoculated and tumor inoculation method**	**Triterpenoid**	**Tumor weight (tested compound)**	**Tumor weight (control)**	**Tumor volume (tested compounds)**	**Tumor volume (control)**	**Reference**

Kallepu 2020	sp(3) -Rich Glycyrrhetinic Acid Analogues Using Late-Stage Functionalization as Potential Breast Tumor Regressing Agents.	India	Cross sectional	SCID mice	MDA-MB-231 cells	GA derivates	D/A*	D/A*	D/A*	D/A*	(Kallepu et al., 2020)
Yan 2018	Lup-20(29)-en-3β,28-di-yl-nitrooxy acetate affects MCF-7 proliferation through the crosstalk between apoptosis and autophagy in mitochondria.	China	Cross sectional	female Balb/c nu/nu mice	MCF-7 cells; s.c.; left axillae	lup-20(29)-en-3β,28-di-yl-nitrooxy acetate (NBT)	N/A	N/A	D/A*	D/A*	(Yan et al., 2018)
Saha 2016	Role of metabolic modulator Bet-CA in altering mitochondrial hyperpolarization to suppress cancer associated angiogenesis and metastasis	India	Cross sectional	female Balb/c mice	4T1 cells; injected in the upper fourth inguinal fat pad	Bet-CA: An ester derivative of betulinic acid and dichloroacetate	N/A	N/A	D/A*	D/A*	(Saha et al., 2016)

Gan 2024	Proteolysis Targeting Chimeras (PROTACs) based on celastrol induce multiple protein degradation for triple-negative breast cancer treatment	China	Cross section al	female Balb/c mice, 6 weeks	4T1 cells; injected; dorsal flank	Celastrol-based Proteolysis Targeting Chimeras (PROTACs): compound 6a	D/A*	D/A*	D/A*	D/A*	(Gan et al., 2024)
Abdelmone em 2021	Lactoferrin-dual drug nanoconjugate: Synergistic anti-tumor efficacy of docetaxel and the NF-κB inhibitor celastrol	Egypt	Cross section al	female Balb/c mice, 7-8 weeks	EAT cells; injected; left side mammary fat pad	lactoferin-celastrol-doxorubicin nanoconjugate	N/A	N/A	D/A*	D/A*	(Abdelmone em et al., 2021)
Suebsakwo ng 2019	A Bioreductive Prodrug of CuB Significantly Inhibits Tumor Growth in the 4T1 Xenograft Mice Model	Thailan d	Cross section al	female Balb/c mice	4T1 cells; s.c. injected into the right and left flanks	prodrug 1 of CuB	D/A*	D/A*	D/A*	D/A*	(Suebsakwo ng et al., 2019)

Lee 2018	The ginsenoside metabolite compound K inhibits hormone-independent breast cancer through downregulation of cyclin D1	Korea	Cross section al	female nude mice, 5 weeks	MCF10DCIS cells; s.c.; in the flank.	20-O-beta-D-glucopyranosyl-20(S)-proto-panaxa-diol (compound K, CK)	D/A*	D/A*	D/A*	D/A*	(Lee et al., 2018)
Farhangfar 2022	In vivo study of anticancer activity of ginsenosideRh2-containing arginine-reduced graphene in a mouse model of breast cancer	Iran	Cross section al	female Balb/c mice, 5–6 weeks	4T1 cells; injected; left flanks	ginsenoside Rh2-containing arginine-reduced graphene	0.34 ± 0.16 g	PBS: 1.86 ± 0.19 g, Rh2: 0.36 ± 0.21 g, graphene-arginine: 0.63 ± 0.18 g	D/A*	D/A*	(Farhangfar et al., 2022b)

Lubet 2016	5MeCDDO Blocks Metabolic Activation but not Progression of Breast, Intestine, and Tongue Cancers. Is Antioxidant Response Element a Prevention Target?	USA	Cross sectional	female Sprague-Dawley rats, 50 days (MNU model) and 43 days (DMBA model)	MNU Model: BW; injection; jugular vein DMBA Model: DMBA; p.o.	2-cyano-3,12-dioxooleana-1,9(11)-dien-28-oic acid (CDDO-Me)	139 mg (DMBA model, 27 mg/kg 5MeCCDO); 551 mg (DMBA model, 2.7 mg/kg 5MeCCDO); 19.2 mg (MNUmodel)	no treatment: 900 mg; 5,6-benzoflavone: 67 mg	N/A	N/A	(Lubet et al., 2016)
Tran 2012 (2)	The synthetic triterpenoid CDDO-methyl ester delays estrogen receptor-negative mammary carcinogenesis in polyoma middle T mice.	USA	Cross sectional	female PyMT mice, 4 weeks	transgenic mice	2-cyano-3,12-dioxooleana-1,9(11)-dien-28-oic acid (CDDO-Me)	N/A	N/A	N/A	N/A	(Tran et al., 2012)
Zhou 2020	CDDO-me elicits anti-breast cancer activity by targeting LRP6 and FZD7 receptor complex	China	Cross sectional	female Balb/c nu/nu mice	MMTV-Wnt1 tumors (from transgenic mice); s.c.	2-cyano-3,12-dioxooleana-1,9(11)-dien-28-oic acid (CDDO-Me)	D/A*	D/A*	D/A*	D/A*	(Zhou et al., 2020)

Kim 2011	CDDO-Methyl Ester Delays Breast Cancer Development in Brca1 -Mutated Mice	China	Cross sectional	female Brca1(Co/Co);MMTV-Cre;p53(+/-) mice	transgenic mice	2-cyano-3,12-dioxooleana-1,9(11)-dien-28-oic acid (CDDO-Me)	N/A	N/A	N/A	N/A	(Kim et al., 2012)
Ball 2020	CDDO-Me Alters the Tumor Microenvironment in Estrogen Receptor Negative Breast Cancer	USA	Cross sectional	female PyMT mice, 4 weeks	transgenic mice	bardoxolone methyl (CDDO-Me)	N/A	N/A	N/A	N/A	(Ball et al., 2020)
Wang 2022 (1)	Rab13 Sustains Breast Cancer Stem Cells by Supporting Tumor-Stroma Cross-talk	China	Cross sectional	female MMTV-PyMT mice and female Balb/c nu/nu mice , 7-8 weeks	MDA-MB-231 cells; into the mammary fat pads of nude mice PYMT-MMTV-transgenic mice	bardoxolone methyl (CDDO-Me)	N/A	N/A	D/A*	D/A*	(H. Wang et al., 2022)

Feng 2020	The Novel Synthetic Triterpene Methyl 3β-O-[4-(2-Aminoethylamino)-4-oxo-butyryl]olean-12-ene-28-oate Inhibits Breast Tumor Cell Growth in Vitro and in Vivo.	China	Cross sectional	female Balb/c mice, 4–6 weeks	MCF-7 cells s.c.	methyl 3β-O-[4-(2-aminoethylamino)-4-oxo-butyryl]olean-12-ene-28-oate (DABO-Me)	N/A	N/A	206 ± 23 mm^3	360 ± 35 mm^3	(Feng et al., 2020)
Bishayee 2013	Chemopreventive effect of a novel oleanane triterpenoid in a chemically induced rodent model of breast cancer.	USA	Cross sectional	female Sprague-Dawley rats, ~36 days	DMBA; p.o.	methyl-25-hydroxy-3-oxoolean-12-en-28-oate (AMR-Me)	AMR-Me (0.8 mg/kg): 11.5 ± 4.9 g; AMR-Me (1.2 mg/kg): 3.2 ± 1.5 g ;AMR-Me (1.6 mg/kg): 0.38 ± 0.03 g	20.8 ± 4.6 g	N/A	N/A	(Bishayee et al., 2013)

Mandal 2013 (1)	Simultaneous disruption of estrogen receptor and Wnt/β-catenin signaling is involved in methyl amooranin-mediated chemopreventio n of mammary gland carcinogenesis in rats.	USA	Cross section al	female Sprague–Dawley rats, 43 days	DMBA; p.o.	methyl-amoorain (methyl-25-hydroxy-3-oxoo-lean-12-en-28-oate) (AMR-Me)	N/A	N/A	N/A	N/A	(Mandal et al., 2013)
Mandal 2013 (2)	Suppression of inflammatory cascade is implicated in methyl amooranin-mediated inhibition of experimental mammary carcinogenesis.	USA	Cross section al	female Sprague–Dawley rats, ~43 days	DMBA; p.o.	methyl-amooranin (methyl-25-hydroxy-3-oxoolean-12-en-28-oate) (AMR-Me)	N/A	N/A	N/A	N/A	(Mandal et al., 2014)

References Table S3

Abdelmoneem, M. A., Abd Elwakil, M. M., Khattab, S. N., Helmy, M. W., Bekhit, A. A., Abdulkader, M. A., Zaky, A., Teleb, M., Elkhodairy, K. A., Albericio, F., & Elzoghby, A. O. (2021). Lactoferrin-dual drug nanoconjugate: Synergistic anti-tumor efficacy of docetaxel and the NF-κB inhibitor celastrol. *Materials Science and Engineering: C*, *118*, 111422. https://doi.org/10.1016/j.msec.2020.111422

Ball, M. S., Bhandari, R., Torres, G. M., Martyanov, V., ElTanbouly, M. A., Archambault, K., Whitfield, M. L., Liby, K. T., & Pioli, P. A. (2020). CDDO-Me Alters the Tumor Microenvironment in Estrogen Receptor Negative Breast Cancer. *Scientific Reports*, *10*(1), 6560. https://doi.org/10.1038/s41598-020-63482-x

Bishayee, A., Mandal, A., Thoppil, R. J., Darvesh, A. S., & Bhatia, D. (2013). Chemopreventive effect of a novel oleanane triterpenoid in a chemically induced rodent model of breast cancer. *International Journal of Cancer*, *133*(5), 1054–1063. https://doi.org/10.1002/ijc.28108

Farhangfar, S. D., Fesahat, F., Zare-Zardini, H., Dehghan-Manshadi, M., Zare, F., Miresmaeili, S. M., Vajihinejad, M., & Soltaninejad, H. (2022). In vivo study of anticancer activity of ginsenoside Rh2-containing arginine-reduced graphene in a mouse model of breast cancer. *Iranian Journal of Basic Medical Sciences*, *25*(12), 1442–1451. https://doi.org/10.22038/IJBMS.2022.66065.14524

Feng, B., Zhao, C., Li, J., Yu, J., Zhang, Y., Zhang, X., Tian, T., & Zhao, L. (2020). The Novel Synthetic Triterpene Methyl 3β-O-[4-(2-Aminoethylamino)-4-oxo-butyryl]olean-12-ene-28-oate Inhibits Breast Tumor Cell Growth in Vitro and in Vivo. *Chemical and Pharmaceutical Bulletin*, *68*(10), 962–970. https://doi.org/10.1248/cpb.c20-00353

Gan, X., Wang, F., Luo, J., Zhao, Y., Wang, Y., Yu, C., & Chen, J. (2024). Proteolysis Targeting Chimeras (PROTACs) based on celastrol induce multiple protein degradation for triple-negative breast cancer treatment. *European Journal of Pharmaceutical Sciences*, *192*, 106624. https://doi.org/10.1016/j.ejps.2023.106624

Kallepu, S., Neeli, P. K., Mallappa, S., Nagendla, N. K., Reddy Mudiam, M. K., Mainkar, P. S., Kotamraju, S., & Chandrasekhar, S. (2020). sp 3 - Rich Glycyrrhetinic Acid Analogues Using Late-Stage Functionalization as Potential Breast Tumor Regressing Agents. *ChemMedChem*, *15*(19), 1826–1833. https://doi.org/10.1002/cmdc.202000400

Kim, E.-H., Deng, C., Sporn, M. B., Royce, D. B., Risingsong, R., Williams, C. R., & Liby, K. T. (2012). CDDO-Methyl Ester Delays Breast Cancer Development in *Brca1* -Mutated Mice. *Cancer Prevention Research*, *5*(1), 89–97. https://doi.org/10.1158/1940-6207.CAPR-11-0359

Lee, S. J., Lee, J. S., Lee, E., Lim, T.-G., & Byun, S. (2018). The ginsenoside metabolite compound K inhibits hormone-independent breast cancer through downregulation of cyclin D1. *Journal of Functional Foods*, *46*, 159–166. https://doi.org/10.1016/j.jff.2018.04.050

Lubet, R. A., Townsend, R., Clapper, M. L., Juliana, M. M., Steele, V. E., McCormick, D. L., & Grubbs, C. J. (2016). 5MeCDDO Blocks Metabolic Activation but not Progression of Breast, Intestine, and Tongue Cancers. Is Antioxidant Response Element a Prevention Target? *Cancer Prevention Research*, *9*(7), 616–623. https://doi.org/10.1158/1940-6207.CAPR-15-0294

Mandal, A., Bhatia, D., & Bishayee, A. (2013). Simultaneous disruption of estrogen receptor and Wnt/β-catenin signaling is involved in methyl amooranin-mediated chemoprevention of mammary gland carcinogenesis in rats. *Molecular and Cellular Biochemistry*, *384*(1–2), 239–250. https://doi.org/10.1007/s11010-013-1803-7

Mandal, A., Bhatia, D., & Bishayee, A. (2014). Suppression of inflammatory cascade is implicated in methyl amooranin-mediated inhibition of experimental mammary carcinogenesis. *Molecular Carcinogenesis*, *53*(12), 999–1010. https://doi.org/10.1002/mc.22067

Saha, S., Ghosh, M., & Dutta, S. K. (2016). Role of metabolic modulator Bet-CA in altering mitochondrial hyperpolarization to suppress cancer associated angiogenesis and metastasis. *Scientific Reports*, *6*(1), 23552. https://doi.org/10.1038/srep23552

Suebsakwong, P., Wang, J., Khetkam, P., Weerapreeyakul, N., Wu, J., Du, Y., Yao, Z.-J., Li, J.-X., & Suksamrarn, A. (2019). A Bioreductive Prodrug of Cucurbitacin B Significantly Inhibits Tumor Growth in the 4T1 Xenograft Mice Model. *ACS Medicinal Chemistry Letters*, *10*(10), 1400–1406. https://doi.org/10.1021/acsmedchemlett.9b00161

Tran, K., Risingsong, R., Royce, D., Williams, C. R., Sporn, M. B., & Liby, K. (2012). The Synthetic Triterpenoid CDDO-Methyl Ester Delays Estrogen Receptor–Negative Mammary Carcinogenesis in Polyoma Middle T Mice. *Cancer Prevention Research*, *5*(5), 726–734. https://doi.org/10.1158/1940-6207.CAPR-11-0404

Wang, H., Xu, H., Chen, W., Cheng, M., Zou, L., Yang, Q., Chan, C. B., Zhu, H., Chen, C., Nie, J., & Jiao, B. (2022). Rab13 Sustains Breast Cancer Stem Cells by Supporting Tumor–Stroma Cross-talk. *Cancer Research*, *82*(11), 2124–2140. https://doi.org/10.1158/0008-5472.CAN-21-4097

Yan, X., Yang, L., Feng, G., Yu, Z., Xiao, M., Cai, W., Xing, Y., Bai, S., Guo, J., Wang, Z., Wang, T., & Zhang, R. (2018). Lup-20(29)-en-3β,28-di-yl-nitrooxy acetate affects MCF-7 proliferation through the crosstalk between apoptosis and autophagy in mitochondria. *Cell Death & Disease*, *9*(2), 241. https://doi.org/10.1038/s41419-017-0255-5

Zhou, L., Wang, Z., Yu, S., Xiong, Y., Fan, J., Lyu, Y., Su, Z., Song, J., Liu, S., Sun, Q., & Lu, D. (2020). CDDO-Me Elicits Anti–Breast Cancer Activity by Targeting LRP6 and FZD7 Receptor Complex. *The Journal of Pharmacology and Experimental Therapeutics*, *373*(1), 149–159. https://doi.org/10.1124/jpet.119.263434

Table S4. Associations (D/A*- data available within the source article in graphical form, N/A- data not available)

Name of the first author and year of publication	Title of the article	Country	Study type	Animal model (sex, type, age)	Type of breast cancer inoculated and tumor inoculation method	Association tested	Tumor weight (tested compound)	Tumor weight (control)	Tumor volume (tested compounds)	Tumor volume (control)	Reference

Hyer 2005	Synthetic triterpenoids cooperate with tumor necrosis factor-related apoptosis-inducing ligand to induce apoptosis of breast cancer cells	USA	Cross sectional	female Balb/c nu/nu mice, 4 weeks	MDA-MB-468 cells; s.c.; the flanks	1-(2-cyano-3,12-dioxooleana-1,9-dien-28-oyl) imidazole (CDDO-Im); CDDO-Im+TRAIL	N/A	N/A	D/A*	D/A*	(Hyer et al., 2005)
Shi 2020	Synergistic breast cancer suppression efficacy of DOX by combination with glycyrrhetinic acid as an angiogenesis inhibitor	China	Cross sectional	female Balb/c mice	4T1 cells; s.c.	GA+ DOX	D/A*	D/A*	N/A	N/A	(Shi et al., 2021)
Liby 2008	Prevention and treatment of experimental estrogen receptor-negative mammary carcinogenesis by the synthetic triterpenoid CDDO-methyl Ester and the rexinoid LG100268.	USA	Cross sectional	female MMTV-neu mice, 10 weeks	transgenic mice	2-cyano-3,12-dioxooleana-1,9(11)-dien-28-oic acid methyl ester (CDDO-Me)+ rexinoid LG100268	N/A	N/A	N/A	N/A	(Liby et al., 2008)
Zheng 2018	Astragaloside IV enhances taxol chemosensitivity of breast cancer via caveolin-1-	China	Cross sectional	female Balb/c nu/nu mice, 5 weeks	MDA-MB-231 cells; injected; mammary fat pads	AS-IV+ taxol	N/A	N/A	D/A*	D/A*	(Zheng et al., 2019)

	targeting oxidant damage										
Cai 2018	Betulinic acid chemosensitizes breast cancer by triggering ER stress-mediated apoptosis by directly targeting GRP78.	China	Cross sectional	female Balb/c nu/nu mice, 5 weeks	MDA-MB-231 cells; injected; mammary fat pads	BA+ taxol	N/A	N/A	D/A*	D/A*	(Cai et al., 2018)
So 2013	Oral administration of a gemini vitamin D analog, a synthetic triterpenoid and the combination prevents mammary tumorigenesis driven by ErbB2 overexpression.	USA	Cross sectional	female MMTV-ErbB2/neu mice, 6–7 weeks	transgenic mice	CDDO-Im+ BXL0124	D/A*	D/A*	N/A	N/A	(So et al., 2013)
Wang 2021 (1)	The Synergistic Effects of Celastrol in combination with Tamoxifen on Apoptosis and Autophagy in MCF-7 Cells.	China	Cross sectional	female Balb/c nu/nu mice, 6 weeks	MCF-7 cells; s.c.; on the back	Cel+ tamoxifen	D/A*	D/A*	D/A*	D/A*	(L. Wang et al., 2021)

Aribi 2012	The triterpenoid CuB augments the antiproliferative activity of chemotherapy in human breast cancer.	USA	Cross sectional	female nude mice, 5 weeks	MDA-MB-231 cells; injected; mammary fat pads	CuB+ gemcitabine; CuB+ docetaxel	N/A	N/A	D/A*	D/A*	(Aribi et al., 2013)
Jin 2018	Higenamine enhances the antitumor effects of CuB in breast cancer by inhibiting the interaction of AKT and CDK2.	China	Cross sectional	female Balb/c nu/nu mice, 3-4 weeks	SKBr3 cells; s.c.; implanted; right flank	CuB + higenamine	D/A*	D/A*	D/A*	D/A*	(Jin et al., 2018)
Nakhjavani 2021	Anti-Cancer Effects of an Optimised Combination of Ginsenoside Rg3 Epimers on Triple Negative Breast Cancer Models	Australi a	Cross sectional	female NSG mice, 6–8 weeks	MDA-MB-231-Luc cells; injected; 4th right mammary fat pad	epimers of Rg3 (20(S)-Rg3-SRg3 and 20(R)-Rg3 RRg3)	N/A	N/A	D/A*	D/A*	(Nakhjavani et al., 2021)
Luo 2024 (1)	Ganoderic acid D attenuates gemcitabine resistance of triple-negative breast cancer cells by inhibiting glycolysis via HIF-1 α destabilization	China	Cross sectional	female Balb/c nu/nu mice, 4 weeks	BT20 cells; s.c; right side	ganoderic acid D+ gemcitabine	D/A*	D/A*	D/A*	D/A*	(B. Luo et al., 2024)

Chang 2024	Ginsenoside Rg3 combined with near-infrared photothermal reversal of multidrug resistance in breast cancer MCF-7/ADR cells	China	Cross sectional	female Balb/c mice, 5 weeks	MCF-7/adr cells; i.p.; right fat pad of the fourth mammary gland	ginsenoside Rg3+ adryamicin (DOX)+near-infrared radiation	N/A	N/A	D/A*	D/A*	(Chang et al., 2024)
Zhang 2008	Antiangiogenic effect of capecitabine combined with ginsenoside Rg3 on breast cancer in mice.	China	Cross sectional	female Balb/c mice, 6 weeks	4T1 cells; injected; mammary fat pad in the right flank	ginsenoside Rg3+ capecitabine	N/A	N/A	D/A*	D/A*	(Zhang et al., 2008)
Zhang 2016	Inhibiting effect of Endostar combined with ginsenoside Rg3 on breast cancer tumor growth in tumor-bearing mice.	China	Cross sectional	female C57 mice	MCF-7 cells; inoculated under right mammary gland	ginsenoside Rg3+ endostatin	N/A	N/A	Rg3 + endostatin: 337.28 ± 42.78	saline: 723.67 ± 81.51; Rg3: 492.33 ± 47.22; endostatin: 485.29 ± 61.17	(Zhang et al., 2016)

Yuan 2017	Ginsenoside Rg3 promotes cytotoxicity of PTX through inhibiting NF-κB signaling and regulating Bax/Bcl-2 expression on triple-negative breast cancer.	China	Cross sectional	male Balb/c nu/nu mice, 5 weeks	MDA-MB-231 cells; s.c.; into nude mice.	ginsenoside Rg3+ PTX	D/A*	D/A*	D/A*	D/A*	(Yuan et al., 2017)
Liu 2020	Ginsenoside Rh2 pretreatment and withdrawal reactivated the pentose phosphate pathway to ameliorate intracellular redox disturbance and promoted intratumoral penetration of adriamycin	China	Cross sectional	female Balb/c mice, 8-10 weeks	MCF-7 cells; s.c.; right flank	ginsenoside Rh2+ DOX	N/A	N/A	D/A*	D/A*	(Liu et al., 2020)
Zhang 2023 (1)	Isotoosendanin exerts inhibition on triple-negative breast cancer through abrogating TGF-β-induced epithelial–mesenchymal transition via directly targeting	China	Cross sectional	female Balb/c mice, 4 weeks	MDA-MB-231 cells; MDA-MB-231-TGFbR1 cells; MDA-MB-231-shTGFbR1 cells; BT549	isotoosendanin (ITSN)+ programmed cell death 1 ligand 1 antibody (PD-L1)	D/A*	D/A*	N/A	N/A	(Jingnan Zhang et al., 2023)

	TGFβR1				cells; 4T1 cells						
Xu 2022	Oleanolic acid combined with olaparib enhances radiosensitization in triple negative breast cancer and hypoxia imaging with ¹⁸F -FETNIM micro PET/CT	China	Cross sectional	female Balb/c nu/nu mice, 4 weeks	MDA-MB-231 cells; s.c.; right breast region	OA+ irradiation; OA+ olaparib + irradiation	N/A	N/A	D/A*	D/A*	(Xu et al., 2022)
Wang 2019	Synergistic anti-breast cancer effect of pulsatilla saponin D and camptothecin through interrupting autophagic-lysosomal function and promoting p62-mediated ubiquitinated protein aggregation	China	Cross sectional	female nude mice	MCF-7 and MDA-MB-231 cells; inoculated; armpit area of mice	pulsatilla saponin D (PSD)+ camptothecin	N/A	N/A	N/A	N/A	(K. Wang et al., 2020)

Li 2017	The effect of saikosaponin D on DOX pharmacokinetics and its MDR reversal in MCF-7/adr cell xenografts.	China	Cross sectional	Balb/c nu/nu mice, 4-6 weeks	MCF-7/adr cells; s.c.; the back	SSd+ DOX	D/A*	D/A*	D/A*	D/A*	(Li et al., 2017)
Luo 2024 (2)	Saikosaponin D potentiates the antineoplastic effects of DOX in drug-resistant breast cancer through perturbing NQO1-mediated intracellular redox balance	China	Cross sectional	nude mice	MCF-7/DOX cells	SsD + DOX	SSD 5 mg/kg + DOX: 0.19; SSD 10 mg/kg + DOX: 0.15	saline: 0.31 g; SSD 10 mg/kg: 0.21 g; DOX: 0.20 g	D/A*	saline: 381.86 mm^3; SSD 10 mg/kg: 239 mm^3; DOX: 212.54 mm^3	(F. Luo et al., 2024)
Zhang 2022 (2)	Toosendanin, a late-stage autophagy inhibitor, sensitizes triple-negative breast cancer to irinotecan chemotherapy	China	Cross sectional	nude mice, 4-5 weeks	MDA-MB-231 cells; s.c.; right armpit	toosendanin (TSN)+ irinotecan	D/A*	D/A*	D/A*	D/A*	(S. Zhang et al., 2022)
Kai 2018	Natural product toosendanin reverses the resistance of human breast cancer cells to adriamycin as a	China	Cross sectional	female Balb/c mice, 5 weeks	4T1 cells; right flank abdominal mammary fat pad	toosendanin + DOX	N/A	N/A	D/A*	D/A*	(Kai et al., 2018)

	novel PI3K inhibitor										
Zhang 2023 (2)	Synergistic Anti-Tumor Effect of Toosendanin and PTX on Triple-Negative Breast Cancer via Regulating ADORA2A-EMT Related Signaling	China	Cross sectional	female Balb/c nu/nu mice, 6 weeks	4T1-fluc-red cells; inoculated; fourth mammary fat pad	toosendanin+ PTX	D/A*	D/A*	D/A*	D/A*	(Juan Zhang et al., 2023)
Elaidy 2023	α-Hederin Saponin Augments the Chemopreventive Effect of Cisplatin against Ehrlich Tumors and Bioinformatic Approach Identifying the Role of SDF1/CXCR4/p-AKT-1/NFκB Signaling	Egypt	Cross sectional	female Swiss Albino mice	EAT cells; s.c.; on the lower ventral side	α-Hederin+ cisplatin	D/A*	D/A*	N/A	N/A	(Elaidy et al., 2023)

References Table S4

Aribi, A., Gery, S., Lee, D. H., Thoennissen, N. H., Thoennissen, G. B., Alvarez, R., Ho, Q., Lee, K., Doan, N. B., Chan, K. T., Toh, M., Said, J. W., & Koeffler, H. P. (2013). The triterpenoid cucurbitacin B augments the antiproliferative activity of chemotherapy in human breast cancer. *International Journal of Cancer*, *132*(12), 2730–2737. https://doi.org/10.1002/ijc.27950

Cai, Y., Zheng, Y., Gu, J., Wang, S., Wang, N., Yang, B., Zhang, F., Wang, D., Fu, W., & Wang, Z. (2018). Betulinic acid chemosensitizes breast cancer by triggering ER stress-mediated apoptosis by directly targeting GRP78. *Cell Death & Disease*, *9*(6), 636. https://doi.org/10.1038/s41419-018-0669-8

Chang, Y., Fu, Q., Lu, Z., Jin, Q., Jin, T., & Zhang, M. (2024). Ginsenoside Rg3 combined with near-infrared photothermal reversal of multidrug resistance in breast cancer MCF-7/ADR cells. *Food Science & Nutrition*, *12*(8), 5750–5761. https://doi.org/10.1002/fsn3.4205

Elaidy, S. M., El-Kherbetawy, M. K., Abed, S. Y., Alattar, A., Alshaman, R., Eladl, M. A., Alamri, E. S., Al balawi, A. N., Zaid, A., Elkazzaz, A. Y., Abdelkhalig, S. M., Hamed, Z. E., & Zaitone, S. A. (2023). α-Hederin Saponin Augments the Chemopreventive Effect of Cisplatin against Ehrlich Tumors and Bioinformatic Approach Identifying the Role of SDF1/CXCR4/p-AKT-1/NFκB Signaling. *Pharmaceuticals*, *16*(3), 405. https://doi.org/10.3390/ph16030405

Hyer, M. L., Croxton, R., Krajewska, M., Krajewski, S., Kress, C. L., Lu, M., Suh, N., Sporn, M. B., Cryns, V. L., Zapata, J. M., & Reed, J. C. (2005). Synthetic Triterpenoids Cooperate with Tumor Necrosis Factor–Related Apoptosis-Inducing Ligand to Induce Apoptosis of Breast Cancer Cells. *Cancer Research*, *65*(11), 4799–4808. https://doi.org/10.1158/0008-5472.CAN-04-3319

Jin, Z.-Q., Hao, J., Yang, X., He, J.-H., Liang, J., Yuan, J.-W., Mao, Y., Liu, D., Cao, R., Wu, X.-Z., Li, X., & Chen, D. (2018). Higenamine enhances the antitumor effects of cucurbitacin B in breast cancer by inhibiting the interaction of AKT and CDK2. *Oncology Reports*. https://doi.org/10.3892/or.2018.6629

Kai, W., Yating, S., Lin, M., Kaiyong, Y., Baojin, H., Wu, Y., Fangzhou, Y., & Yan, C. (2018). Natural product toosendanin reverses the resistance of human breast cancer cells to adriamycin as a novel PI3K inhibitor. *Biochemical Pharmacology*, *152*, 153–164. https://doi.org/10.1016/j.bcp.2018.03.022

Li, C., Xue, H.-G., Feng, L.-J., Wang, M.-L., Wang, P., & Gai, X.-D. (2017). The effect of saikosaponin D on doxorubicin pharmacokinetics and its MDR reversal in MCF-7/adr cell xenografts. *European Review for Medical and Pharmacological Sciences*, *21*(19), 4437–4445.

Liby, K., Risingsong, R., Royce, D. B., Williams, C. R., Yore, M. M., Honda, T., Gribble, G. W., Lamph, W. W., Vannini, N., Sogno, I., Albini, A., & Sporn, M. B. (2008). Prevention and Treatment of Experimental Estrogen Receptor–Negative Mammary Carcinogenesis by the Synthetic Triterpenoid CDDO-Methyl Ester and the Rexinoid LG100268. *Clinical Cancer Research*, *14*(14), 4556–4563. https://doi.org/10.1158/1078-0432.CCR-08-0040

Liu, J., Cai, Q., Wang, W., Lu, M., Liu, J., Zhou, F., Sun, M., Wang, G., & Zhang, J. (2020). Ginsenoside Rh2 pretreatment and withdrawal reactivated the pentose phosphate pathway to ameliorate intracellular redox disturbance and promoted intratumoral penetration of adriamycin. *Redox Biology*, *32*, 101452. https://doi.org/10.1016/j.redox.2020.101452

Luo, B., Song, L., Chen, L., Cai, Y., Zhang, M., & Wang, S. (2024). Ganoderic acid D attenuates gemcitabine resistance of triple-negative breast cancer cells by inhibiting glycolysis via HIF-1α destabilization. *Phytomedicine*, *129*, 155675. https://doi.org/10.1016/j.phymed.2024.155675

Luo, F., Yang, J., Yang, X., Mi, J., Ye, T., Li, G., & Xie, Y. (2024). Saikosaponin D potentiates the antineoplastic effects of doxorubicin in drug-resistant breast cancer through perturbing NQO1-mediated intracellular redox balance. *Phytomedicine*, *133*, 155945. https://doi.org/10.1016/j.phymed.2024.155945

Nakhjavani, M., Smith, E., Palethorpe, H. M., Tomita, Y., Yeo, K., Price, T. J., Townsend, A. R., & Hardingham, J. E. (2021). Anti-Cancer Effects of an Optimised Combination of Ginsenoside Rg3 Epimers on Triple Negative Breast Cancer Models. *Pharmaceuticals*, *14*(7), 633. https://doi.org/10.3390/ph14070633

Shi, J., Li, J., Li, J., Li, R., Wu, X., Gao, F., Zou, L., Mak, W. W. S., Fu, C., Zhang, J., & Leung, G. P.-H. (2021). Synergistic breast cancer suppression efficacy of doxorubicin by combination with glycyrrhetinic acid as an angiogenesis inhibitor. *Phytomedicine*, *81*, 153408. https://doi.org/10.1016/j.phymed.2020.153408

So, J. Y., Wahler, J. E., Yoon, T., Smolarek, A. K., Lin, Y., Shih, W. J., Maehr, H., Uskokovic, M., Liby, K. T., Sporn, M. B., & Suh, N. (2013). Oral Administration of a Gemini Vitamin D Analog, a Synthetic Triterpenoid and the Combination Prevents Mammary Tumorigenesis Driven by ErbB2 Overexpression. *Cancer Prevention Research*, *6*(9), 959–970. https://doi.org/10.1158/1940-6207.CAPR-13-0087

Wang, K., Tu, Y., Wan, J.-B., Chen, M., & He, C. (2020). Synergistic anti-breast cancer effect of pulsatilla saponin D and camptothecin through interrupting autophagic–lysosomal function and promoting p62-mediated ubiquitinated protein aggregation. *Carcinogenesis*, *41*(6), 804–816. https://doi.org/10.1093/carcin/bgz140

Wang, L., Tang, L., Yao, C., Liu, C., & Shu, Y. (2021). The Synergistic Effects of Celastrol in combination with Tamoxifen on Apoptosis and Autophagy in MCF-7 Cells. *Journal of Immunology Research*, *2021*, 1–13. https://doi.org/10.1155/2021/5532269

Xu, A.-L., Xue, Y.-Y., Tao, W.-T., Wang, S.-Q., & Xu, H.-Q. (2022). Oleanolic acid combined with olaparib enhances radiosensitization in triple negative breast cancer and hypoxia imaging with 18F-FETNIM micro PET/CT. *Biomedicine & Pharmacotherapy*, *150*, 113007. https://doi.org/10.1016/j.biopha.2022.113007

Yuan, Z., Jiang, H., Zhu, X., Liu, X., & Li, J. (2017). Ginsenoside Rg3 promotes cytotoxicity of Paclitaxel through inhibiting NF-κB signaling and regulating Bax/Bcl-2 expression on triple-negative breast cancer. *Biomedicine & Pharmacotherapy*, *89*, 227–232. https://doi.org/10.1016/j.biopha.2017.02.038

Zhang, J., Xu, H., Wu, Y., Cho, W. C. S., Xian, Y., & Lin, Z. (2023). Synergistic Anti-Tumor Effect of Toosendanin and Paclitaxel on Triple-Negative Breast Cancer via Regulating ADORA2A-EMT Related Signaling. *Advanced Biology*, *7*(8). https://doi.org/10.1002/adbi.202300062

Zhang, J., Zhang, Z., Huang, Z., Li, M., Yang, F., Wu, Z., Guo, Q., Mei, X., Lu, B., Wang, C., Wang, Z., & Ji, L. (2023). Isotoosendanin exerts inhibition on triple-negative breast cancer through abrogating TGF-β-induced epithelial–mesenchymal transition via directly targeting TGFβR1. *Acta Pharmaceutica Sinica B*, *13*(7), 2990–3007. https://doi.org/10.1016/j.apsb.2023.05.006

Zhang, Q., Kang, X., Yang, B., Wang, J., & Yang, F. (2008). Antiangiogenic Effect of Capecitabine Combined with Ginsenoside Rg3 on Breast Cancer in Mice. *Cancer Biotherapy and Radiopharmaceuticals*, *23*(5), 647–654. https://doi.org/10.1089/cbr.2008.0532

Zhang, S., Dong, Y., Chen, X., TAN, C. S. H., Li, M., Miao, K., & Lu, J.-H. (2022). Toosendanin, a late-stage autophagy inhibitor, sensitizes triple-negative breast cancer to irinotecan chemotherapy. *Chinese Medicine*, *17*(1), 55. https://doi.org/10.1186/s13020-022-00605-8

Zhang, Y., Liu, Q.-Z., Xing, S.-P., & Zhang, J.-L. (2016). Inhibiting effect of Endostar combined with ginsenoside Rg3 on breast cancer tumor growth in tumor-bearing mice. *Asian Pacific Journal of Tropical Medicine*, *9*(2), 180–183. https://doi.org/10.1016/j.apjtm.2016.01.010

Zheng, Y., Dai, Y., Liu, W., Wang, N., Cai, Y., Wang, S., Zhang, F., Liu, P., Chen, Q., & Wang, Z. (2019). Astragaloside IV enhances taxol chemosensitivity of breast cancer via caveolin-1-targeting oxidant damage. *Journal of Cellular Physiology*, *234*(4), 4277–4290. https://doi.org/10.1002/jcp.27196

Table S5. Carriers (D/A*- data available within the source article in graphical form, N/A- data not available)

**Name of the first author and year of publication**	**Title of the article**	**Country**	**Study type**	**Animal model (sex, type, age)**	**Type of breast cancer inoculated and tumor inoculation method**	**Triterpene/ associations**	**Tumor weight (tested compound)**	**Tumor weight (control)**	**Tumor volume (tested compounds)**	**Tumor volume (control)**	**Reference**
Cun 2019	Tumor-Associated Fibroblast-Targeted Regulation and Deep Tumor Delivery of Chemotherapeutic Drugs with a Multifunctional Size-Switchable Nanoparticle	China	Cross sectional	female Balb/c mice	4T1 cells; s.c.;	GA+ gemcitabine	D/A*	D/A*	D/A*	D/A*	(Cun et al., 2019)

Zhang 2015	Improved anti-tumor activity and safety profile of a PTX-loaded glycyrrhetinic acid-graft-hyaluronic acid conjugate as a synergistically targeted drug delivery system	China	Cross sectional	female Balb/c nu/nu mice	MDA-MB-231 cells	GA+ PTX	D/A*	D/A*	D/A*	D/A*	(ZHANG et al., 2015)
Konopleva 2006	Synthetic triterpenoid 2-cyano-3,12-dioxooleana-1,9-dien-28-oic acid induces growth arrest in HER2-overexpressing breast cancer cells	USA	Cross sectional	female nude mice, 5 weeks	MCF-7/Neo or MCF7/HER2 cells; s.c.; right flank	2-cyano-3,12-dioxooleana-1,9-dien-28-oic acid (CDDO)	N/A	N/A	D/A*	D/A*	(Konopleva et al., 2006)
Dutta 2022	Synthesis, characterization, and evaluation of in vitro cytotoxicity and in vivo antitumor activity of asiatic acid-loaded poly lactic-co-glycolic acid nanoparticles: A strategy of treating breast cancer	India	Cross sectional	female Balb/c mice	4T1 cells; s.c.	AA	N/A	N/A	D/A*	D/A*	(Dutta et al., 2022)

Zhang 2024	Replacing cholesterol with asiatic acid to prolong circulation and enhance anti-metastatic effects of non-PEGylated liposomes	China	Cross sectional	female Balb/c mice	4T1 cell; into the left-bottom mammary fat pad	AA+ DOX	D/A*	D/A*	N/A	N/A	(Zhang et al., 2024)
Fu 2023	Bionic natural small molecule co-assemblies towards targeted and synergistic Chemo/PDT/CDT	China	Cross sectional	female Balb/c mice, 6-8 weeks	4T1 cells; injected; right fank	BA+ OA+ Clorine 6 (Ce6)+ Cu2	N/A	N/A	D/A*	D/A*	(Fu et al., 2023)
Li 2024	Efficient Sequential Co-Delivery Nanosystem for Inhibition of Tumor and Tumor-Associated Fibroblast-Induced Resistance and Metastasis	China	Cross sectional	female Balb/c mice, 6 weeks	4T1 cells and NIH 3T3 cells; s.c.; fourth mammary pad	BA+ Cel	D/A*	D/A*	D/A*	D/A*	(Li et al., 2024)
Wang 2018	PTX-betulinic acid hybrid nanosuspensions for enhanced anti-breast cancer activity.	China	Cross sectional	female Balb/c mice, 4 weeks	MCF-7 cells	BA+ PTX	N/A	N/A	D/A*	D/A*	(Wang et al., 2019)

Wang 2020 (1)	Impacts of particle size on the cytotoxicity, cellular internalization, pharmacokinetics and biodistribution of betulinic acid nanosuspensions in combined chemotherapy	China	Cross section al	female Balb/c mice	4T1 cells s.c. into the right mammary fat pad	BA+ taxol	N/A	N/A	D/A*	D/A*	(R. Wang et al., 2020)
Wang 2020 (2)	PTX and betulonic acid synergistically enhance antitumor efficacy by forming co-assembled nanoparticles	China	Cross section al	female Balb/c mice, 6–8 weeks	4T1 cells; s.c. into the flank of the mice	BoA+ PTX	N/A	N/A	D/A*	D/A*	(J. Wang et al., 2020)
Ling 2007	The novel triterpenoid C-28 methyl ester of 2-cyano-3, 12-dioxoolen-1, 9-dien-28-oic acid inhibits metastatic murine breast tumor growth through inactivation of STAT3 signaling.	USA	Cross section al	female Balb/c mice, 6-8 weeks	4T1/rLu/GFP cells; mammary fat pads	C-28 methyl ester of 2-cyano-3, 12-dioxoolen-1, 9-dien-28-oic acid (CDDO-Me)	N/A	N/A	N/A	N/A	(Ling et al., 2007)

Qin 2023	Therapeutic effect of multifunctional celastrol nanoparticles with mitochondrial alkaline drug release in breast cancer	China	Cross sectional	Balb/c mice	4T1 cells; hypodermicaly into the armpits	Cel	D/A*	D/A*	D/A*	D/A*	(Qin et al., 2023)
Liu 2023	Self-assembling nanoarchitectonics of size-controllable celastrol nanoparticles for efficient cancer chemotherapy with reduced systemic toxicity	China	Cross sectional	female Balb/c nu/nu mice	MCF-7 cells; s.c.; right leg	Cel	N/A	N/A	D/A*	D/A*	(Liu and Li, 2023)
Gautam 2024	Celastrol-loaded polymeric mixed micelles shows improved antitumor efficacy in 4 T1 bearing xenograft mouse model through spatial targeting	India	Cross sectional	female Balb/c mice, 4-5 weeks	4T1 cells; s.c.	Cel	D/A*	D/A*	D/A*	D/A*	(Gautam et al., 2024)
Huang 2020	Preparation of high drug-loading celastrol nanosuspensions and their anti-breast cancer activities <i>in vitro</i> and <i>in vivo</i>	China	Cross sectional	Balb/c mice , 6 weeks	4T1 cells; s.c.; right armpit	Cel	N/A	N/A	D/A*	D/A*	(Huang et al., 2020)

Huang 2024	Development of Dual-Targeted Mixed Micelles Loaded with Celastrol and Evaluation on Triple-Negative Breast Cancer Therapy	China	Cross sectional	female Balb/c mice, 4-5 weeks	MDA-MB-231 cells; transplanted; right breast pad	Cel	N/A	N/A	D/A*	D/A*	(Huang et al., 2024)
Zhao 2018	Simultaneous targeting therapy for lung metastasis and breast tumor by blocking the NF-κB signaling pathway using Celastrol-loaded micelles.	China	Cross sectional	Balb/c mice	4T1 cells; s.c. into the mammary gland	Cel	D/A*	D/A*	D/A*	D/A*	(Zhao et al., 2018)
Tan 2017	Mitochondrial alkaline pH-responsive drug release mediated by Celastrol loaded glycolipid-like micelles for cancer therapy.	India	Cross sectional	nude mice, 6-8 weeks	MCF-7 cells; s.c.	Cel	D/A*	D/A*	D/A*	D/A*	(Tan et al., 2018)

Qian 2021	Nanoparticles based on polymers modified with pH-sensitive molecular switch and low molecular weight heparin carrying Celastrol and ferrocene for breast cancer treatment	China	Cross section al	Balb/c mice	3T3/4T1 cells	Cel + ferrocene	Cela 3.24 ± 0.47 Cela+Fc 2.18 ± 0.28 PP/H NPs 1.24 ± 0.19	3.62 ± 0.45	N/A	N/A	(Qian et al., 2021)
Tian 2024	Mechanism of Self-Assembled Celastrol-Erianin Nanomedicine for treatment of breast cancer	China	Cross section al	Balb/c mice	4T1 breast cancer cells; injected in the right hind flank	Cel+ erianin	N/A	N/A	D/A*	D/A*	(Tian et al., 2024)
Soe 2018	Folate receptor-mediated celastrol and irinotecan combination delivery using liposomes for effective chemotherapy.	Korea	Cross section al	female Balb/c nu/nu mice	MDA-MB-231 cells; s.c.; right flanks	Cel+ irinotecan (Ir)	N/A	N/A	D/A*	D/A*	(Soe et al., 2018)
Zhou 2023	A celastrol-based nanodrug with reduced hepatotoxicity for primary and metastatic cancer treatment	China	Cross section al	female Balb/c mice and female nude mice	4T1 cells; into the breast pad; MDA-MB-231 cells, under the left breast fat pad of nude mice.	Cel+ Low molecular weight heparin (LMWH)+ P-selectin targeting peptide (PSN)	D/A*	D/A*	D/A*	D/A*	(M. Zhou et al., 2023)

Elhasany 2020	Combination of magnetic targeting with synergistic inhibition of NF-κB and glutathione via micellar drug nanomedicine enhances its anti-tumor efficacy	Egypt	Cross sectional	female Balb/c mice, 7-8 weeks	EAT cells; left side of the mammary fat pad	Cel+ sulfasalazine	N/A	N/A	D/A*	D/A*	(Elhasany et al., 2020)
Li 2020	Cell-penetrating corosolic acid liposome as a functional carrier for delivering chemotherapeutic drugs	China	Cross sectional	female Balb/c mice, 6-8 weeks	4T1 cells; injected; mammary fat pad	corosolic acid+ DOX	N/A	N/A	D/A*	D/A*	(Li et al., 2020)
Leng 2022	Biomimetic CuB-Polydopamine Nanoparticles for Synergistic Chemo-Photothermal Therapy of Breast Cancer	China	Cross sectional	female Balb/c mice, 6 weeks	4T1 cells; s.c.; the armpits	CuB+ laser	D/A*	D/A*	D/A*	D/A*	(Leng et al., 2022)
Ali 2022	CEG-AgNPs Ameliorates DMBA-Induced Mammary Carcinogenicity by Alleviating Cytokines Expression.	Egypt	Cross sectional	female Albino mice	DMBA; p.o.	cucurbitacin-E-glucoside	N/A	N/A	N/A	N/A	(A Ali et al., 2022)

Lu 2023	Ginsenoside Rb1 stabilized and PTX / protopanaxadiol co-loaded nanoparticles for synergistic treatment of breast tumor	China	Cross sectional	female Balb/c mice	4T1 cells subcutaneously injected into the right axilla	ginsenoside Rb1+ protopanaxadiol (20(S)-PPD)+ PTX	N/A	N/A	D/A*	D/A*	(Lu et al., 2023)
Luo 2021	Carbon nanotubes (CNT)-loaded ginsenosides Rb3 suppresses the PD-1/PD-L1 pathway in triple-negative breast cancer	China	Cross sectional	nude mice	MDA-MB-231 cells	ginsenoside Rb3	N/A	N/A	D/A*	D/A*	(Luo et al., 2021)
Zhao 2024	A pH-triggered N-oxide polyzwitterionic nano-drug loaded system for the anti-tumor immunity activation research	China	Cross sectional	female Balb/c mice	4T1 cells; i.v.; tail vein injection	ginsenoside Rg3	D/A*	D/A*	D/A*	D/A*	(Zhao et al., 2024)
Zuo 2022	Fabrication of Ginsenoside-Based Nanodrugs for Enhanced Antitumor Efficacy on Triple-Negative Breast Cancer	China	Cross sectional	female Balb/c mice, 8 weeks	4T1 cells; injected; second mammary fat pad	ginsenoside Rg3 + ginsenoside Rb1	N/A	N/A	N/A	N/A	(Zuo et al., 2022)

Xia 2022	Targeting therapy and tumor microenvironment remodeling of triple-negative breast cancer by ginsenoside Rg3 based liposomes	China	Cross sectional	female Balb/c mice, 6–8 weeks	4T1 cells; orthotopically injected; mammary fat pad in the lower right quadrant of the abdomen of the mice	ginsenoside Rg3+ docetaxel	D/A*	D/A*	D/A*	D/A*	(Xia et al., 2022)
Dong 2019	Folic acid-modified ginsenoside Rg5-loaded bovine serum albumin nanoparticles for targeted cancer therapy in vitro and in vivo.	China	Cross sectional	female Balb/c nu/nu mice, 5 weeks	MCF-7 cells; s.c.; right limb armpit	ginsenoside Rg5	D/A*	D/A*	750.85 ± 263.39 mm^3	Rg5: 1350.4 ± 269.91 mm^3, Rg5-BSA NPs: 1050.19 ± 294.54 mm^3	(Dong et al., 2019)
Long 2024	Combating multidrug resistance of breast cancer with ginsenoside Rh2-irrigated nano-in-thermogel	China	Cross sectional	female nude mice	MCF and MCF-7/PTX cells; s.c.; breast fat pad	ginsenoside Rh2 + PTX	N/A	N/A	PS1GS1-Gel: 132.53 mm^3, PS5GS1-Gel: 29.74 mm^3	no treatment: 1136.64 mm^3; PTX + GRh2: 336.81 mm^3, GS-Gel: 779.53 mm^3, PS-Gel: 182.96 mm^3	(Long et al., 2024)

Hong 2024	Ginsenoside Rh2-Based Multifunctional Liposomes for Advanced Breast Cancer Therapy.	China	Cross sectional	female Balb/c mice, 4–6 weeks	4T1 cells; i.v.; via the tail	ginsenoside Rh2+ PTX	N/A	N/A	D/A*	D/A*	(Hong et al., 2024)
Hong 2020	One Stone Four Birds: A Novel Liposomal Delivery System Multi-functionalized with Ginsenoside Rh2 for Tumor Targeting Therapy	China	Cross sectional	Balb/c mice	4T1 cells; injected; left inguinal gland	ginsenoside Rh2+ PTX	N/A	N/A	D/A*	D/A*	(Hong et al., 2020)
Wang 2024	The Remarkable Anti-Breast Cancer Efficacy and Anti-Metastasis by Multifunctional Nanoparticles Co-Loading Squamocin, R848 and IR 780	China	Cross sectional	female C57BL/6 mice	4T1 cells; s.c. into the right armpit	ginsenoside Rh2+ squamocin (Squ)+ IR728	N/A	N/A	D/A*	D/A*	(Xi Wang et al., 2024)
Mohapatra 2023	Nimbolide-based nanomedicine inhibits breast cancer stem-like cells by epigenetic reprogramming of DNMTs-SFRP1-Wnt/fβ-catenin signaling axis	India	Cross sectional	female Balb/c mice, 6-8 weeks	ALDH (high) BCSCs cells; s.c.; mammary fat pad	nimbolide	D/A*	D/A*	D/A*	D/A*	(Mohapatra et al., 2023)

Zheng 2021	A nanosensitizer self-assembled from oleanolic acid and chlorin e6 for synergistic chemo/sono-photodynamic cancer therapy	China	Cross sectional	female Balb/c mice	4T1 cells; injected into the mammary fat pad	OA+ Clorine 6 (Ce6)+ photodynamic therapy+ sonodynamic therapy	D/A*	D/A*	D/A*	D/A*	(Zheng et al., 2021)
Wang 2020 (4)	A Self-assembled Nanoparticle Platform Based on Amphiphilic Oleanolic Acid Polyprodrug for Cancer Therapy	China	Cross sectional	female Balb/c mice ,6−8 weeks	4T1 cells; i.v.; tail vein	OA +10-hydroxycamptothecin (HCPT)	0.63 ± 0.08 g	saline: 2.23 ± 0.10 g, OA NPs: 1.89 ± 0.09 g, HCPT: 1.18 ± 0.12 g	1004.6 ± 527.4 mm³	saline: 2897.2 ± 986.7 mm³, OA NPs: 2458.4 ± 1124.3 mm³, HCPT: 1852.1 ± 1022.4 mm³	(Y.-S. Wang et al., 2020)
Kong 2024	Targeted codelivery of DOX and oleanolic acid by reduction responsive hyaluronic acid-based prodrug nano-micelles for enhanced antitumor activity and reduced toxicity	China	Cross sectional	female Balb/c mice, 4 weeks	4T1 cells; s.c.; in the right flank	OA+ DOX	D/A*	D/A*	D/A*	D/A*	(Kong et al., 2024)

Niu 2019	A novel chitosan-based nanomedicine for multi-drug resistant breast cancer therapy	China	Cross sectional	female nude mice , 5-6 weeks	MDA-MB-231 cells; s.c.; right flank	OA+ DOX	N/A	N/A	13 ± 2 mm^3	saline: 746 ± 73 mm^3, OA: 446 ± 43 mm^3, DOX: 251 ± 23 mm^3	(Niu et al., 2019)
Shukla 2020	Development of putrescine anchored nano-crystalsomes bearing DOX and oleanolic acid: Deciphering their role in inhibiting metastatic breast cancer	India	Cross sectional	female Balb/c mice	4T1 cells	OA+ DOX	D/A*	D/A*	D/A*	D/A*	(Shukla et al., 2021)
Bao 2020	Synergistic Chemotherapy for Breast Cancer and Breast Cancer Brain Metastases via PTX-Loaded Oleanolic Acid Nanoparticles.	USA	Cross sectional	female NCr-nu/nu mice, 5-6 weeks	231WT/Luc cells and 231 Br cells; s.c.; both of the left and right flanks	OA+ PTX	N/A	N/A	D/A*	D/A*	(Bao et al., 2020)

Chen 2024	Oleanolic acid derivative self-assembled aggregates based on heparin and chitosan for breast cancer therapy.	China	Cross sectional	female Balb/c mice, 6-8 weeks	4T1 cells; implanted; second breast fat pad in the right lateral	oleanolic quaternary ammonium derivative (QDT)	QDT-HEP/CS NPs 10 mg/kg: 0.66 ± 0,09 g, QDT-HEP/CS NPs 20 mg/kg: 0.57 ± 0.02 g, QDT-HEP/CS NPs 40 mg/kg: 0.47 ± 0.02 g	PBS: 1.04 ± 0.19 g, QDT 10 mg: 0.84 ± 0.18 g	D/A*	D/A*	(Chen et al., 2024)
Wu 2022	Ginsenoside Rg3 nanoparticles with permeation enhancing based chitosan derivatives were encapsulated with DOX by thermosensitive hydrogel and anti-cancer evaluation of peritumoral hydrogel injection combined with PD-L1 antibody	China	Cross sectional	female Balb/c mice, 6–8 weeks	4T1 cells; injected; right mammary fat pad	ginsenoside Rg3+ DOX+ PD-L1 antibody	D/A*	D/A*	D/A*	D/A*	(Wu et al., 2022)

Zhu 2023	PTX-loaded ginsenoside Rg3 liposomes for drug-resistant cancer therapy by dual targeting of the tumor microenvironment and cancer cells.	China	Cross sectional	Balb/c mice	MCF-7/T; s.c.; into the flanks	ginsenoside Rg3+ PTX	D/A*	D/A*	D/A*	D/A*	(Zhu et al., 2023)
Sun 2020	Saikosaponin D loaded macrophage membrane-biomimetic nanoparticles target angiogenic signaling for breast cancer therapy	China	Cross sectional	female Balb/c mice, 4-6 weeks	4T1 cells; mammary fat pad	SsD	1 mg/kg: 301 ± 100 mg, 5 mg/kg: 102 ± 66 mg	783 ± 139 mg	1 mg/kg: of 159 ± 92 mm³; 5 mg/kg: 91 ± 7 mm³	660 ± 40 mm³	(Sun et al., 2020)
Rocha 2016	Evaluation of Antitumor Activity of Long-Circulating and pH-Sensitive Liposomes Containing Ursolic Acid in Animal Models of Breast Tumor and Gliosarcoma	Brazil	Cross sectional	female nude mice, 8-9 weeks	MCF-7, s.c.; dorsal flank	UA	N/A	N/A	N/A	N/A	(Rocha et al., 2016)

Jin 2016	Folate-Chitosan Nanoparticles Loaded with Ursolic Acid Confer Anti-Breast Cancer Activities in vitro and in vivo.	USA	Cross sectional	Balb/c mice, 4-6 weeks	MCF-7 cells; s.c.; into the back	UA	D/A*	D/A*	N/A	N/A	(Jin et al., 2016)
Zhang 2020	Solubilization and delivery of Ursolic-acid for modulating tumor microenvironment and regulatory T cell activities in cancer immunotherapy	China	Cross sectional	female Balb/c mice	4T1 cells; s.c. into the right flank.	UA	N/A	N/A	D/A*	D/A*	(Zhang et al., 2020)
Jiang 2017 (2)	A carrier-free dual-drug nanodelivery system functionalized with aptamer specific targeting HER2-overexpressing cancer cells	China	Cross sectional	female nude mice	BT474 cells; s.c.; right armpit	UA+ DOX	D/A*	D/A*	D/A*	D/A*	(Kai Jiang et al., 2017)
Liu 2017	Self-assembled nanoparticles based on a carboxymethylcellulose-ursolic acid conjugate for anticancer combination therapy	China	Cross sectional	female Balb/c mice, 4–6 weeks	4T1 cells; s.c.; right auxiliary flank region	UA+ hydroxycamptothecin	N/A	N/A	362 ± 129 mm³	PBS: 5059 ± 1526 mm³, UA: 2834 ± 1074 mm³, UA NPs: 538	(Liu et al., 2017)

										± 167 mm³	
Lan 2021	A carrier-free folate receptor-targeted ursolic acid/methotrexate nanodelivery system for synergetic anticancer therapy	China	Cross sectional	Balb/c mice, 4-5 weeks	MCF-7cells; s.c.; right front leg	UA+ methotrexate	D/A*	D/A*	D/A*	D/A*	(Lan et al., 2021)
Sharma 2024	Unveiling the potential of ursolic acid modified hyaluronate nanoparticles for combination drug therapy in triple negative breast cancer	India	Cross sectional	female Balb/c mice	4T1 cells; s.c. into the 4th mamary gland	UA+ PTX	D/A*	D/A*	D/A*	D/A*	(Sharma et al., 2024)

References Table S5

A Ali, A., A Hussein, M., A Emara, A., Abd Elrahman, A. M., A Hassan, A., A Abdelghaney, A., M Bastawey, A., M Maher, A., N Al-Wadayi, A.-M., A Shalaby, M., M Mohamed, M., Gamal El Din, M. A., A Muhammad, S., S Ewees, A., Nasr-Eldin, M. S., A Alshrief, D., H Mohamed, A., Mostafa, H., El-Ella, A. A., … A Hussein, M. (2022). CEG-AgNPs Ameliorates DMBA-Induced Mammary Carcinogenicity by Alleviating Cytokines Expression. *Pakistan Journal of Biological Sciences : PJBS*, *25*(6), 485–494. https://doi.org/10.3923/pjbs.2022.485.494

Bao, Y., Zhang, S., Chen, Z., Chen, A. T., Ma, J., Deng, G., Xu, W., Zhou, J., Yu, Z.-Q., Yao, G., & Chen, J. (2020). Synergistic Chemotherapy for Breast Cancer and Breast Cancer Brain Metastases via Paclitaxel-Loaded Oleanolic Acid Nanoparticles. *Molecular Pharmaceutics*, *17*(4), 1343–1351. https://doi.org/10.1021/acs.molpharmaceut.0c00044

Chen, K., Zhu, X., Sun, R., Zhao, L., Zhao, J., Wu, X., Wang, C., & Zeng, H. (2024). Oleanolic acid derivative self-assembled aggregates based on heparin and chitosan for breast cancer therapy. *International Journal of Biological Macromolecules*, *277*, 134431. https://doi.org/10.1016/j.ijbiomac.2024.134431

Cun, X., Chen, J., Li, M., He, X., Tang, X., Guo, R., Deng, M., Li, M., Zhang, Z., & He, Q. (2019). Tumor-Associated Fibroblast-Targeted Regulation and Deep Tumor Delivery of Chemotherapeutic Drugs with a Multifunctional Size-Switchable Nanoparticle. *ACS Applied Materials & Interfaces*, *11*(43), 39545–39559. https://doi.org/10.1021/acsami.9b13957

Dong, Y., Fu, R., Yang, J., Ma, P., Liang, L., Mi, Y., & Fan, D. (2019). <p>Folic acid-modified ginsenoside Rg5-loaded bovine serum albumin nanoparticles for targeted cancer therapy in vitro and in vivo</p>. *International Journal of Nanomedicine*, *Volume 14*, 6971–6988. https://doi.org/10.2147/IJN.S210882

Dutta, S., Chakraborty, P., Basak, S., Ghosh, S., Ghosh, N., Chatterjee, S., Dewanjee, S., & Sil, P. C. (2022). Synthesis, characterization, and evaluation of in vitro cytotoxicity and in vivo antitumor activity of asiatic acid-loaded poly lactic-co-glycolic acid nanoparticles: A strategy of treating breast cancer. *Life Sciences*, *307*, 120876. https://doi.org/10.1016/j.lfs.2022.120876

Elhasany, K. A., Khattab, S. N., Bekhit, A. A., Ragab, D. M., Abdulkader, M. A., Zaky, A., Helmy, M. W., Ashour, H. M. A., Teleb, M., Haiba, N. S., & Elzoghby, A. O. (2020). Combination of magnetic targeting with synergistic inhibition of NF-κB and glutathione via micellar drug nanomedicine enhances its anti-tumor efficacy. *European Journal of Pharmaceutics and Biopharmaceutics*, *155*, 162–176. https://doi.org/10.1016/j.ejpb.2020.08.004

Fu, S., Wang, M., Li, B., Li, X., Cheng, J., Zhao, H., Zhang, H., Dong, A., Lu, W., & Yang, X. (2023). Bionic natural small molecule co-assemblies towards targeted and synergistic Chemo/PDT/CDT. *Biomaterials Research*, *27*(1). https://doi.org/10.1186/s40824-023-00380-z

Gautam, S., Singh, N., Marwaha, D., Rai, N., Sharma, M., Tiwari, P., Singh, S., Kumar Bakshi, A., Kumar, A., Agarwal, N., Prakash Shukla, R., & Ranjan Mishra, P. (2024). Celastrol-loaded polymeric mixed micelles shows improved antitumor efficacy in 4 T1 bearing xenograft mouse model through spatial targeting. *International Journal of Pharmaceutics*, *659*, 124234. https://doi.org/10.1016/j.ijpharm.2024.124234

Hong, C., Liang, J., Xia, J., Zhu, Y., Guo, Y., Wang, A., Lu, C., Ren, H., Chen, C., Li, S., Wang, D., Zhan, H., & Wang, J. (2020). One Stone Four Birds: A Novel Liposomal Delivery System Multi-functionalized with Ginsenoside Rh2 for Tumor Targeting Therapy. *Nano-Micro Letters*, *12*(1), 129. https://doi.org/10.1007/s40820-020-00472-8

Hong, C., Wang, A., Xia, J., Liang, J., Zhu, Y., Wang, D., Zhan, H., Feng, C., Jiang, X., Pan, J., & Wang, J. (2024). Ginsenoside Rh2-Based Multifunctional Liposomes for Advanced Breast Cancer Therapy. *International Journal of Nanomedicine*, *Volume 19*, 2879–2888. https://doi.org/10.2147/IJN.S437733

Huang, S., Xiao, S., Li, X., Tao, R., Yang, Z., Gao, Z., Hu, J., Meng, Y., Zheng, G., & Chen, X. (2024). Development of Dual-Targeted Mixed Micelles Loaded with Celastrol and Evaluation on Triple-Negative Breast Cancer Therapy. *Pharmaceutics*, *16*(9), 1174. https://doi.org/10.3390/pharmaceutics16091174

Huang, T., Wang, Y., Shen, Y., Ao, H., Guo, Y., Han, M., & Wang, X. (2020). Preparation of high drug-loading celastrol nanosuspensions and their anti-breast cancer activities in vitro and in vivo. *Scientific Reports*, *10*(1), 8851. https://doi.org/10.1038/s41598-020-65773-9

Jiang, K., Han, L., Guo, Y., Zheng, G., Fan, L., Shen, Z., Zhao, R., & Shao, J. (2017). A carrier-free dual-drug nanodelivery system functionalized with aptamer specific targeting HER2-overexpressing cancer cells. *Journal of Materials Chemistry B*, *5*(46), 9121–9129. https://doi.org/10.1039/C7TB02562A

Jin, H., Pi, J., Yang, F., Jiang, J., Wang, X., Bai, H., Shao, M., Huang, L., Zhu, H., Yang, P., Li, L., Li, T., Cai, J., & Chen, Z. W. (2016). Folate-Chitosan Nanoparticles Loaded with Ursolic Acid Confer Anti-Breast Cancer Activities in vitro and in vivo. *Scientific Reports*, *6*(1), 30782. https://doi.org/10.1038/srep30782

Kong, F., Liu, H., Zhao, C., & Qin, J. (2024). Targeted codelivery of doxorubicin and oleanolic acid by reduction responsive hyaluronic acid-based prodrug nano-micelles for enhanced antitumor activity and reduced toxicity. *International Journal of Biological Macromolecules*, *277*, 134135. https://doi.org/10.1016/j.ijbiomac.2024.134135

Konopleva, M., Zhang, W., Shi, Y.-X., McQueen, T., Tsao, T., Abdelrahim, M., Munsell, M. F., Johansen, M., Yu, D., Madden, T., Safe, S. H., Hung, M.-C., & Andreeff, M. (2006). Synthetic triterpenoid 2-cyano-3,12-dioxooleana-1,9-dien-28-oic acid induces growth arrest in HER2-overexpressing breast cancer cells. *Molecular Cancer Therapeutics*, *5*(2), 317–328. https://doi.org/10.1158/1535-7163.MCT-05-0350

Lan, J.-S., Qin, Y.-H., Liu, L., Zeng, R.-F., Yang, Y., Wang, K., Ding, Y., Zhang, T., & Ho, R. J. (2021). A Carrier-Free Folate Receptor-Targeted Ursolic Acid/Methotrexate Nanodelivery System for Synergetic Anticancer Therapy. *International Journal of Nanomedicine*, *Volume 16*, 1775–1787. https://doi.org/10.2147/IJN.S287806

Leng, J., Dai, X., Cheng, X., Zhou, H., Wang, D., Zhao, J., Ma, K., Cui, C., Wang, L., & Guo, Z. (2022). Biomimetic Cucurbitacin B-Polydopamine Nanoparticles for Synergistic Chemo-Photothermal Therapy of Breast Cancer. *Frontiers in Bioengineering and Biotechnology*, *10*. https://doi.org/10.3389/fbioe.2022.841186

Li, C., Wang, Z., Zhang, Y., Zhu, Y., Xu, M., Lei, H., & Zhang, D. (2024). Efficient Sequential Co-Delivery Nanosystem for Inhibition of Tumor and Tumor-Associated Fibroblast-Induced Resistance and Metastasis. *International Journal of Nanomedicine*, *Volume 19*, 1749–1766. https://doi.org/10.2147/IJN.S427783

Li, X., Widjaya, A. S., Liu, J., Liu, X., Long, Z., & Jiang, Y. (2020). Cell-penetrating corosolic acid liposome as a functional carrier for delivering chemotherapeutic drugs. *Acta Biomaterialia*, *106*, 301–313. https://doi.org/10.1016/j.actbio.2020.02.013

Ling, X., Konopleva, M., Zeng, Z., Ruvolo, V., Stephens, L. C., Schober, W., McQueen, T., Dietrich, M., Madden, T. L., & Andreeff, M. (2007). The Novel Triterpenoid C-28 Methyl Ester of 2-Cyano-3, 12-Dioxoolen-1, 9-Dien-28-Oic Acid Inhibits Metastatic Murine Breast Tumor Growth through Inactivation of STAT3 Signaling. *Cancer Research*, *67*(9), 4210–4218. https://doi.org/10.1158/0008-5472.CAN-06-3629

Liu, Y., & Li, J. (2023). Self-assembling nanoarchitectonics of size-controllable celastrol nanoparticles for efficient cancer chemotherapy with reduced systemic toxicity. *Journal of Colloid and Interface Science*, *636*, 216–222. https://doi.org/10.1016/j.jcis.2022.12.162

Liu, Y., Liu, K., Li, C., Wang, L., Liu, J., He, J., Lei, J., & Liu, X. (2017). Self-assembled nanoparticles based on a carboxymethylcellulose–ursolic acid conjugate for anticancer combination therapy. *RSC Advances*, *7*(58), 36256–36268. https://doi.org/10.1039/C7RA05913B

Long, J., Hu, W., Ren, T., Wang, X., Lu, C., Pan, X., Wu, C., & Peng, T. (2024). Combating multidrug resistance of breast cancer with ginsenoside Rh2-irrigated nano-in-thermogel. *International Journal of Pharmaceutics*, *650*, 123718. https://doi.org/10.1016/j.ijpharm.2023.123718

Lu, L., Ao, H., Fu, J., Li, M., Guo, Y., Guo, Y., Han, M., Shi, R., & Wang, X. (2023). Ginsenoside Rb1 stabilized and paclitaxel / protopanaxadiol co-loaded nanoparticles for synergistic treatment of breast tumor. *Biomedicine & Pharmacotherapy*, *163*, 114870. https://doi.org/10.1016/j.biopha.2023.114870

Luo, X., Wang, H., & Ji, D. (2021). Carbon nanotubes (CNT)-loaded ginsenosides Rb3 suppresses the PD-1/PD-L1 pathway in triple-negative breast cancer. *Aging*, *13*(13), 17177–17189. https://doi.org/10.18632/aging.203131

Mohapatra, P., Madhulika, S., Behera, S., Singh, P., Sa, P., Prasad, P., Swain, R. K., & Sahoo, S. K. (2023). Nimbolide-based nanomedicine inhibits breast cancer stem-like cells by epigenetic reprogramming of DNMTs-SFRP1-Wnt/β-catenin signaling axis. *Molecular Therapy Nucleic Acids*, *34*, 102031. https://doi.org/10.1016/j.omtn.2023.102031

Niu, S., Williams, G. R., Wu, J., Wu, J., Zhang, X., Zheng, H., Li, S., & Zhu, L.-M. (2019). A novel chitosan-based nanomedicine for multi-drug resistant breast cancer therapy. *Chemical Engineering Journal*, *369*, 134–149. https://doi.org/10.1016/j.cej.2019.02.201

Qian, Y., Zhang, J., Xu, R., Li, Q., Shen, Q., & Zhu, G. (2021). Nanoparticles based on polymers modified with pH-sensitive molecular switch and low molecular weight heparin carrying Celastrol and ferrocene for breast cancer treatment. *International Journal of Biological Macromolecules*, *183*, 2215–2226. https://doi.org/10.1016/j.ijbiomac.2021.05.204

Qin, Y., Wang, Z., Wang, X., Zhang, T., Hu, Y., Wang, D., Sun, H., Zhang, L., & Zhu, Y. (2023). Therapeutic effect of multifunctional celastrol nanoparticles with mitochondrial alkaline drug release in breast cancer. *Materials Today Advances*, *17*, 100328. https://doi.org/10.1016/j.mtadv.2022.100328

Rocha, T. G. R., Lopes, S. C. de A., Cassali, G. D., Ferreira, Ê., Veloso, E. S., Leite, E. A., Braga, F. C., Ferreira, L. A. M., Balvay, D., Garofalakis, A., Oliveira, M. C., & Tavitian, B. (2016). Evaluation of Antitumor Activity of Long-Circulating and pH-Sensitive Liposomes Containing Ursolic Acid in Animal Models of Breast Tumor and Gliosarcoma. *Integrative Cancer Therapies*, *15*(4), 512–524. https://doi.org/10.1177/1534735416628273

Sharma, R., Yadav, V., Jha, S., Dighe, S., & Jain, S. (2024). Unveiling the potential of ursolic acid modified hyaluronate nanoparticles for combination drug therapy in triple negative breast cancer. *Carbohydrate Polymers*, *338*, 122196. https://doi.org/10.1016/j.carbpol.2024.122196

Shukla, R. P., Urandur, S., Banala, V. T., Marwaha, D., Gautam, S., Rai, N., Singh, N., Tiwari, P., Shukla, P., & Mishra, P. R. (2021). Development of putrescine anchored nano-crystalsomes bearing doxorubicin and oleanolic acid: deciphering their role in inhibiting metastatic breast cancer. *Biomaterials Science*, *9*(5), 1779–1794. https://doi.org/10.1039/D0BM01033B

Soe, Z. C., Thapa, R. K., Ou, W., Gautam, M., Nguyen, H. T., Jin, S. G., Ku, S. K., Oh, K. T., Choi, H.-G., Yong, C. S., & Kim, J. O. (2018). Folate receptor-mediated celastrol and irinotecan combination delivery using liposomes for effective chemotherapy. *Colloids and Surfaces B: Biointerfaces*, *170*, 718–728. https://doi.org/10.1016/j.colsurfb.2018.07.013

Sun, K., Yu, W., Ji, B., Chen, C., Yang, H., Du, Y., Song, M., Cai, H., Yan, F., & Su, R. (2020). Saikosaponin D loaded macrophage membrane-biomimetic nanoparticles target angiogenic signaling for breast cancer therapy. *Applied Materials Today*, *18*, 100505. https://doi.org/10.1016/j.apmt.2019.100505

Tan, Y., Zhu, Y., Zhao, Y., Wen, L., Meng, T., Liu, X., Yang, X., Dai, S., Yuan, H., & Hu, F. (2018). Mitochondrial alkaline pH-responsive drug release mediated by Celastrol loaded glycolipid-like micelles for cancer therapy. *Biomaterials*, *154*, 169–181. https://doi.org/10.1016/j.biomaterials.2017.07.036

Tian, J., Chen, K., Zhang, Q., Qiu, C., Tong, H., Huang, J., Hao, M., Chen, J., Zhao, W., Wong, Y.-K., Gao, L., Luo, P., Wang, J., & Du, Q. (2024). Mechanism of Self-Assembled Celastrol-Erianin Nanomedicine for treatment of breast cancer. *Chemical Engineering Journal*, *499*, 155709. https://doi.org/10.1016/j.cej.2024.155709

Wang, J., Qiao, W., Zhao, H., & Yang, X. (2020). Paclitaxel and betulonic acid synergistically enhance antitumor efficacy by forming co-assembled nanoparticles. *Biochemical Pharmacology*, *182*, 114232. https://doi.org/10.1016/j.bcp.2020.114232

Wang, R., Wang, X., Jia, X., Wang, H., Li, W., & Li, J. (2020). Impacts of particle size on the cytotoxicity, cellular internalization, pharmacokinetics and biodistribution of betulinic acid nanosuspensions in combined chemotherapy. *International Journal of Pharmaceutics*, *588*, 119799. https://doi.org/10.1016/j.ijpharm.2020.119799

Wang, R., Yang, M., Li, G., Wang, X., Zhang, Z., Qiao, H., Chen, J., Chen, Z., Cui, X., & Li, J. (2019). Paclitaxel-betulinic acid hybrid nanosuspensions for enhanced anti-breast cancer activity. *Colloids and Surfaces B: Biointerfaces*, *174*, 270–279. https://doi.org/10.1016/j.colsurfb.2018.11.029

Wang, X., Liu, X., Guo, Y., Gong, T., Lu, W., Han, M., Guo, Y., & Wang, X. (2024). The Remarkable Anti-Breast Cancer Efficacy and Anti-Metastasis by Multifunctional Nanoparticles Co-Loading Squamocin, R848 and IR 780. *International Journal of Nanomedicine*, *Volume 19*, 4679–4699. https://doi.org/10.2147/IJN.S448860

Wang, Y.-S., Li, G.-L., Zhu, S.-B., Jing, F.-C., Liu, R.-D., Li, S.-S., He, J., & Lei, J.-D. (2020). A Self-assembled Nanoparticle Platform Based on Amphiphilic Oleanolic Acid Polyprodrug for Cancer Therapy. *Chinese Journal of Polymer Science*, *38*(8), 819–829. https://doi.org/10.1007/s10118-020-2401-2

Wu, H., Wei, G., Luo, L., Li, L., Gao, Y., Tan, X., Wang, S., Chang, H., Liu, Y., Wei, Y., Song, J., Zhang, Z., & Huo, J. (2022). Ginsenoside Rg3 nanoparticles with permeation enhancing based chitosan derivatives were encapsulated with doxorubicin by thermosensitive hydrogel and anti-cancer evaluation of peritumoral hydrogel injection combined with PD-L1 antibody. *Biomaterials Research*, *26*(1). https://doi.org/10.1186/s40824-022-00329-8

Xia, J., Zhang, S., Zhang, R., Wang, A., Zhu, Y., Dong, M., Ma, S., Hong, C., Liu, S., Wang, D., & Wang, J. (2022). Targeting therapy and tumor microenvironment remodeling of triple-negative breast cancer by ginsenoside Rg3 based liposomes. *Journal of Nanobiotechnology*, *20*(1), 414. https://doi.org/10.1186/s12951-022-01623-2

ZHANG, L., ZHOU, J.-P., & YAO, J. (2015). Improved anti-tumor activity and safety profile of a paclitaxel-loaded glycyrrhetinic acid-graft-hyaluronic acid conjugate as a synergistically targeted drug delivery system. *Chinese Journal of Natural Medicines*, *13*(12), 915–924. https://doi.org/10.1016/S1875-5364(15)30097-2

Zhang, N., Liu, S., Shi, S., Chen, Y., Xu, F., Wei, X., & Xu, Y. (2020). Solubilization and delivery of Ursolic-acid for modulating tumor microenvironment and regulatory T cell activities in cancer immunotherapy. *Journal of Controlled Release*, *320*, 168–178. https://doi.org/10.1016/j.jconrel.2020.01.015

Zhang, Y., Wang, Y., Zhang, H., Huang, S., Li, Y., Long, J., Han, Y., Lin, Q., Gong, T., Sun, X., Zhang, Z., & Zhang, L. (2024). Replacing cholesterol with asiatic acid to prolong circulation and enhance anti-metastatic effects of non-PEGylated liposomes. *Journal of Controlled Release*, *366*, 585–595. https://doi.org/10.1016/j.jconrel.2024.01.009

Zhao, Y., Bai, Y., Li, M., Nie, X., Meng, H., Shosei, S., Liu, L., Yang, Q., Shen, M., & Li, Y. (2024). A pH-triggered N-oxide polyzwitterionic nano-drug loaded system for the anti-tumor immunity activation research. *Journal of Nanobiotechnology*, *22*(1), 420. https://doi.org/10.1186/s12951-024-02677-0

Zhao, Y., Tan, Y., Meng, T., Liu, X., Zhu, Y., Hong, Y., Yang, X., Yuan, H., Huang, X., & Hu, F. (2018). Simultaneous targeting therapy for lung metastasis and breast tumor by blocking the NF-κB signaling pathway using Celastrol-loaded micelles. *Drug Delivery*, *25*(1), 341–352. https://doi.org/10.1080/10717544.2018.1425778

Zheng, Y., Li, Z., Yang, Y., Shi, H., Chen, H., & Gao, Y. (2021). A nanosensitizer self-assembled from oleanolic acid and chlorin e6 for synergistic chemo/sono-photodynamic cancer therapy. *Phytomedicine*, *93*, 153788. https://doi.org/10.1016/j.phymed.2021.153788

Zhou, M., Liao, J., Lai, W., Xu, R., Liu, W., Xie, D., Wang, F., Zhang, Z., Huang, J., Zhang, R., & Li, G. (2023). A celastrol-based nanodrug with reduced hepatotoxicity for primary and metastatic cancer treatment. *EBioMedicine*, *94*, 104724. https://doi.org/10.1016/j.ebiom.2023.104724

Zhu, Y., Wang, A., Zhang, S., Kim, J., Xia, J., Zhang, F., Wang, D., Wang, Q., & Wang, J. (2023). Paclitaxel-loaded ginsenoside Rg3 liposomes for drug-resistant cancer therapy by dual targeting of the tumor microenvironment and cancer cells. *Journal of Advanced Research*, *49*, 159–173. https://doi.org/10.1016/j.jare.2022.09.007

Zuo, S., Wang, J., An, X., Wang, Z., Zheng, X., & Zhang, Y. (2022). Fabrication of Ginsenoside-Based Nanodrugs for Enhanced Antitumor Efficacy on Triple-Negative Breast Cancer. *Frontiers in Bioengineering and Biotechnology*, *10*. https://doi.org/10.3389/fbioe.2022.945472

Table S6. Risk of bias assessment according to SYRCLE (after discrepancies were resolved)

Type of bias	Selection bias			Performance bias		Detection bias		Attrition bias	Reporting bias	Other
First author's name and year of publication	Sequence generation	Baseline characteristics	Allocation concealment	Random housing	Blinding of participants and personnel	Random outcome assessment	Blinding of outcome assessment	Incomplete outcome data	Selective outcome reporting	Report of ethical approval for the animal study
Abdelmoneem 2021	Unclear	Low	High	Low	High	Low	High	Low	Low	Low
Akl 2014	Unclear	Low	High	Low	High	Low	High	Low	Low	Low
Al Marzouqi 2011	Unclear	Low	High	Low	High	Unclear	High	Unclear	Low	Low
Ali 2022	Unclear	Unclear	High	Low	High	Unclear	High	Unclear	Low	Low
Aribi 2012	Unclear	Low	High	Low	High	Unclear	High	Low	Low	Low
Arif 2024	Unclear	Unclear	High	Low	High	Unclear	High	Unclear	Low	Low
Ball 2020	Unclear	Low	High	Unclear	High	Unclear	High	Low	Low	Low
Bao 2020	Unclear	Low	High	Unclear	High	Low	High	Low	Low	Low
Bishayee 2013	Unclear	Low	High	Low	High	Low	High	Low	Low	Low
Blaskovich 2003	Unclear	Low	High	Unclear	High	Low	High	Unclear	Low	Low
Cai 2018	Unclear	Low	High	Unclear	High	Low	High	Unclear	Low	Low
Cevatemre 2017	Unclear	Low	High	Unclear	High	Low	High	Unclear	Low	Low
Chang 2024	Unclear	Low	High	Unclear	High	Low	High	Unclear	Low	Low
Chen 2024	Unclear	Low	High	Unclear	High	High	High	Low	Low	Low
Cheng 2023	Unclear	Low	High	Unclear	High	Low	High	Low	Low	Low
Choi 2011	Unclear	Low	High	Unclear	High	Low	High	Unclear	High	Low
Chu 2020	Unclear	Low	High	Unclear	High	High	High	Low	Low	High
Chun 2013	Unclear	Low	High	Unclear	High	Low	High	Low	Low	Low
Cun 2019	Unclear	Unclear	High	Unclear	High	Low	High	Low	Low	High
Damle 2013	Unclear	Unclear	High	Low	High	High	High	Low	Low	Low

Dasgupta 2016	Unclear	Low	High	Unclear	Unclear	High	High	Unclear	Low	Low
De Angel 2010	Unclear	Low	High	Low	High	Low	High	Low	Low	Low
Dong 2019	Unclear	Low	High	Unclear	High	Low	High	Low	Low	Low
Duan 2018	Unclear	Unclear	High	Low	High	Low	High	Low	Low	Low
Dutta 2022	Unclear	Unclear	High	Unclear	High	High	High	Unclear	Low	Low
Elaidy 2023	Unclear	Low	High	Low	High	Low	High	Low	Low	Low
Elhasany 2020	Unclear	Low	High	Low	High	Low	High	Low	Low	Low
Farhangfar 2022	High	Low	High	Low	High	High	High	Low	Low	Low
Feng 2020	Unclear	Low	High	Unclear	High	High	High	High	Low	Low
Fu 2023	Unclear	Low	High	Unclear	High	Low	High	Low	Low	Low
Gan 2024	Unclear	Low	High	Unclear	High	Low	High	Low	Low	High
Gao 2016	Unclear	Low	High	Unclear	High	High	High	Low	Low	Low
Gautam 2024	Unclear	Low	High	Unclear	High	Low	High	Low	Low	Low
Gou 2020	High	Low	High	Unclear	High	High	High	Unclear	Low	High
Gupta 2014	Unclear	Low	High	Unclear	High	Low	High	Low	Low	Low
He 2024	Low	Low	High	Unclear	High	Low	High	Unclear	Low	Low
Hong 2019 (1)	Unclear	Unclear	High	Unclear	High	Low	High	Low	Low	Low
Hong 2019(2)	Unclear	Unclear	High	Unclear	High	Low	High	Low	Low	Low
Hong 2020	Unclear	High	High	Unclear	High	Low	High	Low	Low	Low
Hong 2024	Unclear	Low	High	Unclear	High	Low	High	Low	Low	Low
Hu 2020	Unclear	Low	High	Unclear	High	High	High	Low	Low	Low
Hu 2020 (1)	Unclear	Low	High	Unclear	High	Low	High	Low	Low	Low
Huang 2020	Unclear	Unclear	High	Unclear	High	Low	High	Low	Low	Low
Huang 2021	Unclear	Low	High	Unclear	High	High	High	Low	Low	High
Huang 2024	Unclear	Low	High	Unclear	High	Low	High	Low	Low	Low
Huynh 2021	Unclear	Low	High	Unclear	High	Low	High	Low	Low	Low
Hyer 2005	Unclear	Low	High	Unclear	High	High	High	Low	High	High

Jeon 2021	Unclear	Low	High	Unclear	High	Low	High	Low	Low	Low
Jiang 2017 (1)	Unclear	Low	High	Unclear	High	High	High	Low	Low	Low
Jiang 2017 (2)	Unclear	Unclear	High	Unclear	High	Low	High	Unclear	Low	Low
Jiao 2019	Unclear	Low	High	Unclear	High	Low	High	Low	Low	Low
Jin 2016	Unclear	Unclear	High	Unclear	High	Low	High	Low	Low	Low
Jin 2018	Unclear	Low	High	Unclear	High	Low	High	Low	Low	Low
Kai 2018	Unclear	Unclear	High	Unclear	High	Low	High	Low	Low	Low
Kallepu 2020	Unclear	High	High	Unclear	High	Low	High	Low	Low	Low
Kim 2011	Unclear	Low	High	Unclear	High	High	High	Unclear	Low	High
Kong 2016	Unclear	Low	High	Unclear	High	Low	High	Low	Low	Low
Kong 2024	Unclear	Unclear	High	Unclear	High	Low	High	Low	Low	High
Konopleva 2006	Unclear	Low	High	Unclear	High	High	High	Unclear	Low	High
Lan 2021	Unclear	Unclear	High	Unclear	High	Low	High	Low	Low	Low
Lee 2018	Unclear	Low	High	Low	High	Low	High	Low	Low	Low
Leng 2022	Unclear	Low	High	Unclear	High	Low	High	Unclear	Low	Low
Li 2017	Unclear	Unclear	High	Unclear	High	Low	High	Low	Low	Low
Li 2018	Unclear	Low	High	Unclear	High	Low	High	Unclear	Low	Low
Li 2020	Unclear	Low	High	Unclear	High	Low	High	Low	Low	High
Li 2021	Unclear	Low	High	Unclear	High	Low	High	Low	Low	Low
Li 2024	Unclear	Low	High	Low	High	High	High	Low	Low	Low
Liang 2018	Unclear	Low	High	Unclear	High	Low	High	Low	Low	High
Liang 2021	Unclear	Low	High	Unclear	High	Low	High	Low	Low	High
Liby 2008	Unclear	High	High	Unclear	High	High	High	Unclear	Low	Low
Lim 2020	Unclear	High	High	Unclear	High	High	High	Low	Low	Low
Ling 2007	Unclear	Low	High	Unclear	High	High	High	Unclear	Low	High
Liu 2017	Unclear	Low	High	Unclear	High	Low	High	Unclear	Low	Low
Liu 2018 (1)	Unclear	Low	High	Unclear	High	Low	High	Low	Low	Low

Liu 2018 (2)	Unclear	Low	High	Unclear	High	Low	High	Low	Low	Low
Liu 2020	Unclear	Low	High	Low	High	Low	High	Low	Low	Low
Liu 2023	Unclear	Low	High	Unclear	High	Low	High	Low	Low	Low
Long 2024	Unclear	Low	High	Unclear	High	Low	High	Low	Low	Low
Lu 2023	Unclear	Unclear	High	Unclear	High	Low	High	Low	Low	Low
Lubet 2016	Low	Low	High	Unclear	High	High	High	Low	Low	High
Luo 2021	High	High	High	High	High	High	High	High	High	High
Luo 2024 (1)	Unclear	Low	High	Unclear	High	Low	High	Low	Low	Low
Luo 2024 (2)	Unclear	High	High	Unclear	High	High	High	Low	Low	Low
Mandal 2013 (1)	Unclear	Low	High	Unclear	High	Low	High	Low	Low	Low
Mandal 2013 (2)	Unclear	Low	High	Unclear	High	Low	High	Low	Low	Low
Mertens-Talcott 2012	Unclear	Low	High	Unclear	High	High	High	Low	Low	Low
Mohapatra 2023	Unclear	Low	High	Low	High	Low	High	Low	Low	Low
Mu 2017	Unclear	Unclear	High	Unclear	High	Low	High	Low	Low	Low
Nakhjavani 2021	Unclear	Low	High	Unclear	High	High	High	Low	Low	Low
Ning 2024	Unclear	Low	High	Low	High	Low	High	High	Low	Low
Niu 2019	Unclear	Low	High	Low	High	Low	High	Low	Low	Low
Peng 2016	Unclear	Low	High	Unclear	High	High	High	High	Low	Low
Peng 2019	Unclear	High	High	Unclear	High	Low	High	High	Low	Low
Qian 2021	Unclear	Unclear	High	Unclear	High	High	High	Low	Low	Low
Qin 2023	Unclear	High	High	Unclear	High	High	High	Low	Low	High
Raja 2011	Unclear	Low	High	Unclear	High	Low	High	Low	Low	Low
Rocha 2016	Unclear	Low	High	Low	High	High	High	Low	Low	Low
Saha 2016	Unclear	Low	High	Unclear	High	Low	High	Low	Low	Low
Sawant 2016	Unclear	Low	High	Unclear	High	High	High	High	Low	Low
Sharma 2024	Unclear	Unclear	High	Unclear	High	High	High	Low	Low	Low
Shi 2020	Unclear	Low	High	Low	High	High	High	Low	Low	Low

Shukla 2020	Unclear	Unclear	High	Unclear	High	Low	High	Low	Low	Low
Sinha 2016	Unclear	Unclear	High	Unclear	High	Low	High	Low	Low	Low
So 2013	Unclear	Low	High	Unclear	High	High	High	High	Low	Low
Soe 2018	Unclear	Unclear	High	Unclear	High	Low	High	Low	Low	Low
Song 2020	Unclear	Low	High	Low	High	Low	High	Low	Low	Low
Suebsakwong 2019	Unclear	Low	High	Unclear	High	Low	High	High	Low	High
Sun 2020	Unclear	Low	High	Unclear	High	High	High	Low	Low	Low
Tan 2017	Unclear	Unclear	High	Unclear	High	Low	High	Low	Low	Low
Tan 2022	Unclear	High	High	Low	High	Low	High	Low	Low	Low
Tian 2024	Unclear	High	High	Unclear	High	Low	High	Low	Low	High
Tran 2012 (2)	Unclear	Low	High	Unclear	High	High	High	Low	Low	Low
Wakimoto 2008	Unclear	Unclear	High	Unclear	High	High	High	Low	Low	High
Wang 2012	Unclear	Low	High	Unclear	High	Low	High	High	Low	Low
Wang 2018	Unclear	Low	High	Unclear	High	Low	High	Low	Low	Low
Wang 2019	Unclear	Unclear	High	Unclear	High	Low	High	Low	Low	Low
Wang 2020 (1)	Unclear	Unclear	High	Unclear	High	Low	High	High	Low	Low
Wang 2020 (2)	Unclear	Low	High	Unclear	High	Low	High	Low	Low	Low
Wang 2020 (3)	Unclear	High	High	Unclear	High	Low	High	Low	Low	Low
Wang 2020 (4)	Unclear	Low	High	Unclear	High	Low	High	Low	Low	High
Wang 2021 (1)	Unclear	Low	High	Unclear	High	Low	High	Low	Low	Low
Wang 2021 (2)	Unclear	Unclear	High	Unclear	High	Low	High	Low	Low	Low
Wang 2022 (1)	Unclear	Low	High	Unclear	High	Low	High	Unclear	Low	Low
Wang 2024	Unclear	Low	High	Unclear	High	Low	High	High	Low	Low
Wu 2020	Unclear	Low	High	Unclear	High	Low	High	Unclear	Low	Low
Wu 2022	Unclear	Low	High	Unclear	High	Low	High	Low	Low	Low
Xia 2022	Unclear	Low	High	Unclear	High	Low	High	Low	Low	Low
Xu 2021	Unclear	Low	High	Unclear	High	Low	High	Unclear	Low	Low

Xu 2022	Unclear	Low	High	Low	High	Low	High	Unclear	Low	Low
Yan 2018	Unclear	Unclear	High	Unclear	High	Low	High	Unclear	Low	High
Yang 2019	Unclear	Low	High	Unclear	High	Low	High	Low	Low	High
Yang 2024 (1)	Unclear	Low	High	Unclear	High	High	High	Unclear	Low	Low
Yang 2024 (2)	Unclear	Low	High	Unclear	High	Low	High	Low	Low	Low
Yang 2024 (3)	Unclear	Low	High	Low	High	Low	High	Low	Low	Low
Ye 2023	Unclear	Unclear	High	Low	High	Low	High	Low	Low	Low
Yin 2021	Unclear	Low	High	Low	High	Low	High	Low	Low	Low
Yuan 2017	Unclear	Low	High	Unclear	High	Low	High	Low	Low	Low
Yue 2016	Unclear	Low	High	Unclear	High	Low	High	Low	Low	Low
Zhang 2008	Unclear	Low	High	Unclear	High	Low	High	Low	Low	Low
Zhang 2015	Unclear	Unclear	High	Low	High	High	High	Low	Low	Low
Zhang 2016	Unclear	Unclear	High	Unclear	High	Low	High	Low	Low	High
Zhang 2017	Unclear	Low	High	Unclear	High	Low	High	Low	Low	Low
Zhang 2018	Unclear	Low	High	Unclear	High	Low	High	Unclear	Low	Low
Zhang 2020	Unclear	Unclear	High	Low	High	Low	High	Low	Low	Low
Zhang 2021 (1)	Unclear	Unclear	High	Low	High	Low	High	Low	Low	High
Zhang 2021 (2)	Unclear	Low	High	Unclear	High	Low	High	Low	Low	Low
Zhang 2022 (1)	Unclear	Low	High	Low	High	Low	High	Low	Low	Low
Zhang 2022 (2)	Unclear	Unclear	High	Unclear	High	High	High	Low	Low	Low
Zhang 2022 (3)	Unclear	Unclear	High	Low	High	High	High	Low	Low	Low
Zhang 2022 (4)	Unclear	Low	High	Unclear	High	High	High	Low	Low	Low
Zhang 2023 (2)	Unclear	Low	High	Low	High	High	High	Low	Low	Low
Zhang 2023 1	Unclear	Low	High	Unclear	High	Low	High	Unclear	Low	Low
Zhang 2024	Unclear	Unclear	High	Unclear	High	Low	High	Low	Low	High
Zhao 2018	Unclear	Unclear	High	Unclear	High	Low	High	Low	Low	Low
Zhao 2019 (1)	Unclear	Low	High	Low	High	Low	High	Low	Low	Low

Zhao 2019 (2)	Unclear	Unclear	High	Low	High	Low	High	Unclear	Low	Low
Zhao 2024	Unclear	Low	High	Low	High	Low	High	Unclear	Low	Low
Zheng 2018	Unclear	Low	High	Low	High	Low	High	Unclear	Low	Low
Zheng 2021	Unclear	Unclear	High	Low	High	Low	High	Low	Low	Low
Zhou 2020	Unclear	Unclear	High	Unclear	High	Low	High	Low	Low	Low
Zhou 2023	Unclear	Unclear	High	Low	High	Low	High	Low	Low	Low
Zhu 2023	Unclear	High	High	Unclear	High	High	High	Low	Low	Low
Zuo 2022	Unclear	Low	High	Unclear	High	Low	High	Low	Low	Low

References Table S6

A Ali, A., A Hussein, M., A Emara, A., Abd Elrahman, A. M., A Hassan, A., A Abdelghaney, A., M Bastawey, A., M Maher, A., N Al-Wadayi, A.-M., A Shalaby, M., M Mohamed, M., Gamal El Din, M. A., A Muhammad, S., S Ewees, A., Nasr-Eldin, M. S., A Alshrief, D., H Mohamed, A., Mostafa, H., El-Ella, A. A., … A Hussein, M. (2022). CEG-AgNPs Ameliorates DMBA-Induced Mammary Carcinogenicity by Alleviating Cytokines Expression. *Pakistan Journal of Biological Sciences : PJBS*, *25*(6), 485–494. https://doi.org/10.3923/pjbs.2022.485.494

Abdelmoneem, M. A., Abd Elwakil, M. M., Khattab, S. N., Helmy, M. W., Bekhit, A. A., Abdulkader, M. A., Zaky, A., Teleb, M., Elkhodairy, K. A., Albericio, F., & Elzoghby, A. O. (2021). Lactoferrin-dual drug nanoconjugate: Synergistic anti-tumor efficacy of docetaxel and the NF-κB inhibitor celastrol. *Materials Science and Engineering: C*, *118*, 111422. https://doi.org/10.1016/j.msec.2020.111422

Akl, M., Foudah, A., Ebrahim, H., Meyer, S., & Sayed, K. (2014). The Marine-Derived Sipholenol A-4-O-3′,4′-Dichlorobenzoate Inhibits Breast Cancer Growth and Motility in Vitro and in Vivo through the Suppression of Brk and FAK Signaling. *Marine Drugs*, *12*(4), 2282–2304. https://doi.org/10.3390/md12042282

Al Marzouqi, N., Iratni, R., Nemmar, A., Arafat, K., Ahmed Al Sultan, M., Yasin, J., Collin, P., Mester, J., Adrian, T. E., & Attoub, S. (2011). Frondoside A inhibits human breast cancer cell survival, migration, invasion and the growth of breast tumor xenografts. *European Journal of Pharmacology*, *668*(1–2), 25–34. https://doi.org/10.1016/j.ejphar.2011.06.023

Aribi, A., Gery, S., Lee, D. H., Thoennissen, N. H., Thoennissen, G. B., Alvarez, R., Ho, Q., Lee, K., Doan, N. B., Chan, K. T., Toh, M., Said, J. W., & Koeffler, H. P. (2013). The triterpenoid cucurbitacin B augments the antiproliferative activity of chemotherapy in human breast cancer. *International Journal of Cancer*, *132*(12), 2730–2737. https://doi.org/10.1002/ijc.27950

Arif, R., Bukhari, S. A., Mustafa, G., Ahmed, S., & Albeshr, M. F. (2024). Network Pharmacology and Experimental Validation to Explore the Potential Mechanism of Nigella sativa for the Treatment of Breast Cancer. *Pharmaceuticals*, *17*(5), 617. https://doi.org/10.3390/ph17050617

Ball, M. S., Bhandari, R., Torres, G. M., Martyanov, V., ElTanbouly, M. A., Archambault, K., Whitfield, M. L., Liby, K. T., & Pioli, P. A. (2020). CDDO-Me Alters the Tumor Microenvironment in Estrogen Receptor Negative Breast Cancer. *Scientific Reports*, *10*(1), 6560. https://doi.org/10.1038/s41598-020-63482-x

Bao, Y., Zhang, S., Chen, Z., Chen, A. T., Ma, J., Deng, G., Xu, W., Zhou, J., Yu, Z.-Q., Yao, G., & Chen, J. (2020). Synergistic Chemotherapy for Breast Cancer and Breast Cancer Brain Metastases via Paclitaxel-Loaded Oleanolic Acid Nanoparticles. *Molecular Pharmaceutics*, *17*(4), 1343–1351. https://doi.org/10.1021/acs.molpharmaceut.0c00044

Bishayee, A., Mandal, A., Thoppil, R. J., Darvesh, A. S., & Bhatia, D. (2013). Chemopreventive effect of a novel oleanane triterpenoid in a chemically induced rodent model of breast cancer. *International Journal of Cancer*, *133*(5), 1054–1063. https://doi.org/10.1002/ijc.28108

Blaskovich, M. A., Sun, J., Cantor, A., Turkson, J., Jove, R., & Sebti, S. M. (2003). Discovery of JSI-124 (cucurbitacin I), a selective Janus kinase/signal transducer and activator of transcription 3 signaling pathway inhibitor with potent antitumor activity against human and murine cancer cells in mice. *Cancer Research*, *63*(6), 1270–1279.

Cai, Y., Zheng, Y., Gu, J., Wang, S., Wang, N., Yang, B., Zhang, F., Wang, D., Fu, W., & Wang, Z. (2018). Betulinic acid chemosensitizes breast cancer by triggering ER stress-mediated apoptosis by directly targeting GRP78. *Cell Death & Disease*, *9*(6), 636. https://doi.org/10.1038/s41419-018-0669-8

Cevatemre, B., Erkısa, M., Aztopal, N., Karakas, D., Alper, P., Tsimplouli, C., Sereti, E., Dimas, K., Armutak, E. I. I., Gurevin, E. G., Uvez, A., Mori, M., Berardozzi, S., Ingallina, C., D'Acquarica, I., Botta, B., Ozpolat, B., & Ulukaya, E. (2018). A promising natural product, pristimerin, results in cytotoxicity against breast cancer stem cells in vitro and xenografts in vivo through apoptosis and an incomplete autopaghy in breast cancer. *Pharmacological Research*, *129*, 500–514. https://doi.org/10.1016/j.phrs.2017.11.027

Chang, Y., Fu, Q., Lu, Z., Jin, Q., Jin, T., & Zhang, M. (2024). Ginsenoside Rg3 combined with near-infrared photothermal reversal of multidrug resistance in breast cancer MCF-7/ADR cells. *Food Science & Nutrition*, *12*(8), 5750–5761. https://doi.org/10.1002/fsn3.4205

Chen, K., Zhu, X., Sun, R., Zhao, L., Zhao, J., Wu, X., Wang, C., & Zeng, H. (2024). Oleanolic acid derivative self-assembled aggregates based on heparin and chitosan for breast cancer therapy. *International Journal of Biological Macromolecules*, *277*, 134431. https://doi.org/10.1016/j.ijbiomac.2024.134431

Cheng, Y., Zhong, X., Nie, X., Gu, H., Wu, X., Li, R., Wu, Y., Lv, K., Leung, G. P.-H., Fu, C., Lee, S. M.-Y., Zhang, J., & Li, J. (2023). Glycyrrhetinic acid suppresses breast cancer metastasis by inhibiting M2-like macrophage polarization via activating JNK1/2 signaling. *Phytomedicine*, *114*, 154757. https://doi.org/10.1016/j.phymed.2023.154757

Choi, S., Oh, J., & Kim, S. (2011). Ginsenoside Rh2 induces Bcl-2 family proteins-mediated apoptosis *in vitro* and in xenografts *in vivo* models. *Journal of Cellular Biochemistry*, *112*(1), 330–340. https://doi.org/10.1002/jcb.22932

Chu, Y., Zhang, W., Kanimozhi, G., Brindha, G. R., & Tian, D. (2020). Ginsenoside Rg1 Induces Apoptotic Cell Death in Triple-Negative Breast Cancer Cell Lines and Prevents Carcinogen-Induced Breast Tumorigenesis in Sprague Dawley Rats. *Evidence-Based Complementary and Alternative Medicine*, *2020*(1). https://doi.org/10.1155/2020/8886955

Chun, J., & Kim, Y. S. (2013). Platycodin D inhibits migration, invasion, and growth of MDA-MB-231 human breast cancer cells via suppression of EGFR-mediated Akt and MAPK pathways. *Chemico-Biological Interactions*, *205*(3), 212–221. https://doi.org/10.1016/j.cbi.2013.07.002

Cun, X., Chen, J., Li, M., He, X., Tang, X., Guo, R., Deng, M., Li, M., Zhang, Z., & He, Q. (2019). Tumor-Associated Fibroblast-Targeted Regulation and Deep Tumor Delivery of Chemotherapeutic Drugs with a Multifunctional Size-Switchable Nanoparticle. *ACS Applied Materials & Interfaces*, *11*(43), 39545–39559. https://doi.org/10.1021/acsami.9b13957

Damle, A. A., Pawar, Y. P., & Narkar, A. A. (2013). Anticancer activity of betulinic acid on MCF-7 tumors in nude mice. *Indian Journal of Experimental Biology*, *51*(7), 485–491.

Dasgupta, A., Sawant, M. A., Kavishwar, G., Lavhale, M., & Sitasawad, S. (2016). AECHL-1 targets breast cancer progression via inhibition of metastasis, prevention of EMT and suppression of Cancer Stem Cell characteristics. *Scientific Reports*, *6*(1), 38045. https://doi.org/10.1038/srep38045

De Angel, R. E., Smith, S. M., Glickman, R. D., Perkins, S. N., & Hursting, S. D. (2010). Antitumor Effects of Ursolic Acid in a Mouse Model of Postmenopausal Breast Cancer. *Nutrition and Cancer*, *62*(8), 1074–1086. https://doi.org/10.1080/01635581.2010.492092

Dong, Y., Fu, R., Yang, J., Ma, P., Liang, L., Mi, Y., & Fan, D. (2019). <p>Folic acid-modified ginsenoside Rg5-loaded bovine serum albumin nanoparticles for targeted cancer therapy in vitro and in vivo</p>. *International Journal of Nanomedicine*, *Volume 14*, 6971–6988. https://doi.org/10.2147/IJN.S210882

Duan, Z., Wei, B., Deng, J., Mi, Y., Dong, Y., Zhu, C., Fu, R., Qu, L., & Fan, D. (2018). The anti-tumor effect of ginsenoside Rh4 in MCF-7 breast cancer cells in vitro and in vivo. *Biochemical and Biophysical Research Communications*, *499*(3), 482–487. https://doi.org/10.1016/j.bbrc.2018.03.174

Dutta, S., Chakraborty, P., Basak, S., Ghosh, S., Ghosh, N., Chatterjee, S., Dewanjee, S., & Sil, P. C. (2022). Synthesis, characterization, and evaluation of in vitro cytotoxicity and in vivo antitumor activity of asiatic acid-loaded poly lactic-co-glycolic acid nanoparticles: A strategy of treating breast cancer. *Life Sciences*, *307*, 120876. https://doi.org/10.1016/j.lfs.2022.120876

Elaidy, S. M., El-Kherbetawy, M. K., Abed, S. Y., Alattar, A., Alshaman, R., Eladl, M. A., Alamri, E. S., Al balawi, A. N., Zaid, A., Elkazzaz, A. Y., Abdelkhalig, S. M., Hamed, Z. E., & Zaitone, S. A. (2023). α-Hederin Saponin Augments the Chemopreventive Effect of Cisplatin against Ehrlich Tumors and Bioinformatic Approach Identifying the Role of SDF1/CXCR4/p-AKT-1/NFκB Signaling. *Pharmaceuticals*, *16*(3), 405. https://doi.org/10.3390/ph16030405

Elhasany, K. A., Khattab, S. N., Bekhit, A. A., Ragab, D. M., Abdulkader, M. A., Zaky, A., Helmy, M. W., Ashour, H. M. A., Teleb, M., Haiba, N. S., & Elzoghby, A. O. (2020). Combination of magnetic targeting with synergistic inhibition of NF-κB and glutathione via micellar drug nanomedicine enhances its anti-tumor efficacy. *European Journal of Pharmaceutics and Biopharmaceutics*, *155*, 162–176. https://doi.org/10.1016/j.ejpb.2020.08.004

Farhangfar, S. D., Fesahat, F., Zare-Zardini, H., Dehghan-Manshadi, M., Zare, F., Miresmaeili, S. M., Vajihinejad, M., & Soltaninejad, H. (2022). In vivo study of anticancer activity of ginsenoside Rh2-containing arginine-reduced graphene in a mouse model of breast cancer. *Iranian Journal of Basic Medical Sciences*, *25*(12), 1442–1451. https://doi.org/10.22038/IJBMS.2022.66065.14524

Feng, B., Zhao, C., Li, J., Yu, J., Zhang, Y., Zhang, X., Tian, T., & Zhao, L. (2020). The Novel Synthetic Triterpene Methyl 3β-O-[4-(2-Aminoethylamino)-4-oxo-butyryl]olean-12-ene-28-oate Inhibits Breast Tumor Cell Growth in Vitro and in Vivo. *Chemical and Pharmaceutical Bulletin*, *68*(10), 962–970. https://doi.org/10.1248/cpb.c20-00353

Fu, S., Wang, M., Li, B., Li, X., Cheng, J., Zhao, H., Zhang, H., Dong, A., Lu, W., & Yang, X. (2023). Bionic natural small molecule co-assemblies towards targeted and synergistic Chemo/PDT/CDT. *Biomaterials Research*, *27*(1). https://doi.org/10.1186/s40824-023-00380-z

Gan, X., Wang, F., Luo, J., Zhao, Y., Wang, Y., Yu, C., & Chen, J. (2024). Proteolysis Targeting Chimeras (PROTACs) based on celastrol induce multiple protein degradation for triple-negative breast cancer treatment. *European Journal of Pharmaceutical Sciences*, *192*, 106624. https://doi.org/10.1016/j.ejps.2023.106624

Gao, J.-L., Shui, Y.-M., Jiang, W., Huang, E.-Y., Shou, Q.-Y., Ji, X., He, B.-C., Lv, G.-Y., & He, T.-C. (2016). Hypoxia pathway and hypoxia-mediated extensive extramedullary hematopoiesis are involved in ursolic acid's anti-metastatic effect in 4T1 tumor bearing mice. *Oncotarget*, *7*(44), 71802–71816. https://doi.org/10.18632/oncotarget.12375

Gautam, S., Singh, N., Marwaha, D., Rai, N., Sharma, M., Tiwari, P., Singh, S., Kumar Bakshi, A., Kumar, A., Agarwal, N., Prakash Shukla, R., & Ranjan Mishra, P. (2024). Celastrol-loaded polymeric mixed micelles shows improved antitumor efficacy in 4 T1 bearing xenograft mouse model through spatial targeting. *International Journal of Pharmaceutics*, *659*, 124234. https://doi.org/10.1016/j.ijpharm.2024.124234

Gou, X., Bai, H., Liu, L., Chen, H., Shi, Q., Chang, L., Ding, M., Shi, Q., Zhou, M., Chen, W., & Zhang, L. (2020). Asiatic Acid Interferes with Invasion and Proliferation of Breast Cancer Cells by Inhibiting WAVE3 Activation through PI3K/AKT Signaling Pathway. *BioMed Research International*, *2020*(1). https://doi.org/10.1155/2020/1874387

Gupta, P., & Srivastava, S. K. (2014). Inhibition of HER2-integrin signaling by Cucurbitacin B leads to *in vitro* and *in vivo* breast tumor growth suppression. *Oncotarget*, *5*(7), 1812–1828. https://doi.org/10.18632/oncotarget.1743

He, K., Meng, X., Su, J., Jiang, S., Chu, M., & Huang, B. (2024). Oleanolic acid inhibits the tumor progression by regulating Lactobacillus through the cytokine-cytokine receptor interaction pathway in 4T1-induced mice breast cancer model. *Heliyon*, *10*(5), e27028. https://doi.org/10.1016/j.heliyon.2024.e27028

Hong, C., Liang, J., Xia, J., Zhu, Y., Guo, Y., Wang, A., Lu, C., Ren, H., Chen, C., Li, S., Wang, D., Zhan, H., & Wang, J. (2020). One Stone Four Birds: A Novel Liposomal Delivery System Multi-functionalized with Ginsenoside Rh2 for Tumor Targeting Therapy. *Nano-Micro Letters*, *12*(1), 129. https://doi.org/10.1007/s40820-020-00472-8

Hong, C., Wang, A., Xia, J., Liang, J., Zhu, Y., Wang, D., Zhan, H., Feng, C., Jiang, X., Pan, J., & Wang, J. (2024). Ginsenoside Rh2-Based Multifunctional Liposomes for Advanced Breast Cancer Therapy. *International Journal of Nanomedicine*, *Volume 19*, 2879–2888. https://doi.org/10.2147/IJN.S437733

Hong, Y., & Fan, D. (2019a). Ginsenoside Rk1 induces cell cycle arrest and apoptosis in MDA-MB-231 triple negative breast cancer cells. *Toxicology*, *418*, 22–31. https://doi.org/10.1016/j.tox.2019.02.010

Hong, Y., & Fan, D. (2019b). Ginsenoside Rk1 induces cell death through ROS-mediated PTEN/PI3K/Akt/mTOR signaling pathway in MCF-7 cells. *Journal of Functional Foods*, *57*, 255–265. https://doi.org/10.1016/j.jff.2019.04.019

Hu, J., Zhang, H., Li, J., Jiang, X., Zhang, Y., Wu, Q., Shen, liwen, Shi, J., & Gao, N. (2020). ROCK1 activation-mediated mitochondrial translocation of Drp1 and cofilin are required for arnidiol-induced mitochondrial fission and apoptosis. *Journal of Experimental & Clinical Cancer Research*, *39*(1), 37. https://doi.org/10.1186/s13046-020-01545-7

Hu, S., Zheng, W., & Jin, L. (2021). Astragaloside IV inhibits cell proliferation and metastasis of breast cancer via promoting the long noncoding RNA TRHDE-AS1. *Journal of Natural Medicines*, *75*(1), 156–166. https://doi.org/10.1007/s11418-020-01469-8

Huang, S., Huang, P., Wu, H., Wang, S., & Liu, G. (2021). Soyasaponin Ag inhibits triple-negative breast cancer progression via targeting the DUSP6/MAPK signaling. *Folia Histochemica et Cytobiologica*, *59*(4), 291–301. https://doi.org/10.5603/FHC.a2021.0029

Huang, S., Xiao, S., Li, X., Tao, R., Yang, Z., Gao, Z., Hu, J., Meng, Y., Zheng, G., & Chen, X. (2024). Development of Dual-Targeted Mixed Micelles Loaded with Celastrol and Evaluation on Triple-Negative Breast Cancer Therapy. *Pharmaceutics*, *16*(9), 1174. https://doi.org/10.3390/pharmaceutics16091174

Huang, T., Wang, Y., Shen, Y., Ao, H., Guo, Y., Han, M., & Wang, X. (2020). Preparation of high drug-loading celastrol nanosuspensions and their anti-breast cancer activities in vitro and in vivo. *Scientific Reports*, *10*(1), 8851. https://doi.org/10.1038/s41598-020-65773-9

Huynh, D. T. N., Jin, Y., Myung, C.-S., & Heo, K.-S. (2021). Ginsenoside Rh1 Induces MCF-7 Cell Apoptosis and Autophagic Cell Death through ROS-Mediated Akt Signaling. *Cancers*, *13*(8), 1892. https://doi.org/10.3390/cancers13081892

Hyer, M. L., Croxton, R., Krajewska, M., Krajewski, S., Kress, C. L., Lu, M., Suh, N., Sporn, M. B., Cryns, V. L., Zapata, J. M., & Reed, J. C. (2005). Synthetic Triterpenoids Cooperate with Tumor Necrosis Factor–Related Apoptosis-Inducing Ligand to Induce Apoptosis of Breast Cancer Cells. *Cancer Research*, *65*(11), 4799–4808. https://doi.org/10.1158/0008-5472.CAN-04-3319

Jeon, H., Huynh, D. T. N., Baek, N., Nguyen, T. L. L., & Heo, K.-S. (2021). Ginsenoside-Rg2 affects cell growth via regulating ROS-mediated AMPK activation and cell cycle in MCF-7 cells. *Phytomedicine*, *85*, 153549. https://doi.org/10.1016/j.phymed.2021.153549

Jiang, K., Han, L., Guo, Y., Zheng, G., Fan, L., Shen, Z., Zhao, R., & Shao, J. (2017). A carrier-free dual-drug nanodelivery system functionalized with aptamer specific targeting HER2-overexpressing cancer cells. *Journal of Materials Chemistry B*, *5*(46), 9121–9129. https://doi.org/10.1039/C7TB02562A

Jiang, K., Lu, Q., Li, Q., Ji, Y., Chen, W., & Xue, X. (2017). Astragaloside IV inhibits breast cancer cell invasion by suppressing Vav3 mediated Rac1/MAPK signaling. *International Immunopharmacology*, *42*, 195–202. https://doi.org/10.1016/j.intimp.2016.10.001

Jiao, L., Wang, S., Zheng, Y., Wang, N., Yang, B., Wang, D., Yang, D., Mei, W., Zhao, Z., & Wang, Z. (2019). Betulinic acid suppresses breast cancer aerobic glycolysis via caveolin-1/NF-κB/c-Myc pathway. *Biochemical Pharmacology*, *161*, 149–162. https://doi.org/10.1016/j.bcp.2019.01.016

Jin, H., Pi, J., Yang, F., Jiang, J., Wang, X., Bai, H., Shao, M., Huang, L., Zhu, H., Yang, P., Li, L., Li, T., Cai, J., & Chen, Z. W. (2016). Folate-Chitosan Nanoparticles Loaded with Ursolic Acid Confer Anti-Breast Cancer Activities in vitro and in vivo. *Scientific Reports*, *6*(1), 30782. https://doi.org/10.1038/srep30782

Jin, Z.-Q., Hao, J., Yang, X., He, J.-H., Liang, J., Yuan, J.-W., Mao, Y., Liu, D., Cao, R., Wu, X.-Z., Li, X., & Chen, D. (2018). Higenamine enhances the antitumor effects of cucurbitacin B in breast cancer by inhibiting the interaction of AKT and CDK2. *Oncology Reports*. https://doi.org/10.3892/or.2018.6629

Kai, W., Yating, S., Lin, M., Kaiyong, Y., Baojin, H., Wu, Y., Fangzhou, Y., & Yan, C. (2018). Natural product toosendanin reverses the resistance of human breast cancer cells to adriamycin as a novel PI3K inhibitor. *Biochemical Pharmacology*, *152*, 153–164. https://doi.org/10.1016/j.bcp.2018.03.022

Kallepu, S., Neeli, P. K., Mallappa, S., Nagendla, N. K., Reddy Mudiam, M. K., Mainkar, P. S., Kotamraju, S., & Chandrasekhar, S. (2020). sp 3 - Rich Glycyrrhetinic Acid Analogues Using Late-Stage Functionalization as Potential Breast Tumor Regressing Agents. *ChemMedChem*, *15*(19), 1826–1833. https://doi.org/10.1002/cmdc.202000400

Kim, E.-H., Deng, C., Sporn, M. B., Royce, D. B., Risingsong, R., Williams, C. R., & Liby, K. T. (2012). CDDO-Methyl Ester Delays Breast Cancer Development in *Brca1* -Mutated Mice. *Cancer Prevention Research*, *5*(1), 89–97. https://doi.org/10.1158/1940-6207.CAPR-11-0359

Kong, F., Liu, H., Zhao, C., & Qin, J. (2024). Targeted codelivery of doxorubicin and oleanolic acid by reduction responsive hyaluronic acid-based prodrug nano-micelles for enhanced antitumor activity and reduced toxicity. *International Journal of Biological Macromolecules*, *277*, 134135. https://doi.org/10.1016/j.ijbiomac.2024.134135

Kong, Y., Lu, Z.-L., Wang, J.-J., Zhou, R., Guo, J., Liu, J., Sun, H.-L., Wang, H., Song, W., Yang, J., & Xu, H.-X. (2016). Platycodin D, a metabolite of Platycodin grandiflorum, inhibits highly metastatic MDA-MB-231 breast cancer growth in vitro and in vivo by targeting the MDM2 oncogene. *Oncology Reports*, *36*(3), 1447–1456. https://doi.org/10.3892/or.2016.4935

Konopleva, M., Zhang, W., Shi, Y.-X., McQueen, T., Tsao, T., Abdelrahim, M., Munsell, M. F., Johansen, M., Yu, D., Madden, T., Safe, S. H., Hung, M.-C., & Andreeff, M. (2006). Synthetic triterpenoid 2-cyano-3,12-dioxooleana-1,9-dien-28-oic acid induces growth arrest in HER2-overexpressing breast cancer cells. *Molecular Cancer Therapeutics*, *5*(2), 317–328. https://doi.org/10.1158/1535-7163.MCT-05-0350

Lan, J.-S., Qin, Y.-H., Liu, L., Zeng, R.-F., Yang, Y., Wang, K., Ding, Y., Zhang, T., & Ho, R. J. (2021). A Carrier-Free Folate Receptor-Targeted Ursolic Acid/Methotrexate Nanodelivery System for Synergetic Anticancer Therapy. *International Journal of Nanomedicine*, *Volume 16*, 1775–1787. https://doi.org/10.2147/IJN.S287806

Lee, S. J., Lee, J. S., Lee, E., Lim, T.-G., & Byun, S. (2018). The ginsenoside metabolite compound K inhibits hormone-independent breast cancer through downregulation of cyclin D1. *Journal of Functional Foods*, *46*, 159–166. https://doi.org/10.1016/j.jff.2018.04.050

Leng, J., Dai, X., Cheng, X., Zhou, H., Wang, D., Zhao, J., Ma, K., Cui, C., Wang, L., & Guo, Z. (2022). Biomimetic Cucurbitacin B-Polydopamine Nanoparticles for Synergistic Chemo-Photothermal Therapy of Breast Cancer. *Frontiers in Bioengineering and Biotechnology*, *10*. https://doi.org/10.3389/fbioe.2022.841186

Li, C., Wang, Z., Zhang, Y., Zhu, Y., Xu, M., Lei, H., & Zhang, D. (2024). Efficient Sequential Co-Delivery Nanosystem for Inhibition of Tumor and Tumor-Associated Fibroblast-Induced Resistance and Metastasis. *International Journal of Nanomedicine*, *Volume 19*, 1749–1766. https://doi.org/10.2147/IJN.S427783

Li, C., Xue, H.-G., Feng, L.-J., Wang, M.-L., Wang, P., & Gai, X.-D. (2017). The effect of saikosaponin D on doxorubicin pharmacokinetics and its MDR reversal in MCF-7/adr cell xenografts. *European Review for Medical and Pharmacological Sciences*, *21*(19), 4437–4445.

Li, X., Widjaya, A. S., Liu, J., Liu, X., Long, Z., & Jiang, Y. (2020). Cell-penetrating corosolic acid liposome as a functional carrier for delivering chemotherapeutic drugs. *Acta Biomaterialia*, *106*, 301–313. https://doi.org/10.1016/j.actbio.2020.02.013

Li, X., Zhu, G., Yao, X., Wang, N., Hu, R., Kong, Q., Zhou, D., Long, L., Cai, J., & Zhou, W. (2018). Celastrol induces ubiquitin-dependent degradation of mTOR in breast cancer cells. *OncoTargets and Therapy*, *Volume 11*, 8977–8985. https://doi.org/10.2147/OTT.S187315

Li, Y., Wang, P., Zou, Z., Pan, Q., Li, X., Liang, Z., Li, L., Lin, Y., Peng, X., Zhang, R., Tian, H., & Han, L. (2021). Ginsenoside (20S)-protopanaxatriol induces non-protective autophagy and apoptosis by inhibiting Akt/mTOR signaling pathway in triple-negative breast cancer cells. *Biochemical and Biophysical Research Communications*, *583*, 184–191. https://doi.org/10.1016/j.bbrc.2021.10.067

Liang, J., Zhang, X., Yuan, J., Zhang, H., Liu, D., Hao, J., Ji, W., Wu, X., & Chen, D. (2019). Cucurbitacin B inhibits the migration and invasion of breast cancer cells by altering the biomechanical properties of cells. *Phytotherapy Research*, *33*(3), 618–630. https://doi.org/10.1002/ptr.6250

Liang, Z., Pan, R., Meng, X., Su, J., Guo, Y., Wei, G., Zhang, Z., & He, K. (2021). Transcriptome study of oleanolic acid in the inhibition of breast tumor growth based on high-throughput sequencing. *Aging*, *13*(19), 22883–22897. https://doi.org/10.18632/aging.203582

Liby, K., Risingsong, R., Royce, D. B., Williams, C. R., Yore, M. M., Honda, T., Gribble, G. W., Lamph, W. W., Vannini, N., Sogno, I., Albini, A., & Sporn, M. B. (2008). Prevention and Treatment of Experimental Estrogen Receptor–Negative Mammary Carcinogenesis by the Synthetic Triterpenoid CDDO-Methyl Ester and the Rexinoid LG100268. *Clinical Cancer Research*, *14*(14), 4556–4563. https://doi.org/10.1158/1078-0432.CCR-08-0040

Lim, G.-E., Sung, J. Y., Yu, S., Kim, Y., Shim, J., Kim, H. J., Cho, M. L., Lee, J.-S., & Kim, Y.-N. (2020). Pygenic Acid A (PA) Sensitizes Metastatic Breast Cancer Cells to Anoikis and Inhibits Metastasis In Vivo. *International Journal of Molecular Sciences*, *21*(22), 8444. https://doi.org/10.3390/ijms21228444

Ling, X., Konopleva, M., Zeng, Z., Ruvolo, V., Stephens, L. C., Schober, W., McQueen, T., Dietrich, M., Madden, T. L., & Andreeff, M. (2007). The Novel Triterpenoid C-28 Methyl Ester of 2-Cyano-3, 12-Dioxoolen-1, 9-Dien-28-Oic Acid Inhibits Metastatic Murine Breast Tumor Growth through Inactivation of STAT3 Signaling. *Cancer Research*, *67*(9), 4210–4218. https://doi.org/10.1158/0008-5472.CAN-06-3629

Liu, C., Dong, L., Sun, Z., Wang, L., Wang, Q., Li, H., Zhang, J., & Wang, X. (2018). Esculentoside A suppresses breast cancer stem cell growth through stemness attenuation and apoptosis induction by blocking IL-6/STAT3 signaling pathway. *Phytotherapy Research*, *32*(11), 2299–2311. https://doi.org/10.1002/ptr.6172

Liu, J., Cai, Q., Wang, W., Lu, M., Liu, J., Zhou, F., Sun, M., Wang, G., & Zhang, J. (2020). Ginsenoside Rh2 pretreatment and withdrawal reactivated the pentose phosphate pathway to ameliorate intracellular redox disturbance and promoted intratumoral penetration of adriamycin. *Redox Biology*, *32*, 101452. https://doi.org/10.1016/j.redox.2020.101452

Liu, Y., & Fan, D. (2018). Ginsenoside Rg5 induces apoptosis and autophagy *via* the inhibition of the PI3K/Akt pathway against breast cancer in a mouse model. *Food & Function*, *9*(11), 5513–5527. https://doi.org/10.1039/C8FO01122B

Liu, Y., & Li, J. (2023). Self-assembling nanoarchitectonics of size-controllable celastrol nanoparticles for efficient cancer chemotherapy with reduced systemic toxicity. *Journal of Colloid and Interface Science*, *636*, 216–222. https://doi.org/10.1016/j.jcis.2022.12.162

Liu, Y., Liu, K., Li, C., Wang, L., Liu, J., He, J., Lei, J., & Liu, X. (2017). Self-assembled nanoparticles based on a carboxymethylcellulose–ursolic acid conjugate for anticancer combination therapy. *RSC Advances*, *7*(58), 36256–36268. https://doi.org/10.1039/C7RA05913B

Long, J., Hu, W., Ren, T., Wang, X., Lu, C., Pan, X., Wu, C., & Peng, T. (2024). Combating multidrug resistance of breast cancer with ginsenoside Rh2-irrigated nano-in-thermogel. *International Journal of Pharmaceutics*, *650*, 123718. https://doi.org/10.1016/j.ijpharm.2023.123718

Lu, L., Ao, H., Fu, J., Li, M., Guo, Y., Guo, Y., Han, M., Shi, R., & Wang, X. (2023). Ginsenoside Rb1 stabilized and paclitaxel / protopanaxadiol co-loaded nanoparticles for synergistic treatment of breast tumor. *Biomedicine & Pharmacotherapy*, *163*, 114870. https://doi.org/10.1016/j.biopha.2023.114870

Lubet, R. A., Townsend, R., Clapper, M. L., Juliana, M. M., Steele, V. E., McCormick, D. L., & Grubbs, C. J. (2016). 5MeCDDO Blocks Metabolic Activation but not Progression of Breast, Intestine, and Tongue Cancers. Is Antioxidant Response Element a Prevention Target? *Cancer Prevention Research*, *9*(7), 616–623. https://doi.org/10.1158/1940-6207.CAPR-15-0294

Luo, B., Song, L., Chen, L., Cai, Y., Zhang, M., & Wang, S. (2024). Ganoderic acid D attenuates gemcitabine resistance of triple-negative breast cancer cells by inhibiting glycolysis via HIF-1α destabilization. *Phytomedicine*, *129*, 155675. https://doi.org/10.1016/j.phymed.2024.155675

Luo, F., Yang, J., Yang, X., Mi, J., Ye, T., Li, G., & Xie, Y. (2024). Saikosaponin D potentiates the antineoplastic effects of doxorubicin in drug-resistant breast cancer through perturbing NQO1-mediated intracellular redox balance. *Phytomedicine*, *133*, 155945. https://doi.org/10.1016/j.phymed.2024.155945

Luo, X., Wang, H., & Ji, D. (2021). Carbon nanotubes (CNT)-loaded ginsenosides Rb3 suppresses the PD-1/PD-L1 pathway in triple-negative breast cancer. *Aging*, *13*(13), 17177–17189. https://doi.org/10.18632/aging.203131

Mandal, A., Bhatia, D., & Bishayee, A. (2013). Simultaneous disruption of estrogen receptor and Wnt/β-catenin signaling is involved in methyl amooranin-mediated chemoprevention of mammary gland carcinogenesis in rats. *Molecular and Cellular Biochemistry*, *384*(1–2), 239–250. https://doi.org/10.1007/s11010-013-1803-7

Mandal, A., Bhatia, D., & Bishayee, A. (2014). Suppression of inflammatory cascade is implicated in methyl amooranin-mediated inhibition of experimental mammary carcinogenesis. *Molecular Carcinogenesis*, *53*(12), 999–1010. https://doi.org/10.1002/mc.22067

Mertens-Talcott, S. U., Noratto, G. D., Li, X., Angel-Morales, G., Bertoldi, M. C., & Safe, S. (2013). Betulinic acid decreases ER-negative breast cancer cell growth in vitro and in vivo: Role of Sp transcription factors and microRNA-27a:ZBTB10. *Molecular Carcinogenesis*, *52*(8), 591–602. https://doi.org/10.1002/mc.21893

Mohapatra, P., Madhulika, S., Behera, S., Singh, P., Sa, P., Prasad, P., Swain, R. K., & Sahoo, S. K. (2023). Nimbolide-based nanomedicine inhibits breast cancer stem-like cells by epigenetic reprogramming of DNMTs-SFRP1-Wnt/β-catenin signaling axis. *Molecular Therapy Nucleic Acids*, *34*, 102031. https://doi.org/10.1016/j.omtn.2023.102031

Mu, L.-H., Wang, Y.-N., Wang, D.-X., Zhang, J., Liu, L., Dong, X.-Z., Hu, Y., & Liu, P. (2017). AG36 Inhibits Human Breast Cancer Cells Proliferation by Promotion of Apoptosis In vitro and In vivo. *Frontiers in Pharmacology*, *8*. https://doi.org/10.3389/fphar.2017.00015

Nakhjavani, M., Smith, E., Palethorpe, H. M., Tomita, Y., Yeo, K., Price, T. J., Townsend, A. R., & Hardingham, J. E. (2021). Anti-Cancer Effects of an Optimised Combination of Ginsenoside Rg3 Epimers on Triple Negative Breast Cancer Models. *Pharmaceuticals*, *14*(7), 633. https://doi.org/10.3390/ph14070633

Ning, J.-Y., Zhang, Z.-H., Zhang, J., Liu, Y.-M., Li, G.-C., Wang, A.-M., Li, Y., Shan, X., Wang, J.-H., Zhang, X., & Zhao, Y. (2024). Ginsenoside Rg3 decreases breast cancer stem-like phenotypes through impairing MYC mRNA stability. *American Journal of Cancer Research*, *14*(2), 601–615. https://doi.org/10.62347/GYXE7741

Niu, S., Williams, G. R., Wu, J., Wu, J., Zhang, X., Zheng, H., Li, S., & Zhu, L.-M. (2019). A novel chitosan-based nanomedicine for multi-drug resistant breast cancer therapy. *Chemical Engineering Journal*, *369*, 134–149. https://doi.org/10.1016/j.cej.2019.02.201

Peng, B., He, R., Xu, Q., Yang, Y., Hu, Q., Hou, H., Liu, X., & Li, J. (2019). Ginsenoside 20(S)-protopanaxadiol inhibits triple-negative breast cancer metastasis in vivo by targeting EGFR-mediated MAPK pathway. *Pharmacological Research*, *142*, 1–13. https://doi.org/10.1016/j.phrs.2019.02.003

Peng, Y., Zhong, Y., & Li, G. (2016). Tubeimoside-1 suppresses breast cancer metastasis through downregulation of CXCR4 chemokine receptor expression. *BMB Reports*, *49*(9), 502–507. https://doi.org/10.5483/BMBRep.2016.49.9.030

Qian, Y., Zhang, J., Xu, R., Li, Q., Shen, Q., & Zhu, G. (2021). Nanoparticles based on polymers modified with pH-sensitive molecular switch and low molecular weight heparin carrying Celastrol and ferrocene for breast cancer treatment. *International Journal of Biological Macromolecules*, *183*, 2215–2226. https://doi.org/10.1016/j.ijbiomac.2021.05.204

Qin, Y., Wang, Z., Wang, X., Zhang, T., Hu, Y., Wang, D., Sun, H., Zhang, L., & Zhu, Y. (2023). Therapeutic effect of multifunctional celastrol nanoparticles with mitochondrial alkaline drug release in breast cancer. *Materials Today Advances*, *17*, 100328. https://doi.org/10.1016/j.mtadv.2022.100328

Raja, S. M., Clubb, R. J., Ortega-Cava, C., Williams, S. H., Bailey, T. A., Duan, L., Zhao, X., Reddi, A. L., Nyong, A. M., Natarajan, A., Band, V., & Band, H. (2011). Anticancer activity of Celastrol in combination with ErbB2-targeted therapeutics for treatment of ErbB2-overexpressing breast cancers. *Cancer Biology & Therapy*, *11*(2), 263–276. https://doi.org/10.4161/cbt.11.2.13959

Rocha, T. G. R., Lopes, S. C. de A., Cassali, G. D., Ferreira, Ê., Veloso, E. S., Leite, E. A., Braga, F. C., Ferreira, L. A. M., Balvay, D., Garofalakis, A., Oliveira, M. C., & Tavitian, B. (2016). Evaluation of Antitumor Activity of Long-Circulating and pH-Sensitive Liposomes Containing Ursolic Acid in Animal Models of Breast Tumor and Gliosarcoma. *Integrative Cancer Therapies*, *15*(4), 512–524. https://doi.org/10.1177/1534735416628273

Saha, S., Ghosh, M., & Dutta, S. K. (2016). Role of metabolic modulator Bet-CA in altering mitochondrial hyperpolarization to suppress cancer associated angiogenesis and metastasis. *Scientific Reports*, *6*(1), 23552. https://doi.org/10.1038/srep23552

Sawant, M. A., Dasgupta, A., Lavhale, M. S., & Sitasawad, S. L. (2016). Novel triterpenoid AECHL-1 induces apoptosis in breast cancer cells by perturbing the mitochondria–endoplasmic reticulum interactions and targeting diverse apoptotic pathways. *Biochimica et Biophysica Acta (BBA) - General Subjects*, *1860*(6), 1056–1070. https://doi.org/10.1016/j.bbagen.2016.02.002

Sharma, R., Yadav, V., Jha, S., Dighe, S., & Jain, S. (2024). Unveiling the potential of ursolic acid modified hyaluronate nanoparticles for combination drug therapy in triple negative breast cancer. *Carbohydrate Polymers*, *338*, 122196. https://doi.org/10.1016/j.carbpol.2024.122196

Shi, J., Li, J., Li, J., Li, R., Wu, X., Gao, F., Zou, L., Mak, W. W. S., Fu, C., Zhang, J., & Leung, G. P.-H. (2021). Synergistic breast cancer suppression efficacy of doxorubicin by combination with glycyrrhetinic acid as an angiogenesis inhibitor. *Phytomedicine*, *81*, 153408. https://doi.org/10.1016/j.phymed.2020.153408

Shukla, R. P., Urandur, S., Banala, V. T., Marwaha, D., Gautam, S., Rai, N., Singh, N., Tiwari, P., Shukla, P., & Mishra, P. R. (2021). Development of putrescine anchored nano-crystalsomes bearing doxorubicin and oleanolic acid: deciphering their role in inhibiting metastatic breast cancer. *Biomaterials Science*, *9*(5), 1779–1794. https://doi.org/10.1039/D0BM01033B

Sinha, S., Khan, S., Shukla, S., Lakra, A. D., Kumar, S., Das, G., Maurya, R., & Meeran, S. M. (2016). Cucurbitacin B inhibits breast cancer metastasis and angiogenesis through VEGF-mediated suppression of FAK/MMP-9 signaling axis. *The International Journal of Biochemistry & Cell Biology*, *77*, 41–56. https://doi.org/10.1016/j.biocel.2016.05.014

So, J. Y., Wahler, J. E., Yoon, T., Smolarek, A. K., Lin, Y., Shih, W. J., Maehr, H., Uskokovic, M., Liby, K. T., Sporn, M. B., & Suh, N. (2013). Oral Administration of a Gemini Vitamin D Analog, a Synthetic Triterpenoid and the Combination Prevents Mammary Tumorigenesis Driven by ErbB2 Overexpression. *Cancer Prevention Research*, *6*(9), 959–970. https://doi.org/10.1158/1940-6207.CAPR-13-0087

Soe, Z. C., Thapa, R. K., Ou, W., Gautam, M., Nguyen, H. T., Jin, S. G., Ku, S. K., Oh, K. T., Choi, H.-G., Yong, C. S., & Kim, J. O. (2018). Folate receptor-mediated celastrol and irinotecan combination delivery using liposomes for effective chemotherapy. *Colloids and Surfaces B: Biointerfaces*, *170*, 718–728. https://doi.org/10.1016/j.colsurfb.2018.07.013

Song, J.-H., Eum, D.-Y., Park, S.-Y., Jin, Y.-H., Shim, J.-W., Park, S.-J., Kim, M.-Y., Park, S.-J., Heo, K., & Choi, Y.-J. (2020). Inhibitory effect of ginsenoside Rg3 on cancer stemness and mesenchymal transition in breast cancer via regulation of myeloid-derived suppressor cells. *PLOS ONE*, *15*(10), e0240533. https://doi.org/10.1371/journal.pone.0240533

Suebsakwong, P., Wang, J., Khetkam, P., Weerapreeyakul, N., Wu, J., Du, Y., Yao, Z.-J., Li, J.-X., & Suksamrarn, A. (2019). A Bioreductive Prodrug of Cucurbitacin B Significantly Inhibits Tumor Growth in the 4T1 Xenograft Mice Model. *ACS Medicinal Chemistry Letters*, *10*(10), 1400–1406. https://doi.org/10.1021/acsmedchemlett.9b00161

Sun, K., Yu, W., Ji, B., Chen, C., Yang, H., Du, Y., Song, M., Cai, H., Yan, F., & Su, R. (2020). Saikosaponin D loaded macrophage membrane-biomimetic nanoparticles target angiogenic signaling for breast cancer therapy. *Applied Materials Today*, *18*, 100505. https://doi.org/10.1016/j.apmt.2019.100505

Tan, H., Zhang, M., Xu, L., Zhang, X., & Zhao, Y. (2022). Gypensapogenin H suppresses tumor growth and cell migration in triple-negative breast cancer by regulating PI3K/AKT/NF-κB/MMP-9 signaling pathway. *Bioorganic Chemistry*, *126*, 105913. https://doi.org/10.1016/j.bioorg.2022.105913

Tan, Y., Zhu, Y., Zhao, Y., Wen, L., Meng, T., Liu, X., Yang, X., Dai, S., Yuan, H., & Hu, F. (2018). Mitochondrial alkaline pH-responsive drug release mediated by Celastrol loaded glycolipid-like micelles for cancer therapy. *Biomaterials*, *154*, 169–181. https://doi.org/10.1016/j.biomaterials.2017.07.036

Tian, J., Chen, K., Zhang, Q., Qiu, C., Tong, H., Huang, J., Hao, M., Chen, J., Zhao, W., Wong, Y.-K., Gao, L., Luo, P., Wang, J., & Du, Q. (2024). Mechanism of Self-Assembled Celastrol-Erianin Nanomedicine for treatment of breast cancer. *Chemical Engineering Journal*, *499*, 155709. https://doi.org/10.1016/j.cej.2024.155709

Tran, K., Risingsong, R., Royce, D., Williams, C. R., Sporn, M. B., & Liby, K. (2012). The Synthetic Triterpenoid CDDO-Methyl Ester Delays Estrogen Receptor–Negative Mammary Carcinogenesis in Polyoma Middle T Mice. *Cancer Prevention Research*, *5*(5), 726–734. https://doi.org/10.1158/1940-6207.CAPR-11-0404

Wakimoto, N., Yin, D., O'Kelly, J., Haritunians, T., Karlan, B., Said, J., Xing, H., & Koeffler, H. P. (2008). Cucurbitacin B has a potent antiproliferative effect on breast cancer cells *in vitro* and *in vivo*. *Cancer Science*, *99*(9), 1793–1797. https://doi.org/10.1111/j.1349-7006.2008.00899.x

Wang, H., Xu, H., Chen, W., Cheng, M., Zou, L., Yang, Q., Chan, C. B., Zhu, H., Chen, C., Nie, J., & Jiao, B. (2022). Rab13 Sustains Breast Cancer Stem Cells by Supporting Tumor–Stroma Cross-talk. *Cancer Research*, *82*(11), 2124–2140. https://doi.org/10.1158/0008-5472.CAN-21-4097

Wang, J., Qiao, W., Zhao, H., & Yang, X. (2020). Paclitaxel and betulonic acid synergistically enhance antitumor efficacy by forming co-assembled nanoparticles. *Biochemical Pharmacology*, *182*, 114232. https://doi.org/10.1016/j.bcp.2020.114232

Wang, K., Tu, Y., Wan, J.-B., Chen, M., & He, C. (2020). Synergistic anti-breast cancer effect of pulsatilla saponin D and camptothecin through interrupting autophagic–lysosomal function and promoting p62-mediated ubiquitinated protein aggregation. *Carcinogenesis*, *41*(6), 804–816. https://doi.org/10.1093/carcin/bgz140

Wang, L., Tang, L., Yao, C., Liu, C., & Shu, Y. (2021). The Synergistic Effects of Celastrol in combination with Tamoxifen on Apoptosis and Autophagy in MCF-7 Cells. *Journal of Immunology Research*, *2021*, 1–13. https://doi.org/10.1155/2021/5532269

Wang, R., Wang, X., Jia, X., Wang, H., Li, W., & Li, J. (2020). Impacts of particle size on the cytotoxicity, cellular internalization, pharmacokinetics and biodistribution of betulinic acid nanosuspensions in combined chemotherapy. *International Journal of Pharmaceutics*, *588*, 119799. https://doi.org/10.1016/j.ijpharm.2020.119799

Wang, R., Yang, M., Li, G., Wang, X., Zhang, Z., Qiao, H., Chen, J., Chen, Z., Cui, X., & Li, J. (2019). Paclitaxel-betulinic acid hybrid nanosuspensions for enhanced anti-breast cancer activity. *Colloids and Surfaces B: Biointerfaces*, *174*, 270–279. https://doi.org/10.1016/j.colsurfb.2018.11.029

Wang, S., Chang, X., Zhang, J., Li, J., Wang, N., Yang, B., Pan, B., Zheng, Y., Wang, X., Ou, H., & Wang, Z. (2021). Ursolic Acid Inhibits Breast Cancer Metastasis by Suppressing Glycolytic Metabolism via Activating SP1/Caveolin-1 Signaling. *Frontiers in Oncology*, *11*. https://doi.org/10.3389/fonc.2021.745584

Wang, W., Zhang, X., Qin, J.-J., Voruganti, S., Nag, S. A., Wang, M.-H., Wang, H., & Zhang, R. (2012). Natural Product Ginsenoside 25-OCH3-PPD Inhibits Breast Cancer Growth and Metastasis through Down-Regulating MDM2. *PLoS ONE*, *7*(7), e41586. https://doi.org/10.1371/journal.pone.0041586

Wang, X., Liu, X., Guo, Y., Gong, T., Lu, W., Han, M., Guo, Y., & Wang, X. (2024). The Remarkable Anti-Breast Cancer Efficacy and Anti-Metastasis by Multifunctional Nanoparticles Co-Loading Squamocin, R848 and IR 780. *International Journal of Nanomedicine*, *Volume 19*, 4679–4699. https://doi.org/10.2147/IJN.S448860

Wang, Y., Zhao, L., Han, X., Wang, Y., Mi, J., Wang, C., Sun, D., Fu, Y., Zhao, X., Guo, H., & Wang, Q. (2020). Saikosaponin A Inhibits Triple-Negative Breast Cancer Growth and Metastasis Through Downregulation of CXCR4. *Frontiers in Oncology*, *9*. https://doi.org/10.3389/fonc.2019.01487

Wang, Y.-S., Li, G.-L., Zhu, S.-B., Jing, F.-C., Liu, R.-D., Li, S.-S., He, J., & Lei, J.-D. (2020). A Self-assembled Nanoparticle Platform Based on Amphiphilic Oleanolic Acid Polyprodrug for Cancer Therapy. *Chinese Journal of Polymer Science*, *38*(8), 819–829. https://doi.org/10.1007/s10118-020-2401-2

Wu, H., Wei, G., Luo, L., Li, L., Gao, Y., Tan, X., Wang, S., Chang, H., Liu, Y., Wei, Y., Song, J., Zhang, Z., & Huo, J. (2022). Ginsenoside Rg3 nanoparticles with permeation enhancing based chitosan derivatives were encapsulated with doxorubicin by thermosensitive hydrogel and anti-cancer evaluation of peritumoral hydrogel injection combined with PD-L1 antibody. *Biomaterials Research*, *26*(1). https://doi.org/10.1186/s40824-022-00329-8

Wu, X.-X., Yue, G. G.-L., Dong, J.-R., Lam, C. W.-K., Wong, C.-K., Qiu, M.-H., & Lau, C. B.-S. (2020). Actein Inhibits Tumor Growth and Metastasis in HER2-Positive Breast Tumor Bearing Mice via Suppressing AKT/mTOR and Ras/Raf/MAPK Signaling Pathways. *Frontiers in Oncology*, *10*. https://doi.org/10.3389/fonc.2020.00854

Xia, J., Zhang, S., Zhang, R., Wang, A., Zhu, Y., Dong, M., Ma, S., Hong, C., Liu, S., Wang, D., & Wang, J. (2022). Targeting therapy and tumor microenvironment remodeling of triple-negative breast cancer by ginsenoside Rg3 based liposomes. *Journal of Nanobiotechnology*, *20*(1), 414. https://doi.org/10.1186/s12951-022-01623-2

Xu, A.-L., Xue, Y.-Y., Tao, W.-T., Wang, S.-Q., & Xu, H.-Q. (2022). Oleanolic acid combined with olaparib enhances radiosensitization in triple negative breast cancer and hypoxia imaging with 18F-FETNIM micro PET/CT. *Biomedicine & Pharmacotherapy*, *150*, 113007. https://doi.org/10.1016/j.biopha.2022.113007

Xu, L., Zhang, X., Xiao, S., Li, X., Jiang, H., Wang, Z., Sun, B., & Zhao, Y. (2021). Panaxadiol as a major metabolite of AD-1 can significantly inhibit the proliferation and migration of breast cancer cells: In vitro and in vivo study. *Bioorganic Chemistry*, *116*, 105392. https://doi.org/10.1016/j.bioorg.2021.105392

Yan, X., Yang, L., Feng, G., Yu, Z., Xiao, M., Cai, W., Xing, Y., Bai, S., Guo, J., Wang, Z., Wang, T., & Zhang, R. (2018). Lup-20(29)-en-3β,28-di-yl-nitrooxy acetate affects MCF-7 proliferation through the crosstalk between apoptosis and autophagy in mitochondria. *Cell Death & Disease*, *9*(2), 241. https://doi.org/10.1038/s41419-017-0255-5

Yang, C., Qian, C., Zheng, W., Dong, G., Zhang, S., Wang, F., Wei, Z., Xu, Y., Wang, A., Zhao, Y., & Lu, Y. (2024). Ginsenoside Rh2 enhances immune surveillance of natural killer (NK) cells via inhibition of ERp5 in breast cancer. *Phytomedicine*, *123*, 155180. https://doi.org/10.1016/j.phymed.2023.155180

Yang, T., Li, X., Wang, X., Meng, X., Zhang, Z., Zhao, M., & Su, R. (2024). Combination of histological and metabolomic assessments to evaluate the potential pharmacological efficacy of saikosaponin D. *Journal of Pharmaceutical and Biomedical Analysis*, *242*, 116001. https://doi.org/10.1016/j.jpba.2024.116001

Yang, X., Liang, B., Zhang, L., Zhang, M., Ma, M., Qing, L., Yang, H., Huang, G., & Zhao, J. (2024). Ursolic acid inhibits the proliferation of triple-negative breast cancer stem-like cells through NRF2-mediated ferroptosis. *Oncology Reports*, *52*(1), 94. https://doi.org/10.3892/or.2024.8753

Yang, Y., Long, L., Zhang, X., Song, K., Wang, D., Xiong, X., Gao, H., & Sha, L. (2019). 16-Tigloyl linked barrigenol-like triterpenoid from Semen Aesculi and its anti-tumor activity *in vivo* and *in vitro*. *RSC Advances*, *9*(54), 31758–31772. https://doi.org/10.1039/C9RA06015D

Ye, Y., Xie, Y., Pei, L., Jiang, Z., Wu, C., & Liu, S. (2023). Platycodin D induces neutrophil apoptosis by downregulating PD-L1 expression to inhibit breast cancer pulmonary metastasis. *International Immunopharmacology*, *115*, 109733. https://doi.org/10.1016/j.intimp.2023.109733

Yin, L., Fan, Z., Liu, P., Chen, L., Guan, Z., Liu, Y., & Luo, Y. (2021). Anemoside A3 activates TLR4-dependent M1-phenotype macrophage polarization to represses breast tumor growth and angiogenesis. *Toxicology and Applied Pharmacology*, *432*, 115755. https://doi.org/10.1016/j.taap.2021.115755

Yuan, Z., Jiang, H., Zhu, X., Liu, X., & Li, J. (2017). Ginsenoside Rg3 promotes cytotoxicity of Paclitaxel through inhibiting NF-κB signaling and regulating Bax/Bcl-2 expression on triple-negative breast cancer. *Biomedicine & Pharmacotherapy*, *89*, 227–232. https://doi.org/10.1016/j.biopha.2017.02.038

Yue, G. G.-L., Xie, S., Lee, J. K.-M., Kwok, H.-F., Gao, S., Nian, Y., Wu, X.-X., Wong, C.-K., Qiu, M.-H., & Lau, C. B.-S. (2016). New potential beneficial effects of actein, a triterpene glycoside isolated from Cimicifuga species, in breast cancer treatment. *Scientific Reports*, *6*(1), 35263. https://doi.org/10.1038/srep35263

Zhang, B., Fu, R., Duan, Z., Shen, S., Zhu, C., & Fan, D. (2022). Ginsenoside CK induces apoptosis in triple-negative breast cancer cells by targeting glutamine metabolism. *Biochemical Pharmacology*, *202*, 115101. https://doi.org/10.1016/j.bcp.2022.115101

Zhang, E., Shi, H., Yang, L., Wu, X., & Wang, Z. (2017). Ginsenoside Rd regulates the Akt/mTOR/p70S6K signaling cascade and suppresses angiogenesis and breast tumor growth. *Oncology Reports*, *38*(1), 359–367. https://doi.org/10.3892/or.2017.5652

Zhang, H., Xu, H.-L., Wang, Y.-C., Lu, Z.-Y., Yu, X.-F., & Sui, D.-Y. (2018). 20(S)-Protopanaxadiol-Induced Apoptosis in MCF-7 Breast Cancer Cell Line through the Inhibition of PI3K/AKT/mTOR Signaling Pathway. *International Journal of Molecular Sciences*, *19*(4), 1053. https://doi.org/10.3390/ijms19041053

Zhang, J., He, Y., Zhou, Y., Hong, L., Jiang, Z., Zhao, Y., & Pan, Z. (2022). Epifriedelinol Ameliorates DMBA-Induced Breast Cancer in Albino Rats by Regulating the PI3K/AKT Pathway. *The Tohoku Journal of Experimental Medicine*, *257*(4), 2022.J030. https://doi.org/10.1620/tjem.2022.J030

Zhang, J., Xu, H., Wu, Y., Cho, W. C. S., Xian, Y., & Lin, Z. (2023). Synergistic Anti-Tumor Effect of Toosendanin and Paclitaxel on Triple-Negative Breast Cancer via Regulating ADORA2A-EMT Related Signaling. *Advanced Biology*, *7*(8). https://doi.org/10.1002/adbi.202300062

Zhang, J., Yang, F., Mei, X., Yang, R., Lu, B., Wang, Z., & Ji, L. (2022). Toosendanin and isotoosendanin suppress triple-negative breast cancer growth via inducing necrosis, apoptosis and autophagy. *Chemico-Biological Interactions*, *351*, 109739. https://doi.org/10.1016/j.cbi.2021.109739

Zhang, J., Zhang, Z., Huang, Z., Li, M., Yang, F., Wu, Z., Guo, Q., Mei, X., Lu, B., Wang, C., Wang, Z., & Ji, L. (2023). Isotoosendanin exerts inhibition on triple-negative breast cancer through abrogating TGF-β-induced epithelial–mesenchymal transition via directly targeting TGFβR1. *Acta Pharmaceutica Sinica B*, *13*(7), 2990–3007. https://doi.org/10.1016/j.apsb.2023.05.006

Zhang, J., Zhou, B., Sun, J., Chen, H., & Yang, Z. (2021). Betulin ameliorates 7,12-dimethylbenz(a)anthracene-induced rat mammary cancer by modulating MAPK and AhR/Nrf-2 signaling pathway. *Journal of Biochemical and Molecular Toxicology*, *35*(7). https://doi.org/10.1002/jbt.22779

ZHANG, L., ZHOU, J.-P., & YAO, J. (2015). Improved anti-tumor activity and safety profile of a paclitaxel-loaded glycyrrhetinic acid-graft-hyaluronic acid conjugate as a synergistically targeted drug delivery system. *Chinese Journal of Natural Medicines*, *13*(12), 915–924. https://doi.org/10.1016/S1875-5364(15)30097-2

Zhang, N., Liu, S., Shi, S., Chen, Y., Xu, F., Wei, X., & Xu, Y. (2020). Solubilization and delivery of Ursolic-acid for modulating tumor microenvironment and regulatory T cell activities in cancer immunotherapy. *Journal of Controlled Release*, *320*, 168–178. https://doi.org/10.1016/j.jconrel.2020.01.015

Zhang, Q., Kang, X., Yang, B., Wang, J., & Yang, F. (2008). Antiangiogenic Effect of Capecitabine Combined with Ginsenoside Rg3 on Breast Cancer in Mice. *Cancer Biotherapy and Radiopharmaceuticals*, *23*(5), 647–654. https://doi.org/10.1089/cbr.2008.0532

Zhang, S., Dong, Y., Chen, X., TAN, C. S. H., Li, M., Miao, K., & Lu, J.-H. (2022). Toosendanin, a late-stage autophagy inhibitor, sensitizes triple-negative breast cancer to irinotecan chemotherapy. *Chinese Medicine*, *17*(1), 55. https://doi.org/10.1186/s13020-022-00605-8

Zhang, Y., Liu, Q.-Z., Xing, S.-P., & Zhang, J.-L. (2016). Inhibiting effect of Endostar combined with ginsenoside Rg3 on breast cancer tumor growth in tumor-bearing mice. *Asian Pacific Journal of Tropical Medicine*, *9*(2), 180–183. https://doi.org/10.1016/j.apjtm.2016.01.010

Zhang, Y., Ma, X., Li, H., Zhuang, J., Feng, F., Liu, L., Liu, C., & Sun, C. (2021). Identifying the Effect of Ursolic Acid Against Triple-Negative Breast Cancer: Coupling Network Pharmacology With Experiments Verification. *Frontiers in Pharmacology*, *12*. https://doi.org/10.3389/fphar.2021.685773

Zhang, Y., Wang, Y., Zhang, H., Huang, S., Li, Y., Long, J., Han, Y., Lin, Q., Gong, T., Sun, X., Zhang, Z., & Zhang, L. (2024). Replacing cholesterol with asiatic acid to prolong circulation and enhance anti-metastatic effects of non-PEGylated liposomes. *Journal of Controlled Release*, *366*, 585–595. https://doi.org/10.1016/j.jconrel.2024.01.009

Zhao, Q., Liu, Y., Zhong, J., Bi, Y., Liu, Y., Ren, Z., Li, X., Jia, J., Yu, M., & Yu, X. (2019). Pristimerin induces apoptosis and autophagy via activation of ROS/ASK1/JNK pathway in human breast cancer in vitro and in vivo. *Cell Death Discovery*, *5*(1), 125. https://doi.org/10.1038/s41420-019-0208-0

Zhao, X., Liu, J., Ge, S., Chen, C., Li, S., Wu, X., Feng, X., Wang, Y., & Cai, D. (2019). Saikosaponin A Inhibits Breast Cancer by Regulating Th1/Th2 Balance. *Frontiers in Pharmacology*, *10*. https://doi.org/10.3389/fphar.2019.00624

Zhao, Y., Bai, Y., Li, M., Nie, X., Meng, H., Shosei, S., Liu, L., Yang, Q., Shen, M., & Li, Y. (2024). A pH-triggered N-oxide polyzwitterionic nano-drug loaded system for the anti-tumor immunity activation research. *Journal of Nanobiotechnology*, *22*(1), 420. https://doi.org/10.1186/s12951-024-02677-0

Zhao, Y., Tan, Y., Meng, T., Liu, X., Zhu, Y., Hong, Y., Yang, X., Yuan, H., Huang, X., & Hu, F. (2018). Simultaneous targeting therapy for lung metastasis and breast tumor by blocking the NF-κB signaling pathway using Celastrol-loaded micelles. *Drug Delivery*, *25*(1), 341–352. https://doi.org/10.1080/10717544.2018.1425778

Zheng, Y., Dai, Y., Liu, W., Wang, N., Cai, Y., Wang, S., Zhang, F., Liu, P., Chen, Q., & Wang, Z. (2019). Astragaloside IV enhances taxol chemosensitivity of breast cancer via caveolin-1-targeting oxidant damage. *Journal of Cellular Physiology*, *234*(4), 4277–4290. https://doi.org/10.1002/jcp.27196

Zheng, Y., Li, Z., Yang, Y., Shi, H., Chen, H., & Gao, Y. (2021). A nanosensitizer self-assembled from oleanolic acid and chlorin e6 for synergistic chemo/sono-photodynamic cancer therapy. *Phytomedicine*, *93*, 153788. https://doi.org/10.1016/j.phymed.2021.153788

Zhou, L., Wang, Z., Yu, S., Xiong, Y., Fan, J., Lyu, Y., Su, Z., Song, J., Liu, S., Sun, Q., & Lu, D. (2020). CDDO-Me Elicits Anti–Breast Cancer Activity by Targeting LRP6 and FZD7 Receptor Complex. *The Journal of Pharmacology and Experimental Therapeutics*, *373*(1), 149–159. https://doi.org/10.1124/jpet.119.263434

Zhou, M., Liao, J., Lai, W., Xu, R., Liu, W., Xie, D., Wang, F., Zhang, Z., Huang, J., Zhang, R., & Li, G. (2023). A celastrol-based nanodrug with reduced hepatotoxicity for primary and metastatic cancer treatment. *EBioMedicine*, *94*, 104724. https://doi.org/10.1016/j.ebiom.2023.104724

Zhu, Y., Wang, A., Zhang, S., Kim, J., Xia, J., Zhang, F., Wang, D., Wang, Q., & Wang, J. (2023). Paclitaxel-loaded ginsenoside Rg3 liposomes for drug-resistant cancer therapy by dual targeting of the tumor microenvironment and cancer cells. *Journal of Advanced Research*, *49*, 159–173. https://doi.org/10.1016/j.jare.2022.09.007

Zuo, S., Wang, J., An, X., Wang, Z., Zheng, X., & Zhang, Y. (2022). Fabrication of Ginsenoside-Based Nanodrugs for Enhanced Antitumor Efficacy on Triple-Negative Breast Cancer. *Frontiers in Bioengineering and Biotechnology*, *10*. https://doi.org/10.3389/fbioe.2022.945472

Table S7. Full-text articles excluded from the analysis

**Title of the article**	**Exclusion reason**	**Reference**
The IKK inhibitors celastrol and parthenolide inhibit breast cancer cell proliferation and migration <i>in vitro</i> and osteolytic bone metastasis <i>in vivo</i>	could not be retrieved	(Idris et al., 2008)
11-Keto-α-Boswellic Acid, a Novel Triterpenoid from <i>Boswellia</i> spp. with Chemotaxonomic Potential and Antitumor Activity against Triple-Negative Breast Cancer Cells	ex vivo	(Schmiech et al., 2021)
JSI124 inhibits breast cancer cell growth by suppressing the function of B cells via the downregulation of signal transducer and activator of transcription 3.	in vitro	(REN et al., 2014)
Inhibition of phosphorylated STAT3 by cucurbitacin I enhances chemoradiosensitivity in medulloblastoma-derived cancer stem cells	in vitro	(Chang et al., 2012)
CDDO-imidazolide induces DNA damage, G2/M arrest and apoptosis in BRCA1-mutated breast cancer cells.	in vitro	(Kim et al., 2011)
Kalopanaxsaponin A inhibits PMA-induced invasion by reducing matrix metalloproteinase-9 via PI3K/Akt- and PKCδ-mediated signaling in MCF-7 human breast cancer cells	in vitro	(Park et al., 2009)
Frondoside A inhibits breast cancer metastasis and antagonizes prostaglandin E receptors EP4 and EP2	no control group	(Ma et al., 2012)
A new multicolor bioluminescence imaging platform to investigate NF-κB activity and apoptosis in human breast cancer cells.	no control group	(Mezzanotte et al., 2014)
Tea polyphenols inhibit the growth and angiogenesis of breast cancer xenografts in a mouse model	no control group	(Lv et al., 2020)

Natural saponin and cholesterol assembled nanostructures as the promising delivery method for saponin	no triterpene	(D. Wang et al., 2022)
Synthesis and anticancer activity of quinopimaric and maleopimaric acids' derivatives	no triterpene	(Tretyakova et al., 2014)
Rationally designed hecogenin thiosemicarbazone analogs as novel MEK inhibitors for the control of breast malignancies	no triterpene	(Elsayed et al., 2017)
Antitumor activity and mechanism of action of the cyclopenta[b]benzofuran, silvestrol	no triterpene	(Cencic et al., 2009)
Cholesterol biosynthesis inhibitors as potent novel anti-cancer agents: Suppression of hormone-dependent breast cancer by the oxidosqualene cyclase inhibitor RO 48-8071	no triterpene	(Liang et al., 2014)
Cryoablation and meriva have strong therapeutic effect on triple-negative breast cancer	no triterpene	(Chandra et al., 2016)
Identification of triptonide as a therapeutic agent for triple negative breast cancer treatment	no triterpene	(Gao et al., 2021)
Actein Inhibits the Proliferation and Adhesion of Human Breast Cancer Cells and Suppresses Migration in vivo.	other animal species	(Wu et al., 2018)
Inhibition by rosemary and carnosol of 7,12 dimethylbenz[alpha]anthracene (DMBA)-induced rat mammary tumorigenesis and in vivo DMBA-DNA adduct formation	other outcome	(Singletary et al., 1996)
Inotodiol suppresses proliferation of breast cancer in rat model of type 2 diabetes mellitus via downregulation of β-catenin signaling.	other outcome	(X. Zhang et al., 2018)
Raddeanin A suppresses breast cancer-associated osteolysis through inhibiting osteoclasts and breast cancer cells.	other outcome	(Wang et al., 2018)
Ginsenoside Rh2 mitigates DOX-induced cardiotoxicity by inhibiting apoptotic and inflammatory damage and weakening pathological remodelling in breast cancer-bearing mice	other outcome	(Hou et al., 2022)
Evaluating the blood toxicity of functionalized graphene-arginine with anticancer drug ginsenoside Rh2 in Balb/c mouse model with breast cancer	other outcome	(Farhangfar et al., 2022a)
Behavioral studies of mice with breast cancer after treatment with new anticancer agent, Rh2-containing arginine-graphene	other outcome	(Farhangfar et al., 2022c)
Effect of the structure of ginsenosides on the <i>in vivo</i> fate of their liposomes	other outcome	(Chen et al., 2022)
Platycodin D Blocks Breast Cancer-Induced Bone Destruction by Inhibiting Osteoclastogenesis and the Growth of Breast Cancer Cells.	other outcome	(Lee et al., 2015)
Application of bacterial directed enzyme prodrug therapy as a targeted chemotherapy approach in a mouse model of breast cancer	other outcome	(Hosseini-Giv et al., 2021)

Ginsenoside Rg2 Attenuated Trastuzumab-Induced Cardiotoxicity in Rats	other outcome	(Liu et al., 2022)
The combination of the histone deacetylase inhibitor vorinostat and synthetic triterpenoids reduces tumorigenesis in mouse models of cancer	other type of cancer investigated	(Tran et al., 2013)
Maslinic acid solid lipid nanoparticles as hydrophobic anticancer drug carriers: Formulation, in vitro activity and in vivo biodistribution	other type of cancer investigated	(Aguilera-Garrido et al., 2023)
Astragali radix and its main bioactive compounds activate the Nrf2-mediated signaling pathway to induce P-glycoprotein and breast cancer resistance protein	other type of cancer investigated	(Lou et al., 2019)
Inhibitory effects of asiatic acid on 7,12-dimethylbenz[<i>a</i>]anthracene and 12-<i>O</i>-tetradecanoylphorbol 13-acetate-induced tumor promotion in mice	other type of cancer investigated	(Park et al., 2007)
Cucurbitacin B exerts anti-cancer activities in human multiple myeloma cells in vitro and in vivo by modulating multiple cellular pathways	other type of cancer investigated	(Yang et al., 2017)
Ginsenoside Rh1, a novel casein kinase II subunit alpha (CK2α) inhibitor, retards metastasis via disrupting HHEX/CCL20 signaling cascade involved in tumor cell extravasation across endothelial barrier	other type of cancer investigated	(Zheng et al., 2023)
A redox-responsive self-assembling COA-4-arm PEG prodrug nanosystem for dual drug delivery suppresses cancer metastasis and drug resistance by downregulating hsp90 expression	other type of cancer investigated	(Y. Zhou et al., 2023)
H6, a novel hederagenin derivative, reverses multidrug resistance in vitro and in vivo	other type of cancer investigated	(Yanting Yang et al., 2018)
Betulinic acid impairs metastasis and reduces immunosuppressive cells in breast cancer models	other type of cancer investigated	(Zeng et al., 2018)
Ginsenoside Rh1 regulates the immune microenvironment of hepatocellular carcinoma via the glucocorticoid receptor	other type of cancer investigated	(Xiong-hui Wang et al., 2024)
A novel co-drug of aspirin and ursolic acid interrupts adhesion, invasion and migration of cancer cells to vascular endothelium via regulating EMT and EGFR-mediated signaling pathways: multiple targets for cancer metastasis prevention and treatment	other type of cancer investigated	(Tang et al., 2016)
Celastrol inhibits cancer metastasis by suppressing M2-like polarization of macrophages.	other type of cancer investigated	(Yuening Yang et al., 2018)
Inhibition of phosphotidylinositol-3 kinase pathway by a novel naphthol derivative of betulinic acid induces cell cycle arrest and apoptosis in cancer cells of different origin.	other type of cancer investigated	(Majeed et al., 2014)
An exosome-like programmable-bioactivating PTX prodrug nanoplatform for enhanced breast cancer metastasis inhibition	other type of cancer investigated	(Kaiyuan Wang et al., 2020)
Cucurbitacin Q: a selective STAT3 activation inhibitor with potent antitumor activity	other type of cancer investigated	(Sun et al., 2005)

Increased antitumor efficacy of ginsenoside Rh₂ via mixed micelles: <i>in vivo</i> and <i>in vitro</i> evaluation	other type of cancer investigated	(Xia et al., 2020)
Betulinic acid, a bioactive pentacyclic triterpenoid, inhibits skeletal-related events induced by breast cancer bone metastases and treatment.	other type of cancer investigated	(Park et al., 2014)
Ursolic Acid Derivative UA232 Promotes Tumor Cell Apoptosis by Inducing Endoplasmic Reticulum Stress and Lysosomal Dysfunction	other type of cancer investigated	(Gou et al., 2022)
P-gp modulatory acetyl-11-keto-β-boswellic acid based nanoemulsified carrier system for augmented oral chemotherapy of docetaxel	plant extract	(Pandey et al., 2017)
In vitro and in vivo antimammary tumor activities and mechanisms of the apple total triterpenoids.	plant extract	(He et al., 2012)
Ganoderma lucidum total triterpenes induce apoptosis in MCF-7 cells and attenuate DMBA induced mammary and skin carcinomas in experimental animals	plant extract	(Smina et al., 2017)
VEGFR-Mediated Cytotoxic Activity of <i>Pulicaria undulata</i> Isolated Metabolites: A Biological Evaluation and In Silico Study	plant extract	(Elhady et al., 2021)
Anti-tumor activity and related mechanism study of <i>Bacillus Polymyxa </i>transformed<i> Panax ginseng</i> C. A. Mey	plant extract	(Gao et al., 2018)
Anticancer effects of saponin and saponin-phospholipid complex of <i>Panax</i> <i>notoginseng</i> grown in Vietnam	plant extract	(Dang Kim et al., 2016)
Rg3-enriched red ginseng extracts enhance apoptosis in CoCl2-stimulated breast cancer cells by suppressing autophagy	plant extract	(Jeong et al., 2024)
Oleanolic-bioenhancer coloaded chitosan modified nanocarriers attenuate breast cancer cells by multimode mechanism and preserve female fertility.	plant extract	(Sharma et al., 2017)
Inhibition of tumor growth by targeted toxins in mice is dramatically improved by saponinum album in a synergistic way	plant extract	(Bachran et al., 2009)
Non-triggered sequential-release liposomes enhance anti-breast cancer efficacy of STS and celastrol-based microemulsion	plant extract	(Qu et al., 2018)
The dual roles of ginsenosides in improving the anti-tumor efficiency of cyclophosphamide in mammary carcinoma mice	plant extract	(Zhu et al., 2021)

References Table S7

Aguilera-Garrido, A., Graván, P., Navarro-Marchal, S. A., Medina-O'Donnell, M., Parra, A., Gálvez-Ruiz, M. J., Marchal, J. A., & Galisteo-González, F. (2023). Maslinic acid solid lipid nanoparticles as hydrophobic anticancer drug carriers: Formulation, in vitro activity and in vivo biodistribution. *Biomedicine & Pharmacotherapy*, *163*, 114828. https://doi.org/10.1016/j.biopha.2023.114828

Bachran, C., Dürkop, H., Sutherland, M., Bachran, D., Müller, C., Weng, A., Melzig, M. F., & Fuchs, H. (2009). Inhibition of Tumor Growth by Targeted Toxins in Mice is Dramatically Improved by Saponinum Album in a Synergistic Way. *Journal of Immunotherapy*, *32*(7), 713–725. https://doi.org/10.1097/CJI.0b013e3181ad4052

Cencic, R., Carrier, M., Galicia-Vázquez, G., Bordeleau, M.-E., Sukarieh, R., Bourdeau, A., Brem, B., Teodoro, J. G., Greger, H., Tremblay, M. L., Porco, J. A., & Pelletier, J. (2009). Antitumor Activity and Mechanism of Action of the Cyclopenta[b]benzofuran, Silvestrol. *PLoS ONE*, *4*(4), e5223. https://doi.org/10.1371/journal.pone.0005223

Chandra, D., Jahangir, A., Cornelis, F., Rombauts, K., Meheus, L., Jorcyk, C. L., & Gravekamp, C. (2016). Cryoablation and Meriva have strong therapeutic effect on triple-negative breast cancer. *OncoImmunology*, *5*(1), e1049802. https://doi.org/10.1080/2162402X.2015.1049802

Chang, C.-J., Chiang, C.-H., Song, W.-S., Tsai, S.-K., Woung, L.-C., Chang, C.-H., Jeng, S.-Y., Tsai, C.-Y., Hsu, C.-C., Lee, H.-F., Huang, C.-S., Yung, M.-C., Liu, J.-H., & Lu, K.-H. (2012). Inhibition of phosphorylated STAT3 by cucurbitacin I enhances chemoradiosensitivity in medulloblastoma-derived cancer stem cells. *Child's Nervous System*, *28*(3), 363–373. https://doi.org/10.1007/s00381-011-1672-x

Chen, C., Xia, J., Ren, H., Wang, A., Zhu, Y., Zhang, R., Gan, Z., & Wang, J. (2022). Effect of the structure of ginsenosides on the in vivo fate of their liposomes. *Asian Journal of Pharmaceutical Sciences*, *17*(2), 219–229. https://doi.org/10.1016/j.ajps.2021.12.002

Dang Kim, T., Nguyen Thanh, H., Nguyen Thuy, D., Vu Duc, L., Vu Thi, T., Vu Manh, H., Boonsiri, P., & Bui Thanh, T. (2016). Anticancer effects of saponin and saponin–phospholipid complex of Panax notoginseng grown in Vietnam. *Asian Pacific Journal of Tropical Biomedicine*, *6*(9), 795–800. https://doi.org/10.1016/j.apjtb.2016.04.013

Elhady, S. S., Abdelhameed, R. F. A., Zekry, S. H., Ibrahim, A. K., Habib, E. S., Darwish, K. M., Hazem, R. M., Mohammad, K. A., Hassanean, H. A., & Ahmed, S. A. (2021). VEGFR-Mediated Cytotoxic Activity of Pulicaria undulata Isolated Metabolites: A Biological Evaluation and In Silico Study. *Life*, *11*(8), 759. https://doi.org/10.3390/life11080759

Elsayed, H. E., Ebrahim, H. Y., Haggag, E. G., Kamal, A. M., & El Sayed, K. A. (2017). Rationally designed hecogenin thiosemicarbazone analogs as novel MEK inhibitors for the control of breast malignancies. *Bioorganic & Medicinal Chemistry*, *25*(24), 6297–6312. https://doi.org/10.1016/j.bmc.2017.09.033

Farhangfar, S. D., Fesahat, F., Miresmaeili, S. M., & Zare-Zardini, H. (2022). Evaluating the blood toxicity of functionalized graphene-arginine with anticancer drug ginsenoside Rh2 in balb/c mouse model with breast cancer. *Iranian Journal of Pediatric Hematology & Oncology*. https://doi.org/10.18502/ijpho.v12i1.8356

Farhangfar, S. D., Fesahat, F., Zare-Zardini, H., Dehghan-Manshadi, M., Zare, F., Mohsen Miresmaeili, S., Vajihinejad, M., Soltaninejad, H., & Ghorani-Azam, A. (2022). Behavioral studies of mice with breast cancer after treatment with new anticancer agent, Rh2-containing arginine-graphene. *Academic Journal of Health Sciences*, *38*(1), 63–65.

Gao, B., Chen, J., Han, B., Zhang, X., Hao, J., Giuliano, A. E., Cui, Y., & Cui, X. (2021). Identification of triptonide as a therapeutic agent for triple negative breast cancer treatment. *Scientific Reports*, *11*(1), 2408. https://doi.org/10.1038/s41598-021-82128-0

Gao, Y., Liu, J., Ji, Q., Zhao, Y., Zang, P., He, Z., Zhu, H., & Zhang, L. (2018). Anti-tumor activity and related mechanism study of Bacillus Polymyxa transformed Panax ginseng C. A. Mey. *Process Biochemistry*, *72*, 198–208. https://doi.org/10.1016/j.procbio.2018.06.013

Gou, W., Luo, N., Yu, B., Wu, H., Wu, S., Tian, C., Guo, J., Ning, H., Bi, C., Wei, H., Hou, W., & Li, Y. (2022). Ursolic Acid Derivative UA232 Promotes Tumor Cell Apoptosis by Inducing Endoplasmic Reticulum Stress and Lysosomal Dysfunction. *International Journal of Biological Sciences*, *18*(6), 2639–2651. https://doi.org/10.7150/ijbs.67166

He, X., Wang, Y., Hu, H., & Zhang, Z. (2012). In Vitro and in Vivo Antimammary Tumor Activities and Mechanisms of the Apple Total Triterpenoids. *Journal of Agricultural and Food Chemistry*, *60*(37), 9430–9436. https://doi.org/10.1021/jf3026925

Hosseini-Giv, N., Bahrami, A. R., & Matin, M. M. (2021). Application of bacterial directed enzyme prodrug therapy as a targeted chemotherapy approach in a mouse model of breast cancer. *International Journal of Pharmaceutics*, *606*, 120931. https://doi.org/10.1016/j.ijpharm.2021.120931

Hou, J., Yun, Y., Cui, C., & Kim, S. (2022). Ginsenoside Rh2 mitigates doxorubicin-induced cardiotoxicity by inhibiting apoptotic and inflammatory damage and weakening pathological remodelling in breast cancer-bearing mice. *Cell Proliferation*, *55*(6). https://doi.org/10.1111/cpr.13246

Idris, A., Libouban, H., Nyangoga, H., Landao-Bassonga, E., Chappard, D., & Ralston, S. (2008). P52. The IKK inhibitors celastrol and parthenolide inhibit breast cancer cell proliferation and migration in vitro and osteolytic bone metastasis in vivo. *Cancer Treatment Reviews*, *34*, 75. https://doi.org/10.1016/j.ctrv.2008.03.112

Jeong, Y.-J., Yu, M.-H., Cho, Y., Jo, M.-Y., Song, K.-H., Choi, Y. H., Kwon, T. K., Kwak, J.-Y., & Chang, Y.-C. (2024). Rg3-enriched red ginseng extracts enhance apoptosis in CoCl2-stimulated breast cancer cells by suppressing autophagy. *Journal of Ginseng Research*, *48*(1), 31–39. https://doi.org/10.1016/j.jgr.2023.06.001

Kim, E.-H., Deng, C.-X., Sporn, M. B., & Liby, K. T. (2011). CDDO-Imidazolide Induces DNA Damage, G2/M Arrest and Apoptosis in BRCA1-Mutated Breast Cancer Cells. *Cancer Prevention Research*, *4*(3), 425–434. https://doi.org/10.1158/1940-6207.CAPR-10-0153

Lee, S. K., Park, K.-K., Kim, H.-J., Kim, K. R., Kang, E. J., Kim, Y. L., Yoon, H., Kim, Y. S., & Chung, W.-Y. (2015). Platycodin D Blocks Breast Cancer-Induced Bone Destruction by Inhibiting Osteoclastogenesis and the Growth of Breast Cancer Cells. *Cellular Physiology and Biochemistry*, *36*(5), 1809–1820. https://doi.org/10.1159/000430152

Liang, Y., Besch-Williford, C., Aebi, J. D., Mafuvadze, B., Cook, M. T., Zou, X., & Hyder, S. M. (2014). Cholesterol biosynthesis inhibitors as potent novel anti-cancer agents: suppression of hormone-dependent breast cancer by the oxidosqualene cyclase inhibitor RO 48-8071. *Breast Cancer Research and Treatment*, *146*(1), 51–62. https://doi.org/10.1007/s10549-014-2996-5

Liu, G., Zhang, J., Sun, F., Ma, J., & Qi, X. (2022). Ginsenoside Rg2 Attenuated Trastuzumab-Induced Cardiotoxicity in Rats. *BioMed Research International*, *2022*(1). https://doi.org/10.1155/2022/8866660

Lou, Y., Guo, Z., Zhu, Y., Zhang, G., Wang, Y., Qi, X., Lu, L., Liu, Z., & Wu, J. (2019). Astragali radix and its main bioactive compounds activate the Nrf2-mediated signaling pathway to induce P-glycoprotein and breast cancer resistance protein. *Journal of Ethnopharmacology*, *228*, 82–91. https://doi.org/10.1016/j.jep.2018.09.026

Lv, P., Shi, F., Chen, X., Xu, L., Wang, C., Tian, S., Yang, H., & Hou, L. (2020). Tea polyphenols inhibit the growth and angiogenesis of breast cancer xenografts in a mouse model. *Journal of Traditional Chinese Medical Sciences*, *7*(2), 141–147. https://doi.org/10.1016/j.jtcms.2020.05.001

Ma, X., Kundu, N., Collin, P. D., Goloubeva, O., & Fulton, A. M. (2012). Frondoside A inhibits breast cancer metastasis and antagonizes prostaglandin E receptors EP4 and EP2. *Breast Cancer Research and Treatment*, *132*(3), 1001–1008. https://doi.org/10.1007/s10549-011-1675-z

Majeed, R., Hamid, A., Sangwan, P. L., Chinthakindi, P. K., Koul, S., Rayees, S., Singh, G., Mondhe, D. M., Mintoo, M. J., Singh, S. K., Rath, S. K., & Saxena, A. K. (2014). Inhibition of phosphotidylinositol-3 kinase pathway by a novel naphthol derivative of betulinic acid induces cell cycle arrest and apoptosis in cancer cells of different origin. *Cell Death & Disease*, *5*(10), e1459–e1459. https://doi.org/10.1038/cddis.2014.387

Mezzanotte, L., An, N., Mol, I. M., Löwik, C. W. G. M., & Kaijzel, E. L. (2014). A New Multicolor Bioluminescence Imaging Platform to Investigate NF-κB Activity and Apoptosis in Human Breast Cancer Cells. *PLoS ONE*, *9*(1), e85550. https://doi.org/10.1371/journal.pone.0085550

Pandey, G., Mittapelly, N., Valicherla, G. R., Shukla, R. P., Sharma, S., Banala, V. T., Urandur, S., Jajoriya, A. K., Mitra, K., Mishra, D. P., Gayen, J. R., & Mishra, P. R. (2017). P-gp modulatory acetyl-11-keto-β-boswellic acid based nanoemulsified carrier system for augmented oral chemotherapy of docetaxel. *Colloids and Surfaces B: Biointerfaces*, *155*, 276–286. https://doi.org/10.1016/j.colsurfb.2017.04.028

Park, B. C., Paek, S.-H., Lee, Y.-S., Kim, S.-J., Lee, E.-S., Choi, H. G., Yong, C. S., & Kim, J.-A. (2007). Inhibitory Effects of Asiatic Acid on 7,12-Dimethylbenz[a]anthracene and 12-O-Tetradecanoylphorbol 13-Acetate-Induced Tumor Promotion in Mice. *Biological and Pharmaceutical Bulletin*, *30*(1), 176–179. https://doi.org/10.1248/bpb.30.176

Park, S. K., Hwang, Y. S., Park, K.-K., Park, H.-J., Seo, J. Y., & Chung, W.-Y. (2009). Kalopanaxsaponin A inhibits PMA-induced invasion by reducing matrix metalloproteinase-9 via PI3K/Akt- and PKC -mediated signaling in MCF-7 human breast cancer cells. *Carcinogenesis*, *30*(7), 1225–1233. https://doi.org/10.1093/carcin/bgp111

Park, S. Y., Kim, H.-J., Kim, K. R., Lee, S. K., Lee, C. K., Park, K.-K., & Chung, W.-Y. (2014). Betulinic acid, a bioactive pentacyclic triterpenoid, inhibits skeletal-related events induced by breast cancer bone metastases and treatment. *Toxicology and Applied Pharmacology*, *275*(2), 152–162. https://doi.org/10.1016/j.taap.2014.01.009

Qu, D., Wang, L., Qin, Y., Guo, M., Guo, J., Huang, M., Liu, Y., Liu, C., Li, H., & Chen, Y. (2018). Non-triggered sequential-release liposomes enhance anti-breast cancer efficacy of STS and celastrol-based microemulsion. *Biomaterials Science*, *6*(12), 3284–3299. https://doi.org/10.1039/C8BM00796A

REN, Y., YU, K., SUN, S., LI, Z., YUAN, J., HAN, X. D., SHI, J., & ZHEN, L. (2014). JSI124 inhibits breast cancer cell growth by suppressing the function of B cells via the downregulation of signal transducer and activator of transcription 3. *Oncology Letters*, *8*(2), 928–932. https://doi.org/10.3892/ol.2014.2221

Schmiech, M., Ulrich, J., Lang, S. J., Büchele, B., Paetz, C., St-Gelais, A., Syrovets, T., & Simmet, T. (2021). 11-Keto-α-Boswellic Acid, a Novel Triterpenoid from Boswellia spp. with Chemotaxonomic Potential and Antitumor Activity against Triple-Negative Breast Cancer Cells. *Molecules*, *26*(2), 366. https://doi.org/10.3390/molecules26020366

Sharma, M., Sharma, S., Sharma, V., Sharma, K., Yadav, S. K., Dwivedi, P., Agrawal, S., Paliwal, S. K., Dwivedi, A. K., Maikhuri, J. P., Gupta, G., Mishra, P. R., & Rawat, A. K. S. (2017). Oleanolic–bioenhancer coloaded chitosan modified nanocarriers attenuate breast cancer cells by multimode mechanism and preserve female fertility. *International Journal of Biological Macromolecules*, *104*, 1345–1358. https://doi.org/10.1016/j.ijbiomac.2017.06.005

Singletary, K., MacDonald, C., & Wallig, M. (1996). Inhibition by rosemary and carnosol of 7,12-dimethylbenz[a]anthracene (DMBA)-induced rat mammary tumorigenesis and in vivo DMBA-DNA adduct formation. *Cancer Letters*, *104*(1), 43–48. https://doi.org/10.1016/0304-3835(96)04227-9

Smina, T. P., Nitha, B., Devasagayam, T. P. A., & Janardhanan, K. K. (2017). Ganoderma lucidum total triterpenes induce apoptosis in MCF-7 cells and attenuate DMBA induced mammary and skin carcinomas in experimental animals. *Mutation Research/Genetic Toxicology and Environmental Mutagenesis*, *813*, 45–51. https://doi.org/10.1016/j.mrgentox.2016.11.010

Sun, J., Blaskovich, M. A., Jove, R., Livingston, S. K., Coppola, D., & Sebti, S. M. (2005). Cucurbitacin Q: a selective STAT3 activation inhibitor with potent antitumor activity. *Oncogene*, *24*(20), 3236–3245. https://doi.org/10.1038/sj.onc.1208470

Tang, Q., Liu, Y., Li, T., Yang, X., Zheng, G., Chen, H., Jia, L., & Shao, J. (2016). A novel co-drug of aspirin and ursolic acid interrupts adhesion, invasion and migration of cancer cells to vascular endothelium via regulating EMT and EGFR-mediated signaling pathways: multiple targets for cancer metastasis prevention and treatment. *Oncotarget*, *7*(45), 73114–73129. https://doi.org/10.18632/oncotarget.12232

Tran, K., Risingsong, R., B.Royce, D., Williams, C. R., Sporn, M. B., Pioli, P. A., Gediya, L. K., Njar, V. C., & Liby, K. T. (2013). The combination of the histone deacetylase inhibitor vorinostat and synthetic triterpenoids reduces tumorigenesis in mouse models of cancer. *Carcinogenesis*, *34*(1), 199–210. https://doi.org/10.1093/carcin/bgs319

Tretyakova, E. V., Smirnova, I. E., Kazakova, O. B., Tolstikov, G. A., Yavorskaya, N. P., Golubeva, I. S., Pugacheva, R. B., Apryshko, G. N., & Poroikov, V. V. (2014). Synthesis and anticancer activity of quinopimaric and maleopimaric acids' derivatives. *Bioorganic & Medicinal Chemistry*, *22*(22), 6481–6489. https://doi.org/10.1016/j.bmc.2014.09.030

Wang, D., Sha, L., Xu, C., Huang, Y., Tang, C., Xu, T., Li, X., Di, D., Liu, J., & Yang, L. (2022). Natural saponin and cholesterol assembled nanostructures as the promising delivery method for saponin. *Colloids and Surfaces B: Biointerfaces*, *214*, 112448. https://doi.org/10.1016/j.colsurfb.2022.112448

Wang, K., Ye, H., Zhang, X., Wang, X., Yang, B., Luo, C., Zhao, Z., Zhao, J., Lu, Q., Zhang, H., Kan, Q., Wang, Y., He, Z., & Sun, J. (2020). An exosome-like programmable-bioactivating paclitaxel prodrug nanoplatform for enhanced breast cancer metastasis inhibition. *Biomaterials*, *257*, 120224. https://doi.org/10.1016/j.biomaterials.2020.120224

Wang, Q., Mo, J., Zhao, C., Huang, K., Feng, M., He, W., Wang, J., Chen, S., Xie, Z., Ma, J., & Fan, S. (2018). Raddeanin A suppresses breast cancer-associated osteolysis through inhibiting osteoclasts and breast cancer cells. *Cell Death & Disease*, *9*(3), 376. https://doi.org/10.1038/s41419-018-0417-0

Wang, X., Fu, Y., Xu, Y., Zhang, P., Zheng, T., Ling, C., & Feng, Y. (2024). Ginsenoside Rh1 regulates the immune microenvironment of hepatocellular carcinoma via the glucocorticoid receptor. *Journal of Integrative Medicine*, *22*(6), 709–718. https://doi.org/10.1016/j.joim.2024.09.004

Wu, X.-X., Yue, G. G.-L., Dong, J.-R., Lam, C. W.-K., Wong, C.-K., Qiu, M.-H., & Lau, C. B.-S. (2018). Actein Inhibits the Proliferation and Adhesion of Human Breast Cancer Cells and Suppresses Migration in vivo. *Frontiers in Pharmacology*, *9*. https://doi.org/10.3389/fphar.2018.01466

Xia, X., Tao, J., Ji, Z., Long, C., Hu, Y., & Zhao, Z. (2020). Increased antitumor efficacy of ginsenoside Rh 2 via mixed micelles: *in vivo* and *in vitro* evaluation. *Drug Delivery*, *27*(1), 1369–1377. https://doi.org/10.1080/10717544.2020.1825542

Yang, T., Liu, J., Yang, M., Huang, N., Zhong, Y., Zeng, T., Wei, R., Wu, Z., Xiao, C., Cao, X., Li, M., Li, L., Han, B., Yu, X., Li, H., & Zou, Q. (2017). Cucurbitacin B exerts anti-cancer activities in human multiple myeloma cells *in vitro* and *in vivo* by modulating multiple cellular pathways. *Oncotarget*, *8*(4), 5800–5813. https://doi.org/10.18632/oncotarget.10584

Yang, Y., Cheng, S., Liang, G., Honggang, L., & Wu, H. (2018). Celastrol inhibits cancer metastasis by suppressing M2-like polarization of macrophages. *Biochemical and Biophysical Research Communications*, *503*(2), 414–419. https://doi.org/10.1016/j.bbrc.2018.03.224

Yang, Y., Guan, D., Lei, L., Lu, J., Liu, J. Q., Yang, G., Yan, C., Zhai, R., Tian, J., Bi, Y., Fu, F., & Wang, H. (2018). H6, a novel hederagenin derivative, reverses multidrug resistance in vitro and in vivo. *Toxicology and Applied Pharmacology*, *341*, 98–105. https://doi.org/10.1016/j.taap.2018.01.015

Zeng, A.-Q., Yu, Y., Yao, Y.-Q., Yang, F.-F., Liao, M., Song, L.-J., Li, Y.-L., Yu, Y., Li, Y.-J., Deng, Y.-L., Yang, S.-P., Zeng, C.-J., Liu, P., Xie, Y.-M., Yang, J.-L., Zhang, Y.-W., Ye, T.-H., & Wei, Y.-Q. (2018). Betulinic acid impairs metastasis and reduces immunosuppressive cells in breast cancer models. *Oncotarget*, *9*(3), 3794–3804. https://doi.org/10.18632/oncotarget.23376

Zhang, X., Bao, C., & Zhang, J. (2018). Inotodiol suppresses proliferation of breast cancer in rat model of type 2 diabetes mellitus via downregulation of β-catenin signaling. *Biomedicine & Pharmacotherapy*, *99*, 142–150. https://doi.org/10.1016/j.biopha.2017.12.084

Zheng, W., Shen, P., Yu, C., Tang, Y., Qian, C., Yang, C., Gao, M., Wu, Y., Yu, S., Tang, W., Wan, G., Wang, A., Lu, Y., & Zhao, Y. (2023). Ginsenoside Rh1, a novel casein kinase II subunit alpha (CK2α) inhibitor, retards metastasis via disrupting HHEX/CCL20 signaling cascade involved in tumor cell extravasation across endothelial barrier. *Pharmacological Research*, *198*, 106986. https://doi.org/10.1016/j.phrs.2023.106986

Zhou, Y., Miao, Y., Huang, Q., Shi, W., Xie, J., Lin, J., Huang, P., Yue, C., Qin, Y., Yu, X., Wang, H., Qin, L., & Chen, J. (2023). A redox-responsive self-assembling COA-4-arm PEG prodrug nanosystem for dual drug delivery suppresses cancer metastasis and drug resistance by downregulating hsp90 expression. *Acta Pharmaceutica Sinica B*, *13*(7), 3153–3167. https://doi.org/10.1016/j.apsb.2022.11.024

Zhu, H., He, Y.-S., Ma, J., Zhou, J., Kong, M., Wu, C.-Y., Mao, Q., Lin, G., & Li, S.-L. (2021). The dual roles of ginsenosides in improving the anti-tumor efficiency of cyclophosphamide in mammary carcinoma mice. *Journal of Ethnopharmacology*, *265*, 113271. https://doi.org/10.1016/j.jep.2020.113271

Table S8. Triterpenes administered in free form in rodent models of breast cancer

**Triterpene**	**Administration route, dosage, frequency, duration of treatment**	**Controls**	**Tumor weight trend compared with controls**	**Tumor volume trend compared with controls**	**Mechanism of action (in vivo)**	**Safety profile and other effects**	**References**
16a-Tigloyl-O-protoaescigenin	i.v.;1 or 3 mg/kg every other day; five doses	Saline	Decreases	Decreases	Reduces lung metastasis	No pathological injuries were observed	(Yang et al., 2019)

GA	i.p.; 50 mg/kg or 100 mg/kg every other day for 21 days	Saline	Decreases	Decreases	Reduces lung metastasis, activation of JNK1/2 pathway, inhibition of angiogenesis, inhibition of M2 polarization and promotion of M1 polarization	No weight changes	(Cheng et al., 2023)

20(S)-PPD	Injected 2.5; 5 and 10 mg/kg daily for five weeks	Vehicle (sodium carboxymethyl cellulose: CMC-Na)	Decreases (vs vehicle); increases (vs cisplatin)	Decreases (vs vehicle); increases (vs cisplatin)	Reduces lung metastasis, EGFR/MAPK signalling pathway inhibition, decreases ERK1/2, JNK, and p38 MAPK	No significant toxicity	(Peng et al., 2019)
20(S)-PPD	p.o.; 50 or 100 mg/kg daily for 25 days	dimethyl sulfoxide (DMSO)	-	Decreases	Increases levels of phospho-AKT, phospho-mTOR, and Bax; increases Bcl-2 levels	Not significant hepatotoxicity, nephrotoxicity, cardiotoxicity, and other immune organ toxicity	(Zhang et al., 2018)

20(S)-protopanaxatriol	i.g.; 20 mg/kg every other day for 14 days	Saline, 3-methyladenine	Decreases	Decreases	Down-regulation of BCL-2, p62, and Ki-67; up-regulation of Bax and LC3B	No significant difference in the structure of the heart, liver, and kidney tissues	(Li et al., 2021)
25-OCH3-PPD	i.p.; 5 or 20 mg/kg, 5 days per week for 6 weeks (MCF7) or 4 weeks (MDA-MB- 468)	Saline	-	-	Reduces lung metastasis,	No toxicity was observed	(Wang et al., 2012)
AA	p.o.; 25 and 50 mg/kg, daily for 14 days	Vehicle (not specified); docetaxel	Decreases (vs vehicle); increases (vs docetaxel)	Decreases (vs vehicle); increases (vs docetaxel)	-	-	(Gou et al., 2020)

Actein	p.o.; 5 or 15 mg/kg daily for 4 weeks; i.p.; 15 or 20 mg/kg daily for the first 10 days, followed by 15 mg/kg per day for 18 days	phosphate-buffered saline (PBS); trastuzumab	Decreases (vs PBS); increases (vs transtuzumab)	Decreases	Reduces lung, liver and brain metastasis; down-regulation of Cr17; decreases Ki67	Weight lost and 50% mortality (for 20 mg/kg dose)	(Wu et al., 2020)

Actein	p.o.; 10 or 15 mg/kg, for 4 weeks	Vehicle (DMSO); doxorubicin (DOX)	Decreases (vs vehicle); increased (vs DOX)	Decreases (vs vehicle)	Reduces lung and liver metastasis; down-regulation of VEGFR1, Ang2, CXCR4 and AKT; increases IL-2, IL-12 and TNF-α; CD4/CD8 ratio increased in the 15mg/kg actein group	No weight lost	(Yue et al., 2016)
AECHL-1	i.p; 1.5 µg/kg; daily for 10 days	PBS	-	-	Decreases CD44+/CD24− expression	-	(Dasgupta et al., 2016)

AECHL-1	i.p.; 5 µg/kg; daily for 10 days	PBS; paclitaxel (PTX)	Decreases	Decreases	Increases Bax:Bcl2 ratio; decreases levels of pNrf2 and GSH	-	(Sawant et al., 2016)
AG36	i.p.; 0.75, 1.5 and 3.0 mg/kg; every 2 days for 17 days	PBS; cyclophosphamide	Lower doses: decreases (vs PBS), increases (vs cyclophosphamide); 3mg/kg: decreases (vs controls)	Higher doses: decreases (vs PBS), increases (vs cyclophosphamide); 0.75 mg/kg: not significant (vs PBS)	-	No significant effects on body weight, some liver toxicity	(Mu et al., 2017)
Anemoside A3	i.p.; A3 at a dosage of 5 mg/kg, 10 mg/kg, or 20 mg/kg daily for 13 days	Saline; PTX	Decreases (vs saline); increases (vs PTX)	Decreases (vs saline); increases (vs PTX)	Polarization of M1 macrophages, inhibition of TLR4/IL-12/VEGF axis.	-	(Yin et al., 2021)

Arnidiol	i.p.; 40 mg/kg or 80 mg/kg, daily for 70 days	Vehicle (not specified)	-	Decreases	Increases cleaved caspase 3	-	(Hu et al., 2020)
AS-IV	i.p.; 20 mg/kg every 3 days for 6 weeks	DMSO	-	Decreases	Reduces lung metastasis, decreases PCN, MMP-2 and MMP-9	-	(Hu et al., 2021)
AS-IV	i.p.; 20 mg/kg, every 3 Days, for 6 weeks	Hanks' Balanced Salt Solution (HBSS)	-	Decreases	Reduces lung metastasis, downregulating Vav3; Inactivating the Rac1/MAPK signaling pathway	-	(Ke Jiang et al., 2017)

BA	i.p.; 50 and 100 mg/kg; every 3 or 4 days (6 doses)	No treatment; DMSO	-	Decreases	-	-	(Damle et al., 2013)
BA	i.p.; 250 mg/kg, every 2 days for 68 days	Saline	-	Decreases	Decreases LDH-A, c-Myc, PDK1 and Ki67; increases Cav-1 expression	No heart, liver, spleen, lungs and kidney toxicity; no alterations to the hematological parameters	(Jiao et al., 2019)

BA	p.o.; 20 mg/kg every second day for 25 days	DMSO	Decreases	Decreases	Reduces lung metastasis, decreases Sp1, Sp3, and Sp4 protein levels; downregulation of miR-27a, and increases mRNA levels of ZBTB10; Sp-regulated VEGFR	-	(Mertens-Talcott et al., 2013)
BA	p.o.; 50 or 100 mg/kg daily for 6 weeks	No treatment; tamoxifen	-	-	Reduces AFP and CA125 (cancer markers)	-	(Arif et al., 2024)

Bet	p.o.; 20 mg/kg	No treatment	-	-	Inhibition of MAPK expression; decreases AhR, CYP1A1, ARNT, and Keap1; increases Nrf2 and HO-1 expression	No toxicity was observed, no changes in weight	(J. Zhang et al., 2021)
Cel	Injected; 2 or 4 mg/kg every other day during the first week and every third day thereafter	Vehicle ((cremophor formulation-20% Cremophor EL, 30% propylene glycol, 50% ethanol): saline= 1:12.5)	-	-	-	Weight loss; 4/10 mice died in the 4mg/kg treated group	(Raja et al., 2011)
Cel	i.g.; 3 mg/kg; daily for 48 days	Saline	-	Decreases	-	-	(Li et al., 2018)

CK	i.p.; 40 or 80 mg/kg, daily for 4 weeks	Vehicle (a mixture of 87% stroke-physiological saline solution, 8% polyoxyethylene castor oil and 5% ethyl alcohol); PTX	Decreases (vs vehicle); increases (vs PTX)	Decreases (vs vehicle); increases (vs PTX)	Downregulation of GLS1 expression; reduced GSH and aminoacid availability; increases ROS	No significant side toxicity on major organs blood; decreased weight	(B. Zhang et al., 2022)

CuB	i.p.; 1 mg/kg, 3 times a week for 6 weeks	Vehicle (not specified)	Decreases	-	-	Decreased white blood cell count, increased platelet count; decreased glutamic acid pyruvic transaminase level; increased serum uric acid and phosphorus; no significant histopathological differences in liver, spleen, and peritoneum	(Wakimoto et al., 2008)
CuB	i.p.; 0.1 mg/kg and 0.25 mg/kg 3 times a week for 3 weeks	Vehicle (not specified)	Decreases	Decreases	Reduces lung metastasits; decreases VEGF, pFAK, MMP-2, MMP-9, CD31 and PCNA	No toxic effects; no significant changes in the body weight	(Sinha et al., 2016)

CuB	p.o.; 2 mg/kg daily for 55 days (xenograft model) and allograft: i.p.; 1 mg/kg CuB, every third day for 28 days	PBS	Decreases	Decreases	Reduces the expression of HER2, ITGA6 and ITGB4; suppresion of phospho YB-1 (S-102), Twist, Y118 and ILK1	No organ weight differences, LDH decrease	(Gupta and Srivastava, 2014)
CuB	i.p.; 0.5 mg/kg;every other day for 20 days	Saline, vincristine	-	-	Reduces lung metastasis, reduced expression of ROCK, CDC42, and Rac1 proteins	-	(Liang et al., 2019)

Epifriedelinol	i.p.; 100 and 200 mg/kg for 4 months	No treatment	Decreases	Decreases	Decreases CA15-3, CRP, SOD, MDA, TGF-β1 and inflammatory cytokines, decreases PI3K, AKT, mTOR levels and increases Map3k1 expression; decreases caspase-3, BAX and increases Bcl-2	-	(Jing Zhang et al., 2022)

Esculentoside A	p.o.; 10, 20, and 40 mg/kg, 5 times a week for 6 weeks	Saline	Decreases	Decreases	Downregulation of ALDH1A1, Sox2, and Oct4; decreases IL-6, phosphorylated STAT3 (Tyr705), and STAT3 (Ser727); decreases BAX and caspase-3 and increases Bcl-2	No significant toxicity	(Liu et al., 2018)
Frondoside A	i.p.; 100 μg/kg daily for 24 days	Vehicle (not specified)	Decreases	Decreases	-	No toxicity was observed	(Al Marzouqi et al., 2011)
Gypensapogenin H	p.o.; 25 or 50 mg/kg every 3 days for 21 days	Vehicle (not specified)	Decreases	Decreases	Down-regulation of MMP-9, NF-κB, p-IKKα and up-regulation of of I-κBα	No organ toxicity	(Tan et al., 2022)

JSI-124	i.p.; 1 mg/kg daily for 25 days	DMSO	-	Decreases	-	Edema at the injection site (peritoneal cavity); no effects on body weight, activity, or food intake	
OA	p.o.; 40 mg/kg daily for 11 days	Vehicle (corn oil); tamoxifen	Decreases	Decreases	Decreases Tnf-α and Il-6	No significant toxicity	(He et al., 2024)
OA	i.g.; 30 mg/kg daily for 14 days	Vehicle (corn oil)	Decreases	-	Downregulation of THBS1, EDN1, CACNG4, CCN2, AXIN2, BMP4; up-regulation of ATF4, SERPINE1, SESN2, PPARGC1A, EGR1 and JAG1	Weight loss	(Liang et al., 2021)

Panaxadiol	p.o.; 40 mg/kg every 3 days for 21 days	Vehicle (not specified), taxol	Decreases	Decreases	Downregulation of MMP9 and P-p38	No liver and kidney toxicity	(Xu et al., 2021)
Platycodin D	i.p.; 1 and 5 mg/kg, five days per week for 4weeks	Vehicle (PEG400:saline:ethanol=4:3:2)	Decreases	Decreases	Decreases MDM2, MDMX, and mutant p53; increases p21 and p27, downregulation of G0/G1 phase-related proteins	No significant toxicity	(Kong et al., 2016)
Platycodin D	i.p.; 5 mg/kg every 2 days for 20 days	Dulbecco's Phosphate-Buffered Saline (DPBS)	-	Decreases	Decreases MMP-9; inactivating EGFR, MAPK and PI3K/Akt pathways	No toxicity was observed	(Chun and Kim, 2013)

Platycodin D	10 and 20 mg/kg daily for 4 weeks	Distilled water	Decreases	Decreases	Reduces lung metastasis, reduced expression of PD-L1 in neutrophils	Increased GOT levels	(Ye et al., 2023)
Pristimerin	i.p.; MCF-7 group: 2 mg/kg the first day, then 1 mg/kg every other day, 10 doses; MDA-MB-231 group: 1 mg/kg every other day for 12 doses or 0.6 mg/kg for five days/week, 19 doses	DMSO	Decreases	-	Decreases PCNA	No significant toxicity	(Cevatemre et al., 2018)

Pristimerin	i.p.; 0.5 mg/kg; daily for 14 days	DMSO	Decreases	Decreases	Increases cleaved caspase-3, LC-3 II and phosphorylation-JNK inhibition of Trx-1	Mild weight loss, no significant toxicity	(Q. Zhao et al., 2019)
Pygenic acid A	i.v.; 16 μM daily for 18 days	DMSO	-	-	Reduces lung metastasis	-	(Lim et al., 2020)
Ginsenoside Rd (Rd)	i.p.; 1, 3, and 10 mg/kg daily for 4 weeks	DMSO; DOX	Decreases (vs DMSO); increases (1 mg/kg vs DOX); decreases (3 and 10 mg/kg vs DOX)	Decreases (vs DMSO); increases (1 mg/kg vs DOX); decreases (3 and 10 mg/kg vs DOX)	Decreases HIF-1α and CD31 protein levels	No significant toxicity, no significant weight loss	(Zhang et al., 2017)

Rg1	p.o; 10 mg/kg daily for 16 weeks	No treatment	-	Decreases	Decreases SOD, CAT, GPx and GSH; increases TBARS and LOOH; reduces the overexpression of carcinogenesis, angiogenesis, and EMT-related gene expression	-	(Chu et al., 2020)
Rg2	i.p.; 5 mg/kg, every three days for 14 days	PBS; 4-hydroxytamoxifen	Decreases (vs PBS); increases (vs 4-hydroxytamoxifen)	Decreases (vs PBS); increases (vs 4-hydroxytamoxifen)	Downregulation of p-Rb; upregulation of p-AMPK; increases cleaved caspase-3	No significant toxicity	(Jeon et al., 2021)

Rg3	i.p.; 10 mg/kg, every 2 days for 3 weeks	Saline	-	-	Reduces expression of c-Myc, Oct4, Sox2, and Lin28	-	(Ning, 2024)
Rg3	p.o.; 2.5, 5, and 10 mg/kg, five times a week for 3 weeks	β-actin	-	-	Suppression of the STAT3-dependent pathway, tumor-derived cytokines, and the NOTCH signaling pathway	-	(Song et al., 2020)

Rg5	i.p.; 10 mg/kg and 20 mg/kg daily for 30 days	Saline; docetaxel	Decreases (vs saline); increases (10 mg/kg vs docetaxel); similar (20 mg/kg vs docetaxel)	Decreases (vs saline); increases (10 mg/kg vs docetaxel); similar (20 mg/kg vs docetaxel)	Increases Bax, cytochrome-c, caspase-3, caspase-9, PARP 3 and decreases Bcl-2; increases LC3B-II, Atg-5, Atg-7, Atg-12 levels and reduces P62 expression	No significant toxicity	(Liu and Fan, 2018)

Rh1	i.p.; 2 or 5 mg/kg, every three days for 16 days	DMSO; 5-fluorouracil	Decreases	Decreases	Decreases the phosphorylated Rb protein and Akt phosphorilation, increases levels of LC3B, cleaved caspase-3 and ROS	-	(Huynh et al., 2021)
Rh2	i.g.; 5, 10 and 20 mg/kg; every day for 4 weeks	Double distilled water	-	Decreases	Reduces lung metastasis, upregulated MICA expresion; reduces ERp5 expression	No significant toxicity	(C. Yang et al., 2024)
Rh2	p.o.; 2 or 5 mg/kg twice per week for 2 weeks	Vehicle (not specified)	-	-	Increases Bax/Bcl-2 ratio	No significant toxicity	(Choi et al., 2011)

Rh4	i.p.; 10 and 20 mg/kg daily for 25 days	Vehicle (polyethylene glycol (PEG) 400:water: 50%:50%)	-	Decreases	-	-	(Duan et al., 2018)
Rk1	i.p.; 10 or 20 mg/kg daily for 21 days	Vehicle (1% tween-80 in saline), docetaxel	Decreases (vs vehicle); decreases (20mg/kg vs docetaxel); increases (10mg/kg vs docetaxel)	-	-	No significant toxicity	(Hong and Fan, 2019a)
Rk1	10 and 20 mg/kg daily for 3 weeks	Vehicle (not specified), docetaxel	Decreases (vs vehicle); increases (10 mg/kg vs docetaxel); similar (20 mg/kg vs docetaxel)	Decreases (vs vehicle); increases (10 mg/kg vs docetaxel); similar (20 mg/kg vs docetaxel)	Increases cleaved caspase 3, decreases p-Akt level	No significant organ toxicity	(Hong and Fan, 2019b)

Sipholenol A	i.p.; 5 and 10 mg/kg, 3 days a week, for 28 days	DMSO	Decreases	Decreases	Reduces Ki-67 and CD-31, lower levels of p-Brk and p-FAK	No toxicity was observed	(Akl et al., 2014)
Soyasaponin Ag	p.o.; 15 mg/kg, twice a week for 2 weeks	Vehicle (CMC-Na)	Decreases	Decreases	Increases DUSP6 protein level; decreases MAPK1 and MAPK14	-	(Huang et al., 2021)
SsA	i.g.; 35 mg/kg daily for 56 days	Vehicle (CMC-Na), tamoxifen	Decreases (vs CMC-Na), increases (vs tamoxifen)	Decreases (vs CMC-Na), increases (vs tamoxifen)	Decreases Ki-67, increases CD4 cells, IFN-γ and IL-12, activation of IL-12/STAT4 pathway	-	(X. Zhao et al., 2019)

SsA	i.p.; 12 mg/kg once a week for 2 weeks	Vehicle (cremophor El and ethanol, 50% v/v); PTX	-	-	Reduces lung metastasis, reduces CXCR4, phosphorylated Akt, mTOR, MMP9, and MMP2	-	(Y. Wang et al., 2020)
SsD	i.p.; 0.4 or 2.0 mg/kg SsD, every 2 days for 30 days	No treatment	-	-	Reduces lung metastasis,	Reversed splenomegaly, no significant toxicity	(T. Yang et al., 2024)

TSN; ITSN	i.g.; 3, 10 or 30 mg/kg TSN OR ITSN, daily for 5 weeks	Vehicle (CMC-Na), 5-flurouracil	Decreases (vs CMC-Na), increases (3, 10 mg/kg TSN and ITSN vs 5-fluorouracil), decreases (30 mg/kg TSN and ITSN vs 5-fluorouracil)	-	Induces the cleaved activation of caspase-9 and caspase-3, decreased Bcl-xL expression	No toxicity was observed	(Jingnan Zhang et al., 2022).
Tubeismoside-1	i.p; 20 mg/kg every other day for a month	DMSO	-	-	Reduces lung metastasis, reduced levels of CXCR4	-	(Peng et al., 2016)
UA	Injected; 20; 50 and 100 mg/kg	Saline	Decreases	Decreases	Decreases PLK1, p-PLK1, and CCNB1, increases p53	-	(Y. Zhang et al., 2021)

UA	p.o.; 0.05%, 0.10%, or 0.25% (wt/wt in the diet) for 8 weeks (5 post tumor inoculation)	No treatment	Decreases	Decreases, increases (0.05% vs control)	Increases cleaved caspase-3 expression; decreases AKT phosforilation, MCP-1 levels and pS6 expression,	No toxicity was observed, no weight changes	(De Angel et al., 2010)
UA	i.p.; 20 mg/kg daily for 30 days	DMSO, PTX, ferritine, deferoxamine	-	Decreases	Induction of ferroptosis through stabilizing KEAP1 and inhibiting NRF2 activation	-	(X. Yang et al., 2024)

UA	i.p.; 25 and 50 mg/kg daily	Saline	-	Decreases	Reduces glycolysis-associated proteins (LDH-A and c-Myc); increases SP1 and Cav-1 expression	No hepatotoxicity, nephrotoxicity or hematotoxicity	(S. Wang et al., 2021)
UA	i.p.; 20 mg/kg once every 2 days for 4 weeks	Vehicle (not specified)	Decreases	Decreases	Reduces lung metastasis, supression of Wnt, Cell cycle, NFκB, Hypoxia and MAPK/ERK pathways, decreases HIF-1α, VEGF, PCNA, and β-catenin	No toxicity was observed, no weight changes	(Gao et al., 2016)

References Table S8

Akl, M., Foudah, A., Ebrahim, H., Meyer, S., & Sayed, K. (2014). The Marine-Derived Sipholenol A-4-O-3′,4′-Dichlorobenzoate Inhibits Breast Cancer Growth and Motility in Vitro and in Vivo through the Suppression of Brk and FAK Signaling. *Marine Drugs*, *12*(4), 2282–2304. https://doi.org/10.3390/md12042282

Al Marzouqi, N., Iratni, R., Nemmar, A., Arafat, K., Ahmed Al Sultan, M., Yasin, J., Collin, P., Mester, J., Adrian, T. E., & Attoub, S. (2011). Frondoside A inhibits human breast cancer cell survival, migration, invasion and the growth of breast tumor xenografts. *European Journal of Pharmacology*, *668*(1–2), 25–34. https://doi.org/10.1016/j.ejphar.2011.06.023

Arif, R., Bukhari, S. A., Mustafa, G., Ahmed, S., & Albeshr, M. F. (2024). Network Pharmacology and Experimental Validation to Explore the Potential Mechanism of Nigella sativa for the Treatment of Breast Cancer. *Pharmaceuticals*, *17*(5), 617. https://doi.org/10.3390/ph17050617

Blaskovich, M. A., Sun, J., Cantor, A., Turkson, J., Jove, R., & Sebti, S. M. (2003). Discovery of JSI-124 (cucurbitacin I), a selective Janus kinase/signal transducer and activator of transcription 3 signaling pathway inhibitor with potent antitumor activity against human and murine cancer cells in mice. *Cancer Research*, *63*(6), 1270–1279.

Cevatemre, B., Erkısa, M., Aztopal, N., Karakas, D., Alper, P., Tsimplouli, C., Sereti, E., Dimas, K., Armutak, E. I. I., Gurevin, E. G., Uvez, A., Mori, M., Berardozzi, S., Ingallina, C., D'Acquarica, I., Botta, B., Ozpolat, B., & Ulukaya, E. (2018). A promising natural product, pristimerin, results in cytotoxicity against breast cancer stem cells in vitro and xenografts in vivo through apoptosis and an incomplete autopaghy in breast cancer. *Pharmacological Research*, *129*, 500–514. https://doi.org/10.1016/j.phrs.2017.11.027

Cheng, Y., Zhong, X., Nie, X., Gu, H., Wu, X., Li, R., Wu, Y., Lv, K., Leung, G. P.-H., Fu, C., Lee, S. M.-Y., Zhang, J., & Li, J. (2023). Glycyrrhetinic acid suppresses breast cancer metastasis by inhibiting M2-like macrophage polarization via activating JNK1/2 signaling. *Phytomedicine*, *114*, 154757. https://doi.org/10.1016/j.phymed.2023.154757

Choi, S., Oh, J., & Kim, S. (2011). Ginsenoside Rh2 induces Bcl-2 family proteins-mediated apoptosis *in vitro* and in xenografts *in vivo* models. *Journal of Cellular Biochemistry*, *112*(1), 330–340. https://doi.org/10.1002/jcb.22932

Chu, Y., Zhang, W., Kanimozhi, G., Brindha, G. R., & Tian, D. (2020). Ginsenoside Rg1 Induces Apoptotic Cell Death in Triple-Negative Breast Cancer Cell Lines and Prevents Carcinogen-Induced Breast Tumorigenesis in Sprague Dawley Rats. *Evidence-Based Complementary and Alternative Medicine*, *2020*(1). https://doi.org/10.1155/2020/8886955

Chun, J., & Kim, Y. S. (2013). Platycodin D inhibits migration, invasion, and growth of MDA-MB-231 human breast cancer cells via suppression of EGFR-mediated Akt and MAPK pathways. *Chemico-Biological Interactions*, *205*(3), 212–221. https://doi.org/10.1016/j.cbi.2013.07.002

Damle, A. A., Pawar, Y. P., & Narkar, A. A. (2013). Anticancer activity of betulinic acid on MCF-7 tumors in nude mice. *Indian Journal of Experimental Biology*, *51*(7), 485–491.

Dasgupta, A., Sawant, M. A., Kavishwar, G., Lavhale, M., & Sitasawad, S. (2016). AECHL-1 targets breast cancer progression via inhibition of metastasis, prevention of EMT and suppression of Cancer Stem Cell characteristics. *Scientific Reports*, *6*(1), 38045. https://doi.org/10.1038/srep38045

De Angel, R. E., Smith, S. M., Glickman, R. D., Perkins, S. N., & Hursting, S. D. (2010). Antitumor Effects of Ursolic Acid in a Mouse Model of Postmenopausal Breast Cancer. *Nutrition and Cancer*, *62*(8), 1074–1086. https://doi.org/10.1080/01635581.2010.492092

Duan, Z., Wei, B., Deng, J., Mi, Y., Dong, Y., Zhu, C., Fu, R., Qu, L., & Fan, D. (2018). The anti-tumor effect of ginsenoside Rh4 in MCF-7 breast cancer cells in vitro and in vivo. *Biochemical and Biophysical Research Communications*, *499*(3), 482–487. https://doi.org/10.1016/j.bbrc.2018.03.174

Gao, J.-L., Shui, Y.-M., Jiang, W., Huang, E.-Y., Shou, Q.-Y., Ji, X., He, B.-C., Lv, G.-Y., & He, T.-C. (2016). Hypoxia pathway and hypoxia-mediated extensive extramedullary hematopoiesis are involved in ursolic acid's anti-metastatic effect in 4T1 tumor bearing mice. *Oncotarget*, *7*(44), 71802–71816. https://doi.org/10.18632/oncotarget.12375

Gou, X., Bai, H., Liu, L., Chen, H., Shi, Q., Chang, L., Ding, M., Shi, Q., Zhou, M., Chen, W., & Zhang, L. (2020). Asiatic Acid Interferes with Invasion and Proliferation of Breast Cancer Cells by Inhibiting WAVE3 Activation through PI3K/AKT Signaling Pathway. *BioMed Research International*, *2020*(1). https://doi.org/10.1155/2020/1874387

Gupta, P., & Srivastava, S. K. (2014). Inhibition of HER2-integrin signaling by Cucurbitacin B leads to *in vitro* and *in vivo* breast tumor growth suppression. *Oncotarget*, *5*(7), 1812–1828. https://doi.org/10.18632/oncotarget.1743

He, K., Meng, X., Su, J., Jiang, S., Chu, M., & Huang, B. (2024). Oleanolic acid inhibits the tumor progression by regulating Lactobacillus through the cytokine-cytokine receptor interaction pathway in 4T1-induced mice breast cancer model. *Heliyon*, *10*(5), e27028. https://doi.org/10.1016/j.heliyon.2024.e27028

Hong, Y., & Fan, D. (2019a). Ginsenoside Rk1 induces cell cycle arrest and apoptosis in MDA-MB-231 triple negative breast cancer cells. *Toxicology*, *418*, 22–31. https://doi.org/10.1016/j.tox.2019.02.010

Hong, Y., & Fan, D. (2019b). Ginsenoside Rk1 induces cell death through ROS-mediated PTEN/PI3K/Akt/mTOR signaling pathway in MCF-7 cells. *Journal of Functional Foods*, *57*, 255–265. https://doi.org/10.1016/j.jff.2019.04.019

Hu, J., Zhang, H., Li, J., Jiang, X., Zhang, Y., Wu, Q., Shen, liwen, Shi, J., & Gao, N. (2020). ROCK1 activation-mediated mitochondrial translocation of Drp1 and cofilin are required for arnidiol-induced mitochondrial fission and apoptosis. *Journal of Experimental & Clinical Cancer Research*, *39*(1), 37. https://doi.org/10.1186/s13046-020-01545-7

Hu, S., Zheng, W., & Jin, L. (2021). Astragaloside IV inhibits cell proliferation and metastasis of breast cancer via promoting the long noncoding RNA TRHDE-AS1. *Journal of Natural Medicines*, *75*(1), 156–166. https://doi.org/10.1007/s11418-020-01469-8

Huang, S., Huang, P., Wu, H., Wang, S., & Liu, G. (2021). Soyasaponin Ag inhibits triple-negative breast cancer progression via targeting the DUSP6/MAPK signaling. *Folia Histochemica et Cytobiologica*, *59*(4), 291–301. https://doi.org/10.5603/FHC.a2021.0029

Huynh, D. T. N., Jin, Y., Myung, C.-S., & Heo, K.-S. (2021). Ginsenoside Rh1 Induces MCF-7 Cell Apoptosis and Autophagic Cell Death through ROS-Mediated Akt Signaling. *Cancers*, *13*(8), 1892. https://doi.org/10.3390/cancers13081892

Jeon, H., Huynh, D. T. N., Baek, N., Nguyen, T. L. L., & Heo, K.-S. (2021). Ginsenoside-Rg2 affects cell growth via regulating ROS-mediated AMPK activation and cell cycle in MCF-7 cells. *Phytomedicine*, *85*, 153549. https://doi.org/10.1016/j.phymed.2021.153549

Jiang, K., Lu, Q., Li, Q., Ji, Y., Chen, W., & Xue, X. (2017). Astragaloside IV inhibits breast cancer cell invasion by suppressing Vav3 mediated Rac1/MAPK signaling. *International Immunopharmacology*, *42*, 195–202. https://doi.org/10.1016/j.intimp.2016.10.001

Jiao, L., Wang, S., Zheng, Y., Wang, N., Yang, B., Wang, D., Yang, D., Mei, W., Zhao, Z., & Wang, Z. (2019). Betulinic acid suppresses breast cancer aerobic glycolysis via caveolin-1/NF-κB/c-Myc pathway. *Biochemical Pharmacology*, *161*, 149–162. https://doi.org/10.1016/j.bcp.2019.01.016

Kong, Y., Lu, Z.-L., Wang, J.-J., Zhou, R., Guo, J., Liu, J., Sun, H.-L., Wang, H., Song, W., Yang, J., & Xu, H.-X. (2016). Platycodin D, a metabolite of Platycodin grandiflorum, inhibits highly metastatic MDA-MB-231 breast cancer growth in vitro and in vivo by targeting the MDM2 oncogene. *Oncology Reports*, *36*(3), 1447–1456. https://doi.org/10.3892/or.2016.4935

Li, X., Zhu, G., Yao, X., Wang, N., Hu, R., Kong, Q., Zhou, D., Long, L., Cai, J., & Zhou, W. (2018). Celastrol induces ubiquitin-dependent degradation of mTOR in breast cancer cells. *OncoTargets and Therapy*, *Volume 11*, 8977–8985. https://doi.org/10.2147/OTT.S187315

Li, Y., Wang, P., Zou, Z., Pan, Q., Li, X., Liang, Z., Li, L., Lin, Y., Peng, X., Zhang, R., Tian, H., & Han, L. (2021). Ginsenoside (20S)-protopanaxatriol induces non-protective autophagy and apoptosis by inhibiting Akt/mTOR signaling pathway in triple-negative breast cancer cells. *Biochemical and Biophysical Research Communications*, *583*, 184–191. https://doi.org/10.1016/j.bbrc.2021.10.067

Liang, J., Zhang, X., Yuan, J., Zhang, H., Liu, D., Hao, J., Ji, W., Wu, X., & Chen, D. (2019). Cucurbitacin B inhibits the migration and invasion of breast cancer cells by altering the biomechanical properties of cells. *Phytotherapy Research*, *33*(3), 618–630. https://doi.org/10.1002/ptr.6250

Liang, Z., Pan, R., Meng, X., Su, J., Guo, Y., Wei, G., Zhang, Z., & He, K. (2021). Transcriptome study of oleanolic acid in the inhibition of breast tumor growth based on high-throughput sequencing. *Aging*, *13*(19), 22883–22897. https://doi.org/10.18632/aging.203582

Lim, G.-E., Sung, J. Y., Yu, S., Kim, Y., Shim, J., Kim, H. J., Cho, M. L., Lee, J.-S., & Kim, Y.-N. (2020). Pygenic Acid A (PA) Sensitizes Metastatic Breast Cancer Cells to Anoikis and Inhibits Metastasis In Vivo. *International Journal of Molecular Sciences*, *21*(22), 8444. https://doi.org/10.3390/ijms21228444

Liu, C., Dong, L., Sun, Z., Wang, L., Wang, Q., Li, H., Zhang, J., & Wang, X. (2018). Esculentoside A suppresses breast cancer stem cell growth through stemness attenuation and apoptosis induction by blocking IL-6/STAT3 signaling pathway. *Phytotherapy Research*, *32*(11), 2299–2311. https://doi.org/10.1002/ptr.6172

Liu, Y., & Fan, D. (2018). Ginsenoside Rg5 induces apoptosis and autophagy *via* the inhibition of the PI3K/Akt pathway against breast cancer in a mouse model. *Food & Function*, *9*(11), 5513–5527. https://doi.org/10.1039/C8FO01122B

Mertens-Talcott, S. U., Noratto, G. D., Li, X., Angel-Morales, G., Bertoldi, M. C., & Safe, S. (2013). Betulinic acid decreases ER-negative breast cancer cell growth in vitro and in vivo: Role of Sp transcription factors and microRNA-27a:ZBTB10. *Molecular Carcinogenesis*, *52*(8), 591–602. https://doi.org/10.1002/mc.21893

Mu, L.-H., Wang, Y.-N., Wang, D.-X., Zhang, J., Liu, L., Dong, X.-Z., Hu, Y., & Liu, P. (2017). AG36 Inhibits Human Breast Cancer Cells Proliferation by Promotion of Apoptosis In vitro and In vivo. *Frontiers in Pharmacology*, *8*. https://doi.org/10.3389/fphar.2017.00015

Ning, J.-Y., Zhang, Z.-H., Zhang, J., Liu, Y.-M., Li, G.-C., Wang, A.-M., Li, Y., Shan, X., Wang, J.-H., Zhang, X., & Zhao, Y. (2024). Ginsenoside Rg3 decreases breast cancer stem-like phenotypes through impairing MYC mRNA stability. *American Journal of Cancer Research*, *14*(2), 601–615. https://doi.org/10.62347/GYXE7741

Peng, B., He, R., Xu, Q., Yang, Y., Hu, Q., Hou, H., Liu, X., & Li, J. (2019). Ginsenoside 20(S)-protopanaxadiol inhibits triple-negative breast cancer metastasis in vivo by targeting EGFR-mediated MAPK pathway. *Pharmacological Research*, *142*, 1–13. https://doi.org/10.1016/j.phrs.2019.02.003

Peng, Y., Zhong, Y., & Li, G. (2016). Tubeimoside-1 suppresses breast cancer metastasis through downregulation of CXCR4 chemokine receptor expression. *BMB Reports*, *49*(9), 502–507. https://doi.org/10.5483/BMBRep.2016.49.9.030

Raja, S. M., Clubb, R. J., Ortega-Cava, C., Williams, S. H., Bailey, T. A., Duan, L., Zhao, X., Reddi, A. L., Nyong, A. M., Natarajan, A., Band, V., & Band, H. (2011). Anticancer activity of Celastrol in combination with ErbB2-targeted therapeutics for treatment of ErbB2-overexpressing breast cancers. *Cancer Biology & Therapy*, *11*(2), 263–276. https://doi.org/10.4161/cbt.11.2.13959

Sawant, M. A., Dasgupta, A., Lavhale, M. S., & Sitasawad, S. L. (2016). Novel triterpenoid AECHL-1 induces apoptosis in breast cancer cells by perturbing the mitochondria–endoplasmic reticulum interactions and targeting diverse apoptotic pathways. *Biochimica et Biophysica Acta (BBA) - General Subjects*, *1860*(6), 1056–1070. https://doi.org/10.1016/j.bbagen.2016.02.002

Sinha, S., Khan, S., Shukla, S., Lakra, A. D., Kumar, S., Das, G., Maurya, R., & Meeran, S. M. (2016). Cucurbitacin B inhibits breast cancer metastasis and angiogenesis through VEGF-mediated suppression of FAK/MMP-9 signaling axis. *The International Journal of Biochemistry & Cell Biology*, *77*, 41–56. https://doi.org/10.1016/j.biocel.2016.05.014

Song, J.-H., Eum, D.-Y., Park, S.-Y., Jin, Y.-H., Shim, J.-W., Park, S.-J., Kim, M.-Y., Park, S.-J., Heo, K., & Choi, Y.-J. (2020). Inhibitory effect of ginsenoside Rg3 on cancer stemness and mesenchymal transition in breast cancer via regulation of myeloid-derived suppressor cells. *PLOS ONE*, *15*(10), e0240533. https://doi.org/10.1371/journal.pone.0240533

Tan, H., Zhang, M., Xu, L., Zhang, X., & Zhao, Y. (2022). Gypensapogenin H suppresses tumor growth and cell migration in triple-negative breast cancer by regulating PI3K/AKT/NF-κB/MMP-9 signaling pathway. *Bioorganic Chemistry*, *126*, 105913. https://doi.org/10.1016/j.bioorg.2022.105913

Wakimoto, N., Yin, D., O'Kelly, J., Haritunians, T., Karlan, B., Said, J., Xing, H., & Koeffler, H. P. (2008). Cucurbitacin B has a potent antiproliferative effect on breast cancer cells *in vitro* and *in vivo*. *Cancer Science*, *99*(9), 1793–1797. https://doi.org/10.1111/j.1349-7006.2008.00899.x

Wang, S., Chang, X., Zhang, J., Li, J., Wang, N., Yang, B., Pan, B., Zheng, Y., Wang, X., Ou, H., & Wang, Z. (2021). Ursolic Acid Inhibits Breast Cancer Metastasis by Suppressing Glycolytic Metabolism via Activating SP1/Caveolin-1 Signaling. *Frontiers in Oncology*, *11*. https://doi.org/10.3389/fonc.2021.745584

Wang, W., Zhang, X., Qin, J.-J., Voruganti, S., Nag, S. A., Wang, M.-H., Wang, H., & Zhang, R. (2012). Natural Product Ginsenoside 25-OCH3-PPD Inhibits Breast Cancer Growth and Metastasis through Down-Regulating MDM2. *PLoS ONE*, *7*(7), e41586. https://doi.org/10.1371/journal.pone.0041586

Wang, Y., Zhao, L., Han, X., Wang, Y., Mi, J., Wang, C., Sun, D., Fu, Y., Zhao, X., Guo, H., & Wang, Q. (2020). Saikosaponin A Inhibits Triple-Negative Breast Cancer Growth and Metastasis Through Downregulation of CXCR4. *Frontiers in Oncology*, *9*. https://doi.org/10.3389/fonc.2019.01487

Wu, X.-X., Yue, G. G.-L., Dong, J.-R., Lam, C. W.-K., Wong, C.-K., Qiu, M.-H., & Lau, C. B.-S. (2020). Actein Inhibits Tumor Growth and Metastasis in HER2-Positive Breast Tumor Bearing Mice via Suppressing AKT/mTOR and Ras/Raf/MAPK Signaling Pathways. *Frontiers in Oncology*, *10*. https://doi.org/10.3389/fonc.2020.00854

Xu, L., Zhang, X., Xiao, S., Li, X., Jiang, H., Wang, Z., Sun, B., & Zhao, Y. (2021). Panaxadiol as a major metabolite of AD-1 can significantly inhibit the proliferation and migration of breast cancer cells: In vitro and in vivo study. *Bioorganic Chemistry*, *116*, 105392. https://doi.org/10.1016/j.bioorg.2021.105392

Yang, C., Qian, C., Zheng, W., Dong, G., Zhang, S., Wang, F., Wei, Z., Xu, Y., Wang, A., Zhao, Y., & Lu, Y. (2024). Ginsenoside Rh2 enhances immune surveillance of natural killer (NK) cells via inhibition of ERp5 in breast cancer. *Phytomedicine*, *123*, 155180. https://doi.org/10.1016/j.phymed.2023.155180

Yang, T., Li, X., Wang, X., Meng, X., Zhang, Z., Zhao, M., & Su, R. (2024). Combination of histological and metabolomic assessments to evaluate the potential pharmacological efficacy of saikosaponin D. *Journal of Pharmaceutical and Biomedical Analysis*, *242*, 116001. https://doi.org/10.1016/j.jpba.2024.116001

Yang, X., Liang, B., Zhang, L., Zhang, M., Ma, M., Qing, L., Yang, H., Huang, G., & Zhao, J. (2024). Ursolic acid inhibits the proliferation of triple-negative breast cancer stem-like cells through NRF2-mediated ferroptosis. *Oncology Reports*, *52*(1), 94. https://doi.org/10.3892/or.2024.8753

Yang, Y., Long, L., Zhang, X., Song, K., Wang, D., Xiong, X., Gao, H., & Sha, L. (2019). 16-Tigloyl linked barrigenol-like triterpenoid from Semen Aesculi and its anti-tumor activity *in vivo* and *in vitro*. *RSC Advances*, *9*(54), 31758–31772. https://doi.org/10.1039/C9RA06015D

Ye, Y., Xie, Y., Pei, L., Jiang, Z., Wu, C., & Liu, S. (2023). Platycodin D induces neutrophil apoptosis by downregulating PD-L1 expression to inhibit breast cancer pulmonary metastasis. *International Immunopharmacology*, *115*, 109733. https://doi.org/10.1016/j.intimp.2023.109733

Yin, L., Fan, Z., Liu, P., Chen, L., Guan, Z., Liu, Y., & Luo, Y. (2021). Anemoside A3 activates TLR4-dependent M1-phenotype macrophage polarization to represses breast tumor growth and angiogenesis. *Toxicology and Applied Pharmacology*, *432*, 115755. https://doi.org/10.1016/j.taap.2021.115755

Yue, G. G.-L., Xie, S., Lee, J. K.-M., Kwok, H.-F., Gao, S., Nian, Y., Wu, X.-X., Wong, C.-K., Qiu, M.-H., & Lau, C. B.-S. (2016). New potential beneficial effects of actein, a triterpene glycoside isolated from Cimicifuga species, in breast cancer treatment. *Scientific Reports*, *6*(1), 35263. https://doi.org/10.1038/srep35263

Zhang, B., Fu, R., Duan, Z., Shen, S., Zhu, C., & Fan, D. (2022). Ginsenoside CK induces apoptosis in triple-negative breast cancer cells by targeting glutamine metabolism. *Biochemical Pharmacology*, *202*, 115101. https://doi.org/10.1016/j.bcp.2022.115101

Zhang, E., Shi, H., Yang, L., Wu, X., & Wang, Z. (2017). Ginsenoside Rd regulates the Akt/mTOR/p70S6K signaling cascade and suppresses angiogenesis and breast tumor growth. *Oncology Reports*, *38*(1), 359–367. https://doi.org/10.3892/or.2017.5652

Zhang, H., Xu, H.-L., Wang, Y.-C., Lu, Z.-Y., Yu, X.-F., & Sui, D.-Y. (2018). 20(S)-Protopanaxadiol-Induced Apoptosis in MCF-7 Breast Cancer Cell Line through the Inhibition of PI3K/AKT/mTOR Signaling Pathway. *International Journal of Molecular Sciences*, *19*(4), 1053. https://doi.org/10.3390/ijms19041053

Zhang, J., He, Y., Zhou, Y., Hong, L., Jiang, Z., Zhao, Y., & Pan, Z. (2022). Epifriedelinol Ameliorates DMBA-Induced Breast Cancer in Albino Rats by Regulating the PI3K/AKT Pathway. *The Tohoku Journal of Experimental Medicine*, *257*(4), 2022.J030. https://doi.org/10.1620/tjem.2022.J030

Zhang, J., Yang, F., Mei, X., Yang, R., Lu, B., Wang, Z., & Ji, L. (2022). Toosendanin and isotoosendanin suppress triple-negative breast cancer growth via inducing necrosis, apoptosis and autophagy. *Chemico-Biological Interactions*, *351*, 109739. https://doi.org/10.1016/j.cbi.2021.109739

Zhang, J., Zhou, B., Sun, J., Chen, H., & Yang, Z. (2021). Betulin ameliorates 7,12-dimethylbenz(a)anthracene-induced rat mammary cancer by modulating MAPK and AhR/Nrf-2 signaling pathway. *Journal of Biochemical and Molecular Toxicology*, *35*(7). https://doi.org/10.1002/jbt.22779

Zhang, Y., Ma, X., Li, H., Zhuang, J., Feng, F., Liu, L., Liu, C., & Sun, C. (2021). Identifying the Effect of Ursolic Acid Against Triple-Negative Breast Cancer: Coupling Network Pharmacology With Experiments Verification. *Frontiers in Pharmacology*, *12*. https://doi.org/10.3389/fphar.2021.685773

Zhao, Q., Liu, Y., Zhong, J., Bi, Y., Liu, Y., Ren, Z., Li, X., Jia, J., Yu, M., & Yu, X. (2019). Pristimerin induces apoptosis and autophagy via activation of ROS/ASK1/JNK pathway in human breast cancer in vitro and in vivo. *Cell Death Discovery*, *5*(1), 125. https://doi.org/10.1038/s41420-019-0208-0

Zhao, X., Liu, J., Ge, S., Chen, C., Li, S., Wu, X., Feng, X., Wang, Y., & Cai, D. (2019). Saikosaponin A Inhibits Breast Cancer by Regulating Th1/Th2 Balance. *Frontiers in Pharmacology*, *10*. https://doi.org/10.3389/fphar.2019.00624

Table S9. Triterpenes and triterpenoids tested in association with other substances in rodent models of breast cancer

Association tested	Administration route, dosage, frequency, duration of treatment	Controls	Tumor weight trend compared with controls)	Tumor volume trend compared with controls	Mechanism of action (in vivo)	Safety profile	References
AS-IV+ taxol	i.g.; 50 mg/kg AS-IV daily for 21 days + i.p.; 10 mg/kg taxol every 3 days for 21 days	Vehicle (CMC-Na), AS-IV, taxol	-	Decreases	Decreases Cav-1 expression and Ki-67 cells	No significant toxicity, no weight loss	(Zheng et al., 2019)
BA+ taxol	i.p.; 250 mg/kg BA, every 2 days + 10 mg/kg taxol every 3 days for 24 days	Vehicle (not specified), betulinic acid, taxol	-	Decreases	Decreases Ki67 expression and enhanced GRP78 and CHOP levels	Weight loss reported	(Cai et al., 2018)

1-(2-cyano-3,12-dioxooleana-1,9-dien-28-oyl) imidazole (CDDO-Im); CDDO-Im+ tumor necrosis factor-related apoptosis-inducing ligand (TRAIL)	i.p. 5 mg/kg CDDO-Im followed 6 hours later by i.p. 5 mg/kg TRAIL, daily for 14 days	Vehicle (not specified), CDDO-Im, TRAIL	-	Decreases	-	Mild anemia and weight loss	(Hyer et al., 2005)
CDDO-Im+ 1α,25-dihydroxy vitamin D3 (BXL0124)	p.o.; 3 mmol/kg CDDO-Im + 0.3 mg/kg BXL0124, 3 times a week for 41 weeks; p.o.; 3 mmol/kg CDDO-Im + 0.3 mg/kg BXL0124, 6 times a week for 3 weeks	Vehicle (DMSO in sesame oil), BXL0124, CDDO-Im	Decreases (vs vehicle and BXL0124), similar (vs CDDO-Im)	-	Down-regulation of ErbB2 signaling pathway (only in the long-term administration group)	No weight and calcium levels changes	(So et al., 2013)

CDDO-Me+ rexinoid LG100268	p.o.; 15 mg/kg CDDO-Me + 5 mg/kg rexinoid LG100268 for 4 weeks	No treatment	-	-	Angiogenesis inhibition	No significant toxicity	(Liby et al., 2008)
Cel+ tamoxifen	p.o.; 0.5 mg/kg Cel + 5 mg/kg tamoxifen 5 mg/kg for 15 days	Saline, Cel, tamoxifen	Decreases	Decreases	Inhibition of Akt/mTOR pathway	-	(L. Wang et al., 2021)
CuB + higenamine	i.p.; 500 µg/kg CuB + 25 µg/kg higenamine, every other day for 13 days	Saline, CuB, hygenamine	Decreases	Decreases	-	No organ toxicity, no weight loss	(Jin et al., 2018)

CuB+ gemcitabine; CuB+ docetaxel	i.p.; 0.5 and 1 mg/kg CuB +, 3 times per week for 36 days	PBS, CuB, gemcitabine, docetaxel	-	Decreases	Inhibition of STAT3 and NF-κB pathways	No organ toxicity, high-dose CuB+ gemcitabine provoked leukopenia	(Aribi et al., 2013)
Epimers of Rg3 (20(S)-Rg3-SRg3 and 20(R)-Rg3 RRg3)	s.c.; study A:SRg3 (23 mg/kg) + RRg3 (11.5 mg/kg); study B:SRg3 (46 mg/kg) + RRg3 (23 mg/kg), 3 times a week for 40 (study A) or 23 (study B) days	Vehicle (10% DMSO, 20% Cremophor EL 70% DPBS)	-	Decreases	Reduces lung metastasis,	Mild weight loss, fur condition, posture and behaviour	(Nakhjavani et al., 2021)

GA+ DOX	GA: DOX= 20:1 molar ration for 20 days	Saline, GA, DOX	Decreases	-	Inhibition of CD31 and VEGF expression	Reduced cardiotoxicity compared to DOX	(Shi et al., 2021)

Ganoderic acid D+ gemcitabine	i.p.; 50 or 100 mg/kg ganoderic acid, daily + 50 mg/kg gemcitabine, every other day	DMSO; gemcitaine	Decreases	Decreases	Decreases Ki-67, activation of p53/MDM2 ubiquitin-proteasome pathway, HIF-1α degradation, inhibition of LDHA, PKM2, GLUT1, and HK2	No significant toxicity	(B. Luo et al., 2024)

ITSN+ programmed cell death 1 ligand 1 antibody (PD-L1)	i.g.; 0.1 and 1 mg/kg daily for 2 months (MDA-MB-231 ; MDA-MB-231-TGFbR1; MDA-MB-231-shTGFbR1 and BT549 group) and for 1 month (4T1 group)	Vehicle (CMC-Na), ITSN, PD-L1	Decreases	-	Decreases expression of vimentin and a-SMA; enhanced E-cadherin; inhibition of TGFbetaR1	-	(Jingnan Zhang et al., 2023)

OA+ irradiation; OA+ olaparib + irradiation	p.o.; 40 mg/kg OA daily + 50 mg/kg olaparib daily+ a fractional dose of 4 Gy every other day (irradiation), for 7 days	Saline, irradiation	-	Decreases	Down regulation of HIF-1α, Glut-1, survivin and VEGF protein and up-regulation of caspase 3	No significant toxicity	(Xu et al., 2022)
PSD+ camptothecin	i.p. 5 mg/kg of PSD + 3 mg/kg of camptothecin every 2 days	Saline, PSD, camptothecin	-	-	Increases LC3II and p62 proteins, increases cleaved caspase 3 and cleaved PARP	No significant toxicity	(K. Wang et al., 2020)
Rg3+ capecitabine	p.o.; 10 mg/kg Rg3 + 200 mg/kg of capecitabine daily	Saline, Rg3, capecitabine	-	Decreases	Decreases VEGFR	No significant toxicity	(Zhang et al., 2008)

Rg3+ endostatin	s.c.; 5 mg/kg Rg3 + 10 mg/kg endostatin, daily for 10 days	Saline, Rg3, endostatin	-	Decreases	Decreases expression of mTOR, PI3K, Akt, JNK and Beclin-1	-	(Zhang et al., 2016)
Rg3+ PTX	i.p.; 6 mg/kg Rg3 daily for 3 weeks + 10 mg/kg PTX on days 1, 8, and 15.	Saline, Rg3, PTX	Decreases	Decreases	Inhibition of NF-κB pathway, increases Bax/Bcl-2 ratio and caspase-3	-	(Yuan et al., 2017)
Rg3+DOX+ near-infrared radiation (NIR)	i.p.; 6 mg/kg Rg3+ DOX + NIR (1.5 W/cm² for 3 minutes), daily for 21 days	Saline, DOX, NIR	-	Decreases	Decreases Ki67, P-gp and ABCG2	No organ toxicity	(Chang et al., 2024)

Rh2+ DOX	Single Rh2 + ADR group: CMC-Na for 6 days followed by coadministration of Rh2 (50 mg/kg i.g.) and ADR on day 7 for 14 days; Rh2 pretreatment and ADR administration group: Rh2 (50 mg/kg, i.g., daily) for 6 successive days followed by ADR administration on day 7 for 14 days	Vehicle (CMC-Na), Rh2 treatment, Rh2 pretreatment	-	Decreases	-	-	(Liu et al., 2020)

SSd+ DOX	i.p.; 5 mg/kg SSd + 5mg/kg Dox every second day for 3 weeks	Saline, SSD, DOX	Decreases	Decreases	Decreases P-gp expression	-	
SSd+ DOX	i.p.; 5 or 10 mg/kg (divided in 3 days) SSD+ 5 mg/kg DOX for 15 days	Saline, SSD, DOX	Decreases	Decreases	Increase of P-gp, PGC-1α, STAT1, and NQO1 protein levels; decreases expression of γ-H2AX and cleaved caspase3; decreases in Ki-67 positive cells	No weight changes	(F. Luo et al., 2024)

TSN + DOX	Injected; 0.62 mg/kg TSN + 4 mg/kg DOX, every two days for 15 days	DMSO, TSN, DOX	-	Decreases	Downregulation of p-Akt	No significant liver and kidney injury	(Kai et al., 2018)
TSN+ irinotecan (Ir)	i.p.; 0.5 mg/kg TSN, every 2 days + 10 mg/kg Ir, every 6 days for 28 days	Vehicle (intralipid), TSN, Ir	Decreases	Decreases	Increases the levels of LC3-II and SQSTM1, increases cleaved caspase 3-positive cells	No hepatic toxicity	(S. Zhang et al., 2022)
TSN+ PTX	i.p.; 3 mg/kg TSN + 5mg/kg PTX daily for 25 days	Saline, TSN, PTX	Decreases	Decreases	-	No hepatic and renal toxicity	(Juan Zhang et al., 2023)

α-Hederin+ cisplatin	p.o.; 80 mg/kg α-Hederin + p.o.; 4 mg/kg cisplatin, twice a week for 21 days	No treatment, α-Hederin, cisplatin	Decreases	-	Down-regulation of NFκB, TNF-alpha, SDF1, CXCR4 and p-AKT	-	(Elaidy et al., 2023)

References Table S9

Aribi, A., Gery, S., Lee, D. H., Thoennissen, N. H., Thoennissen, G. B., Alvarez, R., Ho, Q., Lee, K., Doan, N. B., Chan, K. T., Toh, M., Said, J. W., & Koeffler, H. P. (2013). The triterpenoid cucurbitacin B augments the antiproliferative activity of chemotherapy in human breast cancer. *International Journal of Cancer*, *132*(12), 2730–2737. https://doi.org/10.1002/ijc.27950

Cai, Y., Zheng, Y., Gu, J., Wang, S., Wang, N., Yang, B., Zhang, F., Wang, D., Fu, W., & Wang, Z. (2018). Betulinic acid chemosensitizes breast cancer by triggering ER stress-mediated apoptosis by directly targeting GRP78. *Cell Death & Disease*, *9*(6), 636. https://doi.org/10.1038/s41419-018-0669-8

Chang, Y., Fu, Q., Lu, Z., Jin, Q., Jin, T., & Zhang, M. (2024). Ginsenoside Rg3 combined with near-infrared photothermal reversal of multidrug resistance in breast cancer MCF-7/ADR cells. *Food Science & Nutrition*, *12*(8), 5750–5761. https://doi.org/10.1002/fsn3.4205

Elaidy, S. M., El-Kherbetawy, M. K., Abed, S. Y., Alattar, A., Alshaman, R., Eladl, M. A., Alamri, E. S., Al balawi, A. N., Zaid, A., Elkazzaz, A. Y., Abdelkhalig, S. M., Hamed, Z. E., & Zaitone, S. A. (2023). α-Hederin Saponin Augments the Chemopreventive Effect of Cisplatin against Ehrlich Tumors and Bioinformatic Approach Identifying the Role of SDF1/CXCR4/p-AKT-1/NFκB Signaling. *Pharmaceuticals*, *16*(3), 405. https://doi.org/10.3390/ph16030405

Hyer, M. L., Croxton, R., Krajewska, M., Krajewski, S., Kress, C. L., Lu, M., Suh, N., Sporn, M. B., Cryns, V. L., Zapata, J. M., & Reed, J. C. (2005). Synthetic Triterpenoids Cooperate with Tumor Necrosis Factor–Related Apoptosis-Inducing Ligand to Induce Apoptosis of Breast Cancer Cells. *Cancer Research*, *65*(11), 4799–4808. https://doi.org/10.1158/0008-5472.CAN-04-3319

Jin, Z.-Q., Hao, J., Yang, X., He, J.-H., Liang, J., Yuan, J.-W., Mao, Y., Liu, D., Cao, R., Wu, X.-Z., Li, X., & Chen, D. (2018). Higenamine enhances the antitumor effects of cucurbitacin B in breast cancer by inhibiting the interaction of AKT and CDK2. *Oncology Reports*. https://doi.org/10.3892/or.2018.6629

Kai, W., Yating, S., Lin, M., Kaiyong, Y., Baojin, H., Wu, Y., Fangzhou, Y., & Yan, C. (2018). Natural product toosendanin reverses the resistance of human breast cancer cells to adriamycin as a novel PI3K inhibitor. *Biochemical Pharmacology*, *152*, 153–164. https://doi.org/10.1016/j.bcp.2018.03.022

Li, C., Xue, H.-G., Feng, L.-J., Wang, M.-L., Wang, P., & Gai, X.-D. (2017). The effect of saikosaponin D on doxorubicin pharmacokinetics and its MDR reversal in MCF-7/adr cell xenografts. *European Review for Medical and Pharmacological Sciences*, *21*(19), 4437–4445.

Liby, K., Risingsong, R., Royce, D. B., Williams, C. R., Yore, M. M., Honda, T., Gribble, G. W., Lamph, W. W., Vannini, N., Sogno, I., Albini, A., & Sporn, M. B. (2008). Prevention and Treatment of Experimental Estrogen Receptor–Negative Mammary Carcinogenesis by the Synthetic Triterpenoid CDDO-Methyl Ester and the Rexinoid LG100268. *Clinical Cancer Research*, *14*(14), 4556–4563. https://doi.org/10.1158/1078-0432.CCR-08-0040

Liu, J., Cai, Q., Wang, W., Lu, M., Liu, J., Zhou, F., Sun, M., Wang, G., & Zhang, J. (2020). Ginsenoside Rh2 pretreatment and withdrawal reactivated the pentose phosphate pathway to ameliorate intracellular redox disturbance and promoted intratumoral penetration of adriamycin. *Redox Biology*, *32*, 101452. https://doi.org/10.1016/j.redox.2020.101452

Luo, B., Song, L., Chen, L., Cai, Y., Zhang, M., & Wang, S. (2024). Ganoderic acid D attenuates gemcitabine resistance of triple-negative breast cancer cells by inhibiting glycolysis via HIF-1α destabilization. *Phytomedicine*, *129*, 155675. https://doi.org/10.1016/j.phymed.2024.155675

Luo, F., Yang, J., Yang, X., Mi, J., Ye, T., Li, G., & Xie, Y. (2024). Saikosaponin D potentiates the antineoplastic effects of doxorubicin in drug-resistant breast cancer through perturbing NQO1-mediated intracellular redox balance. *Phytomedicine*, *133*, 155945. https://doi.org/10.1016/j.phymed.2024.155945

Nakhjavani, M., Smith, E., Palethorpe, H. M., Tomita, Y., Yeo, K., Price, T. J., Townsend, A. R., & Hardingham, J. E. (2021). Anti-Cancer Effects of an Optimised Combination of Ginsenoside Rg3 Epimers on Triple Negative Breast Cancer Models. *Pharmaceuticals*, *14*(7), 633. https://doi.org/10.3390/ph14070633

Shi, J., Li, J., Li, J., Li, R., Wu, X., Gao, F., Zou, L., Mak, W. W. S., Fu, C., Zhang, J., & Leung, G. P.-H. (2021). Synergistic breast cancer suppression efficacy of doxorubicin by combination with glycyrrhetinic acid as an angiogenesis inhibitor. *Phytomedicine*, *81*, 153408. https://doi.org/10.1016/j.phymed.2020.153408

So, J. Y., Wahler, J. E., Yoon, T., Smolarek, A. K., Lin, Y., Shih, W. J., Maehr, H., Uskokovic, M., Liby, K. T., Sporn, M. B., & Suh, N. (2013). Oral Administration of a Gemini Vitamin D Analog, a Synthetic Triterpenoid and the Combination Prevents Mammary Tumorigenesis Driven by ErbB2 Overexpression. *Cancer Prevention Research*, *6*(9), 959–970. https://doi.org/10.1158/1940-6207.CAPR-13-0087

Wang, K., Tu, Y., Wan, J.-B., Chen, M., & He, C. (2020). Synergistic anti-breast cancer effect of pulsatilla saponin D and camptothecin through interrupting autophagic–lysosomal function and promoting p62-mediated ubiquitinated protein aggregation. *Carcinogenesis*, *41*(6), 804–816. https://doi.org/10.1093/carcin/bgz140

Wang, L., Tang, L., Yao, C., Liu, C., & Shu, Y. (2021). The Synergistic Effects of Celastrol in combination with Tamoxifen on Apoptosis and Autophagy in MCF-7 Cells. *Journal of Immunology Research*, *2021*, 1–13. https://doi.org/10.1155/2021/5532269

Xu, A.-L., Xue, Y.-Y., Tao, W.-T., Wang, S.-Q., & Xu, H.-Q. (2022). Oleanolic acid combined with olaparib enhances radiosensitization in triple negative breast cancer and hypoxia imaging with 18F-FETNIM micro PET/CT. *Biomedicine & Pharmacotherapy*, *150*, 113007. https://doi.org/10.1016/j.biopha.2022.113007

Yuan, Z., Jiang, H., Zhu, X., Liu, X., & Li, J. (2017). Ginsenoside Rg3 promotes cytotoxicity of Paclitaxel through inhibiting NF-κB signaling and regulating Bax/Bcl-2 expression on triple-negative breast cancer. *Biomedicine & Pharmacotherapy*, *89*, 227–232. https://doi.org/10.1016/j.biopha.2017.02.038

Zhang, J., Xu, H., Wu, Y., Cho, W. C. S., Xian, Y., & Lin, Z. (2023). Synergistic Anti-Tumor Effect of Toosendanin and Paclitaxel on Triple-Negative Breast Cancer via Regulating ADORA2A-EMT Related Signaling. *Advanced Biology*, *7*(8). https://doi.org/10.1002/adbi.202300062

Zhang, J., Zhang, Z., Huang, Z., Li, M., Yang, F., Wu, Z., Guo, Q., Mei, X., Lu, B., Wang, C., Wang, Z., & Ji, L. (2023). Isotoosendanin exerts inhibition on triple-negative breast cancer through abrogating TGF-β-induced epithelial–mesenchymal transition via directly targeting TGFβR1. *Acta Pharmaceutica Sinica B*, *13*(7), 2990–3007. https://doi.org/10.1016/j.apsb.2023.05.006

Zhang, Q., Kang, X., Yang, B., Wang, J., & Yang, F. (2008). Antiangiogenic Effect of Capecitabine Combined with Ginsenoside Rg3 on Breast Cancer in Mice. *Cancer Biotherapy and Radiopharmaceuticals*, *23*(5), 647–654. https://doi.org/10.1089/cbr.2008.0532

Zhang, S., Dong, Y., Chen, X., TAN, C. S. H., Li, M., Miao, K., & Lu, J.-H. (2022). Toosendanin, a late-stage autophagy inhibitor, sensitizes triple-negative breast cancer to irinotecan chemotherapy. *Chinese Medicine*, *17*(1), 55. https://doi.org/10.1186/s13020-022-00605-8

Zhang, Y., Liu, Q.-Z., Xing, S.-P., & Zhang, J.-L. (2016). Inhibiting effect of Endostar combined with ginsenoside Rg3 on breast cancer tumor growth in tumor-bearing mice. *Asian Pacific Journal of Tropical Medicine*, *9*(2), 180–183. https://doi.org/10.1016/j.apjtm.2016.01.010

Zheng, Y., Dai, Y., Liu, W., Wang, N., Cai, Y., Wang, S., Zhang, F., Liu, P., Chen, Q., & Wang, Z. (2019). Astragaloside IV enhances taxol chemosensitivity of breast cancer via caveolin-1-targeting oxidant damage. *Journal of Cellular Physiology*, *234*(4), 4277–4290. https://doi.org/10.1002/jcp.27196

Table S10. Triterpenes, triterpenoids and associations using carriers in rodent models of breast cancer

**Triterpenes/triterpenoids/ associations**	**Carrier used**	**Administration route, dosage, frequency, duration of treatment**	**Controls**	**Tumor weight trend compared with control**	**Tumor volume trend compared with control**	**Mechanism of action (in vivo)**	**Safety profile**	**References**

AA	NPs: polylactic-co-glycolic acid NPs	-	PBS, AA, empty NPs	-	Decreases	Increases cleaved caspase-3	No renal and hepatic toxicity	(Dutta et al., 2022)
AA+ DOX	Liposomes: non-PEGylated liposomes	i.v.; AA + DOX, every 4 days, 4 doses	PBS, AA, AALip, DOX, DOX + AA, DOX-Lip	Decreases	-	Reduces lung metastasis	-	(Zhang et al., 2024)
BA+ Cel	Liposomes: folic acid-modified liposomes containing Cel and BA micelles	i.v.; 2 mg/kg BA + 2 mg/kg Cel, 14 injections	PBS, BA, Cel	Decreases	Decreases	Reduces lung metastasis	No significant toxicity	(Li et al., 2024)
BA+ PTX	NPs	i.v.; 20 mg/kg BA + 10 mg/kg PTX, on days 0,4,8 and 12	Saline, taxol, PTX-NPs, BA-NPs	-	Decreases	-	Body weight decreased	(Wang et al., 2019)

BA+ taxol	NPs: nanosusspension	i.v.; 10 mg/kg BA + 10 mg/kg taxol (particle size: 700, 400 and 160 nm) every other day for 10 days	Saline, BA, taxol	-	Decreases	Increases Bax/Bcl-2 ratio, decreases MMP-2 and -9	No significant organ toxicity	(R. Wang et al., 2020)
Betulonic acid (BoA)+ PTX	NPs: supramolecular co-assembled NPs	i.v.; 6 mg/kg (PTX equiv.), daily for 10 days	Saline, BoA-NPs, PTX	-	Decreases	-	No significant toxicity, no weight changes, increased serum levels of AST, ALT, and LDH	(J. Wang et al., 2020)
Corosolic acid (CA)+ DOX	Liposomes: cholesterol-free liposomes	i.v.; 5 mg/kg CA, 2,5 mg/kg, every 5 days for 15 days	Saline, CA + DOX, CA-lipo, DOX-lipo	-	Decreases	Reduces lung metastasis, reduces pSTAT3 levels	No organ toxicity	(X. Li et al., 2020)

2-cyano-3,12-dioxooleana-1,9-dien-28-oic acid (CDDO)	Liposomes	i.v.; 20 mg/kg/mouse/d (0.4 mg/mouse/day), 3 times a week for 3 weeks	Empty liposomes, CDDO liposomes	-	Decreases (in MCF-7/NEO), similar (in MCF-7/HER2	Decreases HER2 phosphorilation	-	(Konopleva et al., 2006)
CDDO-Me	Liposomes	i.v.; 200 μg/mouse CDDO-Me at 2-day intervals. CDDO-Me treatment was initiated at either day 1 or day 5 (post tumor inoculation) for 55 days	Empty liposomes	-	-	Reduces lung metastasis, restored splenic mature dendritic cells.	No significant toxicity	(Ling et al., 2007)

Cel	Micelles: Cel loaded glucolipid-like conjugates with avb3-ligand Tetraiodothyroacetic acid modification (TET-CSOSA/Cela)	i.v.; 2 mg/kg TET-CSOSA/Cela every two days, 5 doses	Saline, Cel, Cel loaded glucolipid-like conjugates (CSOSA/Cela)	Decreases	Decreases	Reduces lung metastasis, decreases MMP-9,NF-jB and Bcl-2 expression, upregulation of E-cadherin, angiogenesis inhibition	No significant toxicity	(Zhao et al., 2018)
Cel	Micelles: dual-targeted mixed-micelle	i.v.; 2 mg/kg Cel, every other day for 14 days	Saline, Cel	-	Decreases (vs saline); similar (vs Cel)	-	Weight reduction in the free Cel group	(Huang et al., 2024)

Cel	Micelles: ph responsive micelles	i.v.; 2 mg/kg Cel, every other day for 9 days	Saline, Cel, non ph responsive micelles with Cel	Decreases	Decreases	Increases Bax and caspase 3 expression, decreases Bcl-2 expression, decreases Ki-67 positive cells, enhanced CD8+, decreases CD31, angiogenesis inhibition	No significant organ toxicity, no weight changes	(Tan et al., 2018)
Cel	Micelles: polymeric mixed micelles	i.v.; 2 mg/kg Cel, every 2 days, 5 doses	Saline, Cel, PTX	Decreases	Decreases	-	No organ toxicity	(Gautam et al., 2024)

Cel	NPs	i.v.; 2 mg/kg Cel + ferrocene, daily for 14 days	Saline, Cel, Cel + ferrocene	Decreases	Decreases	Reduces lung metastasis	Weigh loss in free Cel treated group	(Qin et al., 2023)
Cel	NPs: nanosusspension	i.v.; 3 mg/kg Cel, i.g.; 3 mg/kg Cel	Saline, PTX, Cel susspension	-	Decreases (vs saline and Cel susspension), similar (vs PTX)	-	Weight loss	(Huang et al., 2020)
Cel	NPs: self-assembled NPs obtained through anti-solvent method (ethanol or DMSO)	i.v.; 4 mg/kg Cel (solvents used: ethanol E-Cel NPs or DMSO for S-Cel NPs), every 2 days, 5 doses	PBS, Cel	-	Decreases	-	Liver toxicity (Cel group), no toxicity for NPs	(Liu and Li, 2023)

Cel + ferrocene	NPs: pH-sensitive NPs constructed by poly(lactic-co-glycolic acid) modified with β-cyclodextrin, polyethyleneimine grafted with benzimidazole and low molecular weight heparin	i.v.; 2 mg/kg Cel; daily for 3 days	Saline	Decreases	-	Reduces lung metastasis	Weigh loss in free Cel treated group	(Qian et al., 2021)

Cel+ erianin	NPs: self-assembled NPs	i.v.; 2 mg/kg Cel + 1.17 mg/kg erianin, on days 0, 6 and 12	PBS, Cel, erianin	-	Decreases	-	Weight loss, gastrointestinal irritation or damage in Cel treated group, toxicity reduced in NPs treated group	(Tian et al., 2024)
Cel+ Ir	Liposomes: folate-targeted liposomes	i.v.; 3 mg/kg Cel + 3 mg/kg Ir, every 3 days, 4 doses	Saline, Cel, Ir	-	Decreases	Decreases CD31, Ki-67, increases apoptotic markers (caspase-3, PARP)	No significant toxicity, decreased toxicity compared with free Cel	(Soe et al., 2018)

Cel+ Low molecular weight heparin (LMWH) + pulsatilla saponin D (PSN)	NPs: self-assembled NPs	i.v.; 2 mg/kg for Cel + 6 mg/kg LMWH, daily for 7 days	Vehicle (not specified), Cel, LMWH, LMWH-Cel NPs	Decreases	Decreases	-	Liver, heart and kidney toxicity (in Cel group), which was attenuated by the NPs formulation	(Zhou et al., 2023)
Cel+ sulfasalazine	Micelles: magnetically targeted micelles	i.v.; 2 mg/kg Cel + 0.32 mg/kg sulfasalazine; twice a week for 3 weeks	No treatment, DMSO, Cel, sulfasalazine, non-magnetical micelles Cel-sulfasalazine	-	Decreases	Decreases NF-κB , TNF-α, COX-2, Ki-67, VEGF and GSH	No weight changes	(Elhasany et al., 2020)

CuB + laser	NPs: polydopamine NPs (PDA@MB)	i.v.; 15 mg/kg CuB ± laser (for 5 min, 24h after i.v. treatment), single dose	PBS ± laser, polydopamine ± laser, CuB, polydopamine NPs (without CuB) + laser	Decreases (PDA@MB+ laser), similar (PDA@MB- laser vs PBS)	Decreases (PDA@MB + laser), similar (PDA@MB- laser vs PBS)	Reduces lung metastasis	No organ toxicity	(Leng et al., 2022)
Cucurbitacin-E-glucoside	NPs: Ag NPs	P.o.; 28.1 and 70.25 mg/kg for 4 weeks	Distilled water	-	-	Decreases MDA, TNF-alpha and IL-6, increases in SOD, GPx and GSH	Decrased AST, ALT, ALP and LDH levels	(A Ali et al., 2022)
GA+ PTX	NPs: lyophilized self-assembled acid-graft-hyaluronic acid (HGA) NPs	i.v.; 2.2 mg/kg GA + 10 mg/kg PTX+ 22 mg/kg HGA, once in a 4-day interval for 20 days	Saline, GA + PTX+ HGA	Decreases (vs saline), similar (vs GA + PTX9 HGA)	Decreases	-	No significant toxicity	(ZHANG et al., 2015)

GA+ gemcitabine	NPs: multifunctional size-switchable NPs	i.v.; 3.5 mg/kg GA + 3 mg/kg gemcitabine, daily for 10 days	PBS, gemcitabine, Gem NPs	Decreases	Decreases	Decreases Wnt 16, α-SMA	-	(Cun et al., 2019)
Nimbolide	NPs: poly(lactic-co-glycolic acid) (PLGA) NPs	i.v.; 10 and 20 mg/kg Nim, single dose	Dosing solution, nimbolide 10 and 20 mg/kg, empty NPs	Decreases	Decreases	Downregulation of Wnt/b-catenin signaling-associated proteins, Oct4, Sox2, and Nanog, reduces ki-67 positive cells and Beta-catenin	No organ toxicity, no weight loss	(Mohapatra et al., 2023)

OA +10-hydroxycamptothecin (HCPT)	NPs: poly[oligo(ethylene glycol) methyl ethermethacrylate]-b-poly[oleanolic acid methacrylate]	Injected; 10 mg/mL (HCPT equiv.), every two days, 3 doses	Saline; HCPT, OA-NPs	Decreases	Decreases	-	No significant toxicity	(Y.-S. Wang et al., 2020)
OA+ clorine 6 (Ce6)+ photodynamic therapy+ sonodynamic therapy	NPs: self-assembled NPs	i.v.; 1 mg/kg Ce6 equiv ± photodynamic therapy ± sonodynamic therapy, every 2 days for 16 days	Saline, OA-C66 NPs ± photodynamic therapy, OA-Ce6 NPs ± sonodynamic therapy	Decreases	Decreases	-	-	(Y. Zheng et al., 2021)
OA+ Ce6+ Cu_2	NPs: NPs coverd with the membranes of 4T1 cells	i.v.; every other day, 3 doses	PBS	-	Decreases	-	No organ toxicity, no weight changes	(Fu et al., 2023)

OA+ DOX	Micelles: hyaluronic acid-based prodrug nano-micelles	i.v.; 5 mg/kg DOX + OA at an equivalent concentration, every 2 days, 4 doses	Saline, free micelles, DOX, DOX + OA	Decreases	Decreases	-	No significant toxicity, reduced the toxicity of DOX	(Kong et al., 2024)
OA+ DOX	NPs: chitosan based NPs	i.v.; 7.5 mg/kg DOX equiv., every two days, for 30 days	Saline, OA, DOX, OA-loaded NPs	-	Decreases	Upregulation of PARP, PTEN and p53, inhibition of TGF-β, MMP-2 and collagen I (antifibrosis effect)	Mild liver toxicity, no damage observed in the other organs	(Niu et al., 2019)
OA+ DOX	NPs: nanosized crystalsomes containing putrescine	i.v.; 1 mg/kg DOX and 8.4 mg/kg OA, every 3 days for 21 days	Saline, OA, DOX, OA + DOX, crystalomes containing OA and DOX	Decreases	Decreases	Decreases TNF-α, IL-6 and IL-1β	Mild weight loss	(Shukla et al., 2021)

OA+ PTX	NPs	i.v. 25 mg/kg PTX + 135 mg/kg OA, 3 times a week (231WT cells) or single dose (231BR cells) 3 times a week	PBS, OA NPs, PTX	-	Decreases	-	-	(Bao et al., 2020)
Oleanolic quaternary ammonium derivative (QDT)	NPs: chitosan-heparin NPs (HEP/CS NPs)	i.p.; 10, 20 or 40 mg/kg QDT every 2 days for 15 days	Saline and PBS	Decreases	Decreases	Upregulation of caspase-3, caspase-9 and cytochrome C	No significant toxicity	(Chen et al., 2024)

Ginsenoside Rb1 (Rb1)+ 20(S)-PPD+ PTX	NPs: NPs stabilized by Rb1	i.v.; 16 mg/kg equivalent PPD + 8 mg/kg equivalent PTX + Rb1 (stabilizer) every 2 days for seven doses	Saline, PTX, 20(S)-PPD, Rb1-20(S)-PPD NPs	-	Decreases	Upregulation of TNF-α, down-regulation of TGF-β, inhibition of angiogenesis	No significant organ toxicity, mild weight loss	(Lu et al., 2023)
Ginsenoside Rb3 (Rb3)	NPs: carbon nanotubes	Injected; 60 µg/m Rg3-carbone nanotubes	Carbon nanotubes	-	Decreases	Reduces the expression of PD-L1 in tumors	-	(Luo et al., 2021)
Rg3	Micelles: ph-responsive hyperbranched zwitterionic drug-loaded micelles (PPH@CR)	i.v.; 2,8 mg/kg Rg3 , every other day, 6 doses	Saline, curcumin, curcumin + Rg3, PPH@C (micelles)	Decreases	Decreases	Decreases level of Ki-67 and CD44	No significant organ toxicity, no weight loss	(Zhao et al., 2024)

Rg3+ docetaxel	Liposomes: cholesterol-free liposomes	i.v.; Rg3 + 10 mg/kg docetaxel every 4 days for 20 days	PBS, Rg3, docetaxel	Decreases	Decreases	Suppresses TGF-β/Smad signaling, activation of CAFs, increases infiltration of CD4+ and CD8+ T cells, decreases MDSCs and Tregs	No significant toxicity, mild weight loss	(Xia et al., 2022)
Rg3+ DOX+ PD-L1 antibody	NPs: chitosan and cell-penetrating peptide (R6F3)-modified NPs	Peritumorally; 4 mg/kg DOX and Rg3 equivalent, on days 1, 4, 8 and 11	PBS, Rg3 NPs, DOX NPs, PD-L1 NPs, Rg3 NPs+ DOX NPs	Decreases	Decreases	Decreases PD-L1 expression	No significant organ toxicity	(Wu et al., 2022)

Rg3+ PTX	Liposomes: Rg3-based liposomes	i.v.; 40 mg/kg Rg3 + 20 mg/kg PTX, every 3 days for 21 days	Saline, PTX, Rg3 + PTX	Decreases	Decreases	Inhibition of IL-6/STAT3/p-STAT3 pathway, suppression of MDSCs, decreases TAFs, inhibition of angiogenesis	No toxicity observed	(Zhu et al., 2023)
Rg3+ Rb1	NPs	i.v.; 1 mg/kg, based on the concentration of Rg3, single dose	Saline	-	-	Reduces lung metastasis	No organ toxicity	(Zuo et al., 2022)
Rg5	NPs: folic acid-modified ginsenoside Rg5-loaded bovine serum albumin NPs	i.p.; 15 mg/kg Rg5, every day for 3 weeks	BSA, Rg5, Rg5-BSA NPs	Decreases	Decreases	-	No weight loss	(Dong et al., 2019)

Rh2 + PTX	NPs: solid lipid NPs dispersed in a poloxamer-based thermogel	Intratumorally; PS1GS1-Gel: SLNs incorporated in a gel at a 1:1 molar ratio of PTX and GRh2, PS5GS1-Gel: SLNs incorporated in a gel at a 5:1 molar ratio of PTX and GRh2, every three days, 3 doses	No treatment, PS-gel (PTX-SLNs in gel), PTX + Rh2, GS-gel (Rh2-SLNs-in-gel)	-	Decreases (vs no treatment and GS-gel), similar (vs PS-gel and PTX+Rh2	Upregulation of caspase-3, decreases Ki67 cells	Rh2 reduced hemotoxicity induced by PTX	(Long et al., 2024)

Rh2+ PTX	Liposomes: cholesterol-free liposomes	i.v.; 30 mg/kg Rh2, 10 mg/kg PTX, every other day for 21 days	PBS, Lipusu, abraxane, Rh2, Rh2-lipo	-	Decreases	Inhibition of angiogenesis	No significant toxicity	(Hong et al., 2024)
Rh2+ PTX	Liposomes: cholesterol-free liposomes	i.v.; 30 mg/kg Rh2, 10 mg/kg PTX, every other day for 21 days	PBS, Lipusu, Rh2, Rh2-lipo	-	Decreases	The uptake of Rh2-lipo is facilitated by GLUT, antiangiogenic effect, increases CD8+ T cells, decreased G-MDSC and M1 TAM (only in PCX-Rh2 group)	Metastatic and necrotic cells in organs (heart, liver, spleen, kiver, kidney)	(Hong et al., 2020)

Rh2+ squamocin (Squ)+ IR728	NPs: spherical NPs containing Squ, ginsenoside Rh2, and IR780	i.v.; 0.1 mg/kg Squ + 10 mg/kg Rh2+ 2.5 mg/kg IR780 + laser, every 3 days, 5 doses.	Saline, PTX, Squ NPs, Squ-IR780 NPs, Squ-Rh2 NPs, Squ-R848 NPs, R848-IR780 NPs, Rh2-IR780 NPs	-	Decreases	Cytokine production	Significant weight loss (reversible after treatment cessation), mild spleen and liver toxicity (reversible after treatment cessasion)	(Wang et al., 2024)
SsD	NPs: macrophage membrane-biomimetic NPs	i.v.; 1 and or 5 mg/kg, 3 times a week for 4 weeks	Lactic-co-glycolic acid	Decreases	Decreases	Reduces lung and spleen metastasis	No organ toxicity, mild weight loss	(Sun et al., 2020)
UA	Liposomes: long-circulating and pH-sensitive liposomes	i.p.; 23 mg/kg UA daily for 5 days	DMSO, UA, SpHL (empty liposomes)	-	-	Decreases the permeability surface area, no significant cytotoxicity	-	(Rocha et al., 2016)

UA	Liposomes: UA was loaded inside the aqueous space	i.v.; 10 mg/kg UA, every other day, 5 doses	PBS	-	Decreases	Inhibition of STAT5 phosphorylation and IL-10 secretion	No toxicity observed	(Zhang et al., 2020)
UA	NPs: folate-chitosan NPs	i.p.; 12,5 mg/kg UA, daily for 9 days	Saline, UA	Decreases	-	Down-regulation of IFN-γ and TNF-α	-	(Jin et al., 2016)
UA+ DOX	NPs: aptamer-functionalized co-assembled NPs	i.v.; 4 mg/kg UA + 1 mg/kg DOX, every 3 days, 9 doses	PBS, UA, DOX, UA + DOX, UA-DOX NPs (without aptamer functionalization)	Decreases	Decreases	Decreases HER2 expression in tumor tissue	No weight changes	(Kai Jiang et al., 2017)
UA+ HCPT	NPs: self-assembled NPs based on carboxymethylcellulose	i.v.; 10 mg/kg UA + 10 mg/kg HCPT, on days 0, 2, 4, 6 and 8	PBS, HCPT, UA, UA NPs	-	Decreases	-	No significant toxicity	(Liu et al., 2017)

UA+ methotrexate	NPs: carrier-free UA-methotrexate NPs	i.v.; 9 mg/kg UA + 2.25 mg/kg methotrexate, every 2 days, 5 doses	Vehicle (tween 80%), UA + methotrexate	Decreases	Decreases	-	No organ toxicity, no weight changes	(Lan et al., 2021)
UA+ PTX	NPs: hyaluronate NPs	i.v.; 5 mg/kg PTX equiv. at an interval of 4 days for 20 days	Vehicle (not specified), UA, UA-hyaluronate hybrid, PTX, UA + PTX	Decreases	Decreases	-	No significant toxicity	(Sharma et al., 2024)

References Table S10

A Ali, A., A Hussein, M., A Emara, A., Abd Elrahman, A. M., A Hassan, A., A Abdelghaney, A., M Bastawey, A., M Maher, A., N Al-Wadayi, A.-M., A Shalaby, M., M Mohamed, M., Gamal El Din, M. A., A Muhammad, S., S Ewees, A., Nasr-Eldin, M. S., A Alshrief, D., H Mohamed, A., Mostafa, H., El-Ella, A. A., … A Hussein, M. (2022). CEG-AgNPs Ameliorates DMBA-Induced Mammary Carcinogenicity by Alleviating Cytokines Expression. *Pakistan Journal of Biological Sciences : PJBS*, *25*(6), 485–494. https://doi.org/10.3923/pjbs.2022.485.494

Bao, Y., Zhang, S., Chen, Z., Chen, A. T., Ma, J., Deng, G., Xu, W., Zhou, J., Yu, Z.-Q., Yao, G., & Chen, J. (2020). Synergistic Chemotherapy for Breast Cancer and Breast Cancer Brain Metastases via Paclitaxel-Loaded Oleanolic Acid Nanoparticles. *Molecular Pharmaceutics*, *17*(4), 1343–1351. https://doi.org/10.1021/acs.molpharmaceut.0c00044

Chen, K., Zhu, X., Sun, R., Zhao, L., Zhao, J., Wu, X., Wang, C., & Zeng, H. (2024). Oleanolic acid derivative self-assembled aggregates based on heparin and chitosan for breast cancer therapy. *International Journal of Biological Macromolecules*, *277*, 134431. https://doi.org/10.1016/j.ijbiomac.2024.134431

Cun, X., Chen, J., Li, M., He, X., Tang, X., Guo, R., Deng, M., Li, M., Zhang, Z., & He, Q. (2019). Tumor-Associated Fibroblast-Targeted Regulation and Deep Tumor Delivery of Chemotherapeutic Drugs with a Multifunctional Size-Switchable Nanoparticle. *ACS Applied Materials & Interfaces*, *11*(43), 39545–39559. https://doi.org/10.1021/acsami.9b13957

Dong, Y., Fu, R., Yang, J., Ma, P., Liang, L., Mi, Y., & Fan, D. (2019). <p>Folic acid-modified ginsenoside Rg5-loaded bovine serum albumin nanoparticles for targeted cancer therapy in vitro and in vivo</p>. *International Journal of Nanomedicine*, *Volume 14*, 6971–6988. https://doi.org/10.2147/IJN.S210882

Dutta, S., Chakraborty, P., Basak, S., Ghosh, S., Ghosh, N., Chatterjee, S., Dewanjee, S., & Sil, P. C. (2022). Synthesis, characterization, and evaluation of in vitro cytotoxicity and in vivo antitumor activity of asiatic acid-loaded poly lactic-co-glycolic acid nanoparticles: A strategy of treating breast cancer. *Life Sciences*, *307*, 120876. https://doi.org/10.1016/j.lfs.2022.120876

Elhasany, K. A., Khattab, S. N., Bekhit, A. A., Ragab, D. M., Abdulkader, M. A., Zaky, A., Helmy, M. W., Ashour, H. M. A., Teleb, M., Haiba, N. S., & Elzoghby, A. O. (2020). Combination of magnetic targeting with synergistic inhibition of NF-κB and glutathione via micellar drug nanomedicine enhances its anti-tumor efficacy. *European Journal of Pharmaceutics and Biopharmaceutics*, *155*, 162–176. https://doi.org/10.1016/j.ejpb.2020.08.004

Fu, S., Wang, M., Li, B., Li, X., Cheng, J., Zhao, H., Zhang, H., Dong, A., Lu, W., & Yang, X. (2023). Bionic natural small molecule co-assemblies towards targeted and synergistic Chemo/PDT/CDT. *Biomaterials Research*, *27*(1). https://doi.org/10.1186/s40824-023-00380-z

Gautam, S., Singh, N., Marwaha, D., Rai, N., Sharma, M., Tiwari, P., Singh, S., Kumar Bakshi, A., Kumar, A., Agarwal, N., Prakash Shukla, R., & Ranjan Mishra, P. (2024). Celastrol-loaded polymeric mixed micelles shows improved antitumor efficacy in 4 T1 bearing xenograft mouse model through spatial targeting. *International Journal of Pharmaceutics*, *659*, 124234. https://doi.org/10.1016/j.ijpharm.2024.124234

Hong, C., Liang, J., Xia, J., Zhu, Y., Guo, Y., Wang, A., Lu, C., Ren, H., Chen, C., Li, S., Wang, D., Zhan, H., & Wang, J. (2020). One Stone Four Birds: A Novel Liposomal Delivery System Multi-functionalized with Ginsenoside Rh2 for Tumor Targeting Therapy. *Nano-Micro Letters*, *12*(1), 129. https://doi.org/10.1007/s40820-020-00472-8

Hong, C., Wang, A., Xia, J., Liang, J., Zhu, Y., Wang, D., Zhan, H., Feng, C., Jiang, X., Pan, J., & Wang, J. (2024). Ginsenoside Rh2-Based Multifunctional Liposomes for Advanced Breast Cancer Therapy. *International Journal of Nanomedicine*, *Volume 19*, 2879–2888. https://doi.org/10.2147/IJN.S437733

Huang, S., Xiao, S., Li, X., Tao, R., Yang, Z., Gao, Z., Hu, J., Meng, Y., Zheng, G., & Chen, X. (2024). Development of Dual-Targeted Mixed Micelles Loaded with Celastrol and Evaluation on Triple-Negative Breast Cancer Therapy. *Pharmaceutics*, *16*(9), 1174. https://doi.org/10.3390/pharmaceutics16091174

Huang, T., Wang, Y., Shen, Y., Ao, H., Guo, Y., Han, M., & Wang, X. (2020). Preparation of high drug-loading celastrol nanosuspensions and their anti-breast cancer activities in vitro and in vivo. *Scientific Reports*, *10*(1), 8851. https://doi.org/10.1038/s41598-020-65773-9

Jiang, K., Han, L., Guo, Y., Zheng, G., Fan, L., Shen, Z., Zhao, R., & Shao, J. (2017). A carrier-free dual-drug nanodelivery system functionalized with aptamer specific targeting HER2-overexpressing cancer cells. *Journal of Materials Chemistry B*, *5*(46), 9121–9129. https://doi.org/10.1039/C7TB02562A

Jin, H., Pi, J., Yang, F., Jiang, J., Wang, X., Bai, H., Shao, M., Huang, L., Zhu, H., Yang, P., Li, L., Li, T., Cai, J., & Chen, Z. W. (2016). Folate-Chitosan Nanoparticles Loaded with Ursolic Acid Confer Anti-Breast Cancer Activities in vitro and in vivo. *Scientific Reports*, *6*(1), 30782. https://doi.org/10.1038/srep30782

Kong, F., Liu, H., Zhao, C., & Qin, J. (2024). Targeted codelivery of doxorubicin and oleanolic acid by reduction responsive hyaluronic acid-based prodrug nano-micelles for enhanced antitumor activity and reduced toxicity. *International Journal of Biological Macromolecules*, *277*, 134135. https://doi.org/10.1016/j.ijbiomac.2024.134135

Konopleva, M., Zhang, W., Shi, Y.-X., McQueen, T., Tsao, T., Abdelrahim, M., Munsell, M. F., Johansen, M., Yu, D., Madden, T., Safe, S. H., Hung, M.-C., & Andreeff, M. (2006). Synthetic triterpenoid 2-cyano-3,12-dioxooleana-1,9-dien-28-oic acid induces growth arrest in HER2-overexpressing breast cancer cells. *Molecular Cancer Therapeutics*, *5*(2), 317–328. https://doi.org/10.1158/1535-7163.MCT-05-0350

Lan, J.-S., Qin, Y.-H., Liu, L., Zeng, R.-F., Yang, Y., Wang, K., Ding, Y., Zhang, T., & Ho, R. J. (2021). A Carrier-Free Folate Receptor-Targeted Ursolic Acid/Methotrexate Nanodelivery System for Synergetic Anticancer Therapy. *International Journal of Nanomedicine*, *Volume 16*, 1775–1787. https://doi.org/10.2147/IJN.S287806

Leng, J., Dai, X., Cheng, X., Zhou, H., Wang, D., Zhao, J., Ma, K., Cui, C., Wang, L., & Guo, Z. (2022). Biomimetic Cucurbitacin B-Polydopamine Nanoparticles for Synergistic Chemo-Photothermal Therapy of Breast Cancer. *Frontiers in Bioengineering and Biotechnology*, *10*. https://doi.org/10.3389/fbioe.2022.841186

Li, C., Wang, Z., Zhang, Y., Zhu, Y., Xu, M., Lei, H., & Zhang, D. (2024). Efficient Sequential Co-Delivery Nanosystem for Inhibition of Tumor and Tumor-Associated Fibroblast-Induced Resistance and Metastasis. *International Journal of Nanomedicine*, *Volume 19*, 1749–1766. https://doi.org/10.2147/IJN.S427783

Li, X., Widjaya, A. S., Liu, J., Liu, X., Long, Z., & Jiang, Y. (2020). Cell-penetrating corosolic acid liposome as a functional carrier for delivering chemotherapeutic drugs. *Acta Biomaterialia*, *106*, 301–313. https://doi.org/10.1016/j.actbio.2020.02.013

Ling, X., Konopleva, M., Zeng, Z., Ruvolo, V., Stephens, L. C., Schober, W., McQueen, T., Dietrich, M., Madden, T. L., & Andreeff, M. (2007). The Novel Triterpenoid C-28 Methyl Ester of 2-Cyano-3, 12-Dioxoolen-1, 9-Dien-28-Oic Acid Inhibits Metastatic Murine Breast Tumor Growth through Inactivation of STAT3 Signaling. *Cancer Research*, *67*(9), 4210–4218. https://doi.org/10.1158/0008-5472.CAN-06-3629

Liu, Y., & Li, J. (2023). Self-assembling nanoarchitectonics of size-controllable celastrol nanoparticles for efficient cancer chemotherapy with reduced systemic toxicity. *Journal of Colloid and Interface Science*, *636*, 216–222. https://doi.org/10.1016/j.jcis.2022.12.162

Liu, Y., Liu, K., Li, C., Wang, L., Liu, J., He, J., Lei, J., & Liu, X. (2017). Self-assembled nanoparticles based on a carboxymethylcellulose–ursolic acid conjugate for anticancer combination therapy. *RSC Advances*, *7*(58), 36256–36268. https://doi.org/10.1039/C7RA05913B

Long, J., Hu, W., Ren, T., Wang, X., Lu, C., Pan, X., Wu, C., & Peng, T. (2024). Combating multidrug resistance of breast cancer with ginsenoside Rh2-irrigated nano-in-thermogel. *International Journal of Pharmaceutics*, *650*, 123718. https://doi.org/10.1016/j.ijpharm.2023.123718

Lu, L., Ao, H., Fu, J., Li, M., Guo, Y., Guo, Y., Han, M., Shi, R., & Wang, X. (2023). Ginsenoside Rb1 stabilized and paclitaxel / protopanaxadiol co-loaded nanoparticles for synergistic treatment of breast tumor. *Biomedicine & Pharmacotherapy*, *163*, 114870. https://doi.org/10.1016/j.biopha.2023.114870

Luo, X., Wang, H., & Ji, D. (2021). Carbon nanotubes (CNT)-loaded ginsenosides Rb3 suppresses the PD-1/PD-L1 pathway in triple-negative breast cancer. *Aging*, *13*(13), 17177–17189. https://doi.org/10.18632/aging.203131

Mohapatra, P., Madhulika, S., Behera, S., Singh, P., Sa, P., Prasad, P., Swain, R. K., & Sahoo, S. K. (2023). Nimbolide-based nanomedicine inhibits breast cancer stem-like cells by epigenetic reprogramming of DNMTs-SFRP1-Wnt/β-catenin signaling axis. *Molecular Therapy Nucleic Acids*, *34*, 102031. https://doi.org/10.1016/j.omtn.2023.102031

Niu, S., Williams, G. R., Wu, J., Wu, J., Zhang, X., Zheng, H., Li, S., & Zhu, L.-M. (2019). A novel chitosan-based nanomedicine for multi-drug resistant breast cancer therapy. *Chemical Engineering Journal*, *369*, 134–149. https://doi.org/10.1016/j.cej.2019.02.201

Qian, Y., Zhang, J., Xu, R., Li, Q., Shen, Q., & Zhu, G. (2021). Nanoparticles based on polymers modified with pH-sensitive molecular switch and low molecular weight heparin carrying Celastrol and ferrocene for breast cancer treatment. *International Journal of Biological Macromolecules*, *183*, 2215–2226. https://doi.org/10.1016/j.ijbiomac.2021.05.204

Qin, Y., Wang, Z., Wang, X., Zhang, T., Hu, Y., Wang, D., Sun, H., Zhang, L., & Zhu, Y. (2023). Therapeutic effect of multifunctional celastrol nanoparticles with mitochondrial alkaline drug release in breast cancer. *Materials Today Advances*, *17*, 100328. https://doi.org/10.1016/j.mtadv.2022.100328

Rocha, T. G. R., Lopes, S. C. de A., Cassali, G. D., Ferreira, Ê., Veloso, E. S., Leite, E. A., Braga, F. C., Ferreira, L. A. M., Balvay, D., Garofalakis, A., Oliveira, M. C., & Tavitian, B. (2016). Evaluation of Antitumor Activity of Long-Circulating and pH-Sensitive Liposomes Containing Ursolic Acid in Animal Models of Breast Tumor and Gliosarcoma. *Integrative Cancer Therapies*, *15*(4), 512–524. https://doi.org/10.1177/1534735416628273

Sharma, R., Yadav, V., Jha, S., Dighe, S., & Jain, S. (2024). Unveiling the potential of ursolic acid modified hyaluronate nanoparticles for combination drug therapy in triple negative breast cancer. *Carbohydrate Polymers*, *338*, 122196. https://doi.org/10.1016/j.carbpol.2024.122196

Shukla, R. P., Urandur, S., Banala, V. T., Marwaha, D., Gautam, S., Rai, N., Singh, N., Tiwari, P., Shukla, P., & Mishra, P. R. (2021). Development of putrescine anchored nano-crystalsomes bearing doxorubicin and oleanolic acid: deciphering their role in inhibiting metastatic breast cancer. *Biomaterials Science*, *9*(5), 1779–1794. https://doi.org/10.1039/D0BM01033B

Soe, Z. C., Thapa, R. K., Ou, W., Gautam, M., Nguyen, H. T., Jin, S. G., Ku, S. K., Oh, K. T., Choi, H.-G., Yong, C. S., & Kim, J. O. (2018). Folate receptor-mediated celastrol and irinotecan combination delivery using liposomes for effective chemotherapy. *Colloids and Surfaces B: Biointerfaces*, *170*, 718–728. https://doi.org/10.1016/j.colsurfb.2018.07.013

Sun, K., Yu, W., Ji, B., Chen, C., Yang, H., Du, Y., Song, M., Cai, H., Yan, F., & Su, R. (2020). Saikosaponin D loaded macrophage membrane-biomimetic nanoparticles target angiogenic signaling for breast cancer therapy. *Applied Materials Today*, *18*, 100505. https://doi.org/10.1016/j.apmt.2019.100505

Tan, Y., Zhu, Y., Zhao, Y., Wen, L., Meng, T., Liu, X., Yang, X., Dai, S., Yuan, H., & Hu, F. (2018). Mitochondrial alkaline pH-responsive drug release mediated by Celastrol loaded glycolipid-like micelles for cancer therapy. *Biomaterials*, *154*, 169–181. https://doi.org/10.1016/j.biomaterials.2017.07.036

Tian, J., Chen, K., Zhang, Q., Qiu, C., Tong, H., Huang, J., Hao, M., Chen, J., Zhao, W., Wong, Y.-K., Gao, L., Luo, P., Wang, J., & Du, Q. (2024). Mechanism of Self-Assembled Celastrol-Erianin Nanomedicine for treatment of breast cancer. *Chemical Engineering Journal*, *499*, 155709. https://doi.org/10.1016/j.cej.2024.155709

Wang, J., Qiao, W., Zhao, H., & Yang, X. (2020). Paclitaxel and betulonic acid synergistically enhance antitumor efficacy by forming co-assembled nanoparticles. *Biochemical Pharmacology*, *182*, 114232. https://doi.org/10.1016/j.bcp.2020.114232

Wang, R., Wang, X., Jia, X., Wang, H., Li, W., & Li, J. (2020). Impacts of particle size on the cytotoxicity, cellular internalization, pharmacokinetics and biodistribution of betulinic acid nanosuspensions in combined chemotherapy. *International Journal of Pharmaceutics*, *588*, 119799. https://doi.org/10.1016/j.ijpharm.2020.119799

Wang, R., Yang, M., Li, G., Wang, X., Zhang, Z., Qiao, H., Chen, J., Chen, Z., Cui, X., & Li, J. (2019). Paclitaxel-betulinic acid hybrid nanosuspensions for enhanced anti-breast cancer activity. *Colloids and Surfaces B: Biointerfaces*, *174*, 270–279. https://doi.org/10.1016/j.colsurfb.2018.11.029

Wang, X., Liu, X., Guo, Y., Gong, T., Lu, W., Han, M., Guo, Y., & Wang, X. (2024). The Remarkable Anti-Breast Cancer Efficacy and Anti-Metastasis by Multifunctional Nanoparticles Co-Loading Squamocin, R848 and IR 780. *International Journal of Nanomedicine*, *Volume 19*, 4679–4699. https://doi.org/10.2147/IJN.S448860

Wang, Y.-S., Li, G.-L., Zhu, S.-B., Jing, F.-C., Liu, R.-D., Li, S.-S., He, J., & Lei, J.-D. (2020). A Self-assembled Nanoparticle Platform Based on Amphiphilic Oleanolic Acid Polyprodrug for Cancer Therapy. *Chinese Journal of Polymer Science*, *38*(8), 819–829. https://doi.org/10.1007/s10118-020-2401-2

Wu, H., Wei, G., Luo, L., Li, L., Gao, Y., Tan, X., Wang, S., Chang, H., Liu, Y., Wei, Y., Song, J., Zhang, Z., & Huo, J. (2022). Ginsenoside Rg3 nanoparticles with permeation enhancing based chitosan derivatives were encapsulated with doxorubicin by thermosensitive hydrogel and anti-cancer evaluation of peritumoral hydrogel injection combined with PD-L1 antibody. *Biomaterials Research*, *26*(1). https://doi.org/10.1186/s40824-022-00329-8

Xia, J., Zhang, S., Zhang, R., Wang, A., Zhu, Y., Dong, M., Ma, S., Hong, C., Liu, S., Wang, D., & Wang, J. (2022). Targeting therapy and tumor microenvironment remodeling of triple-negative breast cancer by ginsenoside Rg3 based liposomes. *Journal of Nanobiotechnology*, *20*(1), 414. https://doi.org/10.1186/s12951-022-01623-2

ZHANG, L., ZHOU, J.-P., & YAO, J. (2015). Improved anti-tumor activity and safety profile of a paclitaxel-loaded glycyrrhetinic acid-graft-hyaluronic acid conjugate as a synergistically targeted drug delivery system. *Chinese Journal of Natural Medicines*, *13*(12), 915–924. https://doi.org/10.1016/S1875-5364(15)30097-2

Zhang, N., Liu, S., Shi, S., Chen, Y., Xu, F., Wei, X., & Xu, Y. (2020). Solubilization and delivery of Ursolic-acid for modulating tumor microenvironment and regulatory T cell activities in cancer immunotherapy. *Journal of Controlled Release*, *320*, 168–178. https://doi.org/10.1016/j.jconrel.2020.01.015

Zhang, Y., Wang, Y., Zhang, H., Huang, S., Li, Y., Long, J., Han, Y., Lin, Q., Gong, T., Sun, X., Zhang, Z., & Zhang, L. (2024). Replacing cholesterol with asiatic acid to prolong circulation and enhance anti-metastatic effects of non-PEGylated liposomes. *Journal of Controlled Release*, *366*, 585–595. https://doi.org/10.1016/j.jconrel.2024.01.009

Zhao, Y., Bai, Y., Li, M., Nie, X., Meng, H., Shosei, S., Liu, L., Yang, Q., Shen, M., & Li, Y. (2024). A pH-triggered N-oxide polyzwitterionic nano-drug loaded system for the anti-tumor immunity activation research. *Journal of Nanobiotechnology*, *22*(1), 420. https://doi.org/10.1186/s12951-024-02677-0

Zhao, Y., Tan, Y., Meng, T., Liu, X., Zhu, Y., Hong, Y., Yang, X., Yuan, H., Huang, X., & Hu, F. (2018). Simultaneous targeting therapy for lung metastasis and breast tumor by blocking the NF-κB signaling pathway using Celastrol-loaded micelles. *Drug Delivery*, *25*(1), 341–352. https://doi.org/10.1080/10717544.2018.1425778

Zheng, Y., Li, Z., Yang, Y., Shi, H., Chen, H., & Gao, Y. (2021). A nanosensitizer self-assembled from oleanolic acid and chlorin e6 for synergistic chemo/sono-photodynamic cancer therapy. *Phytomedicine*, *93*, 153788. https://doi.org/10.1016/j.phymed.2021.153788

Zhou, M., Liao, J., Lai, W., Xu, R., Liu, W., Xie, D., Wang, F., Zhang, Z., Huang, J., Zhang, R., & Li, G. (2023). A celastrol-based nanodrug with reduced hepatotoxicity for primary and metastatic cancer treatment. *EBioMedicine*, *94*, 104724. https://doi.org/10.1016/j.ebiom.2023.104724

Zhu, Y., Wang, A., Zhang, S., Kim, J., Xia, J., Zhang, F., Wang, D., Wang, Q., & Wang, J. (2023). Paclitaxel-loaded ginsenoside Rg3 liposomes for drug-resistant cancer therapy by dual targeting of the tumor microenvironment and cancer cells. *Journal of Advanced Research*, *49*, 159–173. https://doi.org/10.1016/j.jare.2022.09.007

Zuo, S., Wang, J., An, X., Wang, Z., Zheng, X., & Zhang, Y. (2022). Fabrication of Ginsenoside-Based Nanodrugs for Enhanced Antitumor Efficacy on Triple-Negative Breast Cancer. *Frontiers in Bioengineering and Biotechnology*, *10*. https://doi.org/10.3389/fbioe.2022.945472
